# Supplementary material for: Post-infection sequelae of COVID-19 and other infectious diseases—a nationwide Danish study with 40-month follow-up
Source: Nat Commun. 2026 Mar 13;17:3894. doi: 10.1038/s41467-026-70351-0 (PMC13125258; doi:10.1038/s41467-026-70351-0)
Supplement: Supplementary file 1 — Supplementary Information [file 41467_2026_70351_MOESM1_ESM.pdf]

Study population **4**

Supplementary Figure 1: Schematic visualizations of the study populations and analyses. *4*

Supplementary Table 1: Number of individuals in the study population by SARS-CoV-2 test status and outcome. *5*

Characteristics of the study populations **6**

Supplementary Table 2: Study population assessing any outcome by SARS-CoV-2 test status. *6*

Supplementary Table 3: Study population assessing mental disorder outcomes by SARS-CoV-2 test status. *8*

Supplementary Table 4: Study population assessing general medical condition outcomes by SARS-CoV-2 test status. *10*

Supplementary Figure 2: Time-varying visualization of the study population assessing any mental disorder or general medical condition outcomes. *12*

Supplementary Table 5: Study population assessing any outcome by COVID-19 admission status without and with intensive care unit (ICU) admission. *13*

Results: Positive compared with negative and with no SARS-CoV-2 test **15**

Supplementary Table 6: Positive, negative, and no SARS-CoV-2 tests compared with each other. *15*

Results: Positive compared with negative SARS-CoV-2 test **16**

Supplementary Table 7: Age group. *16*

Supplementary Figure 3: Hazard ratios of first specific general medical conditions by age group and severity of COVID-19. .... *18*

Supplementary Table 8: Number of positive tests. *20*

Supplementary Table 9: Time since test. *21*

Supplementary Table 10: Calendar period. *23*

Results: Positive test compared with other infections **27**

Supplementary Table 11: Positive test compared with prescription for anti-infective agents. *27*

Results: Admission with COVID-19 compared with no admission with COVID-19 .... **29**

Supplementary Table 12: Intensive care unit (ICU) status. *29*

Supplementary Table 13: Age group. *30*

Supplementary Table 14: Number of admissions. *33*

Supplementary Table 15: Duration of admission. *34*

Supplementary Table 16: Calendar period. *35*

Results: Admission with COVID-19 compared with other infections **39**

Supplementary Table 17: Admission with COVID-19 compared with admissions with non-COVID-19 infections. *39*

Supplementary Table 18: Admission with COVID-19 compared with admission with non-COVID-19 pulmonary infection. *41*

Results: Positive SARS-CoV-2 test by peak C-Reactive Protein (CRP) **43**

Supplementary Table 19: Positive or negative test and CRP value. *43*

Supplementary Table 20: CRP value. *46*

Supplementary Figure 4: Density, hazard ratios, and confidence intervals of the association between peak C-reactive Protein (CRP), admission status and mental disorders or general medical conditions. *50*

Supplementary Table 21: CRP value and age group. *51*

Supplementary Figure 5: CRP value and age group. *56*

Supplementary Table 22: CRP value and age group (unadjusted rates and rate ratios). *57*

Supplementary Figure 6: CRP value and age group (unadjusted rates and rate ratios). *58*

Results: Admission with COVID-19 and peak C-Reactive Protein (CRP) **59**

Supplementary Table 23: CRP value. *59*

Supplementary Table 24: CRP value in quantiles *61*

Supplementary Table 25: CRP value and age group. *64*

Supplementary Figure 7: CRP value and age group. *66*

Supplementary Table 26: CRP and age group (unadjusted rates and rate ratios). *67*

Supplementary Figure 8: CRP value and age group (unadjusted rates and rate ratios). *68*

Sensitivity Analysis: Positive compared with negative SARS-CoV-2 test **69**

Sensitivity analysis 1: Various levels of adjusting for confounders. *69*

Sensitivity analysis 2: Number of conducted SARS-CoV-2 tests. *70*

Sensitivity analysis 3: Virus variants. *71*

Sensitivity analysis 4: Lockdown. *72*

Sensitivity analysis 5: Vaccination status. *73*

Sensitivity analysis 6: Immigration status. *74*

Sensitivity analysis 7: Excluding individuals with hospital contacts within five years of study start only. *75*

Sensitivity Analysis: Positive compared with negative SARS-CoV-2 test (secondary outcomes) **76**

Sensitivity analysis 1 (secondary outcomes): Various levels of adjusting for confounders. *76*

Sensitivity analysis 2 (secondary outcomes): Number of conducted SARS-CoV-2 tests. *77*

Sensitivity analysis 3 (secondary outcomes): Virus variants. *78*

Sensitivity analysis 4 (secondary outcomes): Lockdown. *79*

Sensitivity analysis 5 (secondary outcomes): Vaccination status. *80*

Sensitivity analysis 6 (secondary outcomes): Immigration status. *81*

Sensitivity analysis 7 (secondary outcomes): Excluding individuals with hospital contacts within five years of study start only. *82*

Sensitivity Analysis: Admission with COVID-19 compared with no admission with COVID-19 .... **83**

Sensitivity analysis 8: Virus variants. *83*

Sensitivity analysis 9: Lockdown. *84*

Sensitivity analysis 10: Vaccination status. *85*

Sensitivity analysis 11: Immigration status. *86*

Sensitivity analysis 12: Excluding individuals with hospital contacts within five years of study start only. *87*

Sensitivity Analysis: Admission with COVID-19 compared with no admission with COVID-19 (secondary outcomes) **88**

Sensitivity analysis 8 (secondary outcomes): Virus variants. *88*

Sensitivity analysis 9 (secondary outcomes): Lockdown. *90*

Sensitivity analysis 10 (secondary outcomes): Vaccination status. *92*

Sensitivity analysis 11 (secondary outcomes): Immigration status. *93*

Sensitivity analysis 12 (secondary outcomes): Excluding individuals with hospital contacts within five years of study start only. *94*

Supplementary Methods 95

Supplementary Definitions 97

Supplementary Table 27: Disorders categorized by ICD-8, and ICD-10 codes. 97

Supplementary Table 28: Charlson Comorbidity Index categorized by ICD-8 and ICD-10 codes. 99

Supplementary Table 29: Definition of variables. 100

Supplementary Table 30: Definition of exposures. 101

Supplementary Table 31: Anti-infective agents categorized by ATC codes. 102

Supplementary Table 32: Infection codes categorized by ICD-10 codes. 102

Supplementary Table 33: Dominating virus variant periods. 103

Supplementary Table 34: Lockdown periods. 103

Supplementary Figure 9: Timeline of lockdown and periods of dominating virus variants. 103

STROBE statement: Checklist of items that should be included in reports of cohort studies 104

References 106

Study population

Supplementary Figure 1: Schematic visualizations of the study populations and analyses.

a Study population and primary analysis.

b Secondary analyses.

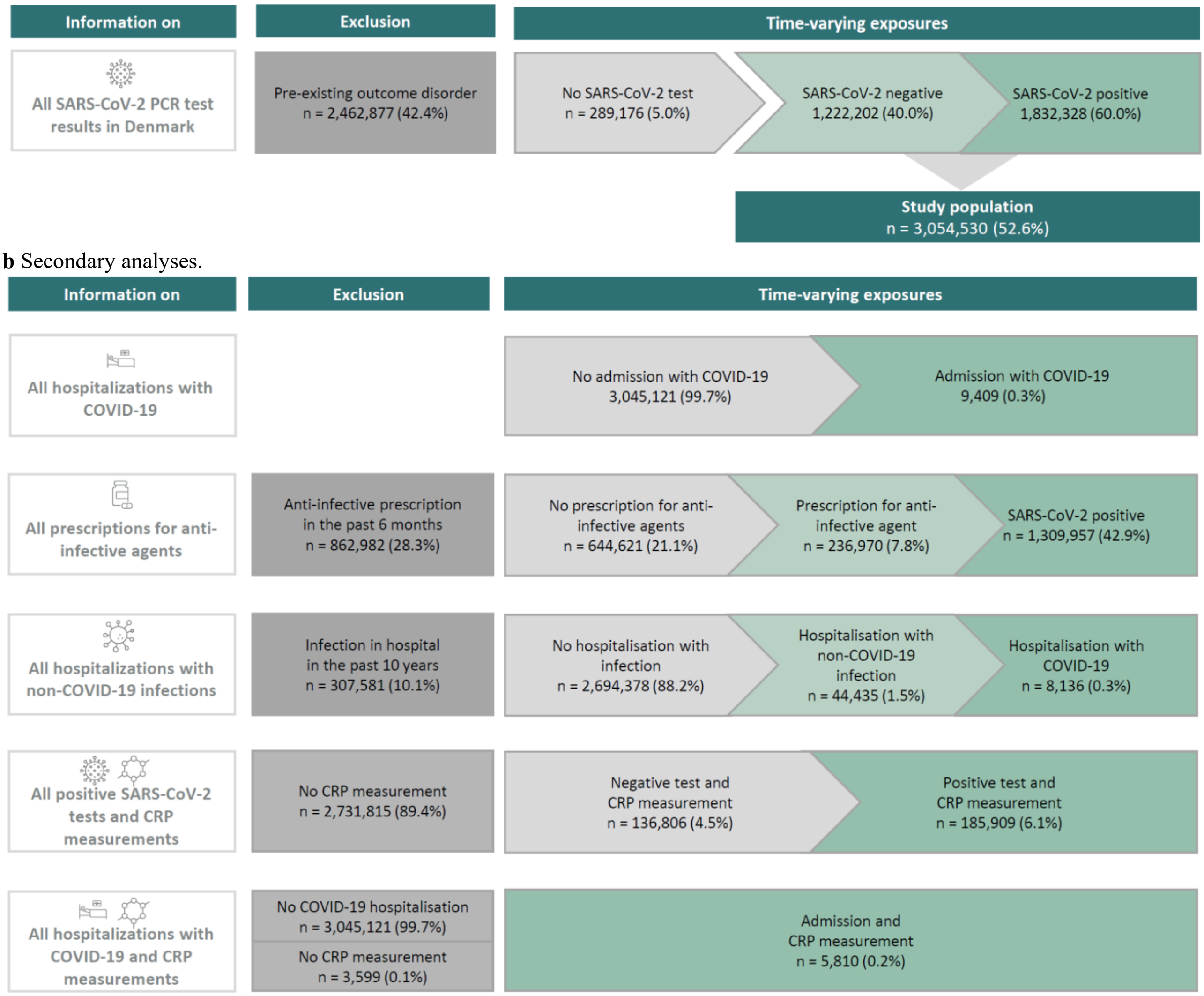

Supplementary Table 1: Number of individuals in the study population by SARS-CoV-2 test status and outcome.

Outcome	Total	SARS-CoV-2 positive[a]	SARS-CoV-2 negative[b]	No SARS-CoV-2 test[c]
Mental disorders or general medical conditions	3,343,706 (100%)	1,832,328 (54.8%)	1,222,202 (36.6%)	289,176 (8.6%)
Mental disorders	5,306,132 (100%)	2,837,134 (53.5%)	2,059,213 (38.8%)	409,785 (7.7%)
General medical conditions	3,517,630 (100%)	1,931,527 (54.9%)	1,289,639 (36.7%)	296,464 (8.4%)
Neurological disorders	4,696,886 (100%)	2,550,658 (54.3%)	1,772,844 (37.7%)	373,384 (7.9%)
Respiratory disorders	5,328,640 (100%)	2,829,420 (53.1%)	2,086,350 (39.2%)	412,870 (7.7%)
Circulatory disorders	4,893,928 (100%)	2,710,089 (55.4%)	1,821,784 (37.2%)	362,055 (7.4%)
Kidney disorders	5,744,041 (100%)	3,064,596 (53.4%)	2,251,002 (39.2%)	428,443 (7.5%)
Gastrointestinal disorders	5,346,048 (100%)	2,870,389 (53.7%)	2,072,810 (38.8%)	402,849 (7.5%)
Endocrine disorders	5,607,407 (100%)	3,011,572 (53.7%)	2,181,515 (38.9%)	414,320 (7.4%)
Hematological disorders	5,673,717 (100%)	3,029,165 (53.4%)	2,220,065 (39.1%)	424,487 (7.5%)
Musculoskeletal disorders	5,658,912 (100%)	3,004,372 (53.1%)	2,226,444 (39.3%)	428,096 (7.6%)
Dermatological disorders	5,701,216 (100%)	3,024,249 (53%)	2,247,859 (39.4%)	429,108 (7.5%)
Neuropsychiatric symptoms	5,549,505 (100%)	2,958,639 (53.3%)	2,166,720 (39%)	424,146 (7.6%)

Table description: The exposure groups, *SARS-CoV-2 positive SARS-CoV-2 test*, *SARS-CoV-2 negative SARS-CoV-2 test*, and *no SARS-CoV-2 test*, were identified by end of follow up.

[a] The group *SARS-CoV-2 positive SARS-CoV-2 test* consisted of everyone with at least one positive test by the end of follow up.

[b] The group *SARS-CoV-2 negative SARS-CoV-2 test* consisted of everyone with at least one negative and no positive test by the end of follow up.

[c] The group *No SARS-CoV-2 test* consisted of everyone without any SARS-CoV-2 test result by the end of follow up.

Characteristics of the study populations

Supplementary Table 2: Study population assessing any outcome by SARS-CoV-2 test status.

	Total	SARS-CoV-2 positive[a]	SARS-CoV-2 negative[b]	No SARS-CoV-2 test[c]
**Total at end of follow-up (% by row)**	3,343,706 (100%)	1,832,328 (54.8%)	1,222,202 (36.6%)	289,176 (8.6%)
**Mental disorders or general medical conditions at end of follow up (% by column)**				
Yes	353,517 (10.6%)	83,232 (4.5%)	183,105 (15.0%)	87,180 (30.1%)
No	2,990,189 (89.4%)	1,749,096 (95.5%)	1,039,097 (85.0%)	201,996 (69.9%)
**Time of Inclusion in Exposure Group (% by column)**				
2020, March – 2020, June	455,117 (13.6%)	6,061 (0.3%)	159,880 (13.1%)	289,176 (100.0%)
2020, July – 2020, December	783,132 (23.4%)	94,146 (5.1%)	688,986 (56.4%)	0 (0.0%)
2021, January – 2021, June	311,007 (9.3%)	75,930 (4.1%)	235,077 (19.2%)	0 (0.0%)
2021, July – 2021, December	424,387 (12.7%)	324,873 (17.7%)	99,514 (8.1%)	0 (0.0%)
2022, January – 2022, June	1,305,213 (39.0%)	1,270,742 (69.4%)	34,471 (2.8%)	0 (0.0%)
2022, July – 2022, December	60,859 (1.8%)	57,632 (3.1%)	3,227 (0.3%)	0 (0.0%)
2023, January – 2023, June	3,991 (0.1%)	2,944 (0.2%)	1,047 (0.1%)	0 (0.0%)
	**No. individuals (% by column) by the start of follow up**			
**Mean Age (SD)**	33.405 (21.249)	29.556 (18.978)	36.515 (22.064)	44.653 (24.890)
**Age group**				
<18	968,789 (29.0%)	629,574 (34.4%)	291,468 (23.8%)	47,747 (16.5%)
18-29	655,452 (19.6%)	367,554 (20.1%)	240,654 (19.7%)	47,244 (16.3%)
30-39	446,540 (13.4%)	270,855 (14.8%)	145,799 (11.9%)	29,886 (10.3%)
40-49	441,909 (13.2%)	253,815 (13.9%)	159,171 (13.0%)	28,923 (10.0%)
50-59	397,926 (11.9%)	186,165 (10.2%)	174,129 (14.2%)	37,632 (13.0%)
60-69	251,883 (7.5%)	86,919 (4.7%)	123,210 (10.1%)	41,754 (14.4%)
70-79	146,234 (4.4%)	33,023 (1.8%)	72,394 (5.9%)	40,817 (14.1%)
≥80	34,973 (1.0%)	4,423 (0.2%)	15,377 (1.3%)	15,173 (5.2%)
**Sex**				
Female	1,636,491 (48.9%)	931,419 (50.8%)	577,952 (47.3%)	127,120 (44.0%)
Male	1,707,215 (51.1%)	900,909 (49.2%)	644,250 (52.7%)	162,056 (56.0%)
**Mean Duration in years (SD)**	3.094 (0.691)	3.286 (0.224)	3.009 (0.751)	2.244 (1.352)
**Charlson Comorbidity Index**				
0	3,179,604 (95.1%)	1,764,015 (96.3%)	1,148,963 (94.0%)	266,626 (92.2%)
1	48,459 (1.4%)	22,918 (1.3%)	19,825 (1.6%)	5,716 (2.0%)
2	96,762 (2.9%)	38,857 (2.1%)	44,240 (3.6%)	13,665 (4.7%)
3+	18,881 (0.6%)	6,538 (0.4%)	9,174 (0.8%)	3,169 (1.1%)
**Parental Mental disorder**				
Yes	549,050 (16.4%)	308,305 (16.8%)	200,394 (16.4%)	40,351 (14.0%)
No	2,794,656 (83.6%)	1,524,023 (83.2%)	1,021,808 (83.6%)	248,825 (86.0%)
**Parental Charlson Comorbidity Index**				
0	1,845,671 (55.2%)	1,001,788 (54.7%)	660,974 (54.1%)	182,909 (63.3%)
1	416,121 (12.4%)	247,283 (13.5%)	144,171 (11.8%)	24,667 (8.5%)
2	405,900 (12.1%)	233,357 (12.7%)	147,215 (12.0%)	25,328 (8.8%)
3+	676,014 (20.2%)	349,900 (19.1%)	269,842 (22.1%)	56,272 (19.5%)
**Education**				
No education	752,891 (22.5%)	469,234 (25.6%)	233,868 (19.1%)	49,789 (17.2%)
Primary school	694,666 (20.8%)	364,955 (19.9%)	254,704 (20.8%)	75,007 (25.9%)
Vocational training or gymnasium	976,466 (29.2%)	497,275 (27.1%)	384,548 (31.5%)	94,643 (32.7%)
Higher education, short cycle	519,627 (15.5%)	288,320 (15.7%)	191,336 (15.7%)	39,971 (13.8%)
Higher education, long cycle	400,056 (12.0%)	212,544 (11.6%)	157,746 (12.9%)	29,766 (10.3%)
**Employment status**				
Kids and Education	1,254,228 (37.6%)	796,435 (43.5%)	397,351 (32.6%)	60,442 (21.2%)
Employed	1,682,738 (50.4%)	919,006 (50.2%)	636,752 (52.2%)	126,980 (44.5%)
Not in workforce	75,350 (2.3%)	31,491 (1.7%)	31,434 (2.6%)	12,425 (4.4%)
Retired	293,936 (8.8%)	69,671 (3.8%)	141,950 (11.6%)	82,315 (28.8%)
Unemployed	29,857 (0.9%)	14,401 (0.8%)	12,042 (1.0%)	3,414 (1.2%)
Missing	7,597 (0.2%)	1,324 (0.1%)	2,673 (0.2%)	3,600 (1.2%)

**Income Quantile**				
0-20%	931,240 (27.9%)	570,846 (31.2%)	288,013 (23.6%)	72,381 (25.0%)
20-40%	620,683 (18.6%)	321,388 (17.5%)	235,373 (19.3%)	63,922 (22.1%)
40-60%	440,545 (13.2%)	193,088 (10.5%)	180,985 (14.8%)	66,472 (23.0%)
60-80%	624,880 (18.7%)	343,012 (18.7%)	234,080 (19.2%)	47,788 (16.5%)
80-100%	726,310 (21.7%)	403,989 (22.0%)	283,744 (23.2%)	38,577 (13.3%)
Missing	48 (0.0%)	5 (0.0%)	7 (0.0%)	36 (0.0%)

Abbreviations: SD, Standard Deviation.

Table description: The exposure groups, *SARS-CoV-2 positive SARS-CoV-2 test*, *SARS-CoV-2 negative SARS-CoV-2 test*, and *no SARS-CoV-2 test*, were identified by end of follow up.

[a] The group *SARS-CoV-2 positive SARS-CoV-2 test* consisted of everyone with at least one positive test by the end of follow up.
[b] The group *SARS-CoV-2 negative SARS-CoV-2 test* consisted of everyone with at least one negative and no positive test by the end of follow up.
[c] The group *No SARS-CoV-2 test* consisted of everyone without any SARS-CoV-2 test result by the end of follow up.

Supplementary Table 3: Study population assessing mental disorder outcomes by SARS-CoV-2 test status.

	Total	SARS-CoV-2 positive[a]	SARS-CoV-2 negative[b]	No SARS-CoV-2 test[c]
**Total at end of follow-up (% by row)**	5,306,132 (100.0%)	2,837,134 (53.5%)	2,059,213 (38.8%)	409,785 (7.7%)
**Mental disorders at end of follow up (% by column)**				
Yes	132,979 (2.5%)	30,292 (1.1%)	70,638 (3.4%)	32,049 (7.8%)
No	5,173,153 (97.5%)	2,806,842 (98.9%)	1,988,575 (96.6%)	377,736 (92.2%)
**Time of Inclusion in Exposure Group (% by column)**				
2020, March – 2020, June	723,924 (13.6%)	11,355 (0.4%)	302,784 (14.7%)	409,785 (100.0%)
2020, July – 2020, December	1,239,407 (23.4%)	142,331 (5.0%)	1,097,076 (53.3%)	0 (0.0%)
2021, January – 2021, June	512,063 (9.7%)	112,719 (4.0%)	399,344 (19.4%)	0 (0.0%)
2021, July – 2021, December	642,029 (12.1%)	470,486 (16.6%)	171,543 (8.3%)	0 (0.0%)
2022, January – 2022, June	2,033,096 (38.3%)	1,962,771 (69.2%)	70,325 (3.4%)	0 (0.0%)
2022, July – 2022, December	139,888 (2.6%)	126,657 (4.5%)	13,231 (0.6%)	0 (0.0%)
2023, January – 2023, June	15,725 (0.3%)	10,815 (0.4%)	4,910 (0.2%)	0 (0.0%)
		**No. individuals (% by column) by the start of follow up**		
**Mean Age (SD)**	41.101 (23.891)	35.591 (21.657)	46.221 (24.467)	53.516 (25.286)
**Age group**				
<18	1,137,208 (21.4%)	763,566 (26.9%)	330,237 (16.0%)	43,405 (10.6%)
18-29	840,348 (15.8%)	490,452 (17.3%)	299,487 (14.5%)	50,409 (12.3%)
30-39	606,391 (11.4%)	386,594 (13.6%)	188,047 (9.1%)	31,750 (7.7%)
40-49	672,695 (12.7%)	407,415 (14.4%)	232,615 (11.3%)	32,665 (8.0%)
50-59	711,505 (13.4%)	360,657 (12.7%)	303,035 (14.7%)	47,813 (11.7%)
60-69	585,567 (11.0%)	229,075 (8.1%)	290,826 (14.1%)	65,666 (16.0%)
70-79	506,474 (9.5%)	146,121 (5.2%)	275,920 (13.4%)	84,433 (20.6%)
≥80	245,944 (4.6%)	53,254 (1.9%)	139,046 (6.8%)	53,644 (13.1%)
**Sex**				
Female	2,645,298 (49.9%)	1,453,427 (51.2%)	1,001,867 (48.7%)	190,004 (46.4%)
Male	2,660,834 (50.1%)	1,383,707 (48.8%)	1,057,346 (51.3%)	219,781 (53.6%)
**Mean Duration in years (SD)**	3.205 (0.516)	3.307 (0.170)	3.153 (0.583)	2.761 (1.121)
**Charlson Comorbidity Index**				
0	4,144,239 (78.1%)	2,335,062 (82.3%)	1,514,689 (73.6%)	294,488 (71.9%)
1	536,807 (10.1%)	268,737 (9.5%)	224,300 (10.9%)	43,770 (10.7%)
2	339,388 (6.4%)	137,836 (4.9%)	164,350 (8.0%)	37,202 (9.1%)
3+	285,698 (5.4%)	95,499 (3.4%)	155,874 (7.6%)	34,325 (8.4%)
**Parental Mental disorder**				
Yes	842,044 (15.9%)	482,906 (17.0%)	309,102 (15.0%)	50,036 (12.2%)
No	4,464,088 (84.1%)	2,354,228 (83.0%)	1,750,111 (85.0%)	359,749 (87.8%)
**Parental Charlson Comorbidity Index**				
0	2,888,118 (54.4%)	1,471,493 (51.9%)	1,144,839 (55.6%)	271,786 (66.3%)
1	608,918 (11.5%)	368,080 (13.0%)	211,067 (10.2%)	29,771 (7.3%)
2	617,996 (11.6%)	361,290 (12.7%)	224,969 (10.9%)	31,737 (7.7%)
3+	1,191,100 (22.4%)	636,271 (22.4%)	478,338 (23.2%)	76,491 (18.7%)
**Education**				
No education	899,644 (17.0%)	571,712 (20.2%)	277,706 (13.5%)	50,226 (12.3%)
Primary school	1,247,718 (23.5%)	602,261 (21.2%)	513,850 (25.0%)	131,607 (32.1%)
Vocational training or gymnasium	1,729,288 (32.6%)	873,346 (30.8%)	715,484 (34.7%)	140,458 (34.3%)
Higher education, short cycle	866,383 (16.3%)	483,216 (17.0%)	329,745 (16.0%)	53,422 (13.0%)
Higher education, long cycle	563,099 (10.6%)	306,599 (10.8%)	222,428 (10.8%)	34,072 (8.3%)
**Employment status**				
Kids and Education	1,500,689 (28.3%)	983,200 (34.7%)	460,676 (22.4%)	56,813 (14.0%)
Employed	2,516,821 (47.5%)	1,447,909 (51.1%)	930,968 (45.3%)	137,944 (34.0%)
Not in workforce	162,535 (3.1%)	73,271 (2.6%)	69,377 (3.4%)	19,887 (4.9%)
Retired	1,072,732 (20.2%)	308,341 (10.9%)	576,968 (28.1%)	187,423 (46.1%)
Unemployed	45,739 (0.9%)	23,038 (0.8%)	18,523 (0.9%)	4,178 (1.0%)
Missing	7,616 (0.1%)	1,375 (0.0%)	2,701 (0.1%)	3,540 (0.9%)
**Income Quantile**				

0-20%	1,084,667 (20.4%)	687,154 (24.2%)	326,787 (15.9%)	70,726 (17.3%)
20-40%	999,965 (18.8%)	490,152 (17.3%)	410,529 (19.9%)	99,284 (24.2%)
40-60%	995,090 (18.8%)	403,561 (14.2%)	462,102 (22.4%)	129,427 (31.6%)
60-80%	1,090,290 (20.5%)	600,726 (21.2%)	422,013 (20.5%)	67,551 (16.5%)
80-100%	1,136,071 (21.4%)	655,536 (23.1%)	437,775 (21.3%)	42,760 (10.4%)
Missing	49 (0.0%)	5 (0.0%)	7 (0.0%)	37 (0.0%)

Abbreviations: SD, Standard Deviation.

Table description: The exposure groups, *SARS-CoV-2 positive SARS-CoV-2 test*, *SARS-CoV-2 negative SARS-CoV-2 test*, and *no SARS-CoV-2 test*, were identified by end of follow up.

[a] The group *SARS-CoV-2 positive SARS-CoV-2 test* consisted of everyone with at least one positive test by the end of follow up.

[b] The group *SARS-CoV-2 negative SARS-CoV-2 test* consisted of everyone with at least one negative and no positive test by the end of follow up.

[c] The group *No SARS-CoV-2 test* consisted of everyone without any SARS-CoV-2 test result by the end of follow up.

Supplementary Table 4: Study population assessing general medical condition outcomes by SARS-CoV-2 test status.

	Total	SARS-CoV-2 positive[a]	SARS-CoV-2 negative[b]	No SARS-CoV-2 test[c]
**Total at end of follow-up (% by row)**	3,517,630 (100.0%)	1,931,527 (54.9%)	1,289,639 (36.7%)	296,464 (8.4%)
**General medical condition at end of follow up (% by column)**				
Yes	320,832 (9.1%)	74,638 (3.9%)	168,329 (13.1%)	77,865 (26.3%)
No	3,196,798 (90.9%)	1,856,889 (96.1%)	1,121,310 (86.9%)	218,599 (73.7%)
**Time of Inclusion in Exposure Group (% by column)**				
2020, March – 2020, June	473,523 (13.5%)	6,381 (0.3%)	170,678 (13.2%)	296,464 (100.0%)
2020, July – 2020, December	821,320 (23.3%)	98,359 (5.1%)	722,961 (56.1%)	0 (0.0%)
2021, January – 2021, June	328,345 (9.3%)	79,646 (4.1%)	248,699 (19.3%)	0 (0.0%)
2021, July – 2021, December	446,726 (12.7%)	341,245 (17.7%)	105,481 (8.2%)	0 (0.0%)
2022, January – 2022, June	1,377,370 (39.2%)	1,340,363 (69.4%)	37,007 (2.9%)	0 (0.0%)
2022, July – 2022, December	65,928 (1.9%)	62,290 (3.2%)	3,638 (0.3%)	0 (0.0%)
2023, January – 2023, June	4,418 (0.1%)	3,243 (0.2%)	1,175 (0.1%)	0 (0.0%)
		**No. individuals (% by column) by the start of follow up**		
**Mean Age (SD)**	33.784 (21.087)	29.852 (18.807)	36.949 (21.858)	45.630 (24.673)
**Age group**				
<18	977,293 (27.8%)	639,886 (33.1%)	291,781 (22.6%)	45,626 (15.4%)
18-29	697,254 (19.8%)	396,539 (20.5%)	255,444 (19.8%)	45,271 (15.3%)
30-39	486,021 (13.8%)	296,275 (15.3%)	159,092 (12.3%)	30,654 (10.3%)
40-49	475,373 (13.5%)	272,253 (14.1%)	172,518 (13.4%)	30,602 (10.3%)
50-59	424,264 (12.1%)	196,694 (10.2%)	187,051 (14.5%)	40,519 (13.7%)
60-69	267,585 (7.6%)	91,047 (4.7%)	131,536 (10.2%)	45,002 (15.2%)
70-79	153,443 (4.4%)	34,198 (1.8%)	76,157 (5.9%)	43,088 (14.5%)
≥80	36,397 (1.0%)	4,635 (0.2%)	16,060 (1.2%)	15,702 (5.3%)
**Sex**				
Female	1,729,059 (49.2%)	990,993 (51.3%)	609,897 (47.3%)	128,169 (43.2%)
Male	1,788,571 (50.8%)	940,534 (48.7%)	679,742 (52.7%)	168,295 (56.8%)
**Mean Duration in years (SD)**	3.121 (0.654)	3.291 (0.210)	3.043 (0.717)	2.347 (1.321)
**Charlson Comorbidity Index**				
0	3,340,690 (95.0%)	1,857,997 (96.2%)	1,210,376 (93.9%)	272,317 (91.9%)
1	52,366 (1.5%)	24,770 (1.3%)	21,492 (1.7%)	6,104 (2.1%)
2	104,020 (3.0%)	41,684 (2.2%)	47,723 (3.7%)	14,613 (4.9%)
3+	20,554 (0.6%)	7,076 (0.4%)	10,048 (0.8%)	3,430 (1.2%)
**Parental Mental disorder**				
Yes	593,835 (16.9%)	333,695 (17.3%)	218,186 (16.9%)	41,954 (14.2%)
No	2,923,795 (83.1%)	1,597,832 (82.7%)	1,071,453 (83.1%)	254,510 (85.8%)
**Parental Charlson Comorbidity Index**				
0	1,914,172 (54.4%)	1,043,244 (54.0%)	686,245 (53.2%)	184,683 (62.3%)
1	441,081 (12.5%)	262,932 (13.6%)	153,023 (11.9%)	25,126 (8.5%)
2	433,453 (12.3%)	249,361 (12.9%)	157,694 (12.2%)	26,398 (8.9%)
3+	728,924 (20.7%)	375,990 (19.5%)	292,677 (22.7%)	60,257 (20.3%)
**Education**				
No education	758,019 (21.5%)	473,113 (24.5%)	235,291 (18.2%)	49,615 (16.7%)
Primary school	750,953 (21.3%)	394,589 (20.4%)	277,856 (21.5%)	78,508 (26.5%)
Vocational training or gymnasium	1,041,877 (29.6%)	533,675 (27.6%)	410,447 (31.8%)	97,755 (33.0%)
Higher education, short cycle	549,978 (15.6%)	307,093 (15.9%)	202,102 (15.7%)	40,783 (13.8%)
Higher education, long cycle	416,803 (11.8%)	223,057 (11.5%)	163,943 (12.7%)	29,803 (10.1%)
**Employment status**				
Kids and Education	1,277,841 (36.4%)	818,533 (42.4%)	402,357 (31.3%)	56,951 (19.4%)
Employed	1,776,021 (50.6%)	978,184 (50.7%)	670,207 (52.1%)	127,630 (43.6%)
Not in workforce	100,835 (2.9%)	42,093 (2.2%)	43,960 (3.4%)	14,782 (5.0%)
Retired	322,689 (9.2%)	75,486 (3.9%)	157,179 (12.2%)	90,024 (30.7%)
Unemployed	32,644 (0.9%)	15,898 (0.8%)	13,252 (1.0%)	3,494 (1.2%)
Missing	7,600 (0.2%)	1,333 (0.1%)	2,684 (0.2%)	3,583 (1.2%)

**Income Quantile**				
0-20%	940,662 (26.7%)	579,675 (30.0%)	289,872 (22.5%)	71,115 (24.0%)
20-40%	662,039 (18.8%)	345,235 (17.9%)	252,139 (19.6%)	64,665 (21.8%)
40-60%	489,032 (13.9%)	213,942 (11.1%)	202,678 (15.7%)	72,412 (24.4%)
60-80%	667,756 (19.0%)	369,085 (19.1%)	249,591 (19.4%)	49,080 (16.6%)
80-100%	758,093 (21.6%)	423,585 (21.9%)	295,352 (22.9%)	39,156 (13.2%)
Missing	48 (0.0%)	5 (0.0%)	7 (0.0%)	36 (0.0%)

Abbreviations: SD, Standard Deviation.

Table description: The exposure groups, *SARS-CoV-2 positive SARS-CoV-2 test*, *SARS-CoV-2 negative SARS-CoV-2 test*, and *no SARS-CoV-2 test*, were identified by end of follow up.

[a] The group *SARS-CoV-2 positive SARS-CoV-2 test* consisted of everyone with at least one positive test by the end of follow up.
[b] The group *SARS-CoV-2 negative SARS-CoV-2 test* consisted of everyone with at least one negative and no positive test by the end of follow up.
[c] The group *No SARS-CoV-2 test* consisted of everyone without any SARS-CoV-2 test result by the end of follow up.

Supplementary Figure 2: Time-varying visualization of the study population assessing any mental disorder or general medical condition outcomes.

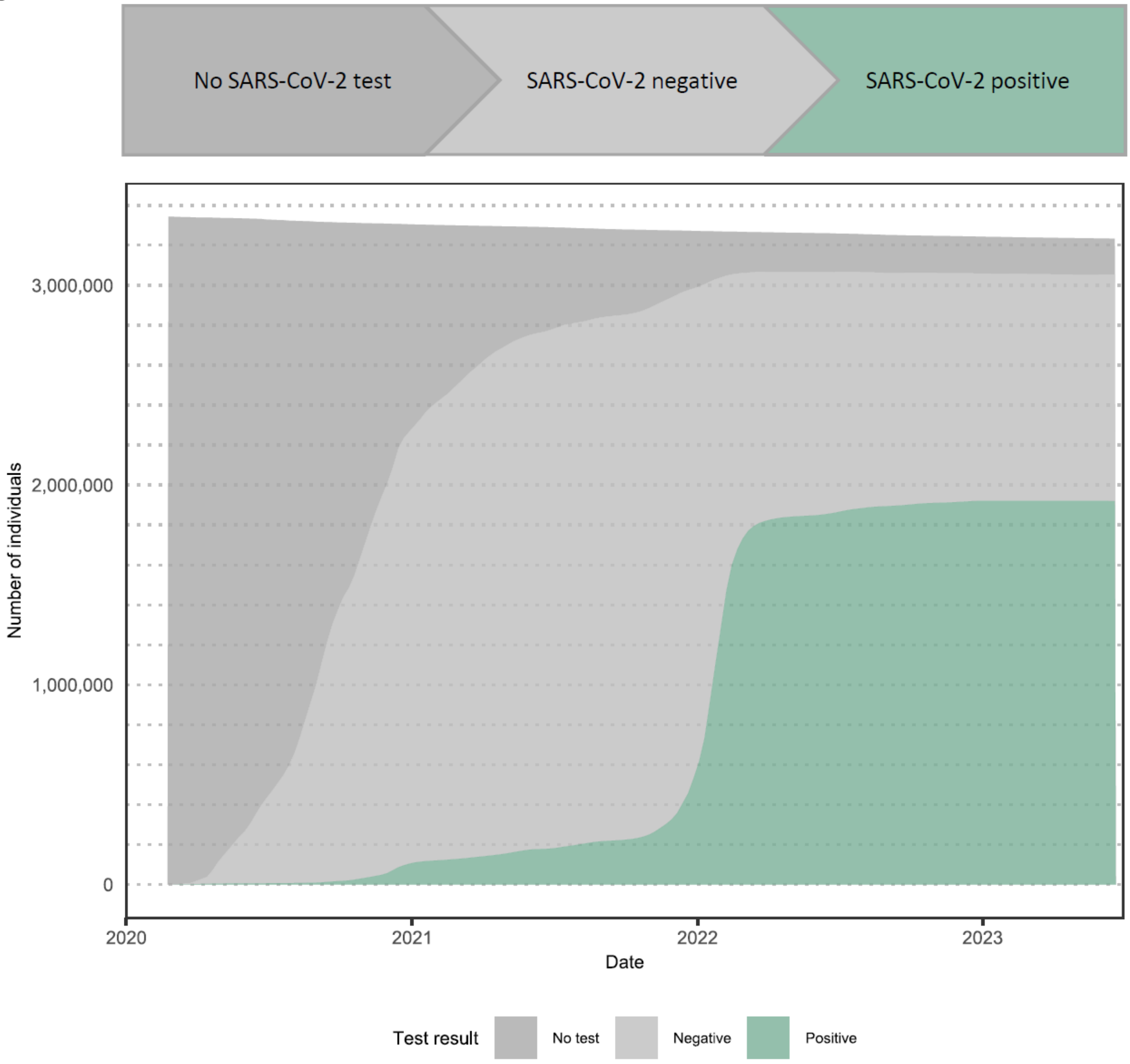

The exposure was hierarchal and time-varying, meaning individuals could change exposure groups over time, but only to the right in the pictogram, i.e., from not tested, to tested negative, to tested positive. This means that the composition of the exposure group changes over time, but the same individual was never in two groups simultaneously. The study population decreases in size over time due to events or censoring.

Supplementary Table 5: Study population assessing any outcome by COVID-19 admission status without and with intensive care unit (ICU) admission.

	No COVID-19 hospitalization[a]	COVID-19-hospitalization without ICU[b]	COVID-19-hospitalization with ICU[c]
**Total at end of follow-up (% by row)**	3,045,121 (99.69%)	8,993 (0.29%)	416 (0.01%)
**Mental disorders or general medical conditions at end of follow up (% by column)**			
Yes	264,846 (8.7%)	1,345 (15.0%)	146 (35.1%)
No	2,780,275 (91.3%)	7,648 (85.0%)	270 (64.9%)
**Time of Inclusion in Exposure Group**			
2020, March – 2020, June	416,885 (13.7%)	487 (5.4%)	43 (10.3%)
2020, July – 2020, December	1,807,864 (59.4%)	851 (9.5%)	68 (16.3%)
2021, January – 2021, June	512,385 (16.8%)	1,145 (12.7%)	92 (22.1%)
2021, July – 2021, December	213,266 (7.0%)	1,317 (14.6%)	94 (22.6%)
2022, January – 2022, June	88,595 (2.9%)	4,175 (46.4%)	96 (23.1%)
2022, July – 2022, December	4,948 (0.2%)	743 (8.3%)	19 (4.6%)
2023, January – 2023, June	1,178 (0.0%)	275 (3.1%)	4 (1.0%)
	**No. individuals (% by column) by the start of follow up**		
**Mean Age (SD)**	33.1 (20.6)	40.5 (20.4)	48.9 (22.4)
**Age group**			
<18	888,566 (29.2%)	817 (9.1%)	50 (12.0%)
18-39	1,024,314 (33.6%)	4,566 (50.8%)	80 (19.2%)
40-59	777,150 (25.5%)	1,898 (21.1%)	133 (32.0%)
60-79	331,188 (10.9%)	1,279 (14.2%)	138 (33.2%)
≥80	23,903 (0.8%)	433 (4.8%)	15 (3.6%)
**Sex**			
Female	1,503,349 (49.4%)	5,871 (65.3%)	151 (36.3%)
Male	1,541,772 (50.6%)	3,122 (34.7%)	265 (63.7%)
**Mean Duration in years (SD)**	2.4 (0.6)	2.4 (0.8)	1.7 (1.1)
**Charlson Comorbidity Index**			
0	2,896,745 (95.1%)	8,084 (89.9%)	347 (83.4%)
1	44,111 (1.4%)	219 (2.4%)	16 (3.8%)
2	87,169 (2.9%)	517 (5.7%)	40 (9.6%)
3+	17,096 (0.6%)	173 (1.9%)	13 (3.1%)
**Parental Mental disorder**			
Yes	507,338 (16.7%)	1,469 (16.3%)	49 (11.8%)
No	2,537,783 (83.3%)	7,524 (83.7%)	367 (88.2%)
**Parental Charlson Comorbidity Index**			
0	1,629,825 (53.5%)	4,983 (55.4%)	245 (58.9%)
1	392,785 (12.9%)	1,042 (11.6%)	38 (9.1%)
2	384,447 (12.6%)	1,162 (12.9%)	37 (8.9%)

3+	638,064 (21.0%)	1,806 (20.1%)	96 (23.1%)
**Education**			
No education	647,343 (21.3%)	731 (8.1%)	57 (13.7%)
Primary school	632,724 (20.8%)	1,747 (19.4%)	114 (27.4%)
Vocational training or gymnasium	897,358 (29.5%)	2,860 (31.8%)	148 (35.6%)
Higher education, short cycle	486,756 (16.0%)	1,969 (21.9%)	61 (14.7%)
Higher education, long cycle	380,940 (12.5%)	1,686 (18.7%)	36 (8.7%)
**Employment status**			
Kids and Education	1,177,987 (38.7%)	1,645 (18.3%)	68 (16.3%)
Employed	1,547,146 (50.8%)	5,427 (60.3%)	201 (48.3%)
Not in workforce	62,546 (2.1%)	357 (4.0%)	23 (5.5%)
Retired	219,007 (7.2%)	1,393 (15.5%)	113 (27.2%)
Unemployed	35,998 (1.2%)	163 (1.8%)	11 (2.6%)
Missing	2,437 (0.1%)	8 (0.1%)	0 (0.0%)
**Income Quantile**			
0-20%	856,239 (28.1%)	901 (10.0%)	53 (12.7%)
20-40%	547,705 (18.0%)	1,849 (20.6%)	93 (22.4%)
40-60%	377,160 (12.4%)	1,952 (21.7%)	99 (23.8%)
60-80%	578,684 (19.0%)	2,283 (25.4%)	84 (20.2%)
80-100%	685,321 (22.5%)	2,008 (22.3%)	87 (20.9%)
Missing	12 (0.0%)	0 (0.0%)	0 (0.0%)

Abbreviations: SD, Standard Deviation; ICU, Intensive Care Unit.

Table description: The exposure groups, *no COVID-19 hospitalization*, *COVID-19 hospitalization without ICU*, and *COVID-19 hospitalization with ICU*, were identified by end of follow up.

[a] The group *no COVID-19 hospitalization* consisted of individuals without admission to a hospital with SARS-CoV-2 infection, i.e., all individuals without SARS-CoV-2 test result, with only negative test results, or a positive test result but no hospital admission by end of follow up.
[b] The group *COVID-19 hospitalization without ICU* consisted of everyone with at least one admission to a hospital with COVID-19 and without ICU admission by the end of follow up.
[c] The group *COVID-19 hospitalization with ICU* consisted of everyone with at least one admission to the hospital with COVID-19 and with ICU admission by the end of follow up.

Results: Positive compared with negative and with no SARS-CoV-2 test

Supplementary Table 6: Positive, negative, and no SARS-CoV-2 tests compared with each other.
Hazard ratios of specific first mental health or general medical disorders for negative and positive SARS-CoV-2 tests compared to individuals not tested, and for positive SARS-CoV-2 tests compared to negative SARS-CoV-2 tests.

Outcome	SARS-CoV-2 test	Cases, No.	HR (95% CI)[a]	p-value
Mental disorders or general medical conditions	No test	87,180	1.00 [reference]	..
	Negative test	183,105	1.73 (1.71 – 1.75)	<0.001
	Positive test	83,232	1.71 (1.68 – 1.73)	<0.001
	Positive vs. negative test	83,232	0.99 (0.98 – 1.00)	0.007
Mental disorders	No test	32,049	1.00 [reference]	..
	Negative test	70,638	1.37 (1.35 – 1.40)	<0.001
	Positive test	30,292	1.12 (1.10 – 1.15)	<0.001
	Positive vs. negative test	30,292	0.82 (0.80 – 0.83)	<0.001
General medical conditions	No test	77,865	1.00 [reference]	..
	Negative test	168,329	1.88 (1.86 – 1.90)	<0.001
	Positive test	74,638	1.90 (1.87 – 1.93)	<0.001
	Positive vs. negative test	74,638	1.01 (1.00 – 1.02)	0.024
Neurological disorders	No test	64,317	1.00 [reference]	..
	Negative test	130,746	1.55 (1.53 – 1.57)	<0.001
	Positive test	52,946	1.54 (1.51 – 1.56)	<0.001
	Positive vs. negative test	52,946	0.99 (0.98 – 1.01)	0.266
Respiratory disorders	No test	12,895	1.00 [reference]	..
	Negative test	46,302	4.44 (4.33 – 4.56)	<0.001
	Positive test	14,805	4.34 (4.20 – 4.49)	<0.001
	Positive vs. negative test	14,805	0.98 (0.96 – 1.00)	0.048
Circulatory disorders	No test	42,430	1.00 [reference]	..
	Negative test	91,490	1.95 (1.92 – 1.98)	<0.001
	Positive test	29,328	1.86 (1.83 – 1.90)	<0.001
	Positive vs. negative test	29,328	0.96 (0.94 – 0.97)	<0.001
Kidney disorders	No test	6,947	1.00 [reference]	..
	Negative test	19,123	2.42 (2.34 – 2.50)	<0.001
	Positive test	4,371	2.02 (1.93 – 2.11)	<0.001
	Positive vs. negative test	4,371	0.83 (0.80 – 0.86)	<0.001
Gastrointestinal disorders	No test	12,428	1.00 [reference]	..
	Negative test	46,488	3.88 (3.78 – 3.98)	<0.001
	Positive test	14,619	3.56 (3.45 – 3.68)	<0.001
	Positive vs. negative test	14,619	0.92 (0.90 – 0.94)	<0.001
Endocrine disorders	No test	9,101	1.00 [reference]	..
	Negative test	21,174	1.95 (1.89 – 2.01)	<0.001
	Positive test	6,400	1.80 (1.72 – 1.87)	<0.001
	Positive vs. negative test	6,400	0.92 (0.89 – 0.95)	<0.001
Hematological disorders	No test	9,456	1.00 [reference]	..
	Negative test	28,632	2.95 (2.87 – 3.04)	<0.001
	Positive test	7,744	2.64 (2.54 – 2.74)	<0.001
	Positive vs. negative test	7,744	0.89 (0.87 – 0.92)	<0.001
Musculoskeletal disorders	No test	8,131	1.00 [reference]	..
	Negative test	23,118	1.74 (1.68 – 1.79)	<0.001
	Positive test	10,922	1.86 (1.79 – 1.94)	<0.001
	Positive vs. negative test	10,922	1.07 (1.05 – 1.10)	<0.001
Dermatological disorders	No test	4,436	1.00 [reference]	..
	Negative test	12,229	2.13 (2.04 – 2.24)	<0.001
	Positive test	6,204	2.27 (2.14 – 2.40)	<0.001
	Positive vs. negative test	6,204	1.06 (1.02 – 1.10)	0.002
Neuropsychiatric symptoms	No test	19,634	1.00 [reference]	..
	Negative test	53,209	2.23 (2.19 – 2.28)	<0.001
	Positive test	18,416	2.42 (2.35 – 2.48)	<0.001
	Positive vs. negative test	18,416	1.08 (1.06 – 1.10)	<0.001

Abbreviations: HR, Hazard Ratio; CI, Confidence Interval.

[a] The estimates are HRs with 95% CI from Cox Proportional Hazards model stratified by age and adjusted for confounders (sex, Charlson Comorbidity Index (CCI), parental CCI, parental mental health, employment status, income, highest level of education). All statistical tests were two-sided without correction for multiple comparisons. All statistical tests were two-sided without correction for multiple comparisons.

Results: Positive compared with negative SARS-CoV-2 test
Supplementary Table 7: Age group.

Hazard ratios of specific first mental health or general medical disorders among individuals with positive SARS-CoV-2 test compared to negative SARS-CoV-2 tests by age group.

Outcome	Age group	Cases, No.		HR (95% CI)[a]	p-value
		SARS-CoV-2 negative	SARS-CoV-2 positive		
Mental disorders or general medical conditions	<18	30,062	20,084	0.96 (0.93 - 0.98)	<0.001
	18-29	28,818	14,920	0.99 (0.97 - 1.02)	0.485
	30-39	18,924	10,589	1.05 (1.02 - 1.08)	0.002
	40-49	22,312	11,811	1.05 (1.02 - 1.07)	0.002
	50-59	28,768	12,088	1.05 (1.03 - 1.08)	<0.001
	60-69	25,327	7,718	1.04 (1.01 - 1.07)	0.018
	70-79	21,921	4,938	0.98 (0.95 - 1.02)	0.347
	≥80	6,973	1,084	0.90 (0.84 - 0.96)	0.001
Mental disorders	<18	13,039	8,848	0.82 (0.80 - 0.85)	<0.001
	18-29	20,025	8,960	0.82 (0.79 - 0.84)	<0.001
	30-39	9,126	4,249	0.84 (0.80 - 0.88)	<0.001
	40-49	7,979	3,385	0.83 (0.79 - 0.87)	<0.001
	50-59	7,845	2,542	0.84 (0.80 - 0.88)	<0.001
	60-69	5,007	1,082	0.76 (0.70 - 0.81)	<0.001
	70-79	4,649	778	0.83 (0.76 - 0.90)	<0.001
	≥80	2,968	448	0.94 (0.84 - 1.04)	0.223
General medical conditions	<18	23,396	15,737	1.00 (0.98 - 1.03)	0.729
	18-29	20,100	11,116	1.07 (1.04 - 1.10)	<0.001
	30-39	16,861	9,863	1.12 (1.08 - 1.15)	<0.001
	40-49	21,741	11,619	1.08 (1.05 - 1.11)	<0.001
	50-59	29,271	12,124	1.06 (1.03 - 1.09)	<0.001
	60-69	26,673	7,970	1.04 (1.01 - 1.06)	0.016
	70-79	23,060	5,096	0.98 (0.94 - 1.01)	0.142
	≥80	7,227	1,113	0.88 (0.82 - 0.94)	<0.001
Neurological disorders	<18	13,139	9,939	1.00 (0.97 - 1.04)	0.932
	18-29	10,999	5,685	0.89 (0.86 - 0.93)	<0.001
	30-39	9,627	5,643	1.02 (0.98 - 1.07)	0.310
	40-49	13,343	7,109	1.02 (0.98 - 1.05)	0.379
	50-59	19,981	8,444	1.06 (1.03 - 1.09)	<0.001
	60-69	22,629	7,080	1.03 (1.00 - 1.06)	0.030
	70-79	28,732	6,833	0.99 (0.97 - 1.02)	0.661
	≥80	12,296	2,213	1.00 (0.96 - 1.05)	0.931
Respiratory disorders	<18	6,298	3,069	0.92 (0.87 - 0.98)	0.006
	18-29	2,279	1,285	1.31 (1.20 - 1.43)	<0.001
	30-39	2,155	1,199	1.30 (1.18 - 1.42)	<0.001
	40-49	3,397	1,619	1.20 (1.11 - 1.29)	<0.001
	50-59	6,211	2,165	1.02 (0.96 - 1.08)	0.530
	60-69	8,892	2,296	1.02 (0.97 - 1.07)	0.447
	70-79	10,820	2,094	0.93 (0.88 - 0.98)	0.006
	≥80	6,250	1,078	1.02 (0.95 - 1.09)	0.647
Circulatory disorders	<18	1,129	830	1.08 (0.96 - 1.22)	0.202
	18-29	3,237	1,774	1.08 (1.01 - 1.16)	0.025
	30-39	4,237	2,439	1.05 (0.99 - 1.12)	0.127
	40-49	9,032	4,564	0.99 (0.95 - 1.03)	0.570
	50-59	17,916	7,025	1.03 (1.00 - 1.06)	0.074
	60-69	21,682	6,107	1.02 (0.99 - 1.06)	0.174
	70-79	22,923	4,762	0.97 (0.94 - 1.00)	0.072
	≥80	11,334	1,827	0.90 (0.85 - 0.95)	<0.001
Kidney disorders	<18	247	152	1.09 (0.83 - 1.43)	0.558
	18-29	307	167	1.19 (0.94 - 1.50)	0.139
	30-39	360	157	0.83 (0.66 - 1.05)	0.123
	40-49	650	248	0.80 (0.68 - 0.95)	0.012
	50-59	1,539	530	0.88 (0.79 - 0.99)	0.027
	60-69	3,301	811	0.88 (0.81 - 0.96)	0.004
	70-79	6,420	1,247	0.88 (0.83 - 0.94)	<0.001

Outcome	Age group	Cases, No. SARS-CoV-2 negative	SARS-CoV-2 positive	HR (95% CI)[a]	p-value
	≥80	6,299	1,059	0.88 (0.82 - 0.94)	<0.001
Gastrointestinal disorders	<18	1,561	1,051	1.07 (0.96 - 1.19)	0.219
	18-29	4,565	2,229	1.06 (1.00 - 1.13)	0.053
	30-39	4,044	1,900	1.00 (0.93 - 1.07)	0.925
	40-49	5,470	2,332	0.94 (0.89 - 1.00)	0.047
	50-59	8,281	2,746	0.95 (0.90 - 1.00)	0.048
	60-69	9,122	2,082	0.91 (0.86 - 0.96)	<0.001
	70-79	8,930	1,640	0.95 (0.90 - 1.01)	0.111
	≥80	4,515	639	0.85 (0.78 - 0.93)	<0.001
Endocrine disorders	<18	715	469	0.93 (0.80 - 1.08)	0.334
	18-29	730	361	0.86 (0.74 - 1.00)	0.044
	30-39	1,004	523	0.94 (0.82 - 1.07)	0.326
	40-49	1,901	839	0.92 (0.83 - 1.01)	0.083
	50-59	3,958	1,423	1.00 (0.94 - 1.07)	0.945
	60-69	4,928	1,295	1.01 (0.95 - 1.08)	0.725
	70-79	5,313	1,043	0.98 (0.92 - 1.06)	0.679
	≥80	2,625	447	0.99 (0.89 - 1.11)	0.900
Hematological disorders	<18	571	421	0.99 (0.84 - 1.17)	0.897
	18-29	1,044	683	1.24 (1.10 - 1.40)	<0.001
	30-39	1,234	769	1.19 (1.06 - 1.34)	0.003
	40-49	1,994	996	1.09 (0.99 - 1.19)	0.087
	50-59	2,626	826	0.83 (0.76 - 0.91)	<0.001
	60-69	4,538	1,073	0.88 (0.82 - 0.95)	0.001
	70-79	8,603	1,622	0.88 (0.83 - 0.93)	<0.001
	≥80	8,022	1,354	0.95 (0.89 - 1.01)	0.078
Musculoskeletal disorders	<18	1,583	1,274	1.05 (0.96 - 1.16)	0.297
	18-29	2,277	1,418	1.12 (1.03 - 1.21)	0.008
	30-39	2,551	1,651	1.10 (1.02 - 1.20)	0.015
	40-49	4,209	2,274	0.98 (0.92 - 1.05)	0.585
	50-59	5,274	2,320	1.08 (1.02 - 1.14)	0.010
	60-69	3,607	1,205	1.16 (1.07 - 1.25)	<0.001
	70-79	2,705	613	1.03 (0.94 - 1.13)	0.550
	≥80	912	167	0.91 (0.76 - 1.08)	0.290
Dermatological disorders	<18	2,962	1,880	0.95 (0.88 - 1.03)	0.219
	18-29	2,123	1,315	1.16 (1.07 - 1.27)	0.001
	30-39	1,459	891	1.05 (0.95 - 1.17)	0.341
	40-49	1,348	779	1.23 (1.10 - 1.37)	<0.001
	50-59	1,459	636	1.23 (1.10 - 1.37)	<0.001
	60-69	1,232	365	1.00 (0.88 - 1.14)	0.993
	70-79	1,114	250	1.03 (0.89 - 1.20)	0.652
	≥80	532	88	0.94 (0.74 - 1.19)	0.590
Neuropsychiatric symptoms	<18	1,869	1,395	1.26 (1.15 - 1.39)	<0.001
	18-29	4,077	2,476	1.37 (1.29 - 1.46)	<0.001
	30-39	3,488	2,110	1.37 (1.27 - 1.47)	<0.001
	40-49	4,559	2,562	1.31 (1.23 - 1.39)	<0.001
	50-59	7,233	2,890	1.14 (1.09 - 1.20)	<0.001
	60-69	9,052	2,528	1.05 (1.00 - 1.10)	0.059
	70-79	13,464	2,793	0.97 (0.93 - 1.01)	0.168
	≥80	9,467	1,662	0.93 (0.88 - 0.98)	0.009

Abbreviations: HR, Hazard Ratio; CI, Confidence Interval.

[a] The estimates are HRs with 95% CI from Cox Proportional Hazards model stratified by age and adjusted for confounders (sex, Charlson Comorbidity Index (CCI), parental CCI, parental mental health, employment status, income, highest level of education). All statistical tests were two-sided without correction for multiple comparisons.

Supplementary Figure 3: Hazard ratios of first specific general medical conditions by age group and severity of COVID-19.

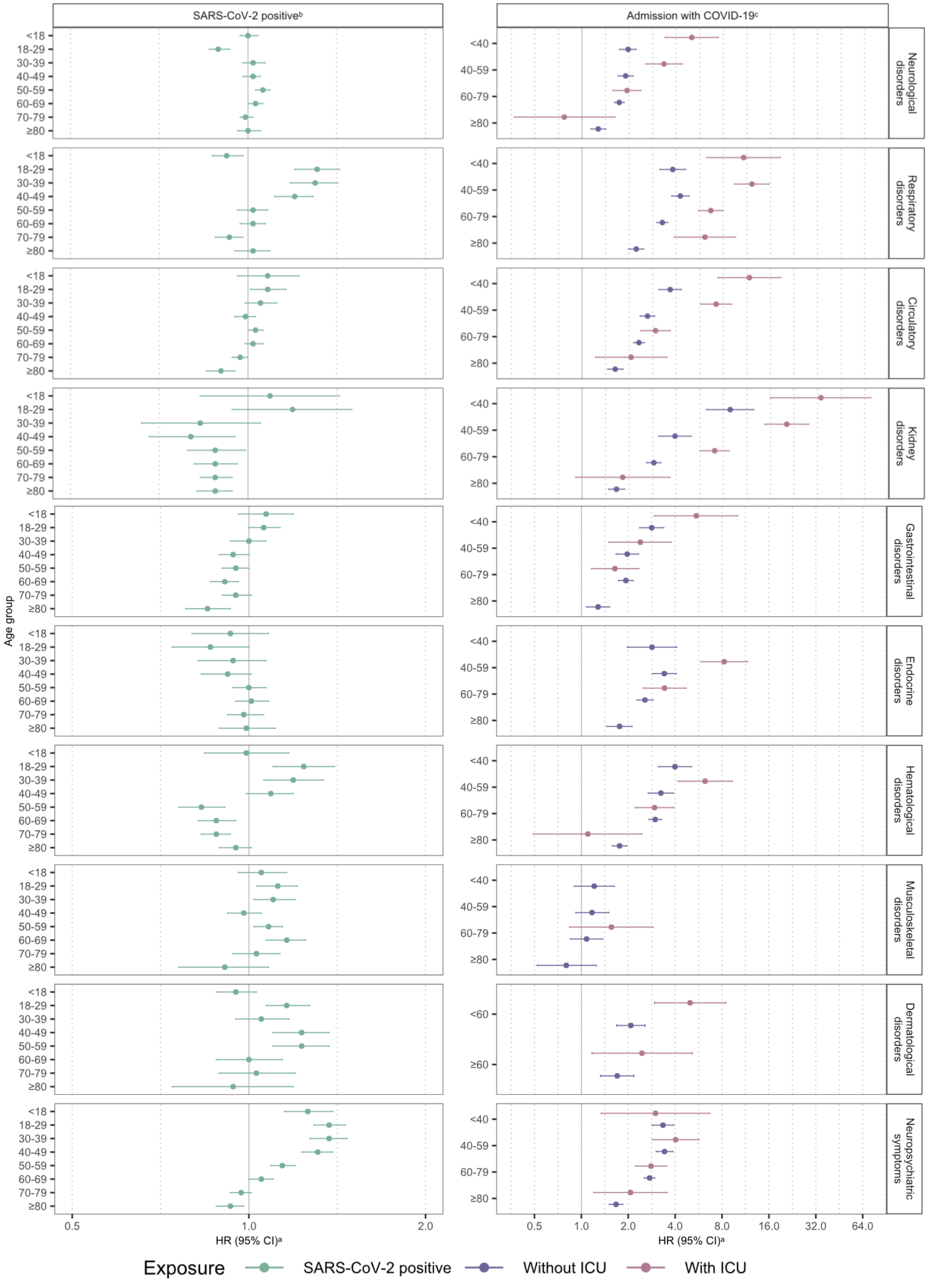

The specific general medical conditions are neurological (n = 4,323,502), respiratory (n = 4,915,770), circulatory (n = 4,531,873), kidney (n = 5,315,598), gastrointestinal (n = 4,943,199), endocrine (n = 5,193,087), hematological (n = 5,249,230), musculoskeletal (n = 5,230,816), dermatological disorders (n = 5,272,108), and neuropsychiatric symptoms (n = 5,125,359). The estimates presented in the figure are available in Supplementary Table 7 and 13. Source data are provided as a Source Data file.

Abbreviations: HR, Hazard Rate Ratio; CI, Confidence Interval; ICU, Intensive Care Unit.
[a] Based on Cox Proportional Hazards model stratified by age and adjusted for confounders (sex, Charlson Comorbidity Index (CCI), parental CCI, parental psychiatric history, employment status, income, and highest level of education).
[b] The exposure was individuals with positive SARS-CoV-2 tests and the reference was individuals with negative SARS-CoV-2 tests.
[c] The exposure was individuals admitted with COVID-19 and the reference group was individuals not admitted to the hospital with COVID-19, i.e., all individuals with negative or positive test results but no admission to hospital with COVID-19. The definition of admission to the hospital and ICU is summarized in Supplementary Table 30.

Supplementary Table 8: Number of positive tests.

Hazard ratios of specific first mental health or general medical disorders among individuals with one positive SARS-CoV-2 test and two or more positive SARS-CoV-2 tests compared to negative SARS-CoV-2 tests.

Outcome	Number of tests	Cases, No.	HR (95% CI)[a]	p-value
Mental disorders or general medical conditions	Negative test	183,105	1.00 [reference]	..
	1 positive test	78,276	1.01 (0.99 – 1.02)	0.338
	2+ positive tests	4,956	1.12 (1.09 – 1.15)	<0.001
Mental disorders	Negative test	82,280	1.00 [reference]	..
	1 positive test	17,440	0.75 (0.74 – 0.77)	<0.001
	2+ positive tests	1,210	0.72 (0.68 – 0.77)	<0.001
General medical conditions	Negative test	173,482	1.00 [reference]	..
	1 positive test	65,405	0.99 (0.98 – 1.01)	0.297
	2+ positive tests	4,080	1.15 (1.11 – 1.18)	<0.001
Neurological disorders	Negative test	153,469	1.00 [reference]	..
	1 positive test	28,589	0.85 (0.84 – 0.87)	<0.001
	2+ positive tests	1,634	0.88 (0.84 – 0.93)	<0.001
Respiratory disorders	Negative test	54,343	1.00 [reference]	..
	1 positive test	6,342	0.82 (0.80 – 0.85)	<0.001
	2+ positive tests	422	1.02 (0.92 – 1.12)	0.698
Circulatory disorders	Negative test	105,961	1.00 [reference]	..
	1 positive test	14,178	0.90 (0.88 – 0.92)	<0.001
	2+ positive tests	679	0.97 (0.90 – 1.05)	0.411
Kidney disorders	Negative test	22,636	1.00 [reference]	..
	1 positive test	815	0.67 (0.62 – 0.73)	<0.001
	2+ positive tests	43	0.83 (0.62 – 1.13)	0.234
Gastrointestinal disorders	Negative test	54,602	1.00 [reference]	..
	1 positive test	6,047	0.78 (0.75 – 0.80)	<0.001
	2+ positive tests	458	1.02 (0.93 – 1.12)	0.739
Endocrine disorders	Negative test	25,301	1.00 [reference]	..
	1 positive test	2,163	0.76 (0.72 – 0.80)	<0.001
	2+ positive tests	110	0.77 (0.64 – 0.93)	0.007
Hematological disorders	Negative test	34,011	1.00 [reference]	..
	1 positive test	2,164	0.78 (0.74 – 0.82)	<0.001
	2+ positive tests	201	1.36 (1.18 – 1.56)	<0.001
Musculoskeletal disorders	Negative test	28,836	1.00 [reference]	..
	1 positive test	4,866	0.85 (0.82 – 0.87)	<0.001
	2+ positive tests	338	0.95 (0.85 – 1.06)	0.387
Dermatological disorders	Negative test	14,800	1.00 [reference]	..
	1 positive test	3,371	0.90 (0.87 – 0.94)	<0.001
	2+ positive tests	262	1.09 (0.96 – 1.23)	0.185
Neuropsychiatric symptoms	Negative test	64,698	1.00 [reference]	..
	1 positive test	6,370	0.84 (0.81 – 0.86)	<0.001
	2+ positive tests	557	1.30 (1.20 – 1.42)	<0.001

Abbreviations: HR, Hazard Ratio; CI, Confidence Interval.

[a] The estimates are HRs with 95% CI from Cox Proportional Hazards model stratified by age and adjusted for confounders (sex, Charlson Comorbidity Index (CCI), parental CCI, parental mental health, employment status, income, highest level of education). All statistical tests were two-sided without correction for multiple comparisons.

Supplementary Table 9: Time since test.

Hazard ratios of specific first mental health or general medical disorders at different time points since positive SARS-CoV-2 test compared to negative SARS-CoV-2 test.

Outcome	Time since positive test	Cases, No.	HR (95% CI)[a]	p-value
	SARS-CoV-2 negative	183,105	1.00 [reference]	..
	<1 month	4,972	1.05 (1.02 – 1.08)	0.003
	1-2 months	9,386	0.98 (0.96 – 1.01)	0.139
Mental disorders or	3-5 months	13,734	1.01 (0.99 – 1.03)	0.513
general medical conditions	6-11 months	27,615	1.02 (1.00 – 1.03)	0.040
	12-17 months	21,760	1.01 (0.99 – 1.02)	0.580
	18-23 months	3,450	1.03 (1.00 – 1.07)	0.066
	24-29 months	1,933	1.00 (0.96 – 1.05)	0.928
	≥30 months	382	0.98 (0.88 – 1.08)	0.630
	SARS-CoV-2 negative	70,638	1.00 [reference]	..
	<1 month	1,742	0.84 (0.80 – 0.88)	<0.001
	1-2 months	3,463	0.82 (0.79 – 0.85)	<0.001
	3-5 months	5,067	0.82 (0.80 – 0.85)	<0.001
Mental disorders	6-11 months	9,888	0.83 (0.81 – 0.85)	<0.001
	12-17 months	7,974	0.82 (0.80 – 0.85)	<0.001
	18-23 months	1,283	0.78 (0.74 – 0.83)	<0.001
	24-29 months	718	0.78 (0.72 – 0.84)	<0.001
	≥30 months	157	0.84 (0.72 – 0.99)	0.032
	SARS-CoV-2 negative	168,329	1.00 [reference]	..
	<1 month	4,497	1.10 (1.06 – 1.13)	<0.001
	1-2 months	8,428	1.02 (1.00 – 1.05)	0.094
	3-5 months	12,155	1.03 (1.01 – 1.06)	0.002
General medical conditions	6-11 months	24,982	1.05 (1.03 – 1.07)	<0.001
	12-17 months	19,384	1.03 (1.01 – 1.04)	0.008
	18-23 months	3,059	1.08 (1.04 – 1.12)	<0.001
	24-29 months	1,788	1.07 (1.02 – 1.12)	0.004
	≥30 months	345	1.01 (0.91 – 1.12)	0.855
	SARS-CoV-2 negative	130,746	1.00 [reference]	..
	<1 month	2,760	0.95 (0.91 – 0.99)	0.010
	1-2 months	6,030	1.02 (0.99 – 1.05)	0.168
Neurological disorders	3-5 months	8,744	1.03 (1.00 – 1.06)	0.018
	6-11 months	18,191	1.03 (1.01 – 1.05)	0.003
	12-17 months	13,643	0.98 (0.95 – 1.00)	0.023
	18-23 months	2,008	0.97 (0.92 – 1.01)	0.138
	24-29 months	1,282	1.00 (0.95 – 1.06)	0.962
	≥30 months	288	1.04 (0.93 – 1.17)	0.506
	SARS-CoV-2 negative	46,302	1.00 [reference]	..
	<1 month	1,471	1.73 (1.64 – 1.83)	<0.001
	1-2 months	1,748	1.05 (1.00 – 1.11)	0.071
	3-5 months	2,376	1.00 (0.96 – 1.05)	0.844
Respiratory disorders	6-11 months	4,732	0.93 (0.90 – 0.97)	<0.001
	12-17 months	3,457	0.94 (0.90 – 0.98)	0.003
	18-23 months	577	1.03 (0.95 – 1.12)	0.482
	24-29 months	369	1.08 (0.97 – 1.19)	0.174
	≥30 months	75	1.01 (0.80 – 1.27)	0.938
	SARS-CoV-2 negative	91,490	1.00 [reference]	..
	<1 month	2,377	1.36 (1.30 – 1.42)	<0.001
	1-2 months	3,562	1.04 (1.00 – 1.08)	0.034
	3-5 months	4,894	0.98 (0.95 – 1.02)	0.306
Circulatory disorders	6-11 months	9,630	0.96 (0.93 – 0.98)	<0.001
	12-17 months	7,004	0.96 (0.93 – 0.98)	0.002
	18-23 months	1,011	0.93 (0.87 – 0.99)	0.018
	24-29 months	709	1.00 (0.93 – 1.08)	0.965
	≥30 months	141	0.91 (0.77 – 1.07)	0.243
	SARS-CoV-2 negative	19,123	1.00 [reference]	..
	<1 month	625	2.12 (1.95 – 2.31)	<0.001
	1-2 months	566	0.96 (0.88 – 1.05)	0.343

Outcome	Time since positive test	Cases, No.	HR (95% CI)[a]	p-value
Kidney disorders	3-5 months	681	0.76 (0.71 – 0.83)	<0.001
	6-11 months	1,300	0.76 (0.72 – 0.81)	<0.001
	12-17 months	919	0.76 (0.70 – 0.81)	<0.001
	18-23 months	150	0.81 (0.69 – 0.95)	0.012
	24-29 months	102	0.82 (0.67 – 0.99)	0.042
	≥30 months	28	0.93 (0.64 – 1.35)	0.693
Gastrointestinal disorders	SARS-CoV-2 negative	46,488	1.00 [reference]	..
	<1 month	955	1.01 (0.94 – 1.08)	0.782
	1-2 months	1,822	0.97 (0.92 – 1.02)	0.206
	3-5 months	2,349	0.92 (0.88 – 0.97)	0.001
	6-11 months	4,871	0.97 (0.93 – 1.00)	0.047
	12-17 months	3,555	0.94 (0.90 – 0.98)	0.002
	18-23 months	613	1.03 (0.95 – 1.11)	0.525
	24-29 months	362	0.97 (0.87 – 1.08)	0.593
	≥30 months	92	1.13 (0.92 – 1.40)	0.234
Endocrine disorders	SARS-CoV-2 negative	21,174	1.00 [reference]	..
	<1 month	713	1.85 (1.71 – 2.00)	<0.001
	1-2 months	797	1.04 (0.96 – 1.12)	0.360
	3-5 months	927	0.83 (0.77 – 0.89)	<0.001
	6-11 months	1,993	0.87 (0.83 – 0.92)	<0.001
	12-17 months	1,516	0.91 (0.86 – 0.97)	0.004
	18-23 months	241	0.95 (0.83 – 1.08)	0.406
	24-29 months	176	1.07 (0.92 – 1.24)	0.383
	≥30 months	37	1.06 (0.76 – 1.46)	0.734
Hematological disorders	SARS-CoV-2 negative	28,632	1.00 [reference]	..
	<1 month	573	1.22 (1.12 – 1.33)	<0.001
	1-2 months	962	0.98 (0.92 – 1.05)	0.606
	3-5 months	1,340	0.92 (0.86 – 0.97)	0.005
	6-11 months	2,395	0.88 (0.84 – 0.92)	<0.001
	12-17 months	1,857	0.92 (0.87 – 0.97)	0.003
	18-23 months	349	1.10 (0.99 – 1.23)	0.070
	24-29 months	212	1.06 (0.92 – 1.21)	0.430
	≥30 months	56	1.16 (0.89 – 1.51)	0.271
Musculoskeletal disorders	SARS-CoV-2 negative	23,118	1.00 [reference]	..
	<1 month	506	0.85 (0.78 – 0.94)	0.001
	1-2 months	1,214	1.01 (0.94 – 1.07)	0.858
	3-5 months	1,862	1.09 (1.03 – 1.15)	0.002
	6-11 months	3,806	1.14 (1.10 – 1.19)	<0.001
	12-17 months	2,824	1.07 (1.02 – 1.12)	0.007
	18-23 months	405	0.98 (0.89 – 1.09)	0.750
	24-29 months	252	1.02 (0.89 – 1.15)	0.816
	≥30 months	53	0.92 (0.70 – 1.22)	0.575
Dermatological disorders	SARS-CoV-2 negative	12,229	1.00 [reference]	..
	<1 month	402	1.20 (1.08 – 1.34)	0.001
	1-2 months	699	1.04 (0.96 – 1.14)	0.326
	3-5 months	990	0.99 (0.92 – 1.07)	0.753
	6-11 months	1,895	1.05 (0.99 – 1.12)	0.093
	12-17 months	1,708	1.10 (1.03 – 1.17)	0.005
	18-23 months	314	1.33 (1.19 – 1.50)	<0.001
	24-29 months	148	1.11 (0.94 – 1.31)	0.229
	≥30 months	48	1.65 (1.24 – 2.21)	0.001
Neuropsychiatric symptoms	SARS-CoV-2 negative	53,209	1.00 [reference]	..
	<1 month	1,309	1.34 (1.26 – 1.42)	<0.001
	1-2 months	2,013	1.02 (0.97 – 1.07)	0.498
	3-5 months	3,084	1.06 (1.02 – 1.11)	0.003
	6-11 months	6,002	1.09 (1.06 – 1.13)	<0.001
	12-17 months	4,558	1.12 (1.08 – 1.16)	<0.001
	18-23 months	839	1.34 (1.25 – 1.44)	<0.001
	24-29 months	508	1.31 (1.19 – 1.43)	<0.001
	≥30 months	103	1.17 (0.96 – 1.42)	0.119

Abbreviations: HR, Hazard Ratio; CI, Confidence Interval.

[a] The estimates are HRs with 95% CI from Cox Proportional Hazards model stratified by age and adjusted for confounders (sex, Charlson Comorbidity Index (CCI), parental CCI, parental mental health, employment status, income, highest level of education). All statistical tests were two-sided without correction for multiple comparisons.

Supplementary Table 10: Calendar period.

Hazard ratios of specific first mental health or general medical disorders at different calendar times. Positive SARS-CoV-2 tests were compared to negative SARS-CoV-2 tests within each calendar period.

		Cases, No.			
**Outcome**	**Calendar period**	**SARS-CoV-2 negative**	**SARS-CoV-2 positive**	**HR (95% CI)[a]**	**p-value**
Mental disorders or general medical conditions	2020-03-01	561	54	0.92 (0.69 – 1.22)	0.540
	2020-05-01	4,102	77	0.80 (0.64 – 1.01)	0.057
	2020-07-01	5,451	48	0.76 (0.57 – 1.01)	0.058
	2020-09-01	10,160	139	1.12 (0.95 – 1.33)	0.180
	2020-11-01	12,396	392	1.18 (1.06 – 1.30)	0.001
	2021-01-01	13,073	793	1.24 (1.16 – 1.34)	<0.001
	2021-03-01	14,961	835	1.07 (1.00 – 1.15)	0.051
	2021-05-01	16,177	1,043	1.06 (1.00 - 1.13)	0.068
	2021-07-01	11,936	858	1.03 (0.96 – 1.11)	0.368
	2021-09-01	14,711	1,238	1.09 (1.03 – 1.16)	0.003
	2021-11-01	14,174	1,557	1.01 (0.96 – 1.06)	0.708
	2022-01-01	10,944	4,736	0.93 (0.90 – 0.96)	<0.001
	2022-03-01	7,759	8,455	0.96 (0.93 – 0.99)	0.019
	2022-05-01	7,827	9,373	0.97 (0.94 – 1.00)	0.070
	2022-07-01	6,022	7,595	0.98 (0.94 – 1.01)	0.207
	2022-09-01	6,709	9,264	1.02 (0.99 – 1.06)	0.195
	2022-11-01	6,800	9,098	0.97 (0.94 – 1.00)	0.058
	2023-01-01	6,733	9,540	1.01 (0.98 – 1.05)	0.429
	2023-03-01	6,396	9,235	1.02 (0.99 – 1.06)	0.187
	2023-05-01	6,213	8,902	1.01 (0.98 – 1.05)	0.493
	p-value of effect modifier	..	..	..	<0.001
Mental disorders	2020-03-01	189	7	0.47 (0.22 - 1.00)	0.049
	2020-05-01	1,322	17	0.62 (0.39 - 1.01)	0.054
	2020-07-01	2,183	22	0.89 (0.59 - 1.36)	0.603
	2020-09-01	3,816	36	0.75 (0.54 - 1.05)	0.093
	2020-11-01	4,927	97	0.69 (0.56 - 0.84)	<0.001
	2021-01-01	5,410	260	0.90 (0.79 - 1.02)	0.085
	2021-03-01	5,756	281	0.83 (0.74 - 0.94)	0.003
	2021-05-01	6,263	376	0.87 (0.78 - 0.96)	0.007
	2021-07-01	4,975	333	0.84 (0.75 - 0.93)	0.002
	2021-09-01	5,818	471	0.89 (0.81 - 0.98)	0.014
	2021-11-01	5,552	579	0.83 (0.76 - 0.91)	<0.001
	2022-01-01	4,266	1,848	0.77 (0.72 - 0.81)	<0.001
	2022-03-01	2,954	3,127	0.79 (0.75 - 0.84)	<0.001
	2022-05-01	3,035	3,484	0.78 (0.75 - 0.83)	<0.001
	2022-07-01	2,367	2,854	0.80 (0.76 - 0.85)	<0.001
	2022-09-01	2,553	3,284	0.82 (0.78 - 0.87)	<0.001
	2022-11-01	2,418	3,260	0.83 (0.79 - 0.88)	<0.001
	2023-01-01	2,387	3,432	0.88 (0.83 - 0.93)	<0.001
	2023-03-01	2,224	3,335	0.91 (0.86 - 0.96)	<0.001
	2023-05-01	2,223	3,189	0.86 (0.81 - 0.91)	<0.001
	p-value of effect modifier	..	..	..	0.001
General medical conditions	2020-03-01	593	54	0.88 (0.66 - 1.17)	0.378
	2020-05-01	4,094	76	0.81 (0.65 - 1.02)	0.069
	2020-07-01	5,166	40	0.68 (0.50 - 0.93)	0.014
	2020-09-01	9,435	135	1.21 (1.02 - 1.43)	0.030
	2020-11-01	11,192	378	1.32 (1.19 - 1.46)	<0.001
	2021-01-01	11,591	724	1.34 (1.25 - 1.45)	<0.001
	2021-03-01	13,489	782	1.17 (1.09 - 1.26)	<0.001
	2021-05-01	14,444	936	1.13 (1.06 - 1.21)	<0.001
	2021-07-01	10,748	737	1.06 (0.98 - 1.14)	0.150
	2021-09-01	13,194	1,101	1.17 (1.10 - 1.24)	<0.001
	2021-11-01	12,659	1,362	1.06 (1.00 - 1.12)	0.037
	2022-01-01	10,033	4,090	0.96 (0.93 - 1.00)	0.051
	2022-03-01	7,263	7,497	0.99 (0.96 - 1.03)	0.692
	2022-05-01	7,324	8,222	0.99 (0.96 - 1.03)	0.641

	2022-07-01	5,670	6,749	1.00 (0.97 - 1.04)	0.857
	2022-09-01	6,390	8,461	1.05 (1.02 - 1.09)	0.002
	2022-11-01	6,467	8,220	0.99 (0.96 - 1.03)	0.676
	2023-01-01	6,472	8,660	1.03 (0.99 - 1.06)	0.120
	2023-03-01	6,131	8,356	1.04 (1.00 - 1.07)	0.027
	2023-05-01	5,974	8,058	1.02 (0.99 - 1.06)	0.203
	p-value of effect modifier	..	..	..	<0.001
Neurological disorders	2020-03-01	239	23	1.23 (0.79 - 1.89)	0.357
	2020-05-01	2,465	43	0.82 (0.61 - 1.11)	0.201
	2020-07-01	3,414	31	0.82 (0.57 - 1.16)	0.258
	2020-09-01	6,613	73	0.96 (0.77 - 1.21)	0.754
	2020-11-01	8,082	180	0.95 (0.82 - 1.10)	0.483
	2021-01-01	8,899	404	1.04 (0.94 - 1.15)	0.483
	2021-03-01	10,041	482	1.03 (0.94 - 1.13)	0.544
	2021-05-01	10,518	573	1.04 (0.96 - 1.13)	0.367
	2021-07-01	7,201	379	0.90 (0.81 - 1.00)	0.050
	2021-09-01	9,805	672	1.08 (1.00 - 1.17)	0.056
	2021-11-01	9,462	758	0.91 (0.84 - 0.98)	0.009
	2022-01-01	8,021	2,553	0.91 (0.87 - 0.95)	<0.001
	2022-03-01	6,308	5,098	0.97 (0.93 - 1.01)	0.115
	2022-05-01	6,521	5,841	0.97 (0.94 - 1.01)	0.128
	2022-07-01	4,752	4,792	1.02 (0.98 - 1.06)	0.390
	2022-09-01	5,795	6,220	1.04 (1.00 - 1.08)	0.050
	2022-11-01	5,656	6,031	1.00 (0.96 - 1.04)	0.939
	2023-01-01	5,887	6,626	1.04 (1.00 - 1.07)	0.056
	2023-03-01	5,526	6,172	1.01 (0.98 - 1.05)	0.502
	2023-05-01	5,541	5,995	0.98 (0.94 - 1.01)	0.195
	p-value of effect modifier	..	..	..	<0.001
Respiratory disorders	2020-03-01	417	68	1.79 (1.38 - 2.32)	<0.001
	2020-05-01	1,949	28	0.70 (0.48 - 1.01)	0.058
	2020-07-01	2,066	29	1.29 (0.89 - 1.86)	0.177
	2020-09-01	3,179	60	1.72 (1.33 - 2.22)	<0.001
	2020-11-01	3,204	162	2.26 (1.93 - 2.65)	<0.001
	2021-01-01	2,862	229	1.91 (1.67 - 2.19)	<0.001
	2021-03-01	3,287	237	1.62 (1.42 - 1.85)	<0.001
	2021-05-01	3,465	254	1.47 (1.30 - 1.67)	<0.001
	2021-07-01	2,416	151	1.16 (0.99 - 1.37)	0.074
	2021-09-01	3,376	234	1.14 (1.00 - 1.30)	0.051
	2021-11-01	3,278	369	1.32 (1.18 - 1.47)	<0.001
	2022-01-01	2,428	743	1.00 (0.92 - 1.10)	0.939
	2022-03-01	1,983	1,468	1.03 (0.96 - 1.10)	0.489
	2022-05-01	1,967	1,531	0.92 (0.85 - 0.98)	0.013
	2022-07-01	1,495	1,152	0.90 (0.83 - 0.97)	0.007
	2022-09-01	1,854	1,536	0.87 (0.81 - 0.93)	<0.001
	2022-11-01	1,915	1,737	0.93 (0.87 - 1.00)	0.047
	2023-01-01	1,846	1,714	0.97 (0.91 - 1.04)	0.446
	2023-03-01	1,713	1,521	0.92 (0.86 - 0.99)	0.028
	2023-05-01	1,602	1,582	1.01 (0.94 - 1.08)	0.844
	p-value of effect modifier	..	..	..	<0.001
Circulatory disorders	2020-03-01	526	60	1.11 (0.84 - 1.45)	0.464
	2020-05-01	2,518	49	0.85 (0.64 - 1.12)	0.250
	2020-07-01	3,046	29	0.83 (0.58 - 1.20)	0.320
	2020-09-01	4,652	78	1.49 (1.19 - 1.87)	<0.001
	2020-11-01	5,653	209	1.64 (1.43 - 1.89)	<0.001
	2021-01-01	6,202	386	1.52 (1.37 - 1.69)	<0.001
	2021-03-01	6,923	381	1.28 (1.15 - 1.42)	<0.001
	2021-05-01	6,885	372	1.15 (1.04 - 1.28)	0.009
	2021-07-01	5,569	314	1.10 (0.98 - 1.23)	0.118
	2021-09-01	6,414	393	1.11 (1.00 - 1.23)	0.041
	2021-11-01	6,403	540	1.10 (1.01 - 1.20)	0.034
	2022-01-01	5,728	1,422	0.93 (0.87 - 0.98)	0.014
	2022-03-01	4,272	2,868	0.99 (0.94 - 1.04)	0.669
	2022-05-01	4,287	3,078	0.93 (0.89 - 0.98)	0.004
	2022-07-01	3,545	2,721	0.93 (0.88 - 0.97)	0.003
	2022-09-01	3,887	3,178	0.93 (0.88 - 0.97)	0.002
	2022-11-01	3,858	3,309	0.95 (0.91 - 1.00)	0.054
	2023-01-01	3,955	3,515	0.96 (0.92 - 1.01)	0.090
	2023-03-01	3,604	3,309	0.99 (0.94 - 1.04)	0.624
	2023-05-01	3,563	3,117	0.93 (0.89 - 0.98)	0.004
	p-value of effect modifier	..	..	..	<0.001
	2020-03-01	154	46	3.63 (2.60 - 5.07)	<0.001
	2020-05-01	613	12	0.92 (0.52 - 1.64)	0.784
	2020-07-01	809	≤5[b]	..	..
	2020-09-01	912	16	1.64 (1.00 - 2.69)	0.051
	2020-11-01	1,066	55	2.54 (1.93 - 3.33)	<0.001
	2021-01-01	1,097	74	1.75 (1.39 - 2.22)	<0.001

Kidney disorders	2021-03-01	1,213	54	1.12 (0.86 - 1.48)	0.403
	2021-05-01	1,223	49	0.97 (0.73 - 1.29)	0.820
	2021-07-01	1,075	47	1.02 (0.76 - 1.37)	0.872
	2021-09-01	1,160	62	1.15 (0.89 - 1.49)	0.271
	2021-11-01	1,206	93	1.26 (1.02 - 1.56)	0.032
	2022-01-01	1,106	237	1.20 (1.04 - 1.39)	0.014
	2022-03-01	986	433	0.93 (0.83 - 1.04)	0.217
	2022-05-01	1,030	464	0.83 (0.74 - 0.93)	0.001
	2022-07-01	900	388	0.75 (0.66 - 0.84)	<0.001
	2022-09-01	982	437	0.70 (0.63 - 0.79)	<0.001
	2022-11-01	925	437	0.74 (0.66 - 0.83)	<0.001
	2023-01-01	878	467	0.79 (0.71 - 0.89)	<0.001
	2023-03-01	906	486	0.80 (0.71 - 0.89)	<0.001
	2023-05-01	882	509	0.85 (0.76 - 0.95)	0.005
	p-value of effect modifier	..	..	..	<0.001
Gastrointestinal disorders	2020-03-01	230	≤5[b]	..	..
	2020-05-01	1,846	14	0.34 (0.20 - 0.58)	<0.001
	2020-07-01	2,097	10	0.41 (0.22 - 0.75)	0.004
	2020-09-01	3,071	13	0.36 (0.21 - 0.62)	<0.001
	2020-11-01	3,380	68	0.86 (0.67 - 1.09)	0.209
	2021-01-01	3,241	143	0.98 (0.83 - 1.16)	0.798
	2021-03-01	3,724	163	0.92 (0.79 - 1.08)	0.297
	2021-05-01	3,702	188	0.95 (0.82 - 1.10)	0.508
	2021-07-01	2,591	146	0.98 (0.83 - 1.16)	0.811
	2021-09-01	3,275	197	0.92 (0.80 - 1.07)	0.278
	2021-11-01	3,218	257	0.90 (0.80 - 1.03)	0.122
	2022-01-01	2,704	771	0.88 (0.81 - 0.96)	0.003
	2022-03-01	1,926	1,561	1.00 (0.93 - 1.07)	0.963
	2022-05-01	1,915	1,562	0.92 (0.86 - 0.98)	0.015
	2022-07-01	1,489	1,255	0.93 (0.86 - 1.01)	0.077
	2022-09-01	1,728	1,710	1.01 (0.94 - 1.08)	0.840
	2022-11-01	1,660	1,643	0.97 (0.90 - 1.04)	0.361
	2023-01-01	1,638	1,708	0.98 (0.92 - 1.06)	0.650
	2023-03-01	1,585	1,633	0.98 (0.91 - 1.05)	0.594
	2023-05-01	1,468	1,573	1.01 (0.94 - 1.08)	0.829
	p-value of effect modifier	..	..	..	<0.001
Endocrine disorders	2020-03-01	147	32	2.67 (1.81 - 3.94)	<0.001
	2020-05-01	613	17	1.37 (0.85 - 2.22)	0.202
	2020-07-01	742	9	1.12 (0.58 - 2.16)	0.734
	2020-09-01	1,148	23	1.84 (1.22 - 2.78)	0.004
	2020-11-01	1,264	96	3.46 (2.81 - 4.26)	<0.001
	2021-01-01	1,398	125	2.21 (1.84 - 2.65)	<0.001
	2021-03-01	1,588	104	1.54 (1.26 - 1.87)	<0.001
	2021-05-01	1,594	75	1.01 (0.80 - 1.28)	0.905
	2021-07-01	1,186	70	1.17 (0.91 - 1.48)	0.215
	2021-09-01	1,425	112	1.42 (1.17 - 1.72)	<0.001
	2021-11-01	1,397	160	1.49 (1.26 - 1.76)	<0.001
	2022-01-01	1,297	364	1.07 (0.94 - 1.21)	0.307
	2022-03-01	1,029	606	0.89 (0.80 - 0.99)	0.025
	2022-05-01	1,006	611	0.82 (0.74 - 0.91)	<0.001
	2022-07-01	837	507	0.75 (0.67 - 0.84)	<0.001
	2022-09-01	950	693	0.87 (0.78 - 0.96)	0.005
	2022-11-01	858	710	0.96 (0.87 - 1.07)	0.479
	2023-01-01	985	717	0.80 (0.72 - 0.88)	<0.001
	2023-03-01	883	711	0.89 (0.81 - 0.99)	0.028
	2023-05-01	827	658	0.90 (0.81 - 1.01)	0.062
	p-value of effect modifier	..	..	..	<0.001
Hematological disorders	2020-03-01	184	8	0.55 (0.27 - 1.12)	0.099
	2020-05-01	1,066	11	0.47 (0.26 - 0.85)	0.013
	2020-07-01	1,342	10	0.65 (0.35 - 1.21)	0.170
	2020-09-01	1,678	15	0.81 (0.49 - 1.35)	0.425
	2020-11-01	1,709	33	0.93 (0.66 - 1.31)	0.662
	2021-01-01	1,673	84	1.25 (1.00 - 1.56)	0.045
	2021-03-01	1,799	76	1.01 (0.80 - 1.27)	0.931
	2021-05-01	2,124	96	0.99 (0.81 - 1.22)	0.927
	2021-07-01	1,592	89	1.17 (0.94 - 1.45)	0.156
	2021-09-01	1,915	102	1.02 (0.83 - 1.25)	0.851
	2021-11-01	1,742	130	1.04 (0.87 - 1.24)	0.696
	2022-01-01	1,550	338	0.98 (0.87 - 1.11)	0.753
	2022-03-01	1,398	750	0.95 (0.86 - 1.04)	0.242
	2022-05-01	1,498	854	0.88 (0.80 - 0.96)	0.003
	2022-07-01	1,262	756	0.89 (0.81 - 0.98)	0.019
	2022-09-01	1,270	838	0.93 (0.85 - 1.02)	0.122
	2022-11-01	1,231	831	0.92 (0.84 - 1.01)	0.068
	2023-01-01	1,214	910	0.96 (0.88 - 1.05)	0.334
	2023-03-01	1,106	812	0.94 (0.86 - 1.03)	0.213
	2023-05-01	1,279	1,001	0.99 (0.91 - 1.08)	0.877
	p-value of effect modifier	..	..	..	0.066

Musculoskeletal disorders	2020-03-01	44	≤5[b]	..	..
	2020-05-01	359	7	0.92 (0.43 - 1.94)	0.826
	2020-07-01	612	≤5[b]	..	..
	2020-09-01	1,195	15	1.03 (0.62 - 1.71)	0.917
	2020-11-01	1,436	20	0.56 (0.36 - 0.87)	0.010
	2021-01-01	1,702	58	0.72 (0.56 - 0.94)	0.015
	2021-03-01	1,854	92	0.96 (0.78 - 1.19)	0.712
	2021-05-01	2,099	96	0.79 (0.64 - 0.97)	0.023
	2021-07-01	1,557	100	1.00 (0.82 - 1.23)	0.991
	2021-09-01	1,884	123	0.92 (0.77 - 1.11)	0.378
	2021-11-01	1,804	147	0.83 (0.70 - 0.98)	0.033
	2022-01-01	1,413	544	0.90 (0.81 - 1.00)	0.044
	2022-03-01	1,056	1,087	1.03 (0.94 - 1.12)	0.547
	2022-05-01	1,079	1,263	1.05 (0.97 - 1.14)	0.239
	2022-07-01	790	1,032	1.15 (1.05 - 1.27)	0.004
	2022-09-01	918	1,367	1.19 (1.09 - 1.30)	<0.001
	2022-11-01	812	1,183	1.14 (1.04 - 1.25)	0.004
	2023-01-01	824	1,298	1.21 (1.10 - 1.32)	<0.001
	2023-03-01	862	1,225	1.07 (0.98 - 1.17)	0.128
	2023-05-01	818	1,260	1.18 (1.08 - 1.30)	<0.001
	p-value of effect modifier	..	..	..	<0.001
Dermatological disorders	2020-03-01	51	≤5[b]	..	..
	2020-05-01	295	10	1.79 (0.95 - 3.37)	0.070
	2020-07-01	446	8	1.68 (0.84 - 3.39)	0.145
	2020-09-01	678	8	1.02 (0.51 - 2.05)	0.949
	2020-11-01	801	32	1.51 (1.06 - 2.16)	0.022
	2021-01-01	838	78	1.92 (1.52 - 2.42)	<0.001
	2021-03-01	1,000	55	1.04 (0.79 - 1.37)	0.762
	2021-05-01	1,153	80	1.14 (0.91 - 1.43)	0.266
	2021-07-01	1,013	89	1.27 (1.02 - 1.57)	0.033
	2021-09-01	945	91	1.23 (0.99 - 1.53)	0.057
	2021-11-01	777	104	1.14 (0.93 - 1.40)	0.214
	2022-01-01	733	372	0.94 (0.82 - 1.07)	0.369
	2022-03-01	492	663	1.07 (0.95 - 1.21)	0.251
	2022-05-01	476	659	1.05 (0.93 - 1.18)	0.464
	2022-07-01	487	649	0.97 (0.86 - 1.09)	0.598
	2022-09-01	375	623	1.18 (1.03 - 1.35)	0.014
	2022-11-01	401	598	0.99 (0.87 - 1.13)	0.933
	2023-01-01	402	641	1.04 (0.91 - 1.18)	0.585
	2023-03-01	447	673	0.95 (0.84 - 1.08)	0.447
	2023-05-01	419	771	1.23 (1.08 - 1.38)	0.001
	p-value of effect modifier	..	..	..	<0.001
Neuropsychiatric symptoms	2020-03-01	212	6	0.85 (0.37 - 1.92)	0.689
	2020-05-01	1,321	8	0.84 (0.42 - 1.68)	0.618
	2020-07-01	2,021	27	3.83 (2.62 - 5.61)	<0.001
	2020-09-01	2,892	11	1.69 (0.93 - 3.05)	0.084
	2020-11-01	3,385	25	2.64 (1.78 - 3.91)	<0.001
	2021-01-01	3,269	39	2.47 (1.80 - 3.38)	<0.001
	2021-03-01	4,054	44	2.26 (1.68 - 3.05)	<0.001
	2021-05-01	4,673	43	1.95 (1.44 - 2.64)	<0.001
	2021-07-01	4,252	30	1.49 (1.04 - 2.14)	0.029
	2021-09-01	4,178	37	1.82 (1.32 - 2.52)	<0.001
	2021-11-01	3,936	50	2.32 (1.76 - 3.07)	<0.001
	2022-01-01	3,809	98	3.33 (2.72 - 4.07)	<0.001
	2022-03-01	3,894	148	3.28 (2.78 - 3.87)	<0.001
	2022-05-01	4,279	159	3.06 (2.61 - 3.59)	<0.001
	2022-07-01	3,749	147	2.93 (2.48 - 3.46)	<0.001
	2022-09-01	4,223	130	2.22 (1.86 - 2.65)	<0.001
	2022-11-01	3,835	158	2.79 (2.37 - 3.27)	<0.001
	2023-01-01	3,984	150	2.45 (2.08 - 2.89)	<0.001
	2023-03-01	4,126	127	2.07 (1.73 - 2.47)	<0.001
	2023-05-01	3,954	142	2.32 (1.96 - 2.75)	<0.001
	p-value of effect modifier	..	..	..	<0.001

Abbreviations: HR, Hazard Ratio; CI, Confidence Interval

[a] The estimates are HRs with 95% CI from Cox Proportional Hazards model stratified by age and adjusted for confounders (sex, Charlson Comorbidity Index (CCI), parental CCI, parental mental health, employment status, income, and highest level of education). All statistical tests were two-sided without correction for multiple comparisons.

[b] Results from ≤5 patients are displayed as "≤5" to ensure data privacy.

Results: Positive test compared with other infections

Supplementary Table 11: Positive test compared with prescription for anti-infective agents.

Hazard ratios of specific first mental health or general medical disorders among individuals with a positive SARS-CoV-2 test compared to individuals with a prescription for anti-infective agents.

Outcome	Infection type[b]	Cases, No.	HR (95% CI)[a]	p-value
Mental disorders or general medical conditions	No infection	95,927	..	..
	Any prescription for anti-infective agents	23,484	1.60 (1.57 - 1.62)	<0.001
	Antibacterial	21,177	1.59 (1.56 - 1.61)	<0.001
	Antivirals	915	1.34 (1.26 - 1.43)	<0.001
	Antimycotics	791	1.79 (1.67 - 1.92)	<0.001
	SARS-CoV-2 positive	54,498	1.12 (1.11 - 1.13)	<0.001
	SARS-CoV-2 positive vs. prescription	54,498	0.71 (0.69 - 0.72)	<0.001
Mental disorders	No infection	32,782	1.00 [reference]	..
	Any prescription for anti-infective agents	8,951	1.41 (1.38 - 1.44)	<0.001
	SARS-CoV-2 positive	18,394	0.89 (0.87 - 0.91)	<0.001
	SARS-CoV-2 positive vs. prescription	18,394	0.63 (0.62 - 0.65)	<0.001
General medical conditions	No infection	85,560	1.00 [reference]	..
	Any prescription for anti-infective agents	22,209	1.65 (1.62 - 1.67)	<0.001
	SARS-CoV-2 positive	48,231	1.18 (1.16 - 1.19)	<0.001
	SARS-CoV-2 positive vs. prescription	48,231	0.72 (0.70 - 0.73)	<0.001
Neurological disorders	No infection	59,569	1.00 [reference]	..
	Any prescription for anti-infective agents	17,033	1.32 (1.30 - 1.35)	<0.001
	SARS-CoV-2 positive	32,013	1.09 (1.07 - 1.10)	<0.001
	SARS-CoV-2 positive vs. prescription	32,013	0.82 (0.80 - 0.84)	<0.001
Respiratory disorders	No infection	15,203	1.00 [reference]	..
	Any prescription for anti-infective agents	7,505	2.45 (2.38 - 2.53)	<0.001
	SARS-CoV-2 positive	7,688	1.38 (1.34 - 1.43)	<0.001
	SARS-CoV-2 positive vs. prescription	7,688	0.56 (0.54 - 0.58)	<0.001
Circulatory disorders	No infection	40,309	1.00 [reference]	..
	Any prescription for anti-infective agents	12,892	1.50 (1.47 - 1.53)	<0.001
	SARS-CoV-2 positive	17,385	1.13 (1.11 - 1.15)	<0.001
	SARS-CoV-2 positive vs. prescription	17,385	0.75 (0.74 - 0.77)	<0.001
Kidney disorders	No infection	4,903	1.00 [reference]	..
	Any prescription for anti-infective agents	3,343	2.15 (2.06 - 2.25)	<0.001
	SARS-CoV-2 positive	1,981	1.20 (1.13 - 1.27)	<0.001
	SARS-CoV-2 positive vs. prescription	1,981	0.56 (0.52 - 0.59)	<0.001
Gastrointestinal disorders	No infection	18,129	1.00 [reference]	..
	Any prescription for anti-infective agents	6,491	1.71 (1.66 - 1.76)	<0.001
	SARS-CoV-2 positive	8,008	1.14 (1.10 - 1.18)	<0.001
	SARS-CoV-2 positive vs. prescription	8,008	0.67 (0.64 - 0.69)	<0.001
Endocrine disorders	No infection	7,788	1.00 [reference]	..
	Any prescription for anti-infective agents	3,284	1.77 (1.70 - 1.85)	<0.001
	SARS-CoV-2 positive	3,557	1.17 (1.12 - 1.23)	<0.001
	SARS-CoV-2 positive vs. prescription	3,557	0.66 (0.63 - 0.69)	<0.001
Hematological disorders	No infection	8,405	1.00 [reference]	..
	Any prescription for anti-infective agents	4,790	1.99 (1.92 - 2.07)	<0.001
	SARS-CoV-2 positive	3,936	1.27 (1.22 - 1.33)	<0.001
	SARS-CoV-2 positive vs. prescription	3,936	0.64 (0.61 - 0.67)	<0.001
Musculoskeletal disorders	No infection	9,219	1.00 [reference]	..
	Any prescription for anti-infective agents	3,128	1.57 (1.51 - 1.64)	<0.001
	SARS-CoV-2 positive	6,055	1.24 (1.19 - 1.29)	<0.001
	SARS-CoV-2 positive vs. prescription	6,055	0.79 (0.75 - 0.82)	<0.001
Dermatological disorders	No infection	4,393	1.00 [reference]	..
	Any prescription for anti-infective agents	2,132	2.79 (2.65 - 2.95)	<0.001
	SARS-CoV-2 positive	3,546	1.53 (1.44 - 1.61)	<0.001
	SARS-CoV-2 positive vs. prescription	3,546	0.55 (0.51 - 0.58)	<0.001
Neuropsychiatric symptoms	No infection	17,956	1.00 [reference]	..
	Any prescription for anti-infective agents	8,408	1.89 (1.84 - 1.94)	<0.001
	SARS-CoV-2 positive	9,678	1.40 (1.36 - 1.44)	<0.001
	SARS-CoV-2 positive vs. prescription	9,678	0.74 (0.72 - 0.76)	<0.001

Abbreviations: HR, Hazard Ratio; CI, Confidence Interval.

[a] The estimates are HRs with 95% CI from Cox Proportional Hazards model stratified by age and adjusted for confounders (sex, employment status, income, the highest level of education, Charlson Comorbidity Index, parental Charlson Comorbidity Index, and parental history of mental disorders). All statistical tests were two-sided without correction for multiple comparisons.
[b] Excluding individuals with a redeemed prescription for any anti-infective agent within the past year to rule out recurring infections. The reference group *no infection* consisted of all individuals without a prescription for an anti-infective agent and negative test results.

Results: Admission with COVID-19 compared with no admission with COVID-19
Supplementary Table 12: Intensive care unit (ICU) status.

Hazard ratios of specific first mental health or general medical disorders among individuals admitted with COVID-19 with and without intensive care unit (ICU) admission compared to individuals without admission to a hospital with COVID-19.

Outcome	Admission status	Cases, No.	HR (95% CI)[a]	p-value
Mental disorders or general medical conditions	No admission	264,846	1.00 [reference]	..
	Admission without ICU	1,345	2.48 (2.35 - 2.62)	<0.001
	Admission with ICU	146	5.29 (4.50 - 6.23)	<0.001
Mental disorders	No admission	100,111	1.00 [reference]	..
	Admission without ICU	751	1.88 (1.75 - 2.03)	<0.001
	Admission with ICU	68	2.49 (1.96 - 3.15)	<0.001
General medical conditions	No admission	241,417	1.00 [reference]	..
	Admission without ICU	1,401	2.53 (2.40 - 2.67)	<0.001
	Admission with ICU	149	5.09 (4.34 - 5.98)	<0.001
Neurological disorders	No admission	181,879	1.00 [reference]	..
	Admission without ICU	1,637	1.67 (1.59 - 1.76)	<0.001
	Admission with ICU	176	2.29 (1.98 - 2.66)	<0.001
Respiratory disorders	No admission	59,608	1.00 [reference]	..
	Admission without ICU	1,294	3.09 (2.92 - 3.27)	<0.001
	Admission with ICU	205	7.77 (6.77 - 8.92)	<0.001
Circulatory disorders	No admission	119,189	1.00 [reference]	..
	Admission without ICU	1,442	2.26 (2.14 - 2.38)	<0.001
	Admission with ICU	187	4.07 (3.53 - 4.70)	<0.001
Kidney disorders	No admission	22,579	1.00 [reference]	..
	Admission without ICU	777	2.37 (2.21 - 2.55)	<0.001
	Admission with ICU	138	7.43 (6.28 - 8.78)	<0.001
Gastrointestinal disorders	No admission	60,312	1.00 [reference]	..
	Admission without ICU	733	1.87 (1.74 - 2.01)	<0.001
	Admission with ICU	62	2.00 (1.56 - 2.57)	<0.001
Endocrine disorders	No admission	26,956	1.00 [reference]	..
	Admission without ICU	540	2.49 (2.28 - 2.71)	<0.001
	Admission with ICU	78	4.55 (3.64 - 5.68)	<0.001
Hematological disorders	No admission	35,294	1.00 [reference]	..
	Admission without ICU	1,002	2.45 (2.30 - 2.61)	<0.001
	Admission with ICU	80	3.07 (2.46 - 3.82)	<0.001
Musculoskeletal disorders	No admission	33,823	1.00 [reference]	..
	Admission without ICU	201	1.10 (0.95 - 1.26)	0.191
	Admission with ICU	16	1.17 (0.72 - 1.91)	0.526
Dermatological disorders	No admission	18,256	1.00 [reference]	..
	Admission without ICU	156	1.90 (1.62 - 2.23)	<0.001
	Admission with ICU	21	3.71 (2.42 - 5.70)	<0.001
Neuropsychiatric symptoms	No admission	70,046	1.00 [reference]	..
	Admission without ICU	1,456	2.46 (2.34 - 2.60)	<0.001
	Admission with ICU	123	2.93 (2.46 - 3.50)	<0.001

Abbreviations: HR, Hazard Ratio; CI, Confidence Interval; ICU, Intensive Care Unit.

[a] The estimates are HRs with 95% CI from Cox Proportional Hazards model stratified by age and adjusted for confounders (sex, Charlson Comorbidity Index (CCI), parental CCI, parental mental health, employment status, income, highest level of education). All statistical tests were two-sided without correction for multiple comparisons.
[b] The reference group *no COVID-19-admission* consisted of individuals without admission to a hospital with SARS-CoV-2 infection, i.e., all individuals with negative or positive test results but no hospital admission.

Supplementary Table 13: Age group.

Hazard ratios of specific first mental health or general medical disorders among individuals with admission with COVID-19 compared to individuals without admission with COVID-19 by age group.

Outcome	Age group	Admission	Cases, No.	HR (95% CI)[a]	p-value
Mental disorders or general medical conditions	<18	No admission	50,034	1.00 [reference]	..
		Admission without ICU	106	3.93 (3.25 - 4.75)	<0.001
		Admission with ICU	6	4.40 (1.98 - 9.80)	<0.001
	18-39	No admission	72,889	1.00 [reference]	..
		Admission without ICU	342	1.96 (1.76 - 2.18)	<0.001
		Admission with ICU	20	5.24 (3.38 - 8.12)	<0.001
	40-59	No admission	74,576	1.00 [reference]	..
		Admission without ICU	350	2.62 (2.36 - 2.92)	<0.001
		Admission with ICU	53	7.25 (5.54 - 9.50)	<0.001
	60-79	No admission	59,434	1.00 [reference]	..
		Admission without ICU	408	2.96 (2.68 - 3.26)	<0.001
		Admission with ICU	62	5.40 (4.21 - 6.93)	<0.001
	≥80	No admission	7,913	1.00 [reference]	..
		Admission without ICU	139	1.99 (1.68 - 2.35)	<0.001
		Admission with ICU	≤5[b]	..	..
Mental disorders	<40	No admission	63,987	1.00 [reference]	..
		Admission without ICU	244	1.52 (1.34 - 1.72)	<0.001
		Admission with ICU	16	2.35 (1.44 - 3.84)	0.001
	40-59	No admission	21,582	1.00 [reference]	..
		Admission without ICU	154	1.80 (1.54 - 2.11)	<0.001
		Admission with ICU	15	1.64 (0.99 - 2.72)	0.056
	60-79	No admission	11,253	1.00 [reference]	..
		Admission without ICU	232	2.69 (2.36 - 3.07)	<0.001
		Admission with ICU	31	3.25 (2.28 - 4.62)	<0.001
	≥80	No admission	3,289	1.00 [reference]	..
		Admission without ICU	121	1.85 (1.54 - 2.22)	<0.001
		Admission with ICU	6	3.42 (1.54 - 7.63)	0.003
General medical conditions	<40	No admission	96,611	1.00 [reference]	..
		Admission without ICU	437	2.46 (2.24 - 2.71)	<0.001
		Admission with ICU	25	5.07 (3.43 - 7.51)	<0.001
	40-59	No admission	74,318	1.00 [reference]	..
		Admission without ICU	381	2.59 (2.34 - 2.87)	<0.001
		Admission with ICU	56	7.01 (5.39 - 9.11)	<0.001
	60-79	No admission	62,296	1.00 [reference]	..
		Admission without ICU	439	2.85 (2.60 - 3.13)	<0.001
		Admission with ICU	64	4.99 (3.90 - 6.37)	<0.001
	≥80	No admission	8,192	1.00 [reference]	..
		Admission without ICU	144	1.90 (1.61 - 2.24)	<0.001
		Admission with ICU	≤5[b]	..	..
Neurological disorders	<40	No admission	54,747	1.00 [reference]	..
		Admission without ICU	260	1.97 (1.74 - 2.22)	<0.001
		Admission with ICU	25	5.03 (3.40 - 7.44)	<0.001
	40-59	No admission	48,501	1.00 [reference]	..
		Admission without ICU	323	1.90 (1.70 - 2.12)	<0.001
		Admission with ICU	53	3.34 (2.55 - 4.38)	<0.001
	60-79	No admission	64,466	1.00 [reference]	..
		Admission without ICU	717	1.73 (1.61 - 1.86)	<0.001
		Admission with ICU	91	1.94 (1.58 - 2.38)	<0.001
	≥80	No admission	14,165	1.00 [reference]	..
		Admission without ICU	337	1.27 (1.14 - 1.42)	<0.001
		Admission with ICU	7	0.77 (0.37 - 1.62)	0.489

Outcome	Age group	Admission	Cases, No.	HR (95% CI)[a]	p-value
Respiratory disorders	<40	No admission	16,163	1.00 [reference]	··
		Admission without ICU	109	3.80 (3.14 - 4.59)	<0.001
		Admission with ICU	13	10.78 (6.26 - 18.57)	<0.001
	40-59	No admission	13,096	1.00 [reference]	··
		Admission without ICU	238	4.25 (3.74 - 4.83)	<0.001
		Admission with ICU	58	12.19 (9.41 - 15.78)	<0.001
	60-79	No admission	23,357	1.00 [reference]	··
		Admission without ICU	630	3.26 (3.01 - 3.53)	<0.001
		Admission with ICU	115	6.65 (5.53 - 7.99)	<0.001
	≥80	No admission	6,992	1.00 [reference]	··
		Admission without ICU	317	2.22 (1.98 - 2.48)	<0.001
		Admission with ICU	19	6.10 (3.89 - 9.58)	<0.001
Circulatory disorders	<40	No admission	13,483	1.00 [reference]	··
		Admission without ICU	145	3.66 (3.10 - 4.31)	<0.001
		Admission with ICU	18	11.74 (7.39 - 18.64)	<0.001
	40-59	No admission	38,132	1.00 [reference]	··
		Admission without ICU	331	2.62 (2.35 - 2.92)	<0.001
		Admission with ICU	74	7.19 (5.73 - 9.04)	<0.001
	60-79	No admission	54,745	1.00 [reference]	··
		Admission without ICU	648	2.31 (2.14 - 2.50)	<0.001
		Admission with ICU	81	2.95 (2.37 - 3.67)	<0.001
	≥80	No admission	12,829	1.00 [reference]	··
		Admission without ICU	318	1.63 (1.46 - 1.83)	<0.001
		Admission with ICU	14	2.06 (1.22 - 3.48)	0.007
Kidney disorders	<40	No admission	1,350	1.00 [reference]	··
		Admission without ICU	33	8.85 (6.24 - 12.54)	<0.001
		Admission with ICU	7	33.62 (15.97 - 70.78)	<0.001
	40-59	No admission	2,859	1.00 [reference]	··
		Admission without ICU	70	3.93 (3.09 - 4.99)	<0.001
		Admission with ICU	38	20.32 (14.74 - 28.01)	<0.001
	60-79	No admission	11,313	1.00 [reference]	··
		Admission without ICU	381	2.88 (2.59 - 3.19)	<0.001
		Admission with ICU	85	7.04 (5.68 - 8.72)	<0.001
	≥80	No admission	7,057	1.00 [reference]	··
		Admission without ICU	293	1.66 (1.48 - 1.87)	<0.001
		Admission with ICU	8	1.82 (0.91 - 3.64)	0.090
Gastrointestinal disorders	<40	No admission	15,216	1.00 [reference]	··
		Admission without ICU	124	2.83 (2.37 - 3.38)	<0.001
		Admission with ICU	10	5.47 (2.94 - 10.18)	<0.001
	40-59	No admission	18,669	1.00 [reference]	··
		Admission without ICU	142	1.97 (1.67 - 2.33)	<0.001
		Admission with ICU	18	2.39 (1.50 - 3.79)	<0.001
	60-79	No admission	21,412	1.00 [reference]	··
		Admission without ICU	331	1.93 (1.73 - 2.15)	<0.001
		Admission with ICU	31	1.64 (1.16 - 2.34)	0.006
	≥80	No admission	5,015	1.00 [reference]	··
		Admission without ICU	136	1.28 (1.08 - 1.52)	0.005
		Admission with ICU	≤5[b]	..	..
Endocrine disorders	<40	No admission	3,769	1.00 [reference]	··
		Admission without ICU	29	2.84 (1.97 - 4.10)	<0.001
		Admission with ICU	≤5[b]	..	..
	40-59	No admission	7,962	1.00 [reference]	··
		Admission without ICU	127	3.41 (2.86 - 4.07)	<0.001
		Admission with ICU	32	8.26 (5.84 - 11.70)	<0.001
	60-79	No admission	12,273	1.00 [reference]	··
		Admission without ICU	268	2.56 (2.27 - 2.89)	<0.001
		Admission with ICU	38	3.42 (2.49 - 4.71)	<0.001
	≥80	No admission	2,952	1.00 [reference]	··
		Admission without ICU	116	1.76 (1.46 - 2.12)	<0.001

Outcome	Age group	Admission	Cases, No.	HR (95% CI)[a]	p-value
		Admission with ICU	≤5[b]	..	..
Hematological disorders	<40	No admission	4,654	1.00 [reference]	··
		Admission without ICU	65	3.99 (3.12 - 5.10)	<0.001
		Admission with ICU	≤5[b]	..	..
	40-59	No admission	6,306	1.00 [reference]	··
		Admission without ICU	112	3.24 (2.68 - 3.91)	<0.001
		Admission with ICU	24	6.23 (4.17 - 9.31)	<0.001
	60-79	No admission	15,321	1.00 [reference]	··
		Admission without ICU	468	2.98 (2.72 - 3.27)	<0.001
		Admission with ICU	47	2.95 (2.21 - 3.92)	<0.001
	≥80	No admission	9,013	1.00 [reference]	··
		Admission without ICU	357	1.76 (1.58 - 1.96)	<0.001
		Admission with ICU	6	1.10 (0.49 - 2.45)	0.816
Musculoskeletal disorders	<40	No admission	10,707	1.00 [reference]	··
		Admission without ICU	44	1.21 (0.90 - 1.63)	0.204
		Admission with ICU	≤5[b]	..	..
	40-59	No admission	14,009	1.00 [reference]	··
		Admission without ICU	66	1.17 (0.92 - 1.50)	0.202
		Admission with ICU	≤5[b]	..	..
	60-79	No admission	8,049	1.00 [reference]	··
		Admission without ICU	71	1.08 (0.85 - 1.37)	0.517
		Admission with ICU	10	1.56 (0.84 - 2.89)	0.162
	≥80	No admission	1,058	1.00 [reference]	··
		Admission without ICU	20	0.80 (0.52 - 1.25)	0.333
		Admission with ICU	≤5[b]	..	..
Dermatological disorders	<60	No admission	14,749	1.00 [reference]	··
		Admission without ICU	89	2.08 (1.69 - 2.56)	<0.001
		Admission with ICU	14	4.99 (2.95 - 8.43)	<0.001
	≥60	No admission	3,507	1.00 [reference]	··
		Admission without ICU	67	1.70 (1.33 - 2.17)	<0.001
		Admission with ICU	7	2.45 (1.17 - 5.15)	0.018
Neuropsychiatric symptoms	<40	No admission	15,256	1.00 [reference]	··
		Admission without ICU	153	3.34 (2.85 - 3.92)	<0.001
		Admission with ICU	6	2.99 (1.34 - 6.66)	0.007
	40-59	No admission	16,959	1.00 [reference]	··
		Admission without ICU	253	3.42 (3.02 - 3.87)	<0.001
		Admission with ICU	32	4.03 (2.85 - 5.70)	<0.001
	60-79	No admission	27,104	1.00 [reference]	··
		Admission without ICU	661	2.74 (2.53 - 2.96)	<0.001
		Admission with ICU	72	2.80 (2.22 - 3.53)	<0.001
	≥80	No admission	10,727	1.00 [reference]	··
		Admission without ICU	389	1.67 (1.51 - 1.85)	<0.001
		Admission with ICU	13	2.07 (1.20 - 3.56)	0.009

Abbreviations: HR, Hazard Ratio; CI, Confidence Interval; ICU, Intensive Care Unit.

[a] The estimates are HRs with 95% CI from Cox Proportional Hazards model stratified by age and adjusted for confounders (sex, Charlson Comorbidity Index (CCI), parental CCI, parental mental health, employment status, income, highest level of education). All statistical tests were two-sided without correction for multiple comparisons.
[b] Results from ≤5 patients are displayed as "≤5" to ensure data privacy.
[c] The reference group *no COVID-19-admission* consisted of individuals without admission to a hospital with SARS-CoV-2 infection, i.e., all individuals with a negative or positive test result but no admission to a hospital.

Supplementary Table 14: Number of admissions.

Hazard ratios of specific first mental health or general medical disorders among individuals with 1 admission with COVID-19 and 2 or more admissions with COVID-19 compared to individuals without admission with COVID-19.

Outcome	Number of readmissions[b]	Cases, No.	HR (95% CI)[a]	p-value
Mental disorders or general medical conditions	No COVID-19-admission	264,814	1.00 [reference]	..
	1 admission	1,426	2.62 (2.48 - 2.76)	<0.001
	≥2 admissions	97	3.78 (3.10 - 4.62)	<0.001
Mental disorders	No COVID-19-admission	100,111	1.00 [reference]	..
	1 admission	748	1.92 (1.78 - 2.06)	<0.001
	≥2 admissions	71	2.20 (1.75 - 2.78)	<0.001
General medical conditions	No COVID-19-admission	241,417	1.00 [reference]	..
	1 admission	1,446	2.70 (2.57 - 2.85)	<0.001
	≥2 admissions	104	3.78 (3.12 - 4.58)	<0.001
Neurological disorders	No COVID-19-admission	181,879	1.00 [reference]	..
	1 admission	1,618	1.76 (1.68 - 1.85)	<0.001
	≥2 admissions	195	2.08 (1.81 - 2.39)	<0.001
Respiratory disorders	No COVID-19-admission	59,608	1.00 [reference]	..
	1 admission	1,322	3.34 (3.16 - 3.53)	<0.001
	≥2 admissions	177	4.39 (3.79 - 5.09)	<0.001
Circulatory disorders	No COVID-19-admission	119,189	1.00 [reference]	..
	1 admission	1,490	2.48 (2.36 - 2.61)	<0.001
	≥2 admissions	139	2.95 (2.50 - 3.49)	<0.001
Kidney disorders	No COVID-19-admission	22,579	1.00 [reference]	..
	1 admission	794	2.82 (2.62 - 3.03)	<0.001
	≥2 admissions	121	3.37 (2.82 - 4.03)	<0.001
Gastrointestinal disorders	No COVID-19-admission	60,312	1.00 [reference]	..
	1 admission	707	1.85 (1.72 - 1.99)	<0.001
	≥2 admissions	88	2.19 (1.78 - 2.70)	<0.001
Endocrine disorders	No COVID-19-admission	26,956	1.00 [reference]	..
	1 admission	559	2.65 (2.44 - 2.89)	<0.001
	≥2 admissions	59	2.53 (1.96 - 3.27)	<0.001
Hematological disorders	No COVID-19-admission	35,294	1.00 [reference]	..
	1 admission	933	2.59 (2.43 - 2.77)	<0.001
	≥2 admissions	149	3.49 (2.97 - 4.10)	<0.001
Musculoskeletal disorders	No COVID-19-admission	33,823	1.00 [reference]	..
	1 admission	185	1.01 (0.88 - 1.17)	0.863
	≥2 admissions	32	1.85 (1.31 - 2.61)	0.001
Dermatological disorders	No COVID-19-admission	18,256	1.00 [reference]	..
	1 admission	152	1.89 (1.61 - 2.22)	<0.001
	≥2 admissions	25	3.33 (2.25 - 4.93)	<0.001
Neuropsychiatric symptoms	No COVID-19-admission	70,046	1.00 [reference]	..
	1 admission	1,377	2.54 (2.41 - 2.68)	<0.001
	≥2 admissions	202	3.40 (2.96 - 3.91)	<0.001

Abbreviations: HR, Hazard Ratio; CI, Confidence Interval.

[a] The estimates are HRs with 95% CI from Cox Proportional Hazards model stratified by age and adjusted for confounders (sex, Charlson Comorbidity Index (CCI), parental CCI, parental mental health, employment status, income, highest level of education). All statistical tests were two-sided without correction for multiple comparisons.
[b] The reference group *no COVID-19-admission* consisted of individuals without admission to a hospital with SARS-CoV-2 infection, i.e., all individuals with negative or positive test results but no hospital admission.

Supplementary Table 15: Duration of admission.

Hazard ratios of specific first mental health or general medical disorders among individuals with admission with COVID-19 compared to individuals without admission with COVID-19 by duration of admission.

Outcome	Duration of admissions[b]	Cases, No.	HR (95% CI)[a]	p-value
	No COVID-19-admission	264,814	1.00 [reference]	..
Mental disorders or general medical conditions	1-2 bed days	598	2.72 (2.51 - 2.95)	<0.001
	3-6 bed days	518	2.31 (2.12 - 2.52)	<0.001
	≥7 bed days	407	3.06 (2.78 - 3.38)	<0.001
	No COVID-19-admission	100,111	1.00 [reference]	..
Mental disorders	1-2 bed days	314	2.07 (1.85 - 2.32)	<0.001
	3-6 bed days	250	1.57 (1.38 - 1.77)	<0.001
	≥7 bed days	255	2.30 (2.03 - 2.60)	<0.001
	No COVID-19-admission	241,417	1.00 [reference]	..
General medical conditions	1-2 bed days	571	2.68 (2.47 - 2.91)	<0.001
	3-6 bed days	519	2.35 (2.16 - 2.56)	<0.001
	≥7 bed days	460	3.10 (2.82 - 3.39)	<0.001
	No COVID-19-admission	181,879	1.00 [reference]	..
Neurological disorders	1-2 bed days	569	1.85 (1.70 - 2.01)	<0.001
	3-6 bed days	614	1.58 (1.46 - 1.71)	<0.001
	≥7 bed days	630	1.79 (1.65 - 1.93)	<0.001
	No COVID-19-admission	59,608	1.00 [reference]	..
Respiratory disorders	1-2 bed days	424	3.32 (3.01 - 3.65)	<0.001
	3-6 bed days	474	2.92 (2.67 - 3.20)	<0.001
	≥7 bed days	601	3.90 (3.60 - 4.23)	<0.001
	No COVID-19-admission	119,189	1.00 [reference]	..
Circulatory disorders	1-2 bed days	482	2.36 (2.16 - 2.59)	<0.001
	3-6 bed days	551	2.10 (1.93 - 2.29)	<0.001
	≥7 bed days	596	2.69 (2.48 - 2.92)	<0.001
	No COVID-19-admission	22,579	1.00 [reference]	..
Kidney disorders	1-2 bed days	237	2.91 (2.56 - 3.31)	<0.001
	3-6 bed days	273	2.29 (2.03 - 2.58)	<0.001
	≥7 bed days	405	3.45 (3.13 - 3.81)	<0.001
	No COVID-19-admission	60,312	1.00 [reference]	..
Gastrointestinal disorders	1-2 bed days	229	1.90 (1.67 - 2.16)	<0.001
	3-6 bed days	262	1.69 (1.49 - 1.91)	<0.001
	≥7 bed days	304	2.03 (1.81 - 2.28)	<0.001
	No COVID-19-admission	26,956	1.00 [reference]	..
Endocrine disorders	1-2 bed days	183	2.78 (2.40 - 3.21)	<0.001
	3-6 bed days	205	2.41 (2.10 - 2.77)	<0.001
	≥7 bed days	230	2.76 (2.43 - 3.15)	<0.001
	No COVID-19-admission	35,294	1.00 [reference]	..
Hematological disorders	1-2 bed days	263	2.46 (2.18 - 2.78)	<0.001
	3-6 bed days	357	2.40 (2.16 - 2.66)	<0.001
	≥7 bed days	462	3.13 (2.86 - 3.44)	<0.001
	No COVID-19-admission	33,823	1.00 [reference]	..
Musculoskeletal disorders	1-2 bed days	71	1.10 (0.87 - 1.39)	0.429
	3-6 bed days	74	1.00 (0.79 - 1.25)	0.972
	≥7 bed days	72	1.18 (0.94 - 1.49)	0.163
	No COVID-19-admission	18,256	1.00 [reference]	..
Dermatological disorders	1-2 bed days	59	1.98 (1.53 - 2.55)	<0.001
	3-6 bed days	53	1.63 (1.24 - 2.13)	<0.001
	≥7 bed days	65	2.55 (1.99 - 3.25)	<0.001
	No COVID-19-admission	70,046	1.00 [reference]	..
Neuropsychiatric symptoms	1-2 bed days	459	2.71 (2.47 - 2.97)	<0.001
	3-6 bed days	539	2.28 (2.09 - 2.48)	<0.001
	≥7 bed days	581	2.54 (2.34 - 2.76)	<0.001

Abbreviations: HR, Hazard Ratio; CI, Confidence Interval.

[a] The estimates are HRs with 95% CI from Cox Proportional Hazards model stratified by age and adjusted for confounders (sex, Charlson Comorbidity Index (CCI), parental CCI, parental mental health, employment status, income, highest level of education). All statistical tests were two-sided without correction for multiple comparisons.
[b] The reference group *no COVID-19-admission* consisted of individuals without admission to a hospital with SARS-CoV-2 infection, i.e., all individuals with negative or positive test results but no hospital admission.

Supplementary Table 16: Calendar period.

Hazard ratios of specific first mental health or general medical disorders at different calendar times. Admissions with COVID-19 were compared to no admission with COVID-19 within each calendar period.

Outcome	Calendar period	Cases, No. No COVID-19 admission	Cases, No. COVID-19 admission	HR (95% CI)[a]	p-value
Mental disorders or general medical conditions	2020-03-01	586	29	1.99 (1.35 – 2.92)	<0.001
	2020-05-01	4,155	24	1.93 (1.29 – 2.89)	0.001
	2020-07-01	5,484	15	2.27 (1.37 – 3.77)	0.002
	2020-09-01	10,283	16	2.41 (1.48 – 3.94)	<0.001
	2020-11-01	12,750	38	4.40 (3.20 – 6.06)	<0.001
	2021-01-01	13,788	78	5.39 (4.31 – 6.73)	<0.001
	2021-03-01	15,740	56	3.27 (2.52 – 4.26)	<0.001
	2021-05-01	17,159	61	3.17 (2.47 – 4.08)	<0.001
	2021-07-01	12,767	27	1.79 (1.23 – 2.61)	0.003
	2021-09-01	15,904	45	2.26 (1.69 – 3.03)	<0.001
	2021-11-01	15,648	83	3.80 (3.07 – 4.72)	<0.001
	2022-01-01	15,519	161	5.21 (4.46 – 6.09)	<0.001
	2022-03-01	16,060	154	3.50 (2.98 – 4.10)	<0.001
	2022-05-01	17,084	116	2.34 (1.95 – 2.81)	<0.001
	2022-07-01	13,525	92	2.22 (1.81 – 2.72)	<0.001
	2022-09-01	15,861	112	2.26 (1.88 – 2.73)	<0.001
	2022-11-01	15,797	101	2.02 (1.66 – 2.46)	<0.001
	2023-01-01	16,182	91	1.76 (1.43 – 2.16)	<0.001
	2023-03-01	15,531	100	2.02 (1.66 – 2.46)	<0.001
	2023-05-01	15,023	92	1.92 (1.56 – 2.36)	<0.001
	p-value of effect modifier	..	..	..	<0.001
Mental disorder	2020-03-01	193	≤5[b]	..	..
	2020-05-01	1,336	≤5[b]	..	..
	2020-07-01	2,198	7	1.75 (0.83 - 3.67)	0.142
	2020-09-01	3,845	7	1.78 (0.85 - 3.73)	0.129
	2020-11-01	5,016	8	1.45 (0.73 - 2.91)	0.291
	2021-01-01	5,643	27	2.75 (1.88 - 4.03)	<0.001
	2021-03-01	6,010	27	2.29 (1.57 - 3.35)	<0.001
	2021-05-01	6,615	24	1.77 (1.18 - 2.64)	0.005
	2021-07-01	5,290	18	1.49 (0.94 - 2.38)	0.089
	2021-09-01	6,264	25	1.67 (1.13 - 2.48)	0.010
	2021-11-01	6,090	41	2.55 (1.88 - 3.47)	<0.001
	2022-01-01	6,046	68	2.94 (2.31 - 3.74)	<0.001
	2022-03-01	5,992	89	2.60 (2.11 - 3.21)	<0.001
	2022-05-01	6,449	70	1.84 (1.45 - 2.33)	<0.001
	2022-07-01	5,152	69	2.06 (1.63 - 2.62)	<0.001
	2022-09-01	5,763	74	1.92 (1.52 - 2.42)	<0.001
	2022-11-01	5,621	57	1.49 (1.14 - 1.93)	0.003
	2023-01-01	5,741	78	1.98 (1.58 - 2.47)	<0.001
	2023-03-01	5,485	74	1.98 (1.57 - 2.49)	<0.001
	2023-05-01	5,362	50	1.35 (1.02 - 1.78)	0.037
	p-value of effect modifier	..	..	..	<0.001
General medical conditions	2020-03-01	617	30	1.88 (1.29 - 2.73)	0.001
	2020-05-01	4,144	26	1.98 (1.34 - 2.91)	0.001
	2020-07-01	5,191	15	2.20 (1.33 - 3.66)	0.002
	2020-09-01	9,553	17	2.45 (1.52 - 3.95)	<0.001
	2020-11-01	11,526	44	4.91 (3.65 - 6.60)	<0.001
	2021-01-01	12,245	70	4.73 (3.73 - 5.98)	<0.001
	2021-03-01	14,215	56	3.21 (2.46 - 4.17)	<0.001
	2021-05-01	15,318	62	3.18 (2.48 - 4.09)	<0.001
	2021-07-01	11,451	34	2.24 (1.60 - 3.14)	<0.001
	2021-09-01	14,246	49	2.48 (1.87 - 3.28)	<0.001
	2021-11-01	13,939	82	3.78 (3.04 - 4.70)	<0.001
	2022-01-01	13,956	167	5.38 (4.62 - 6.27)	<0.001
	2022-03-01	14,606	154	3.44 (2.94 - 4.04)	<0.001
	2022-05-01	15,418	128	2.57 (2.16 - 3.06)	<0.001
	2022-07-01	12,325	94	2.26 (1.84 - 2.76)	<0.001
	2022-09-01	14,739	112	2.20 (1.83 - 2.65)	<0.001
	2022-11-01	14,571	116	2.27 (1.89 - 2.73)	<0.001
	2023-01-01	15,027	105	1.97 (1.63 - 2.39)	<0.001
	2023-03-01	14,386	101	1.99 (1.63 - 2.42)	<0.001
	2023-05-01	13,941	91	1.84 (1.49 - 2.26)	<0.001
	p-value of effect modifier	..	..	..	<0.001
	2020-03-01	246	16	2.38 (1.42 - 4.01)	0.001
	2020-05-01	2,489	19	1.54 (0.98 - 2.43)	0.060
	2020-07-01	3,430	15	1.82 (1.10 - 3.03)	0.021
	2020-09-01	6,670	16	1.51 (0.92 - 2.46)	0.102
	2020-11-01	8,227	35	2.42 (1.74 - 3.38)	<0.001
	2021-01-01	9,249	54	1.95 (1.49 - 2.55)	<0.001
	2021-03-01	10,462	61	1.91 (1.48 - 2.46)	<0.001

	2021-05-01	11,030	61	1.79 (1.39 - 2.30)	<0.001
	2021-07-01	7,531	49	2.14 (1.61 - 2.83)	<0.001
Neurological disorders	2021-09-01	10,402	75	2.24 (1.78 - 2.81)	<0.001
	2021-11-01	10,159	61	1.65 (1.28 - 2.12)	<0.001
	2022-01-01	10,431	143	2.72 (2.30 - 3.21)	<0.001
	2022-03-01	11,265	141	1.72 (1.46 - 2.03)	<0.001
	2022-05-01	12,215	147	1.60 (1.36 - 1.88)	<0.001
	2022-07-01	9,408	136	1.85 (1.56 - 2.19)	<0.001
	2022-09-01	11,859	156	1.60 (1.37 - 1.87)	<0.001
	2022-11-01	11,535	152	1.53 (1.31 - 1.80)	<0.001
	2023-01-01	12,360	153	1.40 (1.19 - 1.64)	<0.001
	2023-03-01	11,551	147	1.43 (1.21 - 1.68)	<0.001
	2023-05-01	11,358	178	1.74 (1.50 - 2.02)	<0.001
	p-value of effect modifier	..	..	..	<0.001
	2020-03-01	436	49	2.70 (1.99 - 3.65)	<0.001
	2020-05-01	1,958	19	1.57 (1.00 - 2.47)	0.051
	2020-07-01	2,077	18	2.94 (1.85 - 4.68)	<0.001
	2020-09-01	3,216	23	3.89 (2.58 - 5.87)	<0.001
	2020-11-01	3,304	62	8.32 (6.46 - 10.71)	<0.001
	2021-01-01	3,007	84	6.67 (5.36 - 8.30)	<0.001
	2021-03-01	3,462	62	4.33 (3.36 - 5.57)	<0.001
	2021-05-01	3,655	64	4.42 (3.45 - 5.67)	<0.001
	2021-07-01	2,534	33	3.15 (2.23 - 4.45)	<0.001
Respiratory disorders	2021-09-01	3,559	51	3.51 (2.66 - 4.63)	<0.001
	2021-11-01	3,584	63	3.85 (3.00 - 4.94)	<0.001
	2022-01-01	3,059	112	5.31 (4.39 - 6.42)	<0.001
	2022-03-01	3,300	151	4.54 (3.85 - 5.35)	<0.001
	2022-05-01	3,400	98	2.90 (2.37 - 3.55)	<0.001
	2022-07-01	2,560	87	3.07 (2.48 - 3.81)	<0.001
	2022-09-01	3,289	101	2.77 (2.27 - 3.38)	<0.001
	2022-11-01	3,526	126	3.11 (2.60 - 3.73)	<0.001
	2023-01-01	3,444	116	2.73 (2.26 - 3.29)	<0.001
	2023-03-01	3,134	100	2.56 (2.09 - 3.13)	<0.001
	2023-05-01	3,103	81	2.09 (1.67 - 2.61)	<0.001
	p-value of effect modifier	..	..	..	<0.001
	2020-03-01	553	33	1.44 (1.01 - 2.06)	0.046
	2020-05-01	2,538	29	1.85 (1.28 - 2.67)	0.001
	2020-07-01	3,061	14	1.71 (1.01 - 2.89)	0.046
	2020-09-01	4,709	21	2.65 (1.72 - 4.06)	<0.001
	2020-11-01	5,812	50	4.35 (3.29 - 5.74)	<0.001
	2021-01-01	6,477	111	5.23 (4.33 - 6.32)	<0.001
	2021-03-01	7,234	70	3.03 (2.40 - 3.84)	<0.001
	2021-05-01	7,203	54	2.33 (1.78 - 3.05)	<0.001
Circulatory disorders	2021-07-01	5,846	37	2.01 (1.45 - 2.77)	<0.001
	2021-09-01	6,766	41	1.84 (1.35 - 2.50)	<0.001
	2021-11-01	6,865	78	3.05 (2.44 - 3.82)	<0.001
	2022-01-01	6,991	159	4.44 (3.79 - 5.20)	<0.001
	2022-03-01	6,944	196	3.93 (3.41 - 4.53)	<0.001
	2022-05-01	7,242	123	2.28 (1.91 - 2.73)	<0.001
	2022-07-01	6,172	94	1.95 (1.59 - 2.39)	<0.001
	2022-09-01	6,946	119	2.12 (1.77 - 2.54)	<0.001
	2022-11-01	7,065	102	1.67 (1.37 - 2.03)	<0.001
	2023-01-01	7,362	108	1.67 (1.38 - 2.02)	<0.001
	2023-03-01	6,804	109	1.82 (1.50 - 2.20)	<0.001
	2023-05-01	6,596	84	1.40 (1.13 - 1.74)	0.002
	p-value of effect modifier	..	..	..	<0.001
	2020-03-01	162	38	4.94 (3.44 - 7.10)	<0.001
	2020-05-01	618	7	1.15 (0.54 - 2.42)	0.717
	2020-07-01	809	≤5[b]	..	..
	2020-09-01	915	13	3.92 (2.26 - 6.78)	<0.001
	2020-11-01	1,095	26	5.15 (3.49 - 7.61)	<0.001
	2021-01-01	1,128	43	4.50 (3.31 - 6.11)	<0.001
	2021-03-01	1,239	28	2.78 (1.91 - 4.05)	<0.001
	2021-05-01	1,251	21	2.15 (1.39 - 3.31)	0.001
	2021-07-01	1,100	22	2.55 (1.67 - 3.89)	<0.001
Kidney disorders	2021-09-01	1,191	31	3.23 (2.26 - 4.62)	<0.001
	2021-11-01	1,251	48	4.05 (3.03 - 5.41)	<0.001
	2022-01-01	1,269	74	4.55 (3.59 - 5.76)	<0.001
	2022-03-01	1,316	103	4.02 (3.29 - 4.92)	<0.001
	2022-05-01	1,433	61	2.14 (1.65 - 2.77)	<0.001
	2022-07-01	1,221	67	2.51 (1.96 - 3.22)	<0.001
	2022-09-01	1,361	58	1.89 (1.45 - 2.46)	<0.001
	2022-11-01	1,288	74	2.30 (1.82 - 2.92)	<0.001
	2023-01-01	1,275	70	2.16 (1.70 - 2.76)	<0.001
	2023-03-01	1,327	65	1.92 (1.49 - 2.46)	<0.001
	2023-05-01	1,327	64	1.82 (1.42 - 2.35)	<0.001
	p-value of effect modifier	..	..	..	<0.001
	2020-03-01	231	≤5[b]	..	..

Gastrointestinal disorders	2020-05-01	1,855	≤5[b]	..	..
	2020-07-01	2,104	≤5[b]	..	..
	2020-09-01	3,080	≤5[b]	..	..
	2020-11-01	3,437	11	1.45 (0.80 - 2.62)	0.220
	2021-01-01	3,358	26	2.03 (1.38 - 2.99)	<0.001
	2021-03-01	3,854	33	2.11 (1.49 - 2.99)	<0.001
	2021-05-01	3,864	26	1.73 (1.18 - 2.55)	0.005
	2021-07-01	2,720	17	1.51 (0.94 - 2.43)	0.092
	2021-09-01	3,444	28	2.02 (1.39 - 2.93)	<0.001
	2021-11-01	3,437	38	2.38 (1.72 - 3.27)	<0.001
	2022-01-01	3,409	66	3.02 (2.36 - 3.85)	<0.001
	2022-03-01	3,399	88	2.83 (2.29 - 3.51)	<0.001
	2022-05-01	3,414	63	1.87 (1.45 - 2.40)	<0.001
	2022-07-01	2,688	56	1.89 (1.45 - 2.47)	<0.001
	2022-09-01	3,373	65	1.77 (1.38 - 2.27)	<0.001
	2022-11-01	3,243	60	1.63 (1.26 - 2.11)	<0.001
	2023-01-01	3,283	63	1.66 (1.29 - 2.14)	<0.001
	2023-03-01	3,139	79	2.13 (1.70 - 2.67)	<0.001
	2023-05-01	2,980	61	1.66 (1.28 - 2.15)	<0.001
	p-value of effect modifier	..	..	..	<0.001
Endocrine disorders	2020-03-01	166	13	1.98 (1.12 - 3.51)	0.019
	2020-05-01	622	8	1.76 (0.87 - 3.54)	0.113
	2020-07-01	748	≤5[b]	..	..
	2020-09-01	1,165	6	2.11 (0.95 - 4.72)	0.068
	2020-11-01	1,331	29	8.02 (5.54 - 11.61)	<0.001
	2021-01-01	1,486	37	5.26 (3.78 - 7.30)	<0.001
	2021-03-01	1,668	24	3.09 (2.06 - 4.62)	<0.001
	2021-05-01	1,652	17	2.20 (1.36 - 3.55)	0.001
	2021-07-01	1,247	9	1.56 (0.81 - 3.02)	0.182
	2021-09-01	1,509	28	3.90 (2.68 - 5.68)	<0.001
	2021-11-01	1,522	35	4.21 (3.01 - 5.89)	<0.001
	2022-01-01	1,604	57	4.80 (3.68 - 6.26)	<0.001
	2022-03-01	1,582	53	3.01 (2.28 - 3.96)	<0.001
	2022-05-01	1,573	44	2.36 (1.74 - 3.20)	<0.001
	2022-07-01	1,307	37	2.31 (1.67 - 3.22)	<0.001
	2022-09-01	1,597	46	2.27 (1.69 - 3.05)	<0.001
	2022-11-01	1,518	50	2.36 (1.77 - 3.13)	<0.001
	2023-01-01	1,665	37	1.61 (1.16 - 2.24)	0.004
	2023-03-01	1,549	45	2.07 (1.54 - 2.79)	<0.001
	2023-05-01	1,443	42	1.95 (1.43 - 2.65)	<0.001
	p-value of effect modifier	..	..	..	<0.001
Hematological disorders	2020-03-01	187	≤5[b]	..	..
	2020-05-01	1,065	12	1.19 (0.67 - 2.10)	0.556
	2020-07-01	1,343	9	1.49 (0.77 - 2.86)	0.236
	2020-09-01	1,684	9	1.69 (0.88 - 3.26)	0.116
	2020-11-01	1,728	14	2.04 (1.20 - 3.45)	0.008
	2021-01-01	1,712	45	3.67 (2.72 - 4.94)	<0.001
	2021-03-01	1,847	28	2.21 (1.52 - 3.22)	<0.001
	2021-05-01	2,200	20	1.38 (0.89 - 2.14)	0.153
	2021-07-01	1,661	20	1.84 (1.18 - 2.86)	0.007
	2021-09-01	1,994	23	1.74 (1.15 - 2.63)	0.008
	2021-11-01	1,849	23	1.63 (1.08 - 2.46)	0.020
	2022-01-01	1,815	73	3.72 (2.94 - 4.71)	<0.001
	2022-03-01	2,030	118	3.67 (3.04 - 4.43)	<0.001
	2022-05-01	2,258	94	2.57 (2.09 - 3.17)	<0.001
	2022-07-01	1,908	110	3.21 (2.65 - 3.90)	<0.001
	2022-09-01	1,999	109	2.89 (2.38 - 3.51)	<0.001
	2022-11-01	1,960	102	2.61 (2.14 - 3.20)	<0.001
	2023-01-01	2,023	101	2.53 (2.06 - 3.09)	<0.001
	2023-03-01	1,846	72	1.92 (1.52 - 2.44)	<0.001
	2023-05-01	2,184	96	2.14 (1.74 - 2.63)	<0.001
	p-value of effect modifier	..	..	..	<0.001
Musculoskeletal disorders	censored[c]	..	..	..	..
Dermatological disorders	censored[c]	..	..	..	..
Neuropsychiatric symptoms	2020-03-01	212	6	0.85 (0.37 - 1.92)	0.689
	2020-05-01	1,321	8	0.84 (0.42 - 1.68)	0.618
	2020-07-01	2,021	27	3.83 (2.62 - 5.61)	<0.001
	2020-09-01	2,892	11	1.69 (0.93 - 3.05)	0.084
	2020-11-01	3,385	25	2.64 (1.78 - 3.91)	<0.001
	2021-01-01	3,269	39	2.47 (1.80 - 3.38)	<0.001
	2021-03-01	4,054	44	2.26 (1.68 - 3.05)	<0.001
	2021-05-01	4,673	43	1.95 (1.44 - 2.64)	<0.001
	2021-07-01	4,252	30	1.49 (1.04 - 2.14)	0.029
	2021-09-01	4,178	37	1.82 (1.32 - 2.52)	<0.001
	2021-11-01	3,936	50	2.32 (1.76 - 3.07)	<0.001
	2022-01-01	3,809	98	3.33 (2.72 - 4.07)	<0.001
	2022-03-01	3,894	148	3.28 (2.78 - 3.87)	<0.001
	2022-05-01	4,279	159	3.06 (2.61 - 3.59)	<0.001

2022-07-01	3,749	147	2.93 (2.48 - 3.46)	<0.001
2022-09-01	4,223	130	2.22 (1.86 - 2.65)	<0.001
2022-11-01	3,835	158	2.79 (2.37 - 3.27)	<0.001
2023-01-01	3,984	150	2.45 (2.08 - 2.89)	<0.001
2023-03-01	4,126	127	2.07 (1.73 - 2.47)	<0.001
2023-05-01	3,954	142	2.32 (1.96 - 2.75)	<0.001
p-value of effect modifier	..	..	..	<0.001

Abbreviations: HR, Hazard Ratio; CI, Confidence Interval.

[a] The estimates are HRs with 95% CI from Cox Proportional Hazards model stratified by age and adjusted for confounders (sex, Charlson Comorbidity Index (CCI), parental CCI, parental mental health, employment status, income, and highest level of education). All statistical tests were two-sided without correction for multiple comparisons.

[b] Results from ≤5 patients are displayed as "≤5" to ensure data privacy.

[c] Results are censored to ensure data privacy.

Results: Admission with COVID-19 compared with other infections
Supplementary Table 17: Admission with COVID-19 compared with admissions with non-COVID-19 infections.

Hazard ratios of specific first mental health or general medical disorders among individuals admitted with COVID-19 compared to admissions with non-COVID-19 infections.

Outcome	Infection type[b]	Cases, No.	HR (95% CI)[a]	p-value
Mental disorders or general medical conditions	No admission	229,424	1.00 [reference]	··
	Any non-COVID infection	5,836	2.29 (2.23 - 2.35)	<0.001
	COVID-19-hospitalization	1,294	2.71 (2.57 - 2.86)	<0.001
	COVID-19 vs. other infection	1,294	1.19 (1.12 - 1.26)	<0.001
Mental disorders	No admission	80,928	1.00 [reference]	··
	Any non-COVID infection	2,779	2.03 (1.95 - 2.11)	<0.001
	COVID-19-hospitalization	567	2.09 (1.93 - 2.27)	<0.001
	COVID-19 vs. other infection	567	1.03 (0.94 - 1.13)	0.517
General medical conditions	No admission	207,045	1.00 [reference]	··
	Any non-COVID infection	5,980	2.41 (2.35 - 2.48)	<0.001
	COVID-19-hospitalization	1,331	2.79 (2.64 - 2.94)	<0.001
	COVID-19 vs. other infection	1,331	1.15 (1.09 - 1.23)	<0.001
Neurological disorders	No admission	144,837	1.00 [reference]	··
	Any non-COVID infection	5,626	1.52 (1.48 - 1.56)	<0.001
	COVID-19-hospitalization	1,271	1.81 (1.71 - 1.91)	<0.001
	COVID-19 vs. other infection	1,271	1.19 (1.12 - 1.26)	<0.001
Respiratory disorders	No admission	40,994	1.00 [reference]	··
	Any non-COVID infection	4,503	3.57 (3.46 - 3.69)	<0.001
	COVID-19-hospitalization	1,003	4.32 (4.05 - 4.60)	<0.001
	COVID-19 vs. other infection	1,003	1.21 (1.13 - 1.29)	<0.001
Circulatory disorders	No admission	96,449	1.00 [reference]	··
	Any non-COVID infection	5,525	2.26 (2.20 - 2.33)	<0.001
	COVID-19-hospitalization	1,246	2.66 (2.51 - 2.81)	<0.001
	COVID-19 vs. other infection	1,246	1.17 (1.10 - 1.25)	<0.001
Kidney disorders	No admission	11,690	1.00 [reference]	··
	Any non-COVID infection	3,043	4.27 (4.09 - 4.45)	<0.001
	COVID-19-hospitalization	499	3.79 (3.46 - 4.15)	<0.001
	COVID-19 vs. other infection	499	0.89 (0.81 - 0.98)	0.015
Gastrointestinal disorders	No admission	45,649	1.00 [reference]	··
	Any non-COVID infection	2,885	2.29 (2.21 - 2.38)	<0.001
	COVID-19-hospitalization	529	2.13 (1.95 - 2.32)	<0.001
	COVID-19 vs. other infection	529	0.93 (0.85 - 1.02)	0.119
Endocrine disorders	No admission	19,219	1.00 [reference]	··
	Any non-COVID infection	1,854	2.73 (2.60 - 2.87)	<0.001
	COVID-19-hospitalization	400	3.09 (2.80 - 3.42)	<0.001
	COVID-19 vs. other infection	400	1.13 (1.02 - 1.26)	0.025
Hematological disorders	No admission	21,495	1.00 [reference]	··
	Any non-COVID infection	3,710	3.56 (3.43 - 3.69)	<0.001
	COVID-19-hospitalization	647	3.35 (3.10 - 3.63)	<0.001
	COVID-19 vs. other infection	647	0.94 (0.87 - 1.02)	0.155
Musculoskeletal disorders	No admission	26,850	1.00 [reference]	··
	Any non-COVID infection	869	1.49 (1.39 - 1.59)	<0.001
	COVID-19-hospitalization	134	1.07 (0.91 - 1.27)	0.411
	COVID-19 vs. other infection	134	0.72 (0.60 - 0.87)	<0.001
Dermatological disorders	No admission	13,616	1.00 [reference]	··
	Any non-COVID infection	724	2.85 (2.64 - 3.08)	<0.001
	COVID-19-hospitalization	109	2.16 (1.79 - 2.62)	<0.001
	COVID-19 vs. other infection	109	0.76 (0.62 - 0.93)	0.007
Neuropsychiatric symptoms	No admission	48,524	1.00 [reference]	··
	Any non-COVID infection	4,647	2.63 (2.55 - 2.71)	<0.001
	COVID-19-hospitalization	955	2.92 (2.74 - 3.12)	<0.001
	COVID-19 vs. other infection	955	1.11 (1.04 - 1.19)	0.003

Abbreviations: HR, Hazard Ratio; CI, Confidence Interval.

[a] The estimates are HRs with 95% CI from Cox Proportional Hazards model stratified by age and adjusted for confounders (sex, employment status, income, the highest level of education, Charlson Comorbidity Index, parental Charlson Comorbidity Index, and parental history of mental disorders). All statistical tests were two-sided without correction for multiple comparisons.

[b] Excluding individuals with any infection in a hospital within the past 10 years to rule out recurring infections. The reference group *no admission* consisted of all individuals without admission to a hospital with SARS-CoV-2 infection or any infection.

Supplementary Table 18: Admission with COVID-19 compared with admission with non-COVID-19 pulmonary infection.

Hazard ratios of specific first mental health or general medical disorders among individuals admitted with COVID-19 compared to admissions with non-COVID-19 pulmonary infections.

Outcome	Infection type[b]	Cases, No.	HR (95% CI)[a]	p-value
Mental disorders or general medical conditions	No admission	254,868	1.00 [reference]	··
	Any non-COVID pulmonary infection	1,780	3.61 (3.45 - 3.78)	<0.001
	Influenza	64	3.27 (2.56 - 4.18)	<0.001
	Bacterial pneumonia	1,199	3.80 (3.59 - 4.02)	<0.001
	Other	517	3.46 (3.17 - 3.77)	<0.001
	COVID-19-hospitalization	1,428	2.60 (2.47 - 2.74)	<0.001
	COVID-19 vs. other pulmonary infection	1,428	0.72 (0.67 - 0.77)	<0.001
Mental disorders	No admission	94,197	1.00 [reference]	··
	Any non-COVID pulmonary infection	913	2.32 (2.17 - 2.48)	<0.001
	COVID-19-hospitalization	722	2.02 (1.87 - 2.17)	<0.001
	COVID-19 vs. other pulmonary infection	722	0.87 (0.79 - 0.96)	0.005
General medical conditions	No admission	231,699	1.00 [reference]	··
	Any non-COVID pulmonary infection	1,919	3.84 (3.67 - 4.02)	<0.001
	COVID-19-hospitalization	1,479	2.68 (2.55 - 2.83)	<0.001
	COVID-19 vs. other pulmonary infection	1,479	0.70 (0.65 - 0.75)	<0.001
Neurological disorders	No admission	169,237	1.00 [reference]	··
	Any non-COVID pulmonary infection	2,052	1.64 (1.57 - 1.72)	<0.001
	COVID-19-hospitalization	1,585	1.78 (1.69 - 1.87)	<0.001
	COVID-19 vs. other pulmonary infection	1,585	1.08 (1.01 - 1.16)	0.018
Respiratory disorders	No admission	50,361	1.00 [reference]	··
	Any non-COVID pulmonary infection	3,262	7.18 (6.92 - 7.45)	<0.001
	COVID-19-hospitalization	1,267	3.99 (3.77 - 4.22)	<0.001
	COVID-19 vs. other pulmonary infection	1,267	0.56 (0.52 - 0.59)	<0.001
Circulatory disorders	No admission	111,614	1.00 [reference]	··
	Any non-COVID pulmonary infection	2,234	2.74 (2.62 - 2.85)	<0.001
	COVID-19-hospitalization	1,472	2.54 (2.41 - 2.67)	<0.001
	COVID-19 vs. other pulmonary infection	1,472	0.93 (0.87 - 0.99)	0.025
Kidney disorders	No admission	17,829	1.00 [reference]	··
	Any non-COVID pulmonary infection	1,365	3.17 (2.99 - 3.35)	<0.001
	COVID-19-hospitalization	704	3.05 (2.82 - 3.29)	<0.001
	COVID-19 vs. other pulmonary infection	704	0.96 (0.88 - 1.05)	0.415
Gastrointestinal disorders	No admission	55,521	1.00 [reference]	··
	Any non-COVID pulmonary infection	1,025	2.04 (1.92 - 2.18)	<0.001
	COVID-19-hospitalization	691	2.02 (1.87 - 2.18)	<0.001
	COVID-19 vs. other pulmonary infection	691	0.99 (0.90 - 1.09)	0.790
Endocrine disorders	No admission	24,204	1.00 [reference]	··
	Any non-COVID pulmonary infection	713	2.34 (2.17 - 2.53)	<0.001
	COVID-19-hospitalization	509	2.78 (2.54 - 3.04)	<0.001
	COVID-19 vs. other pulmonary infection	509	1.19 (1.06 - 1.33)	0.003
Hematological disorders	No admission	29,672	1.00 [reference]	··
	Any non-COVID pulmonary infection	1,669	3.11 (2.96 - 3.28)	<0.001
	COVID-19-hospitalization	861	2.78 (2.60 - 2.98)	<0.001
	COVID-19 vs. other pulmonary infection	861	0.89 (0.82 - 0.97)	0.007
Musculoskeletal disorders	No admission	31,775	1.00 [reference]	··
	Any non-COVID pulmonary infection	261	1.38 (1.22 - 1.56)	<0.001
	COVID-19-hospitalization	183	1.11 (0.96 - 1.28)	0.174
	COVID-19 vs. other pulmonary infection	183	0.80 (0.67 - 0.97)	0.024
Dermatological disorders	No admission	16,638	1.00 [reference]	··
	Any non-COVID pulmonary infection	258	2.94 (2.60 - 3.34)	<0.001
	COVID-19-hospitalization	146	2.06 (1.75 - 2.42)	<0.001
	COVID-19 vs. other pulmonary infection	146	0.70 (0.57 - 0.86)	0.001
Neuropsychiatric symptoms	No admission	61,776	1.00 [reference]	··
	Any non-COVID pulmonary infection	2,141	2.76 (2.64 - 2.89)	<0.001
	COVID-19-hospitalization	1,264	2.66 (2.52 - 2.82)	<0.001
	COVID-19 vs. other pulmonary infection	1,264	0.96 (0.90 - 1.03)	0.315

Abbreviations: HR, Hazard Ratio; CI, Confidence Interval.

[a] The estimates are HRs with 95% CI from Cox Proportional Hazards model stratified by age and adjusted for confounders (sex, employment status, income, the highest level of education, Charlson Comorbidity Index, parental Charlson Comorbidity Index, and parental history of mental disorders). All statistical tests were two-sided without correction for multiple comparisons.
[b] Excluding individuals with any pulmonary infection in a hospital within the past 10 years to rule out recurring pulmonary infections. The reference group *no admission* consisted of all individuals without admission to a hospital with SARS-CoV-2 infection or any pulmonary infection.

Results: Positive SARS-CoV-2 test by peak C-Reactive Protein (CRP)
Supplementary Table 19: Positive or negative test and CRP value.

Hazard ratios of specific first mental health or general medical disorders among individuals with positive SARS-CoV-2 test compared to negative SARS-CoV-2 tests by peak CRP value.

Outcome	Peak CRP value[b]	Cases, No.	HR (95% CI)[a]	p-value
	SARS-CoV-2 negative	39,546		
	<4 mg/L	15,183	1.00 [reference]	..
	4-10 mg/L	10,324	1.06 (1.03 – 1.09)	<0.001
	10-40 mg/L	5,567	1.22 (1.18 – 1.26)	<0.001
	40-100 mg/L	3,669	1.22 (1.17 – 1.26)	<0.001
Mental disorders or	≥100 mg/L	4,803	1.35 (1.31 – 1.40)	<0.001
general medical conditions	SARS-CoV-2 positive	16,713		
	<4 mg/L	7,173	0.96 (0.93 – 0.99)	0.015
	4-10 mg/L	4,520	1.02 (0.98 – 1.05)	0.370
	10-40 mg/L	2,265	1.16 (1.11 – 1.22)	<0.001
	40-100 mg/L	1,210	1.03 (0.97 – 1.09)	0.334
	≥100 mg/L	1,545	1.35 (1.28 – 1.43)	<0.001
	SARS-CoV-2 negative	19,962		
	<4 mg/L	8,083	1.00 [reference]	..
	4-10 mg/L	5,267	0.95 (0.92 – 0.99)	0.005
	10-40 mg/L	3,073	1.06 (1.02 – 1.11)	0.004
	40-100 mg/L	1,796	1.11 (1.05 – 1.17)	<0.001
Mental disorders	≥100 mg/L	1,743	1.06 (1.01 – 1.12)	0.028
	SARS-CoV-2 positive	7,786		
	<4 mg/L	3,638	0.83 (0.79 – 0.86)	<0.001
	4-10 mg/L	2,101	0.77 (0.73 – 0.81)	<0.001
	10-40 mg/L	1,038	0.79 (0.74 – 0.85)	<0.001
	40-100 mg/L	523	0.79 (0.72 – 0.86)	<0.001
	≥100 mg/L	486	0.83 (0.76 – 0.92)	<0.001
	SARS-CoV-2 negative	40,802		
	<4 mg/L	16,094	1.00 [reference]	..
	4-10 mg/L	10,929	1.08 (1.05 – 1.10)	<0.001
	10-40 mg/L	5,881	1.30 (1.27 – 1.34)	<0.001
	40-100 mg/L	3,632	1.31 (1.26 – 1.36)	<0.001
General medical conditions	≥100 mg/L	4,266	1.41 (1.36 – 1.46)	<0.001
	SARS-CoV-2 positive	16,503		
	<4 mg/L	7,327	0.98 (0.95 – 1.01)	0.203
	4-10 mg/L	4,522	1.05 (1.01 – 1.09)	0.006
	10-40 mg/L	2,201	1.25 (1.19 – 1.31)	<0.001
	40-100 mg/L	1,130	1.14 (1.07 – 1.21)	<0.001
	≥100 mg/L	1,323	1.43 (1.35 – 1.51)	<0.001
	SARS-CoV-2 negative	39,220		
	<4 mg/L	14,770	1.00 [reference]	..
	4-10 mg/L	10,991	1.02 (1.00 – 1.05)	0.054
	10-40 mg/L	6,011	1.04 (1.01 – 1.07)	0.016
	40-100 mg/L	3,555	1.00 (0.97 – 1.04)	0.924
Neurological disorders	≥100 mg/L	3,893	0.95 (0.91 – 0.98)	0.003
	SARS-CoV-2 positive	14,926		
	<4 mg/L	6,196	0.94 (0.91 – 0.97)	<0.001
	4-10 mg/L	4,084	0.99 (0.95 – 1.02)	0.519
	10-40 mg/L	2,264	1.12 (1.07 – 1.18)	<0.001
	40-100 mg/L	1,164	1.03 (0.97 – 1.09)	0.380
	≥100 mg/L	1,218	1.03 (0.97 – 1.10)	0.297
	SARS-CoV-2 negative	22,666		
	<4 mg/L	6,299	1.00 [reference]	..
	4-10 mg/L	5,240	1.16 (1.11 – 1.20)	<0.001
	10-40 mg/L	4,449	1.78 (1.71 – 1.85)	<0.001
	40-100 mg/L	2,928	1.88 (1.80 – 1.97)	<0.001
Respiratory disorders	≥100 mg/L	3,750	2.12 (2.03 – 2.21)	<0.001
	SARS-CoV-2 positive	6,553		
	<4 mg/L	1,960	0.92 (0.87 – 0.97)	0.002

Outcome	Peak CRP value[b]	Cases, No.	HR (95% CI)[a]	p-value
	4-10 mg/L	1,519	1.11 (1.04 – 1.17)	0.001
	10-40 mg/L	1,232	1.74 (1.64 – 1.85)	<0.001
	40-100 mg/L	813	1.96 (1.82 – 2.11)	<0.001
	≥100 mg/L	1,029	2.39 (2.24 – 2.56)	<0.001
Circulatory disorders	SARS-CoV-2 negative	35,547		
	<4 mg/L	11,176	1.00 [reference]	..
	4-10 mg/L	9,033	1.15 (1.12 – 1.18)	<0.001
	10-40 mg/L	5,980	1.49 (1.44 – 1.53)	<0.001
	40-100 mg/L	4,165	1.62 (1.56 – 1.68)	<0.001
	≥100 mg/L	5,193	1.73 (1.67 – 1.79)	<0.001
	SARS-CoV-2 positive	10,086		
	<4 mg/L	3,604	0.91 (0.88 – 0.95)	<0.001
	4-10 mg/L	2,586	1.04 (1.00 – 1.09)	0.055
	10-40 mg/L	1,634	1.44 (1.36 – 1.51)	<0.001
	40-100 mg/L	1,017	1.50 (1.40 – 1.60)	<0.001
	≥100 mg/L	1,245	1.75 (1.65 – 1.86)	<0.001
Kidney disorders	SARS-CoV-2 negative	13,746		
	<4 mg/L	2,178	1.00 [reference]	..
	4-10 mg/L	2,662	1.54 (1.46 – 1.63)	<0.001
	10-40 mg/L	3,084	2.78 (2.63 – 2.94)	<0.001
	40-100 mg/L	2,417	3.35 (3.16 – 3.55)	<0.001
	≥100 mg/L	3,405	4.12 (3.90 – 4.35)	<0.001
	SARS-CoV-2 positive	2,931		
	<4 mg/L	507	0.81 (0.74 – 0.90)	<0.001
	4-10 mg/L	541	1.22 (1.10 – 1.34)	<0.001
	10-40 mg/L	605	2.12 (1.93 – 2.32)	<0.001
	40-100 mg/L	550	3.03 (2.76 – 3.33)	<0.001
	≥100 mg/L	728	3.70 (3.39 – 4.03)	<0.001
Gastrointestinal disorders	SARS-CoV-2 negative	21,606		
	<4 mg/L	7,249	1.00 [reference]	..
	4-10 mg/L	5,622	1.08 (1.04 – 1.12)	<0.001
	10-40 mg/L	3,952	1.44 (1.39 – 1.50)	<0.001
	40-100 mg/L	2,360	1.46 (1.39 – 1.53)	<0.001
	≥100 mg/L	2,423	1.35 (1.29 – 1.42)	<0.001
	SARS-CoV-2 positive	6,185		
	<4 mg/L	2,375	0.89 (0.85 – 0.94)	<0.001
	4-10 mg/L	1,742	1.02 (0.97 – 1.08)	0.485
	10-40 mg/L	1,004	1.22 (1.14 – 1.30)	<0.001
	40-100 mg/L	524	1.16 (1.06 – 1.27)	0.001
	≥100 mg/L	540	1.17 (1.07 – 1.28)	0.001
Endocrine disorders	SARS-CoV-2 negative	10,406		
	<4 mg/L	2,426	1.00 [reference]	..
	4-10 mg/L	2,551	1.42 (1.35 – 1.50)	<0.001
	10-40 mg/L	2,233	2.22 (2.09 – 2.35)	<0.001
	40-100 mg/L	1,345	2.19 (2.05 – 2.35)	<0.001
	≥100 mg/L	1,851	2.66 (2.50 – 2.83)	<0.001
	SARS-CoV-2 positive	2,708		
	<4 mg/L	692	0.86 (0.79 – 0.94)	0.001
	4-10 mg/L	643	1.21 (1.11 – 1.33)	<0.001
	10-40 mg/L	584	2.15 (1.96 – 2.36)	<0.001
	40-100 mg/L	328	2.07 (1.84 – 2.33)	<0.001
	≥100 mg/L	461	2.76 (2.49 – 3.06)	<0.001
Hematological disorders	SARS-CoV-2 negative	18,395		
	<4 mg/L	3,942	1.00 [reference]	..
	4-10 mg/L	3,945	1.29 (1.23 – 1.34)	<0.001
	10-40 mg/L	3,923	2.08 (1.99 – 2.18)	<0.001
	40-100 mg/L	2,991	2.52 (2.40 – 2.64)	<0.001
	≥100 mg/L	3,594	2.64 (2.52 – 2.77)	<0.001
	SARS-CoV-2 positive	4,369		
	<4 mg/L	1,006	0.82 (0.77 – 0.88)	<0.001
	4-10 mg/L	926	1.09 (1.01 – 1.18)	0.020
	10-40 mg/L	939	1.88 (1.75 – 2.02)	<0.001

Outcome	Peak CRP value[b]	Cases, No.	HR (95% CI)[a]	p-value
	40-100 mg/L	643	2.12 (1.95 – 2.31)	<0.001
	≥100 mg/L	855	2.66 (2.47 – 2.87)	<0.001
Musculoskeletal disorders	SARS-CoV-2 negative	7,807		
	<4 mg/L	3,229	1.00 [reference]	..
	4-10 mg/L	2,143	0.97 (0.92 – 1.02)	0.278
	10-40 mg/L	1,253	1.19 (1.11 – 1.27)	<0.001
	40-100 mg/L	580	1.04 (0.95 – 1.14)	0.410
	≥100 mg/L	602	1.01 (0.93 – 1.11)	0.773
	SARS-CoV-2 positive	3,594		
	<4 mg/L	1,678	1.00 (0.94 – 1.07)	0.914
	4-10 mg/L	1,035	1.00 (0.93 – 1.08)	0.997
	10-40 mg/L	463	1.03 (0.93 – 1.14)	0.544
	40-100 mg/L	222	1.02 (0.89 – 1.17)	0.813
	≥100 mg/L	196	0.93 (0.80 – 1.08)	0.328
Dermatological disorders	SARS-CoV-2 negative	3,931		
	<4 mg/L	1,326	1.00 [reference]	..
	4-10 mg/L	1,020	1.14 (1.05 – 1.23)	0.002
	10-40 mg/L	701	1.51 (1.38 – 1.66)	<0.001
	40-100 mg/L	448	1.77 (1.58 – 1.97)	<0.001
	≥100 mg/L	436	1.72 (1.54 – 1.92)	<0.001
	SARS-CoV-2 positive	1,819		
	<4 mg/L	759	1.12 (1.01 – 1.23)	0.028
	4-10 mg/L	467	1.13 (1.01 – 1.26)	0.034
	10-40 mg/L	293	1.52 (1.33 – 1.73)	<0.001
	40-100 mg/L	150	1.54 (1.30 – 1.84)	<0.001
	≥100 mg/L	150	1.72 (1.45 – 2.04)	<0.001
Neuropsychiatric symptoms	SARS-CoV-2 negative	26,950		
	<4 mg/L	7,996	1.00 [reference]	..
	4-10 mg/L	6,224	1.02 (0.99 – 1.06)	0.221
	10-40 mg/L	5,001	1.38 (1.33 – 1.43)	<0.001
	40-100 mg/L	3,561	1.60 (1.53 – 1.66)	<0.001
	≥100 mg/L	4,168	1.62 (1.56 – 1.68)	<0.001
	SARS-CoV-2 positive	8,399		
	<4 mg/L	2,952	1.06 (1.01 – 1.11)	0.010
	4-10 mg/L	2,035	1.09 (1.04 – 1.15)	0.001
	10-40 mg/L	1,492	1.46 (1.38 – 1.54)	<0.001
	40-100 mg/L	972	1.62 (1.51 – 1.73)	<0.001
	≥100 mg/L	948	1.49 (1.39 – 1.59)	<0.001

Abbreviations: HR, Hazard Ratio; CI, Confidence Interval.

[a] The estimates are HRs with 95% CI from Cox Proportional Hazards model stratified by age and adjusted for confounders (sex, Charlson Comorbidity Index (CCI), parental CCI, parental mental health, employment status, income, highest level of education). All statistical tests were two-sided without correction for multiple comparisons.
[b] The reference group *SARS-CoV-2 negative and peak CRP <4 mg/L* consisted of individuals with only negative SARS-CoV-2 tests and peak CRP measurement <4 mg/L.

Supplementary Table 20: CRP value.

Hazard ratios of specific first mental health or general medical disorders among individuals with positive SARS-CoV-2 test by peak CRP value in quantiles.

Outcome	Peak CRP value[b]	Cases, No.	HR (95% CI)[a]	p-value
Mental disorders and general medical conditions	[0,0.7)	1,122	1.00 [reference]	..
	[0.7,1)	489	1.08 (0.97 – 1.20)	0.179
	[1,1.1)	1,236	1.08 (1.00 – 1.17)	0.060
	[1.1,2)	982	1.09 (1.00 – 1.19)	0.054
	[2,2.9)	723	1.13 (1.03 – 1.24)	0.009
	[2.9,3)	1,215	1.02 (0.94 – 1.11)	0.651
	[3,3.1)	890	1.02 (0.93 – 1.11)	0.714
	[3.1,4)	516	1.32 (1.19 – 1.47)	<0.001
	[4,4.1)	2,488	1.05 (0.98 – 1.13)	0.161
	[4.1,6)	825	1.14 (1.04 – 1.25)	0.005
	[6,8)	701	1.29 (1.18 – 1.42)	<0.001
	[8,10)	506	1.33 (1.20 – 1.48)	<0.001
	[10,15)	769	1.34 (1.22 – 1.47)	<0.001
	[15,30)	1,071	1.25 (1.15 – 1.36)	<0.001
	[30,50)	728	1.23 (1.12 – 1.35)	<0.001
	[50,90)	758	1.13 (1.03 – 1.24)	0.008
	[90,165)	870	1.36 (1.24 – 1.49)	<0.001
	[165,678]	824	1.65 (1.50 – 1.80)	<0.001
Mental disorders	[0,0.7)	698	1.00 [reference]	..
	[0.7,1)	250	0.92 (0.80 – 1.07)	0.268
	[1,1.1)	660	0.97 (0.87 – 1.08)	0.542
	[1.1,2)	500	0.92 (0.82 – 1.04)	0.186
	[2,2.9)	345	0.85 (0.75 – 0.97)	0.017
	[2.9,3)	507	0.77 (0.69 – 0.86)	<0.001
	[3,3.1)	429	0.91 (0.81 – 1.03)	0.122
	[3.1,4)	249	0.96 (0.83 – 1.12)	0.630
	[4,4.1)	1,134	0.85 (0.77 – 0.93)	0.001
	[4.1,6)	400	0.82 (0.72 – 0.93)	0.002
	[6,8)	343	0.90 (0.79 – 1.03)	0.117
	[8,10)	224	0.83 (0.71 – 0.96)	0.015
	[10,15)	370	0.92 (0.81 – 1.04)	0.189
	[15,30)	476	0.82 (0.73 – 0.92)	0.001
	[30,50)	335	0.91 (0.79 – 1.03)	0.138
	[50,90)	327	0.84 (0.74 – 0.96)	0.010
	[90,165)	297	0.87 (0.75 – 0.99)	0.039
	[165,723]	242	0.96 (0.83 – 1.12)	0.627
General medical conditions	[0,0.7)	1,106	1.00 [reference]	..
	[0.7,1)	542	1.19 (1.07 – 1.32)	0.001
	[1,1.1)	1,240	1.12 (1.03 – 1.21)	0.007
	[1.1,2)	1,035	1.14 (1.05 – 1.25)	0.002
	[2,2.9)	778	1.23 (1.12 – 1.35)	<0.001
	[2.9,3)	1,245	1.09 (1.00 – 1.18)	0.044
	[3,3.1)	877	1.04 (0.95 – 1.14)	0.352
	[3.1,4)	504	1.32 (1.19 – 1.47)	<0.001
	[4,4.1)	2,450	1.09 (1.02 – 1.17)	0.015
	[4.1,6)	867	1.26 (1.15 – 1.38)	<0.001
	[6,8)	701	1.38 (1.25 – 1.52)	<0.001
	[8,10)	504	1.44 (1.29 – 1.60)	<0.001
	[10,15)	752	1.43 (1.30 – 1.57)	<0.001
	[15,30)	1,041	1.40 (1.28 – 1.52)	<0.001
	[30,50)	690	1.39 (1.26 – 1.53)	<0.001
	[50,90)	717	1.31 (1.19 – 1.44)	<0.001
	[90,165)	777	1.50 (1.36 – 1.64)	<0.001
	[165,678]	677	1.80 (1.63 – 1.98)	<0.001
	[0,0.7)	887	1.00 [reference]	..
	[0.7,1)	458	1.10 (0.98 – 1.23)	0.090
	[1,1.1)	978	1.07 (0.97 – 1.17)	0.161

Outcome	Peak CRP value[b]	Cases, No.	HR (95% CI)[a]	p-value
	[1.1,2)	998	1.13 (1.03 – 1.24)	0.007
	[2,2.9)	755	1.19 (1.08 – 1.31)	0.001
	[2.9,3)	924	0.85 (0.77 – 0.93)	<0.001
	[3,3.1)	770	1.04 (0.94 – 1.14)	0.439
	[3.1,4)	426	1.03 (0.91 – 1.15)	0.661
Neurological disorders	[4,4.1)	2,099	1.03 (0.95 – 1.11)	0.466
	[4.1,6)	835	1.09 (1.00 – 1.20)	0.062
	[6,8)	670	1.16 (1.05 – 1.28)	0.004
	[8,10)	480	1.18 (1.05 – 1.31)	0.004
	[10,15)	748	1.20 (1.09 – 1.33)	<0.001
	[15,30)	1,096	1.20 (1.09 – 1.31)	<0.001
	[30,50)	725	1.21 (1.09 – 1.33)	<0.001
	[50,90)	735	1.10 (0.99 – 1.21)	0.066
	[90,165)	746	1.11 (1.01 – 1.23)	0.037
	[165,700]	596	1.09 (0.98 – 1.21)	0.101
	[0,0.7)	239	1.00 [reference]	..
	[0.7,1)	137	1.18 (0.95 – 1.45)	0.129
	[1,1.1)	280	1.14 (0.96 – 1.36)	0.125
	[1.1,2)	307	1.21 (1.02 – 1.44)	0.025
	[2,2.9)	269	1.47 (1.23 – 1.75)	<0.001
	[2.9,3)	337	1.19 (1.00 – 1.40)	0.044
	[3,3.1)	214	1.06 (0.88 – 1.28)	0.522
	[3.1,4)	177	1.49 (1.23 – 1.81)	<0.001
Respiratory disorders	[4,4.1)	603	1.09 (0.94 – 1.26)	0.273
	[4.1,6)	368	1.69 (1.44 – 2.00)	<0.001
	[6,8)	322	1.91 (1.61 – 2.26)	<0.001
	[8,10)	226	1.96 (1.63 – 2.35)	<0.001
	[10,15)	371	2.08 (1.76 – 2.45)	<0.001
	[15,30)	616	2.30 (1.98 – 2.68)	<0.001
	[30,50)	445	2.47 (2.10 – 2.89)	<0.001
	[50,90)	505	2.46 (2.10 – 2.87)	<0.001
	[90,165)	593	2.90 (2.49 – 3.37)	<0.001
	[165,723]	544	3.37 (2.89 – 3.93)	<0.001
	[0,0.7)	428	1.00 [reference]	..
	[0.7,1)	246	1.05 (0.90 – 1.23)	0.562
	[1,1.1)	480	1.04 (0.91 – 1.18)	0.596
	[1.1,2)	579	1.17 (1.03 – 1.32)	0.017
	[2,2.9)	450	1.28 (1.12 – 1.47)	<0.001
	[2.9,3)	707	1.31 (1.16 – 1.48)	<0.001
	[3,3.1)	389	0.94 (0.82 – 1.08)	0.362
	[3.1,4)	325	1.46 (1.26 – 1.69)	<0.001
Circulatory disorders	[4,4.1)	1,245	1.15 (1.03 – 1.29)	0.011
	[4.1,6)	565	1.42 (1.25 – 1.61)	<0.001
	[6,8)	460	1.53 (1.34 – 1.75)	<0.001
	[8,10)	316	1.58 (1.36 – 1.82)	<0.001
	[10,15)	515	1.70 (1.49 – 1.93)	<0.001
	[15,30)	786	1.79 (1.59 – 2.01)	<0.001
	[30,50)	560	1.89 (1.67 – 2.15)	<0.001
	[50,90)	682	1.99 (1.76 – 2.24)	<0.001
	[90,165)	670	1.96 (1.73 – 2.21)	<0.001
	[165,700]	683	2.44 (2.16 – 2.76)	<0.001
	[0,0.7)	50	1.00 [reference]	..
	[0.7,1)	31	1.08 (0.69 – 1.70)	0.724
	[1,1.1)	73	1.32 (0.92 – 1.89)	0.130
	[1.1,2)	83	1.26 (0.89 – 1.79)	0.197
	[2,2.9)	86	1.72 (1.21 – 2.44)	0.002
	[2.9,3)	80	1.16 (0.81 – 1.65)	0.417
	[3,3.1)	49	1.00 (0.68 – 1.49)	0.983
	[3.1,4)	55	1.69 (1.15 – 2.47)	0.008
Kidney disorders	[4,4.1)	186	1.34 (0.98 – 1.83)	0.068
	[4.1,6)	127	2.02 (1.45 – 2.80)	<0.001
	[6,8)	128	2.53 (1.82 – 3.51)	<0.001

Outcome	Peak CRP value[b]	Cases, No.	HR (95% CI)[a]	p-value
	[8,10)	100	2.81 (2.00 – 3.95)	<0.001
	[10,15)	166	2.90 (2.11 – 3.98)	<0.001
	[15,30)	298	3.18 (2.36 – 4.30)	<0.001
	[30,50)	245	3.64 (2.68 – 4.95)	<0.001
	[50,90)	374	4.84 (3.60 – 6.52)	<0.001
	[90,165)	393	4.91 (3.65 – 6.61)	<0.001
	[165,723]	407	6.47 (4.81 – 8.70)	<0.001
Gastrointestinal disorders	[0,0.7)	310	1.00 [reference]	..
	[0.7,1)	164	1.11 (0.92 – 1.35)	0.264
	[1,1.1)	395	1.21 (1.05 – 1.41)	0.011
	[1.1,2)	364	1.17 (1.01 – 1.36)	0.041
	[2,2.9)	295	1.30 (1.11 – 1.52)	0.001
	[2.9,3)	404	1.15 (0.99 – 1.33)	0.070
	[3,3.1)	253	0.98 (0.83 – 1.16)	0.796
	[3.1,4)	190	1.31 (1.10 – 1.57)	0.003
	[4,4.1)	840	1.18 (1.03 – 1.34)	0.014
	[4.1,6)	359	1.33 (1.14 – 1.55)	<0.001
	[6,8)	312	1.51 (1.29 – 1.77)	<0.001
	[8,10)	231	1.61 (1.36 – 1.91)	<0.001
	[10,15)	331	1.52 (1.30 – 1.77)	<0.001
	[15,30)	510	1.62 (1.40 – 1.87)	<0.001
	[30,50)	284	1.39 (1.18 – 1.63)	<0.001
	[50,90)	346	1.55 (1.33 – 1.81)	<0.001
	[90,165)	317	1.46 (1.24 – 1.71)	<0.001
	[165,712]	280	1.61 (1.37 – 1.90)	<0.001
Endocrine disorders	[0,0.7)	66	1.00 [reference]	..
	[0.7,1)	40	1.12 (0.76 – 1.66)	0.573
	[1,1.1)	94	1.33 (0.97 – 1.82)	0.076
	[1.1,2)	117	1.47 (1.09 – 1.99)	0.012
	[2,2.9)	95	1.63 (1.19 – 2.24)	0.002
	[2.9,3)	120	1.40 (1.03 – 1.89)	0.030
	[3,3.1)	77	1.30 (0.94 – 1.81)	0.118
	[3.1,4)	83	2.19 (1.59 – 3.03)	<0.001
	[4,4.1)	218	1.30 (0.99 – 1.71)	0.063
	[4.1,6)	168	2.40 (1.81 – 3.20)	<0.001
	[6,8)	134	2.50 (1.86 – 3.36)	<0.001
	[8,10)	123	3.31 (2.46 – 4.47)	<0.001
	[10,15)	187	3.25 (2.45 – 4.31)	<0.001
	[15,30)	298	3.44 (2.63 – 4.50)	<0.001
	[30,50)	184	3.13 (2.36 – 4.15)	<0.001
	[50,90)	209	3.17 (2.40 – 4.19)	<0.001
	[90,165)	230	3.49 (2.65 – 4.60)	<0.001
	[165,723]	265	5.07 (3.86 – 6.66)	<0.001
Hematological disorders	[0,0.7)	138	1.00 [reference]	..
	[0.7,1)	70	0.93 (0.70 – 1.24)	0.633
	[1,1.1)	162	1.06 (0.85 – 1.34)	0.589
	[1.1,2)	157	0.93 (0.74 – 1.18)	0.562
	[2,2.9)	152	1.20 (0.95 – 1.51)	0.126
	[2.9,3)	151	0.84 (0.67 – 1.06)	0.141
	[3,3.1)	101	0.78 (0.60 – 1.01)	0.059
	[3.1,4)	75	0.90 (0.68 – 1.20)	0.483
	[4,4.1)	407	1.13 (0.93 – 1.37)	0.213
	[4.1,6)	203	1.29 (1.04 – 1.60)	0.022
	[6,8)	181	1.44 (1.15 – 1.80)	0.001
	[8,10)	135	1.53 (1.21 – 1.95)	<0.001
	[10,15)	262	1.90 (1.54 – 2.34)	<0.001
	[15,30)	489	2.30 (1.90 – 2.79)	<0.001
	[30,50)	330	2.27 (1.85 – 2.77)	<0.001
	[50,90)	421	2.57 (2.11 – 3.12)	<0.001
	[90,165)	519	3.12 (2.58 – 3.77)	<0.001
	[165,723]	416	3.12 (2.57 – 3.80)	<0.001
	[0,0.7)	206	1.00 [reference]	..

Outcome	Peak CRP value[b]	Cases, No.	HR (95% CI)[a]	p-value
	[0.7,1)	116	1.22 (0.97 – 1.53)	0.093
	[1,1.1)	210	1.00 (0.83 – 1.21)	0.987
	[1.1,2)	279	1.42 (1.19 – 1.70)	<0.001
	[2,2.9)	219	1.52 (1.26 – 1.85)	<0.001
	[2.9,3)	313	1.34 (1.12 – 1.60)	0.001
	[3,3.1)	185	1.10 (0.90 – 1.34)	0.347
	[3.1,4)	150	1.64 (1.33 – 2.03)	<0.001
Musculoskeletal disorders	[4,4.1)	480	1.07 (0.91 – 1.26)	0.403
	[4.1,6)	243	1.45 (1.20 – 1.75)	<0.001
	[6,8)	170	1.32 (1.07 – 1.62)	0.008
	[8,10)	142	1.63 (1.32 – 2.02)	<0.001
	[10,15)	163	1.27 (1.04 – 1.57)	0.021
	[15,30)	212	1.21 (1.00 – 1.47)	0.054
	[30,50)	145	1.35 (1.09 – 1.67)	0.006
	[50,90)	147	1.30 (1.05 – 1.61)	0.016
	[90,165)	122	1.15 (0.92 – 1.44)	0.223
	[165,723]	92	1.09 (0.85 – 1.40)	0.494
	[0,0.7)	131	1.00 [reference]	..
	[0.7,1)	49	0.95 (0.69 – 1.32)	0.777
	[1,1.1)	138	1.08 (0.85 – 1.37)	0.539
	[1.1,2)	94	0.92 (0.70 – 1.19)	0.516
	[2,2.9)	82	1.07 (0.81 – 1.41)	0.624
	[2.9,3)	96	0.78 (0.60 – 1.01)	0.064
	[3,3.1)	117	1.30 (1.01 – 1.67)	0.039
	[3.1,4)	52	1.07 (0.78 – 1.48)	0.661
Dermatological disorders	[4,4.1)	261	1.05 (0.85 – 1.29)	0.675
	[4.1,6)	91	1.01 (0.77 – 1.32)	0.939
	[6,8)	67	0.95 (0.71 – 1.28)	0.739
	[8,10)	48	0.98 (0.70 – 1.36)	0.894
	[10,15)	100	1.37 (1.05 – 1.78)	0.019
	[15,30)	132	1.26 (0.99 – 1.61)	0.064
	[30,50)	101	1.55 (1.19 – 2.01)	0.001
	[50,90)	95	1.39 (1.07 – 1.82)	0.015
	[90,165)	86	1.39 (1.06 – 1.83)	0.018
	[165,723]	79	1.72 (1.29 – 2.28)	<0.001
	[0,0.7)	404	1.00 [reference]	..
	[0.7,1)	217	1.05 (0.89 – 1.24)	0.539
	[1,1.1)	451	1.04 (0.91 – 1.19)	0.560
	[1.1,2)	430	0.96 (0.83 – 1.10)	0.520
	[2,2.9)	347	1.04 (0.90 – 1.20)	0.615
	[2.9,3)	440	0.91 (0.79 – 1.04)	0.172
	[3,3.1)	433	1.25 (1.09 – 1.44)	0.001
	[3.1,4)	230	1.06 (0.90 – 1.25)	0.487
Neuropsychiatric symptoms	[4,4.1)	975	1.00 (0.89 – 1.12)	0.944
	[4.1,6)	460	1.13 (0.99 – 1.30)	0.066
	[6,8)	334	1.05 (0.91 – 1.21)	0.517
	[8,10)	266	1.20 (1.03 – 1.40)	0.021
	[10,15)	448	1.32 (1.16 – 1.51)	<0.001
	[15,30)	741	1.45 (1.28 – 1.64)	<0.001
	[30,50)	534	1.55 (1.36 – 1.76)	<0.001
	[50,90)	645	1.68 (1.48 – 1.90)	<0.001
	[90,165)	600	1.57 (1.38 – 1.78)	<0.001
	[165,700]	444	1.42 (1.24 – 1.62)	<0.001

Abbreviations: HR, Hazard Ratio; CI, Confidence Interval; CRP, C-Reactive Protein.

[a] The estimates are HRs with 95% CI from Cox Proportional Hazards model stratified by age and adjusted for confounders (sex, Charlson Comorbidity Index (CCI), parental CCI, parental mental health, employment status, income, highest level of education). All statistical tests were two-sided without correction for multiple comparisons.

Supplementary Figure 4: Density, hazard ratios, and confidence intervals of the association between peak C-reactive Protein (CRP), admission status and mental disorders or general medical conditions.

a Density of peak CRP by admission status.

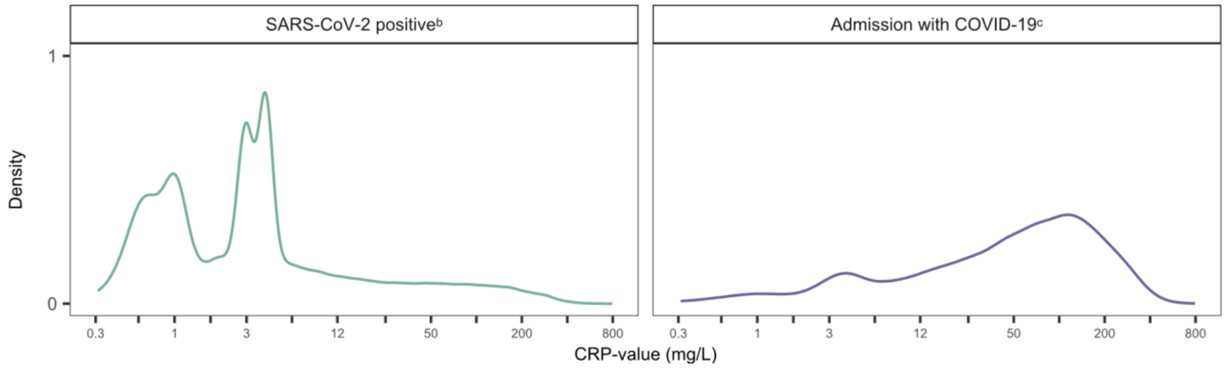

b Hazard ratios and 95% confidence intervals of the association between peak CRP, admission status and mental disorders or general medical conditions.

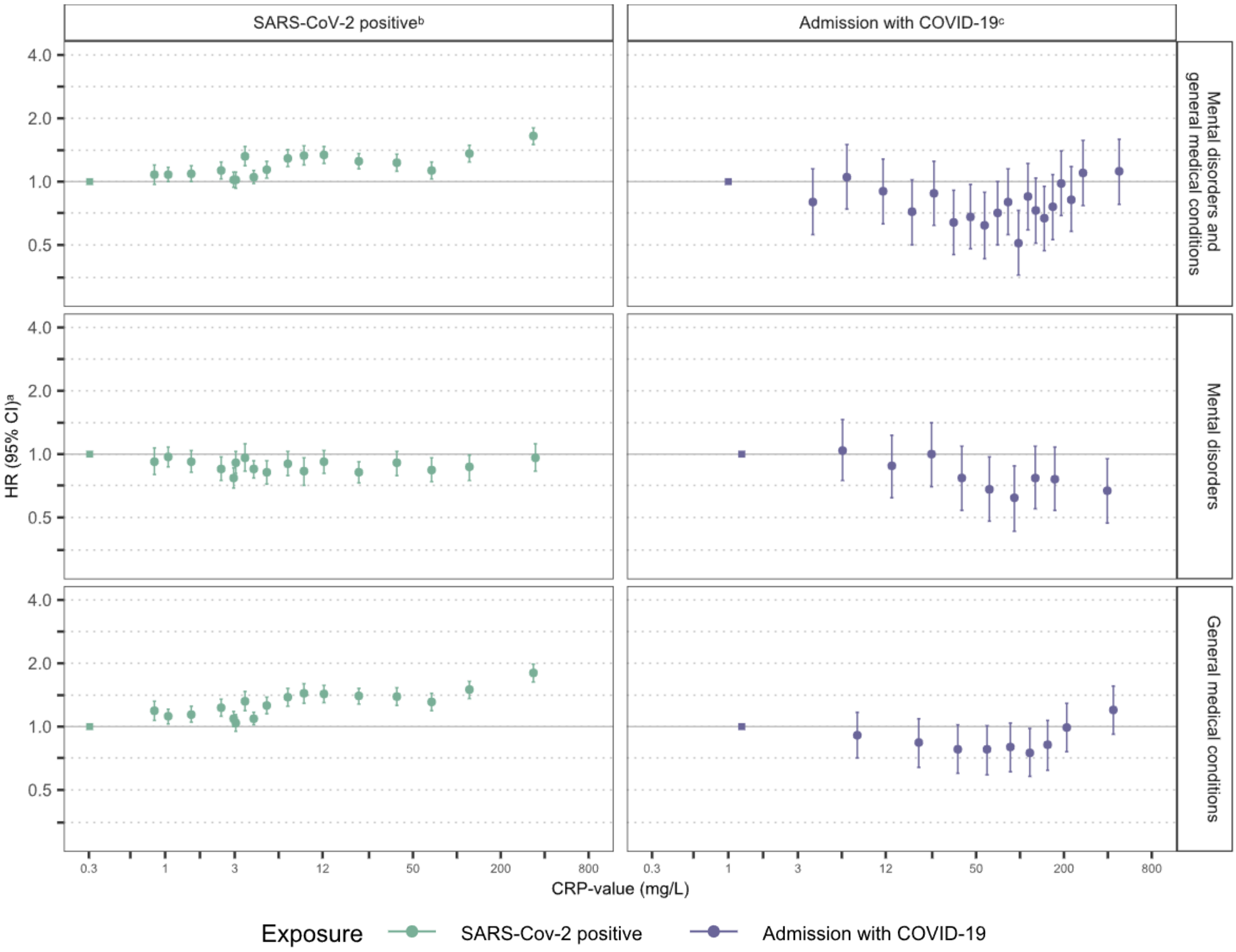

The estimates presented in panel **b** of the figure are available in Supplementary Table 20 and 23. Source data are provided as a Source Data file.

Abbreviations: HR, Hazard Ratio; CI, Confidence Interval; CRP, C-Reactive Protein.

[a] Based on Cox Proportional Hazards model stratified by age and adjusted for confounders (sex, Charlson Comorbidity Index (CCI), parental CCI, parental psychiatric history, employment status, income, and highest level of education).

[b] Including individuals with a CRP measurement within 14 days before a positive SARS-CoV-2 test. The CRP groups were defined by dividing all individuals in 18 equally sized groups depending on case distribution. The reference group was the lowest CRP group marked with a green square in the plot.

[c] Including individuals with a CRP measurement within 2 days before or during admission with COVID-19. The CRP groups were defined by dividing all individuals in 20 (for the outcome "*any mental disorders or general medical conditions*") or 10 (all other outcomes) equally sized groups depending on case distribution. The reference group was the lowest CRP group marked with a purple square in the plot. The definition of admission to the hospital is summarized in Supplementary Table 30.

Supplementary Table 21: CRP value and age group.

Hazard ratios of any first mental health or general medical disorders among individuals with positive SARS-CoV-2 test by peak C-Reactive Protein (CRP) value in quantiles and by age group.

Outcome	Age group	Peak CRP value[b]	Cases, No.	HR (95% CI)[a]	p-value
		[0,0.7)	239	1.00 [reference]	..
		[0.7,1)	33	0.92 (0.64 - 1.32)	0.649
		[1,1.1)	149	1.03 (0.84 - 1.26)	0.773
		[1.1,2)	72	1.17 (0.90 - 1.53)	0.238
		[2,2.9)	38	0.91 (0.65 - 1.28)	0.591
		[2.9,3)	153	1.04 (0.85 - 1.27)	0.721
		[3,3.1)	97	0.93 (0.73 - 1.18)	0.545
		[3.1,4)	37	1.17 (0.83 - 1.65)	0.381
Mental disorders and general medical conditions	<18	[4,4.1)	282	1.08 (0.91 - 1.29)	0.367
		[4.1,6)	74	1.28 (0.99 - 1.66)	0.065
		[6,8)	63	1.26 (0.95 - 1.66)	0.104
		[8,10)	41	0.95 (0.68 - 1.32)	0.754
		[10,15)	73	1.03 (0.79 - 1.33)	0.847
		[15,30)	132	0.94 (0.76 - 1.17)	0.598
		[30,50)	78	0.71 (0.55 - 0.92)	0.009
		[50,90)	83	0.71 (0.55 - 0.91)	0.008
		[90,165)	98	1.04 (0.82 - 1.32)	0.746
		[165,678]	56	1.12 (0.84 - 1.50)	0.430
		[0,0.7)	410	1.00 [reference]	..
		[0.7,1)	199	1.38 (1.17 - 1.63)	<0.001
		[1,1.1)	585	1.24 (1.09 - 1.40)	0.001
		[1.1,2)	332	1.26 (1.09 - 1.46)	0.002
		[2,2.9)	258	1.21 (1.03 - 1.41)	0.018
		[2.9,3)	353	1.11 (0.96 - 1.28)	0.149
		[3,3.1)	281	1.15 (0.99 - 1.34)	0.068
		[3.1,4)	190	1.59 (1.34 - 1.89)	<0.001
Mental disorders and general medical conditions	18-39	[4,4.1)	900	1.14 (1.01 - 1.28)	0.031
		[4.1,6)	296	1.23 (1.05 - 1.42)	0.008
		[6,8)	254	1.31 (1.12 - 1.54)	0.001
		[8,10)	199	1.47 (1.24 - 1.74)	<0.001
		[10,15)	297	1.40 (1.21 - 1.63)	<0.001
		[15,30)	374	1.21 (1.06 - 1.40)	0.007
		[30,50)	227	1.15 (0.98 - 1.35)	0.098
		[50,90)	196	0.96 (0.81 - 1.14)	0.642
		[90,165)	198	1.21 (1.02 - 1.44)	0.025
		[165,678]	165	1.38 (1.15 - 1.65)	<0.001
		[0,0.7)	360	1.00 [reference]	..
		[0.7,1)	175	0.92 (0.77 - 1.11)	0.385
		[1,1.1)	331	0.85 (0.73 - 0.99)	0.032
		[1.1,2)	361	0.98 (0.85 - 1.14)	0.818
		[2,2.9)	289	1.15 (0.99 - 1.35)	0.069
		[2.9,3)	466	0.98 (0.85 - 1.12)	0.778
		[3,3.1)	344	0.95 (0.82 - 1.10)	0.496
		[3.1,4)	184	1.26 (1.05 - 1.50)	0.011
Mental disorders and general medical conditions	40-59	[4,4.1)	868	1.00 (0.88 - 1.13)	0.967
		[4.1,6)	277	1.06 (0.91 - 1.24)	0.437
		[6,8)	247	1.31 (1.11 - 1.54)	0.001
		[8,10)	163	1.33 (1.10 - 1.59)	0.003
		[10,15)	236	1.35 (1.15 - 1.59)	<0.001
		[15,30)	305	1.36 (1.17 - 1.59)	<0.001
		[30,50)	201	1.41 (1.19 - 1.68)	<0.001
		[50,90)	209	1.29 (1.08 - 1.52)	0.004
		[90,165)	223	1.38 (1.17 - 1.63)	<0.001
		[165,678]	258	1.92 (1.63 - 2.25)	<0.001
		[0,0.7)	110	1.00 [reference]	..
		[0.7,1)	79	1.12 (0.84 - 1.50)	0.435

Outcome	Age group	Peak CRP value[b]	Cases, No.	HR (95% CI)[a]	p-value
		[1,1.1)	157	1.38 (1.08 - 1.76)	0.009
		[1.1,2)	204	1.19 (0.95 - 1.50)	0.136
		[2,2.9)	129	1.25 (0.97 - 1.62)	0.083
		[2.9,3)	229	1.11 (0.88 - 1.39)	0.373
		[3,3.1)	157	1.16 (0.91 - 1.48)	0.231
		[3.1,4)	97	1.31 (1.00 - 1.72)	0.052
Mental disorders and general medical conditions	60-79	[4,4.1)	412	1.15 (0.93 - 1.42)	0.183
		[4.1,6)	163	1.27 (1.00 - 1.62)	0.051
		[6,8)	123	1.41 (1.09 - 1.83)	0.009
		[8,10)	95	1.66 (1.26 - 2.19)	<0.001
		[10,15)	144	1.71 (1.33 - 2.19)	<0.001
		[15,30)	209	1.59 (1.26 - 2.00)	<0.001
		[30,50)	166	1.66 (1.30 - 2.11)	<0.001
		[50,90)	216	1.78 (1.41 - 2.23)	<0.001
		[90,165)	286	1.97 (1.58 - 2.46)	<0.001
		[165,678]	299	2.22 (1.78 - 2.76)	<0.001
		[0,0.7)	≤5[b]	1.00 [reference]	..
		[0.7,1)	≤5[b]	..	..
		[1,1.1)	14	2.83 (0.81 - 9.84)	0.102
		[1.1,2)	13	1.86 (0.53 - 6.52)	0.334
		[2,2.9)	9	1.61 (0.44 - 5.95)	0.475
		[2.9,3)	14	1.04 (0.30 - 3.62)	0.952
		[3,3.1)	11	1.39 (0.39 - 4.97)	0.616
		[3.1,4)	8	2.71 (0.72 - 10.21)	0.141
Mental disorders and general medical conditions	≥80	[4,4.1)	26	1.27 (0.39 - 4.21)	0.691
		[4.1,6)	15	1.90 (0.55 - 6.57)	0.310
		[6,8)	14	2.97 (0.85 - 10.35)	0.087
		[8,10)	8	1.18 (0.31 - 4.45)	0.810
		[10,15)	19	1.77 (0.52 - 5.98)	0.359
		[15,30)	51	2.77 (0.86 - 8.89)	0.086
		[30,50)	56	3.52 (1.10 - 11.26)	0.034
		[50,90)	54	2.11 (0.66 - 6.75)	0.210
		[90,165)	65	2.31 (0.72 - 7.36)	0.157
		[165,678]	46	2.37 (0.74 - 7.62)	0.148
		[0,0.7)	176	1.00 [reference]	..
		[0.7,1)	26	0.91 (0.61 - 1.38)	0.668
		[1,1.1)	95	0.87 (0.68 - 1.11)	0.269
		[1.1,2)	39	0.82 (0.58 - 1.16)	0.257
		[2,2.9)	22	0.64 (0.41 - 1.00)	0.050
		[2.9,3)	90	0.84 (0.65 - 1.08)	0.166
		[3,3.1)	68	0.90 (0.68 - 1.19)	0.448
		[3.1,4)	24	1.01 (0.66 - 1.55)	0.951
Mental disorders	<18	[4,4.1)	170	0.91 (0.74 - 1.13)	0.389
		[4.1,6)	37	0.81 (0.57 - 1.15)	0.235
		[6,8)	35	0.98 (0.68 - 1.41)	0.914
		[8,10)	21	0.70 (0.44 - 1.09)	0.117
		[10,15)	35	0.70 (0.49 - 1.01)	0.058
		[15,30)	63	0.71 (0.53 - 0.95)	0.020
		[30,50)	30	0.45 (0.31 - 0.67)	<0.001
		[50,90)	34	0.49 (0.34 - 0.71)	<0.001
		[90,165)	27	0.50 (0.34 - 0.75)	0.001
		[165,723]	18	0.65 (0.40 - 1.05)	0.078
		[0,0.7)	321	1.00 [reference]	..
		[0.7,1)	122	1.02 (0.83 - 1.25)	0.878
		[1,1.1)	348	1.01 (0.87 - 1.17)	0.914
		[1.1,2)	240	1.09 (0.92 - 1.29)	0.306
		[2,2.9)	163	0.94 (0.78 - 1.13)	0.497
		[2.9,3)	217	0.84 (0.71 - 1.00)	0.054
		[3,3.1)	195	1.03 (0.86 - 1.23)	0.738
		[3.1,4)	109	1.02 (0.82 - 1.26)	0.886
Mental disorders	18-39	[4,4.1)	551	0.93 (0.81 - 1.07)	0.320
		[4.1,6)	181	0.86 (0.71 - 1.03)	0.094

Outcome	Age group	Peak CRP value[b]	Cases, No.	HR (95% CI)[a]	p-value
		[6,8)	160	0.96 (0.80 - 1.16)	0.688
		[8,10)	99	0.83 (0.66 - 1.04)	0.097
		[10,15)	181	1.02 (0.85 - 1.23)	0.813
		[15,30)	175	0.70 (0.58 - 0.84)	<0.001
		[30,50)	130	0.90 (0.73 - 1.10)	0.298
		[50,90)	111	0.77 (0.62 - 0.96)	0.019
		[90,165)	87	0.79 (0.63 - 1.00)	0.055
		[165,723]	64	0.86 (0.66 - 1.12)	0.270
Mental disorders	40-59	[0,0.7)	158	1.00 [reference]	..
		[0.7,1)	63	0.71 (0.53 - 0.95)	0.023
		[1,1.1)	159	0.94 (0.76 - 1.17)	0.591
		[1.1,2)	152	0.81 (0.65 - 1.01)	0.060
		[2,2.9)	110	0.83 (0.65 - 1.05)	0.124
		[2.9,3)	144	0.68 (0.54 - 0.85)	0.001
		[3,3.1)	128	0.84 (0.67 - 1.06)	0.146
		[3.1,4)	72	0.85 (0.64 - 1.13)	0.261
		[4,4.1)	302	0.77 (0.64 - 0.94)	0.009
		[4.1,6)	96	0.64 (0.50 - 0.83)	0.001
		[6,8)	89	0.78 (0.60 - 1.01)	0.064
		[8,10)	59	0.79 (0.59 - 1.06)	0.122
		[10,15)	88	0.86 (0.66 - 1.11)	0.251
		[15,30)	105	0.84 (0.66 - 1.08)	0.176
		[30,50)	72	1.02 (0.77 - 1.34)	0.907
		[50,90)	61	0.85 (0.63 - 1.14)	0.281
		[90,165)	67	0.96 (0.72 - 1.28)	0.784
		[165,723]	53	0.93 (0.68 - 1.27)	0.645
Mental disorders	60-79	[0,0.7)	37	1.00 [reference]	..
		[0.7,1)	35	1.36 (0.86 - 2.16)	0.190
		[1,1.1)	55	1.31 (0.87 - 1.99)	0.201
		[1.1,2)	57	0.93 (0.61 - 1.41)	0.727
		[2,2.9)	42	0.96 (0.62 - 1.49)	0.847
		[2.9,3)	47	0.74 (0.48 - 1.14)	0.174
		[3,3.1)	32	0.76 (0.47 - 1.22)	0.255
		[3.1,4)	34	1.15 (0.72 - 1.83)	0.555
		[4,4.1)	89	0.75 (0.51 - 1.10)	0.142
		[4.1,6)	70	1.31 (0.88 - 1.95)	0.182
		[6,8)	46	1.15 (0.74 - 1.77)	0.531
		[8,10)	28	1.04 (0.64 - 1.70)	0.871
		[10,15)	48	1.16 (0.76 - 1.78)	0.493
		[15,30)	91	1.44 (0.98 - 2.11)	0.062
		[30,50)	69	1.55 (1.04 - 2.31)	0.032
		[50,90)	84	1.60 (1.09 - 2.36)	0.017
		[90,165)	83	1.49 (1.01 - 2.19)	0.045
		[165,723]	80	1.62 (1.09 - 2.39)	0.016
Mental disorders	≥80	[0,0.7)	6	1.00 [reference]	..
		[0.7,1)	≤5[b]	..	..
		[1,1.1)	≤5[b]	..	..
		[1.1,2)	12	0.91 (0.34 - 2.42)	0.846
		[2,2.9)	8	0.74 (0.26 - 2.12)	0.572
		[2.9,3)	9	0.77 (0.27 - 2.16)	0.617
		[3,3.1)	6	0.67 (0.22 - 2.08)	0.487
		[3.1,4)	10	1.31 (0.48 - 3.60)	0.603
		[4,4.1)	22	0.83 (0.33 - 2.04)	0.678
		[4.1,6)	16	0.99 (0.39 - 2.52)	0.979
		[6,8)	13	0.91 (0.35 - 2.40)	0.853
		[8,10)	17	1.52 (0.60 - 3.86)	0.377
		[10,15)	18	0.92 (0.36 - 2.31)	0.853
		[15,30)	42	1.16 (0.49 - 2.72)	0.739
		[30,50)	34	1.18 (0.50 - 2.81)	0.709
		[50,90)	37	1.06 (0.45 - 2.52)	0.890
		[90,165)	33	0.91 (0.38 - 2.19)	0.841
		[165,723]	27	1.01 (0.42 - 2.45)	0.977

Outcome	Age group	Peak CRP value[b]	Cases, No.	HR (95% CI)[a]	p-value
		[0,0.7)	206	1.00 [reference]	..
		[0.7,1)	33	1.10 (0.76 - 1.59)	0.605
		[1,1.1)	126	1.03 (0.82 - 1.28)	0.815
		[1.1,2)	68	1.31 (0.99 - 1.72)	0.057
		[2,2.9)	38	1.11 (0.79 - 1.57)	0.541
		[2.9,3)	134	1.11 (0.89 - 1.38)	0.353
		[3,3.1)	80	0.93 (0.72 - 1.21)	0.603
		[3.1,4)	33	1.27 (0.88 - 1.83)	0.202
General medical conditions	<18	[4,4.1)	220	1.04 (0.86 - 1.26)	0.673
		[4.1,6)	65	1.36 (1.03 - 1.80)	0.030
		[6,8)	49	1.22 (0.89 - 1.66)	0.214
		[8,10)	35	1.05 (0.73 - 1.50)	0.785
		[10,15)	60	1.10 (0.82 - 1.47)	0.520
		[15,30)	112	1.06 (0.84 - 1.34)	0.614
		[30,50)	61	0.76 (0.57 - 1.01)	0.056
		[50,90)	61	0.71 (0.53 - 0.94)	0.018
		[90,165)	68	1.01 (0.77 - 1.33)	0.920
		[165,678]	44	1.34 (0.97 - 1.86)	0.076
		[0,0.7)	400	1.00 [reference]	..
		[0.7,1)	224	1.62 (1.38 - 1.91)	<0.001
		[1,1.1)	543	1.23 (1.08 - 1.40)	0.001
		[1.1,2)	320	1.27 (1.10 - 1.47)	0.001
		[2,2.9)	260	1.30 (1.12 - 1.52)	0.001
		[2.9,3)	347	1.18 (1.02 - 1.37)	0.022
		[3,3.1)	256	1.15 (0.99 - 1.35)	0.075
		[3.1,4)	170	1.51 (1.26 - 1.81)	<0.001
General medical conditions	18-39	[4,4.1)	865	1.21 (1.07 - 1.36)	0.002
		[4.1,6)	301	1.38 (1.18 - 1.60)	<0.001
		[6,8)	250	1.45 (1.24 - 1.70)	<0.001
		[8,10)	185	1.54 (1.29 - 1.83)	<0.001
		[10,15)	272	1.46 (1.25 - 1.71)	<0.001
		[15,30)	354	1.37 (1.19 - 1.58)	<0.001
		[30,50)	185	1.17 (0.98 - 1.39)	0.084
		[50,90)	175	1.08 (0.90 - 1.29)	0.397
		[90,165)	148	1.17 (0.97 - 1.42)	0.096
		[165,678]	114	1.38 (1.12 - 1.69)	0.003
		[0,0.7)	377	1.00 [reference]	..
		[0.7,1)	200	1.02 (0.86 - 1.21)	0.810
		[1,1.1)	389	0.97 (0.84 - 1.12)	0.682
		[1.1,2)	406	1.09 (0.95 - 1.26)	0.224
		[2,2.9)	316	1.25 (1.08 - 1.45)	0.004
		[2.9,3)	487	1.04 (0.91 - 1.19)	0.585
		[3,3.1)	362	1.01 (0.87 - 1.16)	0.911
		[3.1,4)	188	1.28 (1.07 - 1.53)	0.006
General medical conditions	40-59	[4,4.1)	890	1.04 (0.92 - 1.17)	0.563
		[4.1,6)	314	1.25 (1.07 - 1.45)	0.004
		[6,8)	263	1.44 (1.23 - 1.68)	<0.001
		[8,10)	170	1.47 (1.23 - 1.76)	<0.001
		[10,15)	243	1.47 (1.25 - 1.73)	<0.001
		[15,30)	300	1.47 (1.27 - 1.72)	<0.001
		[30,50)	197	1.56 (1.31 - 1.85)	<0.001
		[50,90)	211	1.53 (1.29 - 1.81)	<0.001
		[90,165)	210	1.57 (1.33 - 1.86)	<0.001
		[165,678]	220	2.11 (1.79 - 2.50)	<0.001
		[0,0.7)	119	1.00 [reference]	..
		[0.7,1)	81	1.08 (0.81 - 1.43)	0.612
		[1,1.1)	168	1.37 (1.08 - 1.73)	0.009
		[1.1,2)	225	1.22 (0.98 - 1.53)	0.076
		[2,2.9)	153	1.37 (1.08 - 1.74)	0.009
		[2.9,3)	262	1.22 (0.98 - 1.52)	0.070
		[3,3.1)	166	1.19 (0.94 - 1.51)	0.149
		[3.1,4)	102	1.35 (1.04 - 1.76)	0.025

Outcome	Age group	Peak CRP value[b]	Cases, No.	HR (95% CI)[a]	p-value
General medical conditions	60-79	[4,4.1)	446	1.22 (1.00 - 1.50)	0.051
		[4.1,6)	174	1.33 (1.06 - 1.68)	0.016
		[6,8)	128	1.44 (1.12 - 1.84)	0.004
		[8,10)	105	1.79 (1.37 - 2.32)	<0.001
		[10,15)	149	1.73 (1.36 - 2.21)	<0.001
		[15,30)	226	1.76 (1.41 - 2.20)	<0.001
		[30,50)	193	2.08 (1.65 - 2.61)	<0.001
		[50,90)	215	2.03 (1.62 - 2.54)	<0.001
		[90,165)	282	2.23 (1.80 - 2.77)	<0.001
		[165,678]	258	2.38 (1.91 - 2.96)	<0.001
General medical conditions	≥80	[0,0.7)	≤5[b]	1.00 [reference]	..
		[0.7,1)	≤5[b]		
		[1,1.1)	14	1.76 (0.58 - 5.34)	0.320
		[1.1,2)	16	1.70 (0.57 - 5.08)	0.344
		[2,2.9)	11	1.41 (0.45 - 4.44)	0.552
		[2.9,3)	15	0.94 (0.31 - 2.82)	0.907
		[3,3.1)	13	1.08 (0.35 - 3.31)	0.896
		[3.1,4)	11	2.39 (0.76 - 7.51)	0.136
		[4,4.1)	29	1.05 (0.37 - 2.99)	0.928
		[4.1,6)	13	1.12 (0.36 - 3.43)	0.846
		[6,8)	11	1.64 (0.52 - 5.15)	0.397
		[8,10)	9	1.01 (0.31 - 3.28)	0.991
		[10,15)	28	1.84 (0.65 - 5.25)	0.254
		[15,30)	49	2.02 (0.73 - 5.61)	0.176
		[30,50)	54	2.71 (0.98 - 7.49)	0.055
		[50,90)	55	1.81 (0.65 - 5.00)	0.253
		[90,165)	69	2.16 (0.79 - 5.94)	0.134
		[165,678]	41	2.09 (0.75 - 5.85)	0.159

Abbreviations: HR, Hazard Ratio; CI, Confidence Interval.

[a] The estimates are HRs with 95% CI from Cox Proportional Hazards model stratified by age and adjusted for confounders (sex, Charlson Comorbidity Index (CCI), parental CCI, parental mental health, employment status, income, highest level of education). All statistical tests were two-sided without correction for multiple comparisons.
[b] Results from ≤5 patients are displayed as "≤5" to ensure data privacy.

Supplementary Figure 5: CRP value and age group.

Density of peak C-reactive Protein (CRP) and hazard ratios of any first mental disorder and general medical condition comparing levels of CRP within age groups among individuals with positive SARS-CoV-2 tests and CRP measurements[b] (n = 322,715).

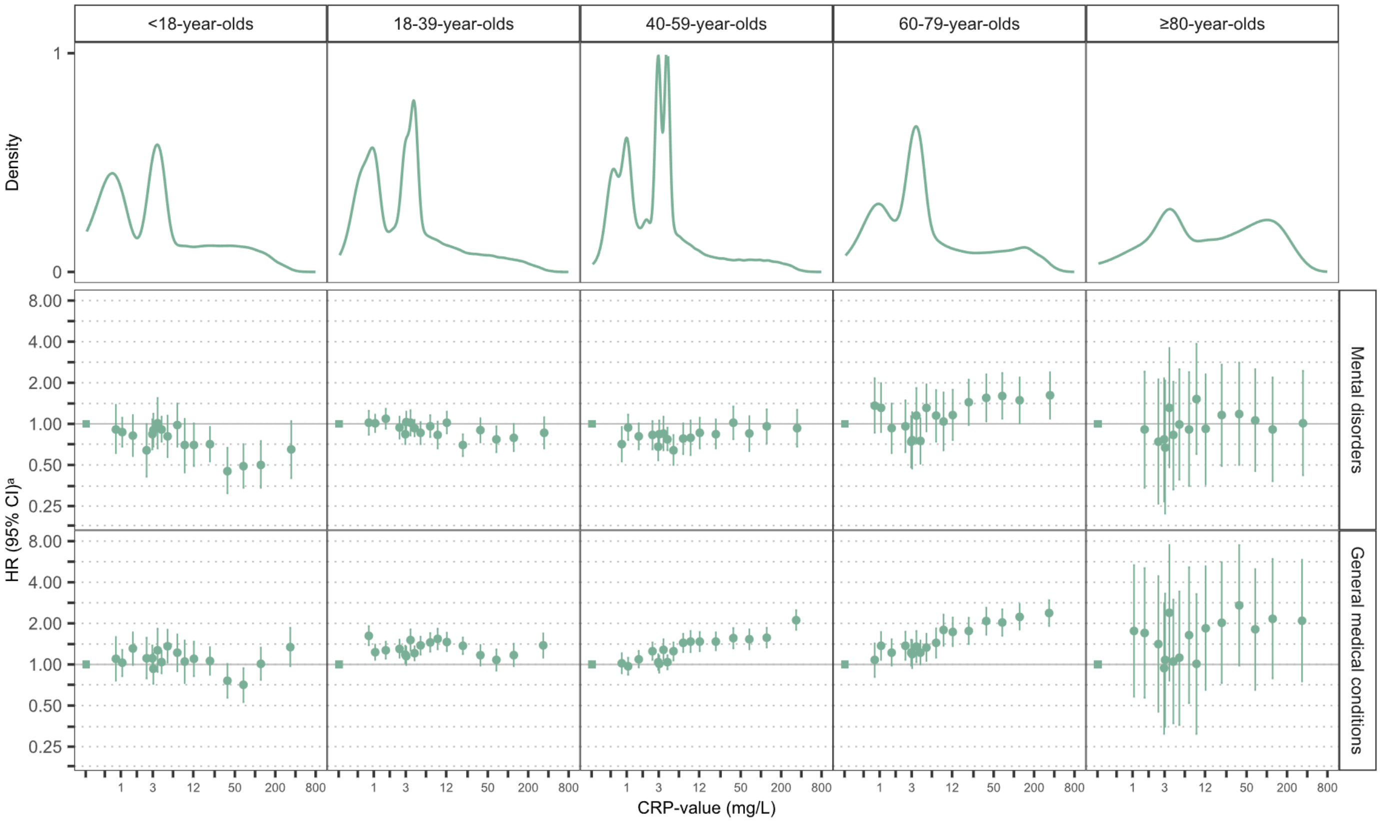

Abbreviations: HR, Hazard Rate Ratio; CI, Confidence Interval; CRP, C-Reactive Protein.

[a] The estimates are HRs with 95% CI from Cox Proportional Hazards model stratified by age and adjusted for confounders (sex, Charlson Comorbidity Index (CCI), parental CCI, parental psychiatric history, employment status, income, and highest level of education).

[b] Including individuals with a CRP measurement within 14 days before a positive SARS-CoV-2 test. The CRP groups were defined by dividing all individuals in 18 equally sized groups depending on case distribution. The reference group was the lowest CRP group marked with a green square in the plot.

Supplementary Table 22: CRP value and age group (unadjusted rates and rate ratios).

Raw, unadjusted rates and rate ratios of any first mental disorder and general medical condition by age group among individuals with a positive SARS-CoV-2 test and CRP measurements up to 14 days before the SARS-CoV-2 test.

a Raw rates and 95% CI.

		Peak CRP value				
**Outcome**	**Age group**	**<4 mg/L**	**4-10 mg/L**	**10-40 mg/L**	**40-100 mg/L**	**≥100 mg/L**
Mental disorders	<18	0.023 (0.021-0.025)	0.022 (0.019-0.025)	0.015 (0.012-0.018)	0.010 (0.008-0.014)	0.013 (0.010-0.018)
	18-29	0.024 (0.022-0.025)	0.023 (0.021-0.025)	0.022 (0.019-0.024)	0.019 (0.015-0.022)	0.021 (0.018-0.026)
	30-39	0.014 (0.013-0.016)	0.013 (0.012-0.015)	0.013 (0.011-0.016)	0.013 (0.010-0.017)	0.012 (0.009-0.017)
	40-49	0.009 (0.009-0.010)	0.010 (0.009-0.011)	0.013 (0.011-0.015)	0.012 (0.009-0.016)	0.011 (0.008-0.015)
	50-59	0.008 (0.007-0.009)	0.007 (0.006-0.008)	0.008 (0.007-0.010)	0.010 (0.008-0.013)	0.010 (0.008-0.013)
	60-69	0.005 (0.005-0.006)	0.005 (0.005-0.006)	0.008 (0.006-0.010)	0.009 (0.007-0.012)	0.008 (0.006-0.010)
	70-79	0.006 (0.005-0.007)	0.006 (0.005-0.007)	0.009 (0.007-0.011)	0.011 (0.009-0.014)	0.011 (0.009-0.013)
	≥80	0.008 (0.006-0.010)	0.009 (0.007-0.012)	0.011 (0.009-0.014)	0.009 (0.007-0.012)	0.009 (0.007-0.012)
General medical conditions	<18	0.043 (0.040-0.046)	0.045 (0.040-0.050)	0.040 (0.035-0.046)	0.028 (0.023-0.034)	0.047 (0.039-0.057)
	18-29	0.043 (0.040-0.045)	0.045 (0.042-0.048)	0.048 (0.044-0.053)	0.035 (0.030-0.041)	0.044 (0.037-0.052)
	30-39	0.043 (0.041-0.046)	0.047 (0.044-0.051)	0.052 (0.046-0.058)	0.043 (0.036-0.051)	0.048 (0.040-0.058)
	40-49	0.050 (0.048-0.053)	0.054 (0.050-0.058)	0.076 (0.068-0.085)	0.07 (0.059-0.083)	0.088 (0.075-0.103)
	50-59	0.063 (0.059-0.066)	0.072 (0.067-0.077)	0.096 (0.086-0.107)	0.103 (0.089-0.118)	0.123 (0.108-0.139)
	60-69	0.090 (0.084-0.097)	0.098 (0.090-0.106)	0.148 (0.132-0.167)	0.164 (0.142-0.190)	0.194 (0.171-0.219)
	70-79	0.143 (0.130-0.157)	0.158 (0.142-0.177)	0.211 (0.184-0.242)	0.238 (0.204-0.279)	0.276 (0.243-0.313)
	≥80	0.222 (0.180-0.273)	0.192 (0.149-0.246)	0.386 (0.320-0.465)	0.373 (0.304-0.458)	0.367 (0.300-0.449)

B Raw rate ratios and 95% CI with reference group having peak CRP <4 mg/L.

		Peak CRP value				
**Outcome**	**Age group**	**<4 mg/L**	**4-10 mg/L**	**10-40 mg/L**	**40-100 mg/L**	**≥100 mg/L**
Mental disorders	<18	1.00 [reference]	0.97 (0.84-1.12)	0.65 (0.54-0.80)	0.46 (0.35-0.62)	0.60 (0.43-0.82)
	18-29	1.00 [reference]	0.97 (0.88-1.07)	0.91 (0.80-1.04)	0.78 (0.65-0.95)	0.90 (0.73-1.12)
	30-39	1.00 [reference]	0.92 (0.81-1.05)	0.93 (0.78-1.11)	0.90 (0.69-1.17)	0.85 (0.63-1.16)
	40-49	1.00 [reference]	1.01 (0.88-1.17)	1.34 (1.10-1.62)	1.31 (0.99-1.73)	1.20 (0.88-1.64)
	50-59	1.00 [reference]	0.86 (0.74-1.01)	1.04 (0.84-1.30)	1.26 (0.96-1.66)	1.24 (0.95-1.62)
	60-69	1.00 [reference]	0.99 (0.80-1.23)	1.45 (1.12-1.87)	1.64 (1.21-2.22)	1.48 (1.10-1.99)
	70-79	1.00 [reference]	1.05 (0.81-1.36)	1.54 (1.18-2.01)	2.02 (1.53-2.66)	1.88 (1.44-2.46)
	≥80	1.00 [reference]	1.23 (0.86-1.74)	1.45 (1.04-2.03)	1.25 (0.86-1.81)	1.22 (0.84-1.77)
General medical conditions	<18	1.00 [reference]	1.05 (0.92-1.19)	0.94 (0.81-1.10)	0.64 (0.52-0.80)	1.11 (0.90-1.36)
	18-29	1.00 [reference]	1.06 (0.98-1.16)	1.13 (1.01-1.26)	0.83 (0.70-0.98)	1.03 (0.86-1.23)
	30-39	1.00 [reference]	1.10 (1.00-1.20)	1.20 (1.06-1.36)	1.01 (0.84-1.21)	1.11 (0.91-1.36)
	40-49	1.00 [reference]	1.08 (0.98-1.17)	1.52 (1.35-1.72)	1.40 (1.17-1.66)	1.76 (1.49-2.08)
	50-59	1.00 [reference]	1.15 (1.05-1.25)	1.53 (1.36-1.73)	1.64 (1.40-1.91)	1.95 (1.70-2.24)
	60-69	1.00 [reference]	1.08 (0.97-1.21)	1.64 (1.43-1.88)	1.82 (1.55-2.13)	2.15 (1.87-2.47)
	70-79	1.00 [reference]	1.11 (0.96-1.28)	1.48 (1.25-1.74)	1.67 (1.39-2.00)	1.93 (1.65-2.26)
	≥80	1.00 [reference]	0.86 (0.62-1.19)	1.74 (1.31-2.30)	1.68 (1.25-2.25)	1.65 (1.24-2.21)

Abbreviations: RR, Rate Ratio; CI, Confidence Interval; CRP, C-Reactive Protein.

Supplementary Figure 6: CRP value and age group (unadjusted rates and rate ratios).

Raw, unadjusted rates and rate ratios of any first mental disorder and general medical condition by age group among individuals with a positive SARS-CoV-2 test and CRP measurements up to 14 days before the SARS-CoV-2 test (n = 322,715).

a Raw rates and 95% CI.

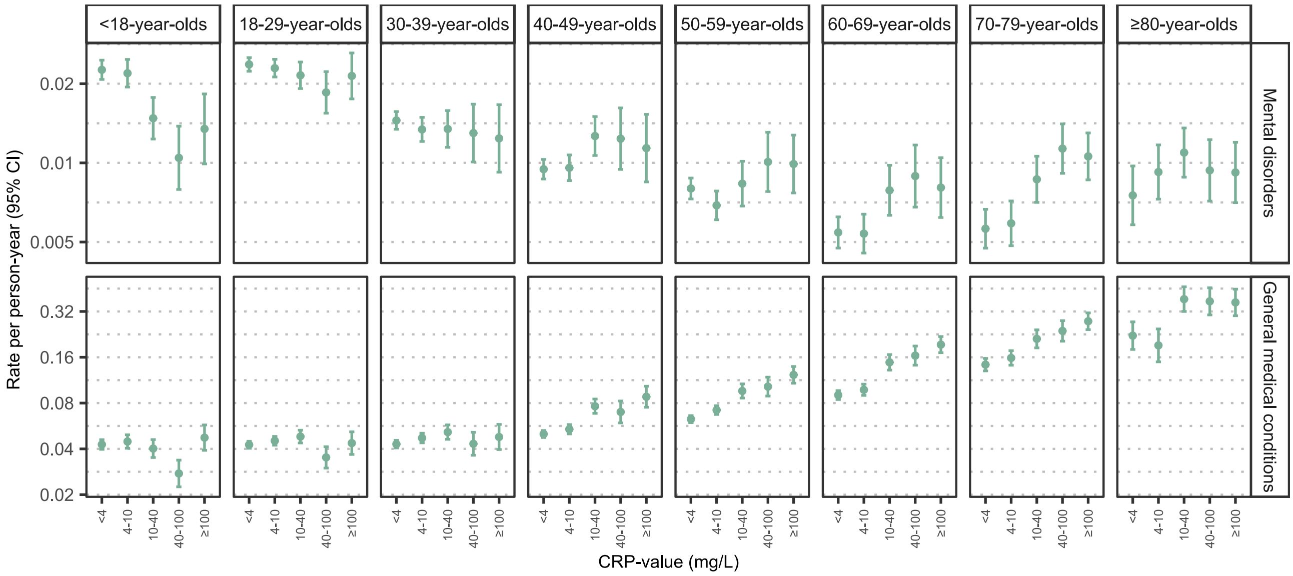

b Raw rate ratios and 95% CI with reference group having peak CRP <4 mg/L.

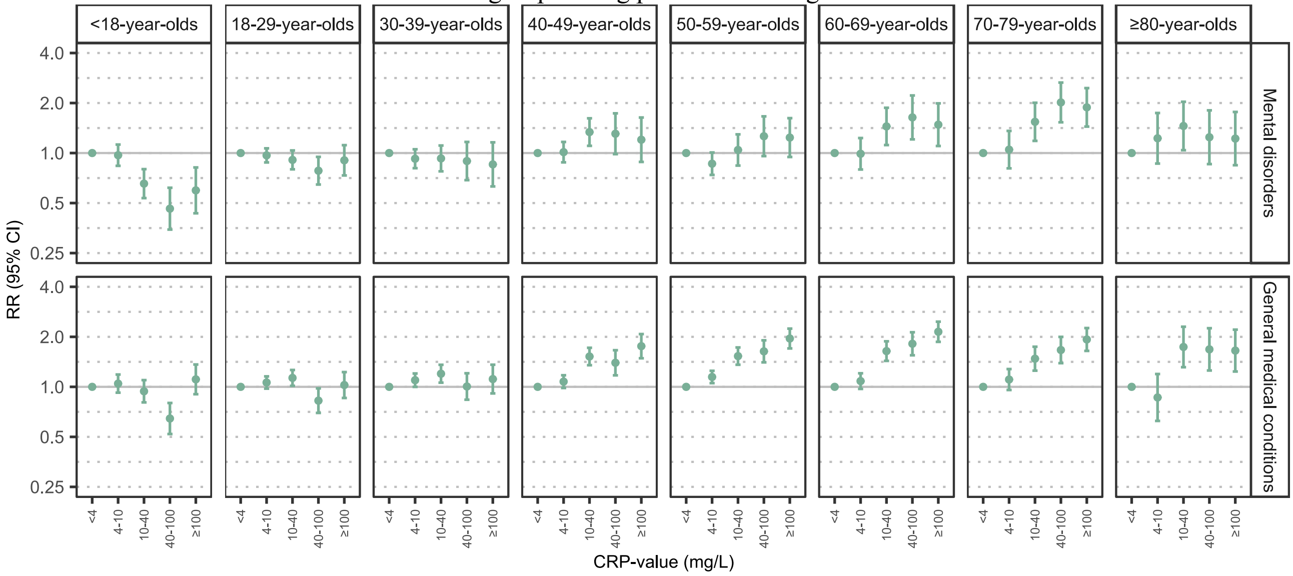

Abbreviations: RR, Rate Ratio; CI, Confidence Interval; CRP, C-Reactive Protein.

Results: Admission with COVID-19 and peak C-Reactive Protein (CRP)
Supplementary Table 23: CRP value.

Hazard ratios of specific first mental health or general medical disorders among individuals with admission with COVID-19 by peak CRP value.

Outcome	Peak CRP value[b]	Cases, No.	HR (95% CI)[a]	p-value
Mental disorders or general medical conditions	<4 mg/L	83	1.00 [reference]	..
	4-10 mg/L	112	0.95 (0.72 - 1.27)	0.736
	10-40 mg/L	225	0.80 (0.62 - 1.04)	0.091
	40-100 mg/L	289	0.73 (0.56 - 0.93)	0.012
	≥100 mg/L	517	0.86 (0.68 - 1.10)	0.225
Mental disorders	<4 mg/L	59	1.00 [reference]	..
	4-10 mg/L	90	1.03 (0.74 - 1.43)	0.873
	10-40 mg/L	158	0.84 (0.62 - 1.13)	0.244
	40-100 mg/L	172	0.74 (0.55 - 1.00)	0.048
	≥100 mg/L	239	0.72 (0.54 - 0.97)	0.028
General medical conditions	<4 mg/L	102	1.00 [reference]	..
	4-10 mg/L	123	0.92 (0.71 - 1.20)	0.549
	10-40 mg/L	235	0.83 (0.65 - 1.04)	0.111
	40-100 mg/L	308	0.80 (0.64 - 1.01)	0.059
	≥100 mg/L	534	0.91 (0.73 - 1.13)	0.395
Neurological disorders	<4 mg/L	126	1.00 [reference]	..
	4-10 mg/L	150	0.84 (0.67 - 1.07)	0.161
	10-40 mg/L	324	0.80 (0.65 - 0.99)	0.041
	40-100 mg/L	410	0.73 (0.59 - 0.89)	0.002
	≥100 mg/L	648	0.72 (0.59 - 0.88)	0.001
Respiratory disorders	<4 mg/L	46	1.00 [reference]	..
	4-10 mg/L	88	1.25 (0.87 - 1.78)	0.225
	10-40 mg/L	250	1.51 (1.10 - 2.07)	0.011
	40-100 mg/L	354	1.58 (1.16 - 2.16)	0.004
	≥100 mg/L	673	1.98 (1.46 - 2.68)	<0.001
Circulatory disorders	<4 mg/L	66	1.00 [reference]	..
	4-10 mg/L	122	1.32 (0.97 - 1.78)	0.073
	10-40 mg/L	252	1.18 (0.89 - 1.54)	0.246
	40-100 mg/L	389	1.26 (0.96 - 1.64)	0.092
	≥100 mg/L	684	1.46 (1.13 - 1.89)	0.004
Kidney disorders	<4 mg/L	29	1.00 [reference]	..
	4-10 mg/L	47	0.99 (0.63 - 1.58)	0.978
	10-40 mg/L	140	1.16 (0.78 - 1.73)	0.469
	40-100 mg/L	212	1.24 (0.84 - 1.83)	0.289
	≥100 mg/L	432	1.66 (1.14 - 2.43)	0.009
Gastrointestinal disorders	<4 mg/L	47	1.00 [reference]	..
	4-10 mg/L	61	0.88 (0.60 - 1.29)	0.519
	10-40 mg/L	160	1.05 (0.75 - 1.45)	0.792
	40-100 mg/L	194	0.95 (0.69 - 1.32)	0.773
	≥100 mg/L	274	0.89 (0.65 - 1.22)	0.476
Endocrine disorders	<4 mg/L	12	1.00 [reference]	..
	4-10 mg/L	38	2.14 (1.12 - 4.09)	0.022
	10-40 mg/L	103	2.45 (1.35 - 4.47)	0.003
	40-100 mg/L	155	2.66 (1.47 - 4.80)	0.001
	≥100 mg/L	261	3.05 (1.70 - 5.47)	<0.001
Hematological disorders	<4 mg/L	35	1.00 [reference]	..
	4-10 mg/L	82	1.46 (0.99 - 2.18)	0.059
	10-40 mg/L	197	1.42 (0.99 - 2.04)	0.058
	40-100 mg/L	283	1.48 (1.04 - 2.11)	0.031
	≥100 mg/L	432	1.51 (1.06 - 2.14)	0.021
Musculoskeletal disorders	<4 mg/L	17	1.00 [reference]	..
	4-10 mg/L	25	1.07 (0.58 - 1.98)	0.837
	10-40 mg/L	36	0.76 (0.42 - 1.35)	0.347
	40-100 mg/L	42	0.74 (0.42 - 1.32)	0.308
	≥100 mg/L	74	0.90 (0.53 - 1.55)	0.714

Outcome	Peak CRP value[b]	Cases, No.	HR (95% CI)[a]	p-value
Dermatological disorders	<4 mg/L	15	1.00 [reference]	..
	4-10 mg/L	13	0.68 (0.32 - 1.42)	0.303
	10-40 mg/L	25	0.72 (0.38 - 1.38)	0.328
	40-100 mg/L	48	1.23 (0.67 - 2.23)	0.506
	≥100 mg/L	60	1.10 (0.61 - 1.98)	0.759
Neuropsychiatric symptoms	<4 mg/L	93	1.00 [reference]	..
	4-10 mg/L	146	0.99 (0.76 - 1.28)	0.911
	10-40 mg/L	273	0.78 (0.61 - 0.98)	0.036
	40-100 mg/L	425	0.89 (0.71 - 1.12)	0.315
	≥100 mg/L	564	0.77 (0.61 - 0.96)	0.021

Abbreviations: HR, Hazard Ratio; CI, Confidence Interval*; CRP, C-Reactive Protein.*

[a] The estimates are HRs with 95% CI from Cox Proportional Hazards model stratified by age and adjusted for confounders (sex, Charlson Comorbidity Index (CCI), parental CCI, parental mental health, employment status, income, highest level of education). All statistical tests were two-sided without correction for multiple comparisons.

[b] The reference group *<4 mg/L* consisted of individuals admission to a hospital with COVID-19 and peak CRP measurement <4 mg/L.

Supplementary Table 24: CRP value in quantiles

Hazard ratios of specific first mental health or general medical disorders among individuals hospitalized with COVID-19 by peak CRP value in quantiles.

Outcome	Peak CRP value[b]	Cases, No.	HR (95% CI)[a]	p-value
	[0,3)	66	1.00 [reference]	..
	[3,4.75)	57	0.80 (0.56 - 1.15)	0.226
	[4.75,8.78)	61	1.05 (0.74 - 1.50)	0.765
	[8.78,15)	60	0.90 (0.63 - 1.28)	0.549
	[15,22)	60	0.72 (0.50 - 1.02)	0.065
	[22,30)	62	0.88 (0.62 - 1.25)	0.480
	[30,41)	62	0.64 (0.45 - 0.91)	0.013
	[41,51)	62	0.68 (0.48 - 0.97)	0.033
Mental disorders and general medical conditions	[51,64)	58	0.62 (0.43 - 0.89)	0.009
	[64,77)	64	0.71 (0.50 - 1.00)	0.053
	[77,89)	60	0.80 (0.56 - 1.15)	0.229
	[89,107)	63	0.51 (0.36 - 0.73)	<0.001
	[107,120)	57	0.85 (0.59 - 1.22)	0.385
	[120,137)	63	0.73 (0.51 - 1.04)	0.080
	[137,157)	64	0.67 (0.47 - 0.95)	0.024
	[157,179)	60	0.76 (0.53 - 1.08)	0.128
	[179,205)	63	0.98 (0.69 - 1.40)	0.931
	[205,246)	61	0.82 (0.58 - 1.18)	0.286
	[246,297)	61	1.10 (0.77 - 1.57)	0.599
	[297,770]	62	1.12 (0.78 - 1.59)	0.546
	[0,4)	59	1.00 [reference]	..
	[4,9)	82	1.04 (0.75 - 1.46)	0.808
	[9,19.2)	75	0.88 (0.62 - 1.23)	0.446
	[19.2,31.8)	71	1.00 (0.70 - 1.41)	0.991
Mental disorders	[31.8,50)	71	0.77 (0.54 - 1.09)	0.136
	[50,76)	72	0.68 (0.48 - 0.97)	0.033
	[76,110)	66	0.62 (0.43 - 0.88)	0.008
	[110,147)	77	0.77 (0.55 - 1.09)	0.142
	[147,204)	73	0.76 (0.54 - 1.08)	0.128
	[204,770]	72	0.67 (0.47 - 0.95)	0.025
	[0,4)	102	1.00 [reference]	..
	[4,14)	156	0.91 (0.71 - 1.17)	0.470
	[14,29)	128	0.84 (0.64 - 1.09)	0.188
	[29,48.3)	135	0.78 (0.60 - 1.02)	0.067
General medical conditions	[48.3,73)	128	0.78 (0.59 - 1.01)	0.061
	[73,101)	131	0.80 (0.61 - 1.04)	0.095
	[101,135)	131	0.75 (0.58 - 0.98)	0.037
	[135,177)	130	0.82 (0.62 - 1.07)	0.137
	[177,247)	130	0.99 (0.76 - 1.29)	0.919
	[247,770]	131	1.20 (0.92 - 1.56)	0.184
	[0,4.17)	166	1.00 [reference]	..
	[4.17,14)	162	0.95 (0.77 - 1.18)	0.666
	[14,29)	165	0.86 (0.69 - 1.07)	0.171
	[29,46)	162	0.92 (0.74 - 1.15)	0.469
Neurological disorders	[46,66.9)	174	0.85 (0.69 - 1.06)	0.151
	[66.9,97)	165	0.74 (0.60 - 0.92)	0.007
	[97,130)	159	0.70 (0.56 - 0.88)	0.002
	[130,171)	173	0.74 (0.59 - 0.92)	0.006
	[171,240)	161	0.82 (0.66 - 1.02)	0.079
	[240,770]	171	0.95 (0.77 - 1.19)	0.679
	[0,11)	140	1.00 [reference]	..
	[11,25)	142	1.44 (1.14 - 1.82)	0.002
	[25,46)	139	1.18 (0.93 - 1.49)	0.176
	[46,67)	143	1.42 (1.12 - 1.80)	0.004
Respiratory disorders	[67,92.9)	141	1.35 (1.07 - 1.72)	0.012
	[92.9,120)	142	1.52 (1.20 - 1.93)	0.001
	[120,150)	128	1.44 (1.13 - 1.84)	0.003

Outcome	Peak CRP value[b]	Cases, No.	HR (95% CI)[a]	p-value
	[150,191)	153	1.61 (1.27 - 2.03)	<0.001
	[191,255)	141	1.85 (1.45 - 2.34)	<0.001
	[255,770]	142	2.21 (1.75 - 2.80)	<0.001
Circulatory disorders	[0,7.12)	152	1.00 [reference]	..
	[7.12,22.1)	150	1.01 (0.81 - 1.27)	0.911
	[22.1,41)	151	1.12 (0.89 - 1.41)	0.318
	[41,61)	146	1.05 (0.83 - 1.32)	0.697
	[61,85)	154	1.13 (0.90 - 1.41)	0.309
	[85,114)	155	1.01 (0.80 - 1.27)	0.941
	[114,148)	151	1.07 (0.85 - 1.35)	0.565
	[148,190)	143	1.15 (0.91 - 1.45)	0.235
	[190,260)	159	1.35 (1.08 - 1.69)	0.010
	[260,770]	152	1.80 (1.43 - 2.26)	<0.001
Kidney disorders	[0,11)	81	1.00 [reference]	..
	[11,29)	89	1.12 (0.83 - 1.51)	0.469
	[29,49)	86	1.18 (0.87 - 1.61)	0.277
	[49,71.1)	88	1.22 (0.90 - 1.66)	0.198
	[71.1,100)	84	1.18 (0.86 - 1.60)	0.305
	[100,127)	88	1.40 (1.03 - 1.90)	0.032
	[127,162)	86	1.24 (0.91 - 1.68)	0.175
	[162,204)	86	1.64 (1.20 - 2.23)	0.002
	[204,297)	86	1.46 (1.07 - 1.98)	0.016
	[297,770]	86	3.65 (2.68 - 4.97)	<0.001
Gastrointestinal disorders	[0,5.45)	74	1.00 [reference]	..
	[5.45,14.5)	73	1.17 (0.85 - 1.62)	0.334
	[14.5,29.5)	74	1.05 (0.76 - 1.45)	0.785
	[29.5,46)	68	1.05 (0.75 - 1.47)	0.762
	[46,63.3)	79	1.24 (0.90 - 1.72)	0.184
	[63.3,91.1)	74	0.90 (0.65 - 1.26)	0.546
	[91.1,120)	73	1.10 (0.79 - 1.53)	0.559
	[120,160)	73	0.88 (0.63 - 1.23)	0.447
	[160,214)	74	1.02 (0.73 - 1.41)	0.929
	[214,770]	74	0.92 (0.66 - 1.28)	0.631
Endocrine disorders	[0,12.4)	56	1.00 [reference]	..
	[12.4,25)	56	1.83 (1.26 - 2.65)	0.001
	[25,44)	57	1.42 (0.98 - 2.06)	0.065
	[44,67)	58	1.36 (0.94 - 1.97)	0.101
	[67,88)	55	1.66 (1.14 - 2.42)	0.008
	[88,115)	57	1.53 (1.05 - 2.22)	0.026
	[115,150)	57	1.49 (1.02 - 2.17)	0.037
	[150,186)	59	1.94 (1.34 - 2.81)	<0.001
	[186,251)	57	1.81 (1.24 - 2.63)	0.002
	[251,770]	57	2.08 (1.43 - 3.02)	<0.001
Hematological disorders	[0,8.82)	103	1.00 [reference]	..
	[8.82,22)	99	1.14 (0.87 - 1.51)	0.345
	[22,38)	104	1.24 (0.94 - 1.63)	0.127
	[38,56)	104	1.20 (0.91 - 1.58)	0.187
	[56,79)	102	1.11 (0.84 - 1.47)	0.443
	[79,108)	105	1.13 (0.85 - 1.48)	0.400
	[108,139)	100	1.15 (0.87 - 1.52)	0.322
	[139,177)	106	1.15 (0.88 - 1.52)	0.310
	[177,247)	103	1.18 (0.89 - 1.56)	0.243
	[247,770]	103	1.55 (1.17 - 2.04)	0.002
Musculoskeletal disorders	[0,4)	17	1.00 [reference]	..
	[4,8.26)	22	1.12 (0.59 - 2.11)	0.725
	[8.26,19.9)	19	0.81 (0.42 - 1.56)	0.529
	[19.9,38.7)	20	0.74 (0.39 - 1.43)	0.375
	[38.7,56.5)	19	0.88 (0.46 - 1.71)	0.711
	[56.5,85.8)	19	0.72 (0.37 - 1.40)	0.333
	[85.8,126)	20	0.68 (0.35 - 1.32)	0.254
	[126,157)	19	1.00 (0.51 - 1.97)	0.990
	[157,219)	19	0.85 (0.44 - 1.66)	0.642

Outcome	Peak CRP value[b]	Cases, No.	HR (95% CI)[a]	p-value
	[219,770]	20	0.91 (0.47 - 1.76)	0.782
Dermatological disorders	[0,4)	15	1.00 [reference]	..
	[4,16)	17	0.58 (0.29 - 1.17)	0.128
	[16,37.2)	17	0.74 (0.37 - 1.49)	0.401
	[37.2,54)	15	1.12 (0.54 - 2.32)	0.758
	[54,78.9)	16	0.97 (0.47 - 1.99)	0.931
	[78.9,94)	17	2.00 (0.98 - 4.08)	0.056
	[94,120)	16	1.19 (0.58 - 2.46)	0.639
	[120,157)	15	1.15 (0.55 - 2.41)	0.704
	[157,225)	16	0.96 (0.46 - 1.98)	0.910
	[225,770]	17	1.21 (0.59 - 2.48)	0.596
Neuropsychiatric symptoms	[0,5)	146	1.00 [reference]	..
	[5,14)	145	1.02 (0.81 - 1.29)	0.842
	[14,31)	156	0.79 (0.63 - 0.99)	0.044
	[31,50)	152	0.83 (0.66 - 1.04)	0.104
	[50,68.7)	151	0.96 (0.76 - 1.21)	0.738
	[68.7,91.1)	151	0.93 (0.74 - 1.17)	0.540
	[91.1,124)	146	0.74 (0.58 - 0.93)	0.011
	[124,164)	153	0.77 (0.61 - 0.97)	0.030
	[164,224)	148	0.81 (0.64 - 1.02)	0.076
	[224,770]	153	0.86 (0.68 - 1.08)	0.195

Abbreviations: HR, Hazard Ratio; CI, Confidence Interval; CRP, C-Reactive Protein.

[a] The estimates are HRs with 95% CI from Cox Proportional Hazards model stratified by age and adjusted for confounders (sex, Charlson Comorbidity Index (CCI), parental CCI, parental mental health, employment status, income, highest level of education). All statistical tests were two-sided without correction for multiple comparisons.

Supplementary Table 25: CRP value and age group.

Hazard ratios of specific first mental health or general medical disorders among individuals hospitalized with COVID-19 by peak C-Reactive Protein (CRP) value in quantiles and by age group.

Outcome	Age group	Peak CRP value[b]	Cases, No.	HR (95% CI)[a]	p-value
		[0,3)	36	1.00 [reference]	..
		[3,4.75)	30	0.71 (0.44 - 1.16)	0.169
		[4.75,8.78)	24	0.81 (0.48 - 1.36)	0.424
		[8.78,15)	24	0.76 (0.45 - 1.28)	0.299
		[15,22)	20	0.64 (0.37 - 1.11)	0.111
		[22,30)	21	0.86 (0.50 - 1.47)	0.579
		[30,41)	14	0.42 (0.22 - 0.78)	0.006
		[41,51)	8	0.32 (0.15 - 0.68)	0.003
		[51,64)	7	0.29 (0.13 - 0.66)	0.003
Mental disorders and general medical conditions	<40	[64,77)	12	0.58 (0.30 - 1.12)	0.104
		[77,89)	12	0.61 (0.31 - 1.17)	0.137
		[89,107)	9	0.33 (0.16 - 0.69)	0.003
		[107,120)	14	1.22 (0.66 - 2.28)	0.529
		[120,137)	8	0.44 (0.20 - 0.94)	0.035
		[137,157)	11	0.74 (0.37 - 1.45)	0.375
		[157,179)	6	0.41 (0.17 - 0.98)	0.045
		[179,205)	8	0.71 (0.33 - 1.53)	0.386
		[205,246)	8	0.53 (0.24 - 1.14)	0.102
		[246,297)	10	0.67 (0.33 - 1.36)	0.267
		[297,770]	6	0.51 (0.22 - 1.22)	0.131
		[0,3)	15	1.00 [reference]	..
		[3,4.75)	18	1.25 (0.63 - 2.48)	0.529
		[4.75,8.78)	19	1.55 (0.79 - 3.07)	0.205
		[8.78,15)	16	0.97 (0.48 - 1.97)	0.931
		[15,22)	23	1.24 (0.65 - 2.39)	0.513
		[22,30)	17	0.89 (0.44 - 1.79)	0.748
		[30,41)	15	0.62 (0.30 - 1.27)	0.189
		[41,51)	23	0.96 (0.50 - 1.85)	0.910
		[51,64)	17	0.78 (0.39 - 1.56)	0.476
Mental disorders and general medical conditions	40-59	[64,77)	23	0.87 (0.45 - 1.67)	0.674
		[77,89)	17	1.14 (0.57 - 2.29)	0.713
		[89,107)	17	0.60 (0.30 - 1.20)	0.149
		[107,120)	15	0.97 (0.47 - 1.99)	0.939
		[120,137)	18	0.87 (0.44 - 1.74)	0.700
		[137,157)	21	0.92 (0.47 - 1.81)	0.819
		[157,179)	18	0.92 (0.46 - 1.84)	0.823
		[179,205)	16	0.81 (0.40 - 1.65)	0.566
		[205,246)	18	0.91 (0.46 - 1.82)	0.792
		[246,297)	14	1.32 (0.64 - 2.75)	0.455
		[297,770]	22	2.08 (1.07 - 4.03)	0.030
		[0,3)	15	1.00 [reference]	..
		[3,4.75)	9	0.60 (0.26 - 1.37)	0.223
		[4.75,8.78)	18	1.22 (0.61 - 2.42)	0.574
		[8.78,15)	20	1.24 (0.63 - 2.43)	0.532
		[15,22)	17	0.65 (0.32 - 1.31)	0.230
		[22,30)	24	1.19 (0.62 - 2.27)	0.600
		[30,41)	33	1.10 (0.59 - 2.03)	0.765
		[41,51)	31	1.04 (0.56 - 1.94)	0.900
		[51,64)	34	0.95 (0.51 - 1.75)	0.867
Mental disorders and general medical conditions	≥60	[64,77)	29	0.96 (0.51 - 1.81)	0.910
		[77,89)	31	1.08 (0.58 - 2.02)	0.798
		[89,107)	37	0.78 (0.42 - 1.43)	0.419
		[107,120)	28	1.00 (0.53 - 1.89)	0.989
		[120,137)	37	1.09 (0.59 - 2.00)	0.780
		[137,157)	32	0.80 (0.43 - 1.49)	0.489
		[157,179)	36	1.17 (0.64 - 2.15)	0.616
		[179,205)	39	1.78 (0.97 - 3.24)	0.061

Outcome	Age group	Peak CRP value[b]	Cases, No.	HR (95% CI)[a]	p-value
		[205,246)	35	1.27 (0.69 - 2.33)	0.447
		[246,297)	37	1.70 (0.93 - 3.11)	0.087
		[297,770]	34	1.31 (0.71 - 2.41)	0.392
Mental disorders	<60	[0,4)	43	1.00 [reference]	..
		[4,9)	57	1.07 (0.72 - 1.59)	0.744
		[9,19.2)	47	0.92 (0.61 - 1.40)	0.702
		[19.2,31.8)	32	0.86 (0.54 - 1.36)	0.526
		[31.8,50)	30	0.61 (0.38 - 0.98)	0.042
		[50,76)	31	0.62 (0.39 - 0.99)	0.047
		[76,110)	24	0.49 (0.30 - 0.81)	0.006
		[110,147)	27	0.66 (0.41 - 1.07)	0.093
		[147,204)	22	0.55 (0.32 - 0.92)	0.022
		[204,770]	31	0.64 (0.40 - 1.02)	0.063
Mental disorders	≥60	[0,4)	16	1.00 [reference]	..
		[4,9)	25	1.02 (0.55 - 1.92)	0.945
		[9,19.2)	28	0.86 (0.47 - 1.60)	0.640
		[19.2,31.8)	39	1.25 (0.70 - 2.23)	0.458
		[31.8,50)	41	1.00 (0.56 - 1.78)	0.987
		[50,76)	41	0.79 (0.44 - 1.42)	0.434
		[76,110)	42	0.77 (0.43 - 1.38)	0.383
		[110,147)	50	0.91 (0.52 - 1.61)	0.756
		[147,204)	51	0.97 (0.55 - 1.71)	0.917
		[204,770]	41	0.73 (0.41 - 1.30)	0.286
General medical conditions	<60	[0,4)	82	1.00 [reference]	..
		[4,14)	113	0.84 (0.63 - 1.12)	0.226
		[14,29)	81	0.85 (0.62 - 1.15)	0.287
		[29,48.3)	64	0.61 (0.44 - 0.85)	0.003
		[48.3,73)	66	0.74 (0.53 - 1.03)	0.075
		[73,101)	59	0.70 (0.50 - 0.98)	0.040
		[101,135)	56	0.74 (0.53 - 1.05)	0.090
		[135,177)	56	0.84 (0.59 - 1.18)	0.316
		[177,247)	56	0.86 (0.61 - 1.21)	0.384
		[247,770]	57	1.20 (0.85 - 1.69)	0.290
General medical conditions	≥60	[0,4)	20	1.00 [reference]	..
		[4,14)	43	1.15 (0.68 - 1.96)	0.599
		[14,29)	47	1.02 (0.61 - 1.73)	0.928
		[29,48.3)	71	1.33 (0.81 - 2.20)	0.260
		[48.3,73)	62	1.06 (0.63 - 1.75)	0.835
		[73,101)	72	1.19 (0.72 - 1.96)	0.501
		[101,135)	75	1.04 (0.63 - 1.71)	0.879
		[135,177)	74	1.11 (0.67 - 1.82)	0.690
		[177,247)	74	1.53 (0.93 - 2.51)	0.094
		[247,770]	74	1.63 (0.99 - 2.68)	0.055

Abbreviations: HR, Hazard Ratio; CI, Confidence Interval; CRP, C-Reactive Protein.

[a] The estimates are HRs with 95% CI from Cox Proportional Hazards model stratified by age and adjusted for confounders (sex, Charlson Comorbidity Index (CCI), parental CCI, parental mental health, employment status, income, highest level of education). All statistical tests were two-sided without correction for multiple comparisons.

Supplementary Figure 7: CRP value and age group.

Density of peak C-reactive Protein (CRP) and hazard ratios of any first mental disorder and general medical condition comparing levels of CRP within age groups among individuals admitted with COVID-19 and CRP measurements[b] (n = 5,810).

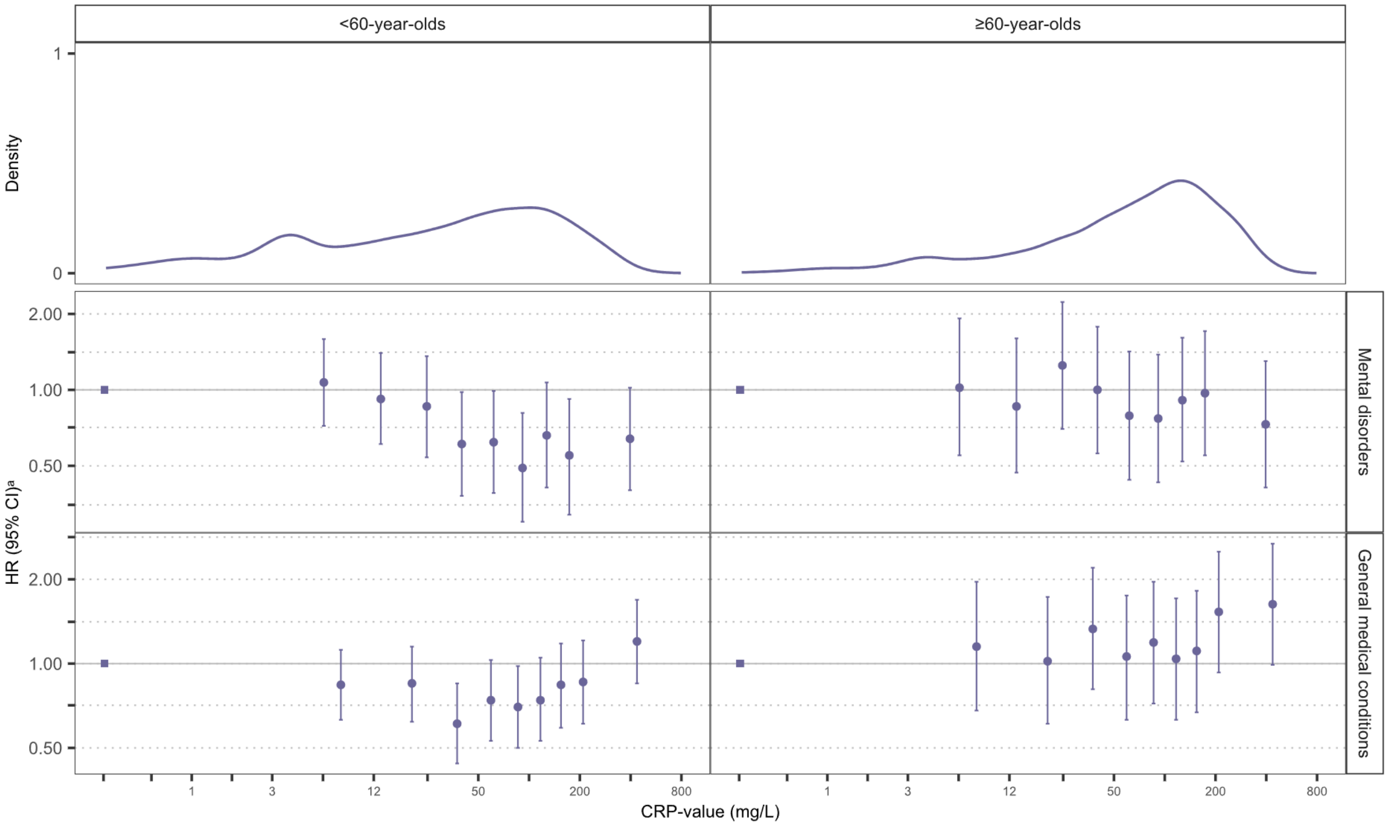

Abbreviations: HR, Hazard Rate Ratio; CI, Confidence Interval; CRP, C-Reactive Protein.

[a] The estimates are HRs with 95% CI from Cox Proportional Hazards model stratified by age and adjusted for confounders (sex, Charlson Comorbidity Index (CCI), parental CCI, parental psychiatric history, employment status, income, and highest level of education).
[b] Including individuals with a CRP measurement within 2 days before or during admission with COVID-19. The CRP groups were defined by dividing all individuals in 10 equally sized groups depending on case distribution. The reference group was the lowest CRP group marked with a purple square in the plot. The definition of admission to the hospital is summarized in Supplementary Table 30.

Supplementary Table 26: CRP and age group (unadjusted rates and rate ratios).

Raw, unadjusted rates and rate ratios of any first mental disorder and general medical condition by age group among individuals admitted with COVID-19 and with CRP measurements 2 days before or during admission with COVID-19.

a Raw rates and 95% CI.

		Peak CRP value				
**Outcome**	**Age group**	**<4 mg/L**	**4-10 mg/L**	**10-40 mg/L**	**40-100 mg/L**	**≥100 mg/L**
Mental disorders	<40	0.038 (0.025-0.051)	0.039 (0.027-0.051)	0.025 (0.018-0.032)	0.018 (0.011-0.024)	0.021 (0.014-0.028)
	40-59	0.013 (0.005-0.022)	0.017 (0.010-0.025)	0.016 (0.011-0.022)	0.016 (0.011-0.020)	0.012 (0.009-0.016)
	≥60	0.015 (0.008-0.023)	0.016 (0.010-0.022)	0.015 (0.012-0.019)	0.014 (0.011-0.016)	0.013 (0.011-0.015)
General medical conditions	<40	0.091 (0.067-0.115)	0.080 (0.059-0.100)	0.064 (0.049-0.080)	0.050 (0.035-0.064)	0.069 (0.053-0.085)
	40-59	0.121 (0.076-0.166)	0.122 (0.082-0.162)	0.112 (0.087-0.138)	0.117 (0.094-0.139)	0.128 (0.108-0.148)
	≥60	0.203 (0.114-0.292)	0.231 (0.148-0.314)	0.266 (0.213-0.320)	0.289 (0.244-0.333)	0.314 (0.279-0.349)

b Raw rate ratios and 95% CI with reference group having peak CRP <4 mg/L.

		Peak CRP value				
**Outcome**	**Age group**	**<4 mg/L**	**4-10 mg/L**	**10-40 mg/L**	**40-100 mg/L**	**≥100 mg/L**
Mental disorders	<40	1.00 [reference]	1.03 (0.66-1.63)	0.66 (0.42-1.04)	0.47 (0.28-0.78)	0.56 (0.35-0.90)
	40-59	1.00 [reference]	1.29 (0.60-2.77)	1.22 (0.60-2.45)	1.17 (0.59-2.33)	0.91 (0.46-1.80)
	≥60	1.00 [reference]	1.04 (0.56-1.93)	1.00 (0.58-1.72)	0.89 (0.53-1.51)	0.86 (0.51-1.43)
General medical conditions	<40	1.00 [reference]	0.88 (0.60-1.27)	0.71 (0.49-1.01)	0.55 (0.37-0.81)	0.76 (0.53-1.08)
	40-59	1.00 [reference]	1.01 (0.62-1.65)	0.93 (0.60-1.44)	0.96 (0.63-1.47)	1.06 (0.71-1.58)
	≥60	1.00 [reference]	1.14 (0.65-2.00)	1.31 (0.81-2.12)	1.42 (0.89-2.26)	1.54 (0.98-2.43)

Abbreviations: RR, Rate Ratio; CI, Confidence Interval; CRP, C-Reactive Protein.

Supplementary Figure 8: CRP value and age group (unadjusted rates and rate ratios).

Raw, unadjusted rates and rate ratios of any first mental disorder and general medical condition by age group among individuals admitted with COVID-19 and with CRP measurements 2 days before or during admission with COVID-19 (n = 5,810).

a Raw rates and 95% CI.

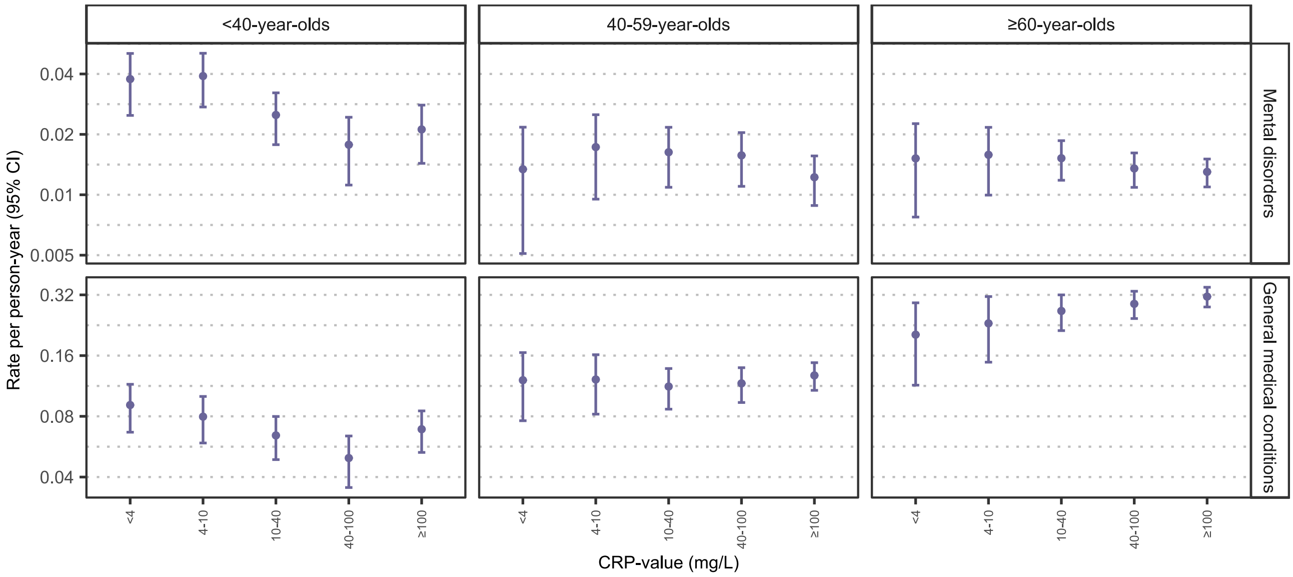

b Raw rate ratios with reference group having peak CRP <4 mg/L.

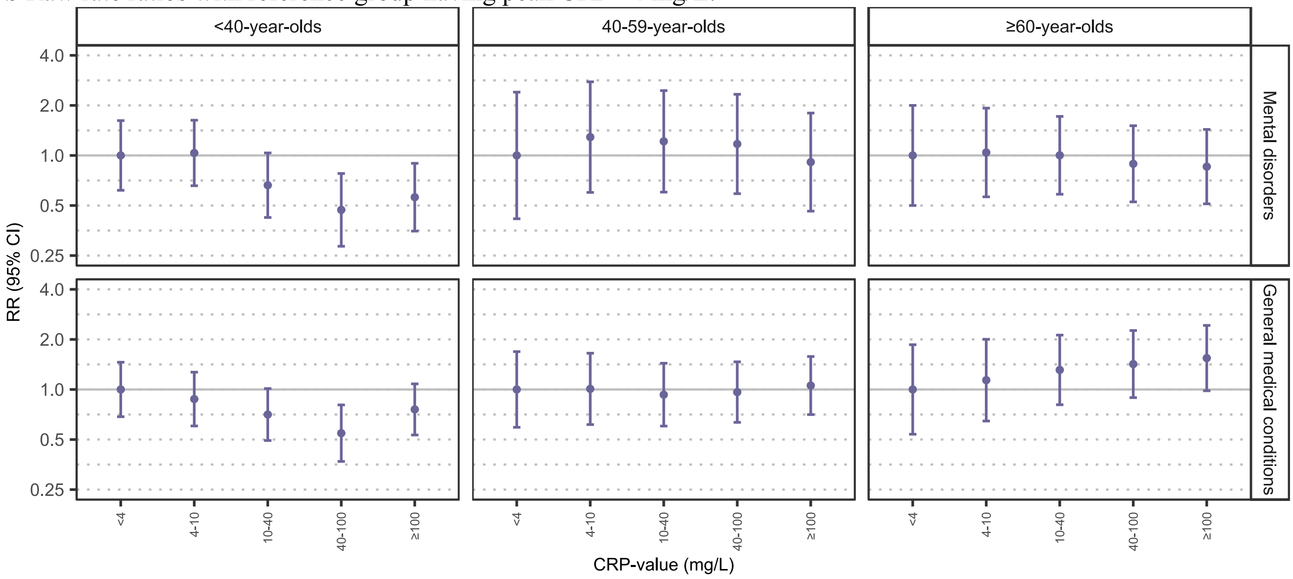

Abbreviations: RR, Rate Ratio; CI, Confidence Interval; CRP, C-Reactive Protein.

Sensitivity Analysis: Positive compared with negative SARS-CoV-2 test
Sensitivity analysis 1: Various levels of adjusting for confounders.

Results from the primary outcome analysis of positive compared to negative SARS-CoV-2 tests were tested by adjusting for various levels of confounders. All models have calendar time as the underlying time scale. After having adjusted for age, adding additional confounders did not change the general effects. All statistical tests were two-sided without correction for multiple comparisons.

Outcome	Adjustment	HR (95% CI)[a]	p-value
Mental disorders or general medical conditions	Unadjusted[a]	0.81 (0.81 – 0.82)	<0.001
	Age adjusted[b]	1.00 (0.99 - 1.01)	0.766
	Age and sex adjusted[c]	1.00 (0.99 - 1.01)	0.649
	Adjusted[d]	1.01 (1.00 - 1.02)	0.052
Mental disorders	Unadjusted[a]	0.92 (0.90 - 0.93)	<0.001
	Age adjusted[b]	0.82 (0.80 - 0.83)	<0.001
	Age and sex adjusted[c]	0.80 (0.79 - 0.82)	<0.001
	Adjusted[d]	0.82 (0.81 - 0.84)	<0.001
General medical conditions	Unadjusted[a]	0.79 (0.78 - 0.80)	<0.001
	Age adjusted[b]	1.02 (1.01 - 1.03)	0.001
	Age and sex adjusted[c]	1.02 (1.01 - 1.03)	<0.001
	Adjusted[d]	1.04 (1.03 - 1.05)	<0.001

Abbreviations: HR, Hazard Ratio; CI, Confidence Interval.

[a] Unadjusted model has no confounders adjustment .
[b] Age adjusted model is stratified by age in 10-year bands.
[c] Age and sex adjusted model is stratified by age in 10-year bands and adjusted for sex.
[d] The adjusted model is stratified by age in 10-year bands and adjusted for sex, Charlson Comorbidity Index (CCI), parental CCI, parental mental health, employment status, income, the highest level of education.

Sensitivity analysis 2: Number of conducted SARS-CoV-2 tests.

We explored how peoples' testing behavior affected results by adjusting for the number of SARS-CoV-2 tests conducted per individual by effect modification. People were divided into four groups: 0-3 tests, 4-9 tests, 10-14 tests, and 15 or more tests. Afterwards, the results for positive test results were compared to negative test results within each group. The effect of the number of tests was significant (p-value <0.001), and the hazard ratios for mental disorders and general medical conditions was only increased for individuals with more than 15 tests.

		Cases, No.			
**Outcome**	**Number of tests**	**Negative SARS-CoV-2**	**Positive SARS-CoV-2**	**HR (95% CI)[a]**	**p-value**
Mental disorders or general medical conditions	0-3 tests	29,101	3,645	0.92 (0.88 - 0.95)	<0.001
	4-9 tests	17,751	9,313	0.95 (0.93 - 0.98)	0.001
	10-14 tests	5,722	5,274	1.02 (0.98 - 1.06)	0.425
	≥15 tests	7,603	8,961	1.04 (1.01 - 1.08)	0.009
	p-value of effect modifier	..	..	..	<0.001
Mental disorders	0-3 tests	33,327	3,605	0.76 (0.74 - 0.79)	<0.001
	4-9 tests	22,701	10,888	0.80 (0.78 - 0.82)	<0.001
	10-14 tests	6,724	6,106	0.87 (0.84 - 0.91)	<0.001
	≥15 tests	7,886	9,693	0.94 (0.91 - 0.96)	<0.001
	p-value of effect modifier	..	..	..	<0.001
General medical conditions	0-3 tests	82,055	10,471	0.97 (0.95 - 0.99)	0.006
	4-9 tests	49,151	25,191	0.97 (0.95 - 0.98)	<0.001
	10-14 tests	15,413	13,874	1.03 (1.01 - 1.06)	0.006
	≥15 tests	21,710	25,102	1.05 (1.03 - 1.07)	<0.001
	p-value of effect modifier	..	..	..	<0.001

Abbreviations: HR, Hazard Ratio; CI, Confidence Interval.

[a] The estimates are HRs with 95% CI from Cox Proportional Hazards model stratified by age and adjusted for confounders (sex, Charlson Comorbidity Index (CCI), parental CCI, parental mental health, employment status, income, and highest level of education). All statistical tests were two-sided without correction for multiple comparisons.

Sensitivity analysis 3: Virus variants.

Different virus variants of SARS-CoV-2 have been connected to different levels of contagiousness and severity. The effect of virus variants was analyzed by dividing the underlying calendar time into five groups. The time periods where the variants were most dominant in Denmark is defined in Supplementary Table 33.

To explore whether different virus variants have exposed individuals to different levels of rate, we included the variant as an effect modifier and stratified by it, i.e., positive test results were compared to negative test results within each period. The overall effect was significant (p-value <0.001), but the hazard ratios for mental disorders and general medical conditions were generally still increased except for the Omicron variant, which was not associated with a significantly increased rate of mental disorders and general medical conditions. Analysis should be interpreted with caution, since the age groups dominating the exposure groups at different points in calendar time varies. Therefore, it is equally likely that the underlying effect was due to differences in age groups and not dominating virus variants.

		**Cases, No.**			
**Outcome**	**Virus variant**	**Negative SARS-CoV-2**	**Positive SARS-CoV-2**	**HR (95% CI)[a]**	**p-value**
Mental disorders or general medical conditions	Original	42,183	1,280	1.12 (1.06 - 1.18)	<0.001
	Alpha	33,696	2,029	1.09 (1.04 - 1.14)	<0.001
	Delta	39,770	3,375	1.05 (1.02 - 1.09)	0.003
	Omicron	26,067	19,889	0.98 (0.96 - 1.00)	0.016
	Omicron sub types	41,389	56,659	1.02 (1.01 - 1.03)	0.002
	p-value of effect modifier	..	..	..	<0.001
Mental disorder	Original	16,415	362	0.78 (0.70 - 0.86)	<0.001
	Alpha	13,050	704	0.86 (0.79 - 0.92)	<0.001
	Delta	15,885	1,300	0.86 (0.82 - 0.91)	<0.001
	Omicron	10,147	7,495	0.78 (0.76 - 0.81)	<0.001
	Omicron sub types	15,141	20,431	0.84 (0.83 - 0.86)	<0.001
	p-value of effect modifier	..	..	..	0.001
General medical conditions	Original	38,920	1,210	1.21 (1.14 - 1.28)	<0.001
	Alpha	30,195	1,853	1.17 (1.11 - 1.22)	<0.001
	Delta	35,668	2,952	1.09 (1.05 - 1.13)	<0.001
	Omicron	24,099	17,382	0.98 (0.96 - 1.01)	0.148
	Omicron sub types	39,447	51,241	1.02 (1.01 - 1.04)	0.001
	p-value of effect modifier	..	..	..	<0.001

Abbreviations: HR, Hazard Ratio; CI, Confidence Interval.

[a] The estimates are HRs with 95% CI from Cox Proportional Hazards model stratified by age and adjusted for confounders (sex, Charlson Comorbidity Index (CCI), parental CCI, parental mental health, employment status, income, and highest level of education). All statistical tests were two-sided without correction for multiple comparisons.

Sensitivity analysis 4: Lockdown.

The effect of lockdown was analyzed by dividing the underlying calendar time into six groups defined in Supplementary Table 34.

We then assessed the effect of lockdowns by including it as an effect modifier and stratifying by it, i.e., positive test results were compared to negative test results within each lockdown period. The effect modifier was significant (p-value <0.001), and the hazard ratios for mental disorders or general medical conditions were not increased in any lockdown period. Analysis should be interpreted with caution, since the age groups dominating the exposure groups at different points in calendar time varies. Therefore, it is equally likely that the underlying effect was due to differences in age groups and not calendar time.

		Cases, No.			
**Outcome**	**Lockdown**	**Negative SARS-CoV-2**	**Positive SARS-CoV-2**	**HR (95% CI)[a]**	**p-value**
Mental disorders or general medical conditions	1st lockdown	257	46	1.22 (0.89 - 1.68)	0.210
	1st inter lockdown	30,466	554	0.80 (0.58 - 1.12)	0.192
	2nd lockdown	15,020	903	1.03 (0.74 - 1.42)	0.879
	2nd inter lockdown	69,906	5,181	0.86 (0.63 - 1.19)	0.369
	3rd lockdown	8,635	2,106	0.79 (0.57 - 1.08)	0.142
	Post lockdowns	58,821	74,442	0.82 (0.60 - 1.13)	0.229
	p-value of effect modifier	..	..	..	<0.001
Mental disorder	1st lockdown	101	≤5[b]	..	..
	1st inter lockdown	11,539	139	0.66 (0.56 - 0.78)	<0.001
	2nd lockdown	6,207	296	0.91 (0.81 - 1.03)	0.131
	2nd inter lockdown	27,503	1,927	0.86 (0.82 - 0.90)	<0.001
	3rd lockdown	3,461	758	0.73 (0.67 - 0.79)	<0.001
	Post lockdowns	21,827	27,168	0.83 (0.82 - 0.85)	<0.001
	p-value of effect modifier	..	..	..	<0.001
General medical conditions	1st lockdown	272	46	1.16 (0.84 - 1.59)	0.369
	1st inter lockdown	28,443	530	1.05 (0.96 - 1.15)	0.255
	2nd lockdown	13,356	831	1.37 (1.28 - 1.47)	<0.001
	2nd inter lockdown	62,712	4,608	1.11 (1.08 - 1.14)	<0.001
	3rd lockdown	7,831	1,832	0.99 (0.94 - 1.04)	0.694
	Post lockdowns	55,715	66,791	1.01 (1.00 - 1.03)	0.028
	p-value of effect modifier	..	..	..	<0.001

Abbreviations: HR, Hazard Ratio; CI, Confidence Interval.

[a] The estimates are HRs with 95% CI from Cox Proportional Hazards model stratified by age and adjusted for confounders (sex, Charlson Comorbidity Index (CCI), parental CCI, parental mental health, employment status, income, and highest level of education). All statistical tests were two-sided without correction for multiple comparisons.

[b] Results from ≤5 patients are displayed as "≤5" to ensure data privacy.

Sensitivity analysis 5: Vaccination status.

Since December 2020 people in Denmark were offered vaccination against COVID-19 and the vaccines have been connected to protection against contracting COVID-19 and severe COVID-19.

To explore the effect of vaccinations, we included number of vaccinations as an effect modifier and stratified by it, i.e., positive SARS-CoV-2 test results were compared to negative SARS-CoV-2 test results within each vaccination group. The overall effect was significant (p-value 0.010), and the hazard ratios for mental disorders and general medical conditions were only increased for individuals not vaccinated. Otherwise, the hazard ratios for mental disorders and general medical conditions were not significantly increased.

		Cases, No.			
**Outcome**	**Vaccination**	**Negative SARS-CoV-2**	**Positive SARS-CoV-2**	**HR (95% CI)[a]**	**p-value**
Mental disorders or general medical conditions	No vaccination	26,970	5,475	1.05 (1.01 - 1.08)	0.016
	1 vaccination	2,649	686	1.03 (0.94 - 1.12)	0.549
	2 vaccinations	13,642	7,205	1.01 (0.97 - 1.04)	0.619
	3+ vaccinations	16,916	13,827	0.97 (0.95 - 1.00)	0.019
	p-value of effect modifier	..	..	..	0.010
Mental disorders	No vaccination	33,438	6,018	0.73 (0.70 - 0.75)	<0.001
	1 vaccination	3,235	838	0.82 (0.76 - 0.88)	<0.001
	2 vaccinations	17,920	11,067	0.83 (0.81 - 0.86)	<0.001
	3+ vaccinations	16,045	12,369	0.83 (0.81 - 0.85)	<0.001
	p-value of effect modifier	..	..	..	<0.001
General medical conditions	No vaccination	73,529	14,693	1.12 (1.09 - 1.14)	<0.001
	1 vaccination	7,186	1,833	1.14 (1.08 - 1.20)	<0.001
	2 vaccinations	36,973	17,595	1.05 (1.03 - 1.07)	<0.001
	3+ vaccinations	50,641	40,517	0.99 (0.98 - 1.01)	0.389
	p-value of effect modifier	..	..	..	<0.001

Abbreviations: HR, Hazard Ratio; CI, Confidence Interval.

[a] The estimates are HRs with 95% CI from Cox Proportional Hazards model stratified by age and adjusted for confounders (sex, Charlson Comorbidity Index (CCI), parental CCI, parental mental health, employment status, income, and highest level of education). All statistical tests were two-sided without correction for multiple comparisons.

Sensitivity analysis 6: Immigration status.

The purpose of the analysis was to investigate how immigrants and their descendants influence results because information on pre-existing comorbidities and parental information is not available before immigration. Based on immigration status, all individuals were divided into three groups, i) Danish origin, ii) immigrated to Denmark, and iii) descendants of immigrants. Positive SARS-CoV-2 tests were compared with negative SARS-CoV-2 tests within each group. The effect modification with immigration status was not significant (p-value 0.064), which meant that the test result alone explains the rates of disorders and that immigration status did not influence the outcome.

		Cases, No.			
**Outcome**	**Immigration status**	**Negative SARS-CoV-2**	**Positive SARS-CoV-2**	**HR (95% CI)[a]**	**p-value**
Mental disorders or general medical conditions	Danish origin	160,480	70,903	1.00 (0.99 - 1.01)	0.934
	Immigrants	18,131	9,115	1.02 (1.00 - 1.05)	0.112
	Immigrant descendants	4,516	3,217	0.96 (0.92 - 1.01)	0.088
	p-value of effect modifier	..	..	..	0.064
Mental disorders	Danish origin	61,626	25,767	0.84 (0.82 - 0.85)	<0.001
	Immigrants	7,129	3,241	0.81 (0.78 - 0.85)	<0.001
	Immigrant descendants	1,902	1,286	0.80 (0.74 - 0.86)	<0.001
	p-value of effect modifier	..	..	..	0.155
General medical conditions	Danish origin	148,625	63,777	1.03 (1.01 - 1.04)	<0.001
	Immigrants	15,934	8,151	1.07 (1.04 - 1.10)	<0.001
	Immigrant descendants	3,781	2,713	1.00 (0.95 - 1.06)	0.886
	p-value of effect modifier	..	..	..	0.007

Abbreviations: HR, Hazard Ratio; CI, Confidence Interval.

[a] The estimates are HRs with 95% CI from Cox Proportional Hazards model stratified by age and adjusted for confounders (sex, Charlson Comorbidity Index (CCI), parental CCI, parental mental health, employment status, income, and highest level of education). All statistical tests were two-sided without correction for multiple comparisons.

Sensitivity analysis 7: Excluding individuals with hospital contacts within five years of study start only.

The purpose of the analysis was to investigate how exclusion criterion affected results. Instead of excluding individuals with a pre-existing outcome disorder at some point before the start of the study, we only excluded individuals with a hospital contact for the outcome disorder five years before the start of the study. The number of individuals in the study population is showed below.

Number of individuals in the study population by SARS-CoV-2 test status and outcome.

Outcome	Total	SARS-CoV-2 positive[a]	SARS-CoV-2 negative[b]	No SARS-CoV-2 test[c]
Mental disorders or general medical conditions	4,617,100 (100%)	2,330,480 (50.5%)	1,714,830 (37.1%)	571,790 (12.4%)
Mental disorders	5,591,077 (100%)	2,955,097 (52.9%)	2,192,191 (39.2%)	443,789 (7.9%)
General medical conditions	4,508,295 (100%)	2,363,967 (52.4%)	1,710,520 (37.9%)	433,808 (9.6%)

Table description: The exposure groups, *SARS-CoV-2 positive SARS-CoV-2 test*, *SARS-CoV-2 negative SARS-CoV-2 test*, and *no SARS-CoV-2 test*, were identified by end of follow up.

[a] The group *SARS-CoV-2 positive SARS-CoV-2 test* consisted of everyone with at least one positive test by the end of follow up.
[b] The group *SARS-CoV-2 negative SARS-CoV-2 test* consisted of everyone with at least one negative and no positive test by the end of follow up.
[c] The group *No SARS-CoV-2 test* consisted of everyone without any SARS-CoV-2 test result by the end of follow up.

We show rates of specific first mental health or general medical disorders for i) negative and positive SARS-CoV-2 tests compared to individuals not tested, and ii) for positive SARS-CoV-2 tests compared to negative SARS-CoV-2 tests.

Outcome	SARS-CoV-2 test	Cases, No.	HR (95% CI)[a]	p-value
Mental disorders or general medical conditions	No test	141,902	1.00 [reference]	..
	Negative test	332,000	3.16 (3.14 - 3.19)	<0.001
	Positive test	130,290	3.33 (3.29 - 3.36)	<0.001
	Positive vs. negative test	..	1.05 (1.04 - 1.06)	<0.001
Mental disorders	No test	38,707	1.00 [reference]	..
	Negative test	82,829	1.39 (1.37 - 1.42)	<0.001
	Positive test	33,369	1.13 (1.10 - 1.15)	<0.001
	Positive vs. negative test	..	0.81 (0.80 - 0.82)	<0.001
General medical conditions	No test	145,429	1.00 [reference]	..
	Negative test	288,096	1.86 (1.85 - 1.88)	<0.001
	Positive test	111,648	1.86 (1.84 - 1.89)	<0.001
	Positive vs. negative test	..	1.00 (0.99 - 1.01)	0.998

Abbreviations: HR, Hazard Ratio; CI, Confidence Interval.

[a] The estimates are HRs with 95% CI from Cox Proportional Hazards model stratified by age and adjusted for confounders (sex, Charlson Comorbidity Index (CCI), parental CCI, parental mental health, employment status, income, highest level of education). All statistical tests were two-sided without correction for multiple comparisons.

Sensitivity Analysis: Positive compared with negative SARS-CoV-2 test (secondary outcomes)
Sensitivity analysis 1 (secondary outcomes): Various levels of adjusting for confounders.

All statistical tests were two-sided without correction for multiple comparisons.

Outcome	Adjustment	HR (95% CI)[a]	p-value
Neurological disorders	Unadjusted[a]	0.69 (0.68 - 0.69)	<0.001
	Age adjusted[b]	0.98 (0.97 - 0.99)	0.001
	Age and sex adjusted[c]	0.98 (0.97 - 0.99)	0.001
	Adjusted[d]	1.01 (0.99 - 1.02)	0.292
Respiratory disorders	Unadjusted[a]	0.69 (0.67 - 0.70)	<0.001
	Age adjusted[b]	0.98 (0.96 - 1.00)	0.041
	Age and sex adjusted[c]	0.98 (0.96 - 1.00)	0.048
	Adjusted[d]	1.03 (1.01 - 1.05)	0.007
Circulatory disorders	Unadjusted[a]	0.56 (0.55 - 0.57)	<0.001
	Age adjusted[b]	0.97 (0.96 - 0.98)	<0.001
	Age and sex adjusted[c]	0.97 (0.96 - 0.99)	0.001
	Adjusted[d]	1.00 (0.99 - 1.02)	0.738
Kidney disorders	Unadjusted[a]	0.40 (0.39 - 0.41)	<0.001
	Age adjusted[b]	0.84 (0.81 - 0.87)	<0.001
	Age and sex adjusted[c]	0.84 (0.81 - 0.87)	<0.001
	Adjusted[d]	0.89 (0.85 - 0.92)	<0.001
Gastrointestinal disorders	Unadjusted[a]	0.68 (0.66 - 0.69)	<0.001
	Age adjusted[b]	0.91 (0.89 - 0.93)	<0.001
	Age and sex adjusted[c]	0.91 (0.89 - 0.93)	<0.001
	Adjusted[d]	0.96 (0.94 - 0.98)	<0.001
Endocrine disorders	Unadjusted[a]	0.58 (0.57 - 0.60)	<0.001
	Age adjusted[b]	0.90 (0.87 - 0.93)	<0.001
	Age and sex adjusted[c]	0.90 (0.87 - 0.93)	<0.001
	Adjusted[d]	0.98 (0.94 - 1.01)	0.134
Hematological disorders	Unadjusted[a]	0.50 (0.49 - 0.52)	<0.001
	Age adjusted[b]	0.90 (0.87 - 0.92)	<0.001
	Age and sex adjusted[c]	0.90 (0.87 - 0.92)	<0.001
	Adjusted[d]	0.95 (0.92 - 0.98)	<0.001
Musculoskeletal disorders	Unadjusted[a]	0.95 (0.93 - 0.98)	<0.001
	Age adjusted[b]	1.07 (1.04 - 1.10)	<0.001
	Age and sex adjusted[c]	1.05 (1.03 - 1.08)	<0.001
	Adjusted[d]	1.07 (1.04 - 1.10)	<0.001
Dermatological disorders	Unadjusted[a]	1.14 (1.10 - 1.19)	<0.001
	Age adjusted[b]	1.07 (1.03 - 1.11)	<0.001
	Age and sex adjusted[c]	1.06 (1.02 - 1.10)	0.003
	Adjusted[d]	1.08 (1.04 - 1.12)	<0.001
Neuropsychiatric symptoms	Unadjusted[a]	0.69 (0.67 - 0.70)	<0.001
	Age adjusted[b]	1.07 (1.05 - 1.09)	<0.001
	Age and sex adjusted[c]	1.07 (1.05 - 1.09)	<0.001
	Adjusted[d]	1.11 (1.09 - 1.14)	<0.001

Abbreviations: HR, Hazard Ratio; CI, Confidence Interval.

[a] Unadjusted model has no confounders adjustment .
[b] Age adjusted model is stratified by age in 10-year bands.
[c] Age and sex adjusted model is stratified by age in 10-year bands and adjusted for sex.
[d] The adjusted model is stratified by age in 10-year bands and adjusted for sex, Charlson Comorbidity Index (CCI), parental CCI, parental mental health, employment status, income, the highest level of education.

Sensitivity analysis 2 (secondary outcomes): Number of conducted SARS-CoV-2 tests.

		Cases, No.			
**Outcome**	**Number of tests**	**Negative SARS-CoV-2**	**Positive SARS-CoV-2**	**HR (95% CI)[a]**	**p-value**
Neurological disorders	0-3 tests	65,269	7,982	0.95 (0.92 - 0.97)	<0.001
	4-9 tests	39,173	18,688	0.95 (0.94 - 0.97)	<0.001
	10-14 tests	11,501	9,767	0.99 (0.97 - 1.02)	0.694
	≥15 tests	14,803	16,509	1.00 (0.98 - 1.02)	0.875
	p-value of effect modifier	..	..	..	0.001
Respiratory disorders	0-3 tests	23,057	2,109	1.00 (0.96 - 1.05)	0.976
	4-9 tests	14,981	5,133	0.83 (0.80 - 0.85)	<0.001
	10-14 tests	3,838	2,769	0.96 (0.91 - 1.00)	0.075
	≥15 tests	4,426	4,794	1.07 (1.03 - 1.12)	0.001
	p-value of effect modifier	..	..	..	<0.001
Circulatory disorders	0-3 tests	48,613	5,372	0.97 (0.95 - 1.00)	0.079
	4-9 tests	25,635	9,893	0.93 (0.90 - 0.95)	<0.001
	10-14 tests	7,391	4,817	0.95 (0.91 - 0.98)	0.006
	≥15 tests	9,851	9,246	1.00 (0.97 - 1.03)	0.914
	p-value of effect modifier	..	..	..	<0.001
Kidney disorders	0-3 tests	10,295	923	0.90 (0.84 - 0.97)	0.004
	4-9 tests	6,136	1,643	0.75 (0.71 - 0.79)	<0.001
	10-14 tests	1,446	737	0.79 (0.72 - 0.86)	<0.001
	≥15 tests	1,246	1,068	0.92 (0.85 - 1.00)	0.051
	p-value of effect modifier	..	..	..	<0.001
Gastrointestinal disorders	0-3 tests	23,106	1,866	0.85 (0.81 - 0.89)	<0.001
	4-9 tests	14,053	4,911	0.85 (0.82 - 0.88)	<0.001
	10-14 tests	3,972	2,683	0.94 (0.89 - 0.99)	0.014
	≥15 tests	5,357	5,159	0.98 (0.94 - 1.02)	0.293
	p-value of effect modifier	..	..	..	<0.001
Endocrine disorders	0-3 tests	11,398	1,301	1.06 (1.00 - 1.13)	0.051
	4-9 tests	6,225	2,371	0.92 (0.88 - 0.97)	0.002
	10-14 tests	1,569	1,026	0.92 (0.85 - 1.00)	0.051
	≥15 tests	1,982	1,702	0.89 (0.83 - 0.95)	0.001
	p-value of effect modifier	..	..	..	<0.001
Hematological disorders	0-3 tests	15,054	1,268	0.88 (0.83 - 0.93)	<0.001
	4-9 tests	9,123	2,881	0.83 (0.79 - 0.86)	<0.001
	10-14 tests	2,223	1,403	0.90 (0.84 - 0.96)	0.002
	≥15 tests	2,232	2,192	0.99 (0.93 - 1.05)	0.795
	p-value of effect modifier	..	..	..	<0.001
Musculoskeletal disorders	0-3 tests	9,591	1,026	0.90 (0.84 - 0.96)	0.003
	4-9 tests	6,916	3,243	1.00 (0.95 - 1.04)	0.913
	10-14 tests	2,572	2,087	1.03 (0.97 - 1.09)	0.411
	≥15 tests	4,039	4,566	1.06 (1.02 - 1.11)	0.009
	p-value of effect modifier	..	..	..	0.001
Dermatological disorders	0-3 tests	5,109	607	1.04 (0.96 - 1.14)	0.337
	4-9 tests	3,987	2,025	0.96 (0.91 - 1.02)	0.221
	10-14 tests	1,348	1,256	1.00 (0.92 - 1.08)	0.959
	≥15 tests	1,785	2,316	1.08 (1.02 - 1.16)	0.015
	p-value of effect modifier	..	..	..	0.038
Neuropsychiatric symptoms	0-3 tests	25,336	2,513	1.01 (0.97 - 1.05)	0.630
	4-9 tests	17,180	6,304	0.96 (0.93 - 0.99)	0.006
	10-14 tests	5,009	3,415	1.01 (0.97 - 1.06)	0.686
	≥15 tests	5,684	6,184	1.16 (1.12 - 1.21)	<0.001
	p-value of effect modifier	..	..	..	<0.001

Abbreviations: HR, Hazard Ratio; CI, Confidence Interval.

[a] The estimates are HRs with 95% CI from Cox Proportional Hazards model stratified by age and adjusted for confounders (sex, Charlson Comorbidity Index (CCI), parental CCI, parental mental health, employment status, income, and highest level of education). All statistical tests were two-sided without correction for multiple comparisons.

Sensitivity analysis 3 (secondary outcomes): Virus variants.

Outcome	Virus variant	Cases, No. Negative SARS-CoV-2	Cases, No. Positive SARS-CoV-2	HR (95% CI)[a]	p-value
Neurological disorders	Original	27,383	641	0.97 (0.90 - 1.05)	0.436
	Alpha	22,267	1,134	1.04 (0.98 - 1.11)	0.185
	Delta	25,886	1,706	0.97 (0.92 - 1.02)	0.192
	Omicron	19,919	11,684	0.95 (0.93 - 0.97)	<0.001
	Omicron sub types	35,291	37,781	1.01 (1.00 - 1.03)	0.147
	p-value of effect modifier	..	..	..	<0.001
Respiratory disorders	Original	12,925	533	1.80 (1.65 - 1.97)	<0.001
	Alpha	7,281	526	1.55 (1.42 - 1.70)	<0.001
	Delta	8,837	682	1.18 (1.09 - 1.28)	<0.001
	Omicron	6,237	3,305	0.98 (0.94 - 1.03)	0.493
	Omicron sub types	11,022	9,759	0.94 (0.91 - 0.97)	<0.001
	p-value of effect modifier	..	..	..	<0.001
Circulatory disorders	Original	20,920	714	1.40 (1.30 - 1.51)	<0.001
	Alpha	15,082	829	1.23 (1.15 - 1.32)	<0.001
	Delta	17,676	1,132	1.09 (1.03 - 1.16)	0.004
	Omicron	14,077	6,504	0.96 (0.93 - 0.99)	0.004
	Omicron sub types	23,735	20,149	0.95 (0.93 - 0.97)	<0.001
	p-value of effect modifier	..	..	..	<0.001
Kidney disorders	Original	4,370	194	1.96 (1.70 - 2.27)	<0.001
	Alpha	2,658	115	1.07 (0.89 - 1.29)	0.455
	Delta	3,240	179	1.14 (0.98 - 1.32)	0.094
	Omicron	3,085	1,009	0.95 (0.88 - 1.02)	0.189
	Omicron sub types	5,770	2,874	0.78 (0.74 - 0.81)	<0.001
	p-value of effect modifier	..	..	..	<0.001
Gastrointestinal disorders	Original	13,027	216	0.71 (0.62 - 0.81)	<0.001
	Alpha	8,068	375	0.93 (0.84 - 1.03)	0.174
	Delta	8,754	557	0.93 (0.85 - 1.01)	0.104
	Omicron	6,482	3,484	0.95 (0.91 - 0.99)	0.025
	Omicron sub types	10,157	9,987	0.97 (0.95 - 1.00)	0.063
	p-value of effect modifier	..	..	..	<0.001
Endocrine disorders	Original	4,930	272	2.39 (2.11 - 2.70)	<0.001
	Alpha	3,471	205	1.34 (1.17 - 1.55)	<0.001
	Delta	3,867	308	1.35 (1.20 - 1.52)	<0.001
	Omicron	3,263	1,436	0.93 (0.87 - 0.99)	0.035
	Omicron sub types	5,643	4,179	0.86 (0.82 - 0.89)	<0.001
	p-value of effect modifier	..	..	..	<0.001
Hematological disorders	Original	7,189	145	0.93 (0.79 - 1.10)	0.390
	Alpha	4,250	183	0.99 (0.85 - 1.15)	0.898
	Delta	5,032	296	1.07 (0.95 - 1.20)	0.267
	Omicron	4,336	1,689	0.93 (0.87 - 0.98)	0.013
	Omicron sub types	7,825	5,431	0.94 (0.91 - 0.97)	0.001
	p-value of effect modifier	..	..	..	0.286
Musculoskeletal disorders	Original	4,874	82	0.65 (0.52 - 0.81)	<0.001
	Alpha	4,275	203	0.88 (0.76 - 1.01)	0.074
	Delta	5,153	352	0.91 (0.82 - 1.02)	0.101
	Omicron	3,424	2,494	0.99 (0.94 - 1.05)	0.762
	Omicron sub types	5,392	7,791	1.15 (1.11 - 1.19)	<0.001
	p-value of effect modifier	..	..	..	<0.001
Dermatological disorders	Original	2,856	113	1.60 (1.33 - 1.94)	<0.001
	Alpha	2,348	155	1.18 (1.00 - 1.38)	0.051
	Delta	2,662	258	1.19 (1.04 - 1.35)	0.009
	Omicron	1,676	1,506	1.03 (0.96 - 1.11)	0.411
	Omicron sub types	2,687	4,172	1.05 (1.00 - 1.11)	0.045
	p-value of effect modifier	..	..	..	<0.001
Neuropsychiatric symptoms	Original	11,870	425	1.62 (1.47 - 1.78)	<0.001
	Alpha	8,890	613	1.48 (1.36 - 1.61)	<0.001
	Delta	11,046	954	1.39 (1.30 - 1.49)	<0.001
	Omicron	7,598	4,112	1.08 (1.04 - 1.13)	<0.001
	Omicron sub types	13,805	12,312	1.03 (1.01 - 1.06)	0.012
	p-value of effect modifier	..	..	..	<0.001

Abbreviations: HR, Hazard Ratio; CI, Confidence Interval.

[a] The estimates are HRs with 95% CI from Cox Proportional Hazards model stratified by age and adjusted for confounders (sex, Charlson Comorbidity Index (CCI), parental CCI, parental mental health, employment status, income, and highest level of education). All statistical tests were two-sided without correction for multiple comparisons.

Sensitivity analysis 4 (secondary outcomes): Lockdown.

Outcome	Lockdown	Cases, No. Negative SARS-CoV-2	Cases, No. Positive SARS-CoV-2	HR (95% CI)[a]	p-value
Neurological disorders	1st lockdown	95	16	1.51 (0.88 - 2.58)	0.131
	1st inter lockdown	19,677	285	0.87 (0.77 - 0.98)	0.021
	2nd lockdown	9,940	453	1.06 (0.97 - 1.17)	0.206
	2nd inter lockdown	45,824	2,727	0.99 (0.95 - 1.03)	0.711
	3rd lockdown	5,830	1,024	0.86 (0.80 - 0.92)	<0.001
	Post lockdowns	49,380	48,441	1.00 (0.99 - 1.01)	0.986
	p-value of effect modifier	..	..	..	<0.001
Respiratory disorders	1st lockdown	259	66	2.32 (1.77 - 3.04)	<0.001
	1st inter lockdown	10,045	218	1.36 (1.19 - 1.55)	<0.001
	2nd lockdown	3,373	292	2.14 (1.90 - 2.41)	<0.001
	2nd inter lockdown	15,366	1,165	1.32 (1.24 - 1.40)	<0.001
	3rd lockdown	1,835	368	1.16 (1.04 - 1.31)	0.010
	Post lockdowns	15,424	12,696	0.94 (0.92 - 0.97)	<0.001
	p-value of effect modifier	..	..	..	<0.001
Circulatory disorders	1st lockdown	301	52	1.33 (0.99 - 1.79)	0.061
	1st inter lockdown	15,017	306	1.22 (1.09 - 1.36)	0.001
	2nd lockdown	7,279	453	1.57 (1.43 - 1.73)	<0.001
	2nd inter lockdown	31,081	1,864	1.14 (1.09 - 1.19)	<0.001
	3rd lockdown	4,383	673	1.00 (0.92 - 1.08)	0.928
	Post lockdowns	33,429	25,980	0.95 (0.93 - 0.96)	<0.001
	p-value of effect modifier	..	..	..	<0.001
Kidney disorders	1st lockdown	99	43	4.67 (3.25 - 6.71)	<0.001
	1st inter lockdown	3,226	57	1.14 (0.88 - 1.49)	0.314
	2nd lockdown	1,326	108	2.22 (1.82 - 2.70)	<0.001
	2nd inter lockdown	5,617	280	1.10 (0.98 - 1.25)	0.106
	3rd lockdown	906	109	1.13 (0.93 - 1.39)	0.222
	Post lockdowns	7,949	3,774	0.81 (0.78 - 0.84)	<0.001
	p-value of effect modifier	..	..	..	<0.001
Gastrointestinal disorders	1st lockdown	91	≤5[b]	..	
	1st inter lockdown	10,046	90	0.52 (0.43 - 0.64)	<0.001
	2nd lockdown	3,728	159	0.98 (0.83 - 1.14)	0.757
	2nd inter lockdown	15,984	896	0.93 (0.87 - 1.00)	0.039
	3rd lockdown	2,106	362	0.90 (0.80 - 1.01)	0.064
	Post lockdowns	14,533	13,109	0.97 (0.95 - 0.99)	0.015
	p-value of effect modifier	..	..	..	<0.001
Endocrine disorders	1st lockdown	84	27	3.18 (2.05 - 4.93)	<0.001
	1st inter lockdown	3,605	116	2.04 (1.69 - 2.45)	<0.001
	2nd lockdown	1,623	159	2.50 (2.13 - 2.95)	<0.001
	2nd inter lockdown	6,956	483	1.33 (1.21 - 1.45)	<0.001
	3rd lockdown	963	198	1.33 (1.14 - 1.56)	<0.001
	Post lockdowns	7,943	5,417	0.86 (0.83 - 0.89)	<0.001
	p-value of effect modifier	..	..	..	<0.001
Hematological disorders	1st lockdown	99	6	0.63 (0.28 - 1.43)	0.270
	1st inter lockdown	5,546	63	0.72 (0.56 - 0.92)	0.008
	2nd lockdown	2,007	92	1.19 (0.97 - 1.47)	0.096
	2nd inter lockdown	8,819	463	1.05 (0.95 - 1.15)	0.353
	3rd lockdown	1,233	143	0.86 (0.72 - 1.02)	0.091
	Post lockdowns	10,928	6,977	0.94 (0.91 - 0.97)	<0.001
	p-value of effect modifier	..	..	..	0.006
Musculoskeletal disorders	1st lockdown	20	censored[c]	..	..
	1st inter lockdown	3,434	censored[c]	..	..
	2nd lockdown	1,894	63	0.72 (0.56 - 0.93)	0.011
	2nd inter lockdown	8,954	532	0.90 (0.82 - 0.98)	0.014
	3rd lockdown	1,047	193	0.79 (0.67 - 0.92)	0.003
	Post lockdowns	7,769	10,092	1.12 (1.08 - 1.15)	<0.001
	p-value of effect modifier	..	..	..	<0.001
Dermatological disorders	1st lockdown	21	≤5[b]	..	..
	1st inter lockdown	2,071	49	1.46 (1.10 - 1.93)	0.010
	2nd lockdown	1,017	87	1.82 (1.46 - 2.27)	<0.001
	2nd inter lockdown	4,757	390	1.16 (1.04 - 1.29)	0.005
	3rd lockdown	589	176	1.01 (0.85 - 1.20)	0.947
	Post lockdowns	3,774	5,502	1.05 (1.00 - 1.09)	0.030
	p-value of effect modifier	..	..	..	<0.001
Neuropsychiatric symptoms	1st lockdown	113	6	0.55 (0.24 - 1.25)	0.154
	1st inter lockdown	8,841	237	1.71 (1.50 - 1.95)	<0.001
	2nd lockdown	3,784	235	1.55 (1.36 - 1.77)	<0.001
	2nd inter lockdown	19,068	1,514	1.43 (1.35 - 1.50)	<0.001
	3rd lockdown	2,329	469	1.22 (1.10 - 1.35)	<0.001
	Post lockdowns	19,074	15,955	1.04 (1.02 - 1.06)	0.001
	p-value of effect modifier	..	..	..	<0.001

Abbreviations: HR, Hazard Ratio; CI, Confidence Interval.

[a] The estimates are HRs with 95% CI from Cox Proportional Hazards model stratified by age and adjusted for confounders (sex, Charlson Comorbidity Index (CCI), parental CCI, parental mental health, employment status, income, and highest level of education). All statistical tests were two-sided without correction for multiple comparisons.

[b] Results from ≤5 patients are displayed as "≤5" to ensure data privacy. [c] Results are censored to ensure data privacy.

Sensitivity analysis 5 (secondary outcomes): Vaccination status.

Outcome	Vaccination	Cases, No. Negative SARS-CoV-2	Cases, No. Positive SARS-CoV-2	HR (95% CI)[a]	p-value
Neurological disorders	No vaccination	48,615	8,442	1.06 (1.03 - 1.09)	<0.001
	1 vaccination	5,025	1,222	1.21 (1.13 - 1.29)	<0.001
	2 vaccinations	29,324	11,157	0.98 (0.95 - 1.01)	0.127
	3+ vaccinations	47,782	32,125	0.98 (0.97 - 1.00)	0.016
	p-value of effect modifier	..	..	..	<0.001
Respiratory disorders	No vaccination	20,528	3,352	1.25 (1.19 - 1.31)	<0.001
	1 vaccination	1,530	364	1.28 (1.14 - 1.44)	<0.001
	2 vaccinations	9,128	2,681	1.03 (0.98 - 1.09)	0.198
	3+ vaccinations	15,116	8,408	0.95 (0.92 - 0.98)	<0.001
	p-value of effect modifier	..	..	..	<0.001
Circulatory disorders	No vaccination	31,661	3,323	1.24 (1.19 - 1.29)	<0.001
	1 vaccination	3,810	407	1.08 (0.97 - 1.20)	0.141
	2 vaccinations	20,207	3,791	0.98 (0.94 - 1.02)	0.299
	3+ vaccinations	35,812	21,807	0.95 (0.94 - 0.97)	<0.001
	p-value of effect modifier	..	..	..	<0.001
Kidney disorders	No vaccination	5,849	520	1.22 (1.10 - 1.35)	<0.001
	1 vaccination	576	65	1.24 (0.96 - 1.61)	0.100
	2 vaccinations	4,157	465	0.85 (0.76 - 0.94)	0.002
	3+ vaccinations	8,541	3,321	0.83 (0.80 - 0.87)	<0.001
	p-value of effect modifier	..	..	..	<0.001
Gastrointestinal disorders	No vaccination	19,960	2,078	0.90 (0.85 - 0.95)	<0.001
	1 vaccination	1,806	265	0.99 (0.87 - 1.13)	0.851
	2 vaccinations	9,985	2,713	0.95 (0.90 - 1.00)	0.037
	3+ vaccinations	14,737	9,563	0.96 (0.94 - 0.99)	0.003
	p-value of effect modifier	..	..	..	0.167
Endocrine disorders	No vaccination	7,602	1,025	1.42 (1.31 - 1.53)	<0.001
	1 vaccination	784	108	1.16 (0.95 - 1.43)	0.151
	2 vaccinations	4,632	1,025	1.03 (0.95 - 1.11)	0.454
	3+ vaccinations	8,156	4,242	0.88 (0.85 - 0.92)	<0.001
	p-value of effect modifier	..	..	..	<0.001
Hematological disorders	No vaccination	9,994	1,068	1.06 (0.99 - 1.14)	0.119
	1 vaccination	967	125	1.06 (0.88 - 1.28)	0.544
	2 vaccinations	6,233	1,180	0.98 (0.91 - 1.05)	0.504
	3+ vaccinations	11,438	5,371	0.91 (0.88 - 0.94)	<0.001
	p-value of effect modifier	..	..	..	0.001
Musculoskeletal disorders	No vaccination	8,910	1,508	1.05 (0.99 - 1.13)	0.113
	1 vaccination	1,035	178	0.98 (0.83 - 1.15)	0.789
	2 vaccinations	5,485	2,152	1.04 (0.98 - 1.11)	0.158
	3+ vaccinations	7,688	7,084	1.09 (1.05 - 1.13)	<0.001
	p-value of effect modifier	..	..	..	0.425
Dermatological disorders	No vaccination	6,060	1,716	1.20 (1.12 - 1.28)	<0.001
	1 vaccination	621	164	0.87 (0.73 - 1.04)	0.122
	2 vaccinations	2,478	1,537	1.07 (1.00 - 1.16)	0.061
	3+ vaccinations	3,070	2,787	1.04 (0.98 - 1.10)	0.167
	p-value of effect modifier	..	..	..	<0.001
Neuropsychiatric symptoms	No vaccination	18,525	2,813	1.41 (1.35 - 1.48)	<0.001
	1 vaccination	2,409	407	1.33 (1.19 - 1.48)	<0.001
	2 vaccinations	12,664	3,372	1.18 (1.13 - 1.23)	<0.001
	3+ vaccinations	19,611	11,824	1.02 (1.00 - 1.04)	0.120
	p-value of effect modifier	..	..	..	<0.001

Abbreviations: HR, Hazard Ratio; CI, Confidence Interval.

[a] The estimates are HRs with 95% CI from Cox Proportional Hazards model stratified by age and adjusted for confounders (sex, Charlson Comorbidity Index (CCI), parental CCI, parental mental health, employment status, income, and highest level of education). All statistical tests were two-sided without correction for multiple comparisons.

Sensitivity analysis 6 (secondary outcomes): Immigration status.

Outcome	Immigration status	Cases, No. Negative SARS-CoV-2	Cases, No. Positive SARS-CoV-2	HR (95% CI)[a]	p-value
Neurological disorders	Danish origin	119,123	47,059	1.00 (0.99 - 1.01)	0.851
	Immigrants	9,866	4,691	0.99 (0.96 - 1.03)	0.749
	Immigrant descendants	1,760	1,200	0.88 (0.82 - 0.95)	0.001
	p-value of effect modifier	..	..	..	0.003
Respiratory disorders	Danish origin	42,329	12,805	1.01 (0.98 - 1.03)	0.634
	Immigrants	3,084	1,421	1.19 (1.12 - 1.27)	<0.001
	Immigrant descendants	890	580	1.14 (1.02 - 1.27)	0.017
	p-value of effect modifier	..	..	..	<0.001
Circulatory disorders	Danish origin	84,392	26,098	0.98 (0.96 - 1.00)	0.017
	Immigrants	6,553	2,824	1.02 (0.98 - 1.07)	0.372
	Immigrant descendants	547	406	1.13 (0.99 - 1.28)	0.068
	p-value of effect modifier	..	..	..	0.029
Kidney disorders	Danish origin	17,885	3,960	0.88 (0.84 - 0.91)	<0.001
	Immigrants	1,141	359	0.87 (0.78 - 0.99)	0.029
	Immigrant descendants	97	52	0.97 (0.69 - 1.37)	0.873
	p-value of effect modifier	..	..	..	0.838
Gastrointestinal disorders	Danish origin	40,870	12,047	0.93 (0.91 - 0.96)	<0.001
	Immigrants	4,943	2,120	1.01 (0.96 - 1.07)	0.582
	Immigrant descendants	678	452	1.02 (0.90 - 1.15)	0.749
	p-value of effect modifier	..	..	..	0.005
Endocrine disorders	Danish origin	18,628	5,191	0.93 (0.90 - 0.96)	<0.001
	Immigrants	2,356	1,061	1.08 (1.00 - 1.16)	0.050
	Immigrant descendants	190	148	1.15 (0.92 - 1.42)	0.218
	p-value of effect modifier	..	..	..	<0.001
Hematological disorders	Danish origin	25,734	6,279	0.90 (0.87 - 0.92)	<0.001
	Immigrants	2,516	1,181	1.21 (1.13 - 1.30)	<0.001
	Immigrant descendants	384	284	1.09 (0.93 - 1.27)	0.294
	p-value of effect modifier	..	..	..	<0.001
Musculoskeletal disorders	Danish origin	21,043	9,621	1.06 (1.03 - 1.10)	<0.001
	Immigrants	1,770	1,033	1.11 (1.03 - 1.20)	0.008
	Immigrant descendants	305	268	1.11 (0.94 - 1.31)	0.200
	p-value of effect modifier	..	..	..	0.510
Dermatological disorders	Danish origin	10,477	5,131	1.07 (1.03 - 1.12)	0.001
	Immigrants	1,200	635	1.04 (0.95 - 1.15)	0.401
	Immigrant descendants	553	438	1.09 (0.96 - 1.24)	0.168
	p-value of effect modifier	..	..	..	0.807
Neuropsychiatric symptoms	Danish origin	47,861	15,425	1.07 (1.05 - 1.10)	<0.001
	Immigrants	4,627	2,369	1.27 (1.20 - 1.33)	<0.001
	Immigrant descendants	723	622	1.33 (1.19 - 1.48)	<0.001
	p-value of effect modifier	..	..	..	<0.001

Abbreviations: HR, Hazard Ratio; CI, Confidence Interval.

[a] The estimates are HRs with 95% CI from Cox Proportional Hazards model stratified by age and adjusted for confounders (sex, Charlson Comorbidity Index (CCI), parental CCI, parental mental health, employment status, income, and highest level of education). All statistical tests were two-sided without correction for multiple comparisons.

Sensitivity analysis 7 (secondary outcomes): Excluding individuals with hospital contacts within five years of study start only.

Number of individuals in the study population by SARS-CoV-2 test status and outcome.

Outcome	Total	SARS-CoV-2 positive[a]	SARS-CoV-2 negative[b]	No SARS-CoV-2 test[c]
Neurological disorders	5,264,659 (100%)	2,776,201 (52.7%)	2,040,215 (38.8%)	448,243 (8.5%)
Respiratory disorders	5,610,260 (100%)	2,979,417 (53.1%)	2,198,857 (39.2%)	431,986 (7.7%)
Circulatory disorders	5,293,153 (100%)	2,847,163 (53.8%)	2,017,059 (38.1%)	428,931 (8.1%)
Kidney disorders	5,765,914 (100%)	3,073,038 (53.3%)	2,261,790 (39.2%)	431,086 (7.5%)
Gastrointestinal disorders	5,654,291 (100%)	2,999,329 (53%)	2,222,868 (39.3%)	432,094 (7.6%)
Endocrine disorders	5,667,092 (100%)	3,029,214 (53.5%)	2,212,080 (39%)	425,798 (7.5%)
Hematological disorders	5,746,164 (100%)	3,059,717 (53.2%)	2,254,464 (39.2%)	431,983 (7.5%)
Musculoskeletal disorders	5,734,182 (100%)	3,040,299 (53%)	2,260,437 (39.4%)	433,446 (7.6%)
Dermatological disorders	5,765,478 (100%)	3,059,886 (53.1%)	2,273,225 (39.4%)	432,367 (7.5%)
Neuropsychiatric symptoms	5,680,051 (100%)	3,014,021 (53.1%)	2,229,071 (39.2%)	436,959 (7.7%)

Hazard ratio of specific first mental health or general medical disorders.

Outcome	SARS-CoV-2 test	Cases, No.	HR (95% CI)[a]	p-value
Neurological disorders	No test	89,139	1.00 [reference]	..
	Negative test	175,541	1.56 (1.55 - 1.58)	<0.001
	Positive test	65,533	1.55 (1.53 - 1.57)	<0.001
	Positive vs. negative test	..	0.99 (0.98 - 1.01)	0.308
Respiratory disorders	No test	16,789	1.00 [reference]	..
	Negative test	57,412	4.13 (4.04 - 4.22)	<0.001
	Positive test	18,043	4.08 (3.96 - 4.20)	<0.001
	Positive vs. negative test	..	0.99 (0.97 - 1.01)	0.235
Circulatory disorders	No test	67,707	1.00 [reference]	..
	Negative test	134,828	1.92 (1.90 - 1.95)	<0.001
	Positive test	39,093	1.83 (1.81 - 1.86)	<0.001
	Positive vs. negative test	..	0.95 (0.94 - 0.97)	<0.001
Kidney disorders	No test	7,481	1.00 [reference]	..
	Negative test	20,305	2.39 (2.31 - 2.47)	<0.001
	Positive test	4,609	1.99 (1.90 - 2.08)	<0.001
	Positive vs. negative test	..	0.83 (0.80 - 0.86)	<0.001
Gastrointestinal disorders	No test	15,356	1.00 [reference]	..
	Negative test	56,833	3.86 (3.77 - 3.95)	<0.001
	Positive test	17,020	3.53 (3.42 - 3.63)	<0.001
	Positive vs. negative test	..	0.91 (0.90 - 0.93)	<0.001
Endocrine disorders	No test	13,269	1.00 [reference]	..
	Negative test	27,789	1.75 (1.71 - 1.80)	<0.001
	Positive test	7,755	1.61 (1.56 - 1.67)	<0.001
	Positive vs. negative test	..	0.92 (0.90 - 0.95)	<0.001
Hematological disorders	No test	10,332	1.00 [reference]	..
	Negative test	31,006	2.94 (2.86 - 3.02)	<0.001
	Positive test	8,303	2.63 (2.54 - 2.73)	<0.001
	Positive vs. negative test	..	0.90 (0.87 - 0.92)	<0.001
Musculoskeletal disorders	No test	8,590	1.00 [reference]	..
	Negative test	24,281	1.73 (1.68 - 1.79)	<0.001
	Positive test	11,336	1.86 (1.79 - 1.94)	<0.001
	Positive vs. negative test	..	1.07 (1.05 - 1.10)	<0.001
Dermatological disorders	No test	4,579	1.00 [reference]	..
	Negative test	12,678	2.14 (2.05 - 2.24)	<0.001
	Positive test	6,441	2.29 (2.17 - 2.43)	<0.001
	Positive vs. negative test	..	1.07 (1.03 - 1.11)	<0.001
Neuropsychiatric symptoms	No test	21,329	1.00 [reference]	..
	Negative test	57,432	2.22 (2.18 - 2.26)	<0.001
	Positive test	19,580	2.40 (2.34 - 2.47)	<0.001
	Positive vs. negative test	..	1.08 (1.06 - 1.10)	<0.001

Abbreviations: HR, Hazard Ratio; CI, Confidence Interval.

[a] The estimates are HRs with 95% CI from Cox Proportional Hazards model stratified by age and adjusted for confounders (sex, Charlson Comorbidity Index (CCI), parental CCI, parental mental health, employment status, income, highest level of education). All statistical tests were two-sided without correction for multiple comparisons.

Sensitivity Analysis: Admission with COVID-19 compared with no admission with COVID-19
Sensitivity analysis 8: Virus variants.

Different virus variants of SARS-CoV-2 have been connected to different levels of contagiousness and severity. The effect of virus variants was analyzed by dividing the underlying calendar time into five groups. The time periods where the variants were most dominant in Denmark is defined in Supplementary Table 33.

To explore whether different virus variants have exposed individuals to different levels of rate, we included the variant as an effect modifier and stratified by it, i.e., admissions with COVID-19 were compared to no admission with COVID-19 within each calendar period. The overall effect was significant (p-value <0.001), and the hazard ratios for mental disorders or general medical conditions were increased for all variants. Analysis should be interpreted with caution, since the age groups dominating the exposure groups at different points in calendar time varies. Therefore, it is equally likely that the underlying effect was due to differences in age groups and not dominating virus variants.

		Cases, No.			
**Outcome**	**Virus variant**	**No COVID-19 admission**	**COVID-19 admission**	**HR (95% CI)[a]**	**p-value**
Mental disorders or general medical conditions	Original	43,280	183	3.13 (2.70 - 3.63)	<0.001
	Alpha	35,592	133	3.39 (2.86 - 4.02)	<0.001
	Delta	43,013	132	2.43 (2.05 - 2.89)	<0.001
	Omicron	45,536	420	3.75 (3.41 - 4.13)	<0.001
	Omicron sub types	97,425	623	2.04 (1.88 - 2.20)	<0.001
	p-value of effect modifier	..	..	..	<0.001
Mental disorder	Original	16,728	49	1.55 (1.17 - 2.06)	0.002
	Alpha	13,699	55	2.03 (1.56 - 2.65)	<0.001
	Delta	17,110	75	1.83 (1.46 - 2.29)	<0.001
	Omicron	17,425	217	2.52 (2.20 - 2.89)	<0.001
	Omicron sub types	35,149	423	1.79 (1.62 - 1.97)	<0.001
	p-value of effect modifier	..	..	..	0.001
General medical conditions	Original	39,942	188	3.05 (2.64 - 3.53)	<0.001
	Alpha	31,917	131	3.28 (2.76 - 3.90)	<0.001
	Delta	38,478	142	2.63 (2.23 - 3.10)	<0.001
	Omicron	41,045	436	3.86 (3.51 - 4.24)	<0.001
	Omicron sub types	90,032	656	2.09 (1.94 - 2.26)	<0.001
	p-value of effect modifier	..	..	..	<0.001

Abbreviations: HR, Hazard Ratio; CI, Confidence Interval.

[a] The estimates are HRs with 95% CI from Cox Proportional Hazards model stratified by age and adjusted for confounders (sex, Charlson Comorbidity Index (CCI), parental CCI, parental mental health, employment status, income, and highest level of education). All statistical tests were two-sided without correction for multiple comparisons.

Sensitivity analysis 9: Lockdown.

The effect of lockdown was analyzed by dividing the underlying calendar time into six groups defined in Supplementary Table 34.

We then assessed the effect of lockdowns by including it as an effect modifier and stratifying by it, i.e., admissions with COVID-19 were compared to no admission with COVID-19 within each lockdown period. The effect modifier was significant (p-value <0.001), and the hazard ratios for mental disorders or general medical conditions were increased during all lockdown periods. Analysis should be interpreted with caution, since the age groups dominating the exposure groups at different points in calendar time varies. Therefore, it is equally likely that the underlying effect was due to differences in age groups and not calendar time.

		Cases, No.			
**Outcome**	**Lockdown**	**No COVID-19 admission**	**COVID-19 admission**	**HR (95% CI)[a]**	**p-value**
Mental disorders or general medical conditions	1st lockdown	278	25	2.26 (1.48 - 3.44)	<0.001
	1st inter lockdown	30,936	84	2.34 (1.89 - 2.90)	<0.001
	2nd lockdown	15,832	91	5.62 (4.57 - 6.91)	<0.001
	2nd inter lockdown	74,839	248	2.77 (2.45 - 3.14)	<0.001
	3rd lockdown	10,639	102	5.58 (4.59 - 6.79)	<0.001
	Post lockdowns	132,322	941	2.35 (2.21 - 2.51)	<0.001
	p-value of effect modifier	..	..	..	<0.001
Mental disorder	1st lockdown	103	censored[c]	..	..
	1st inter lockdown	11,656	censored[c]	..	..
	2nd lockdown	6,472	31	2.83 (1.99 - 4.03)	<0.001
	2nd inter lockdown	29,306	124	1.89 (1.59 - 2.26)	<0.001
	3rd lockdown	4,181	38	2.77 (2.01 - 3.82)	<0.001
	Post lockdowns	48,393	602	1.95 (1.80 - 2.11)	<0.001
	p-value of effect modifier	..	..	..	0.001
General medical conditions	1st lockdown	292	26	2.15 (1.43 - 3.24)	<0.001
	1st inter lockdown	28,881	92	2.44 (1.99 - 3.00)	<0.001
	2nd lockdown	14,103	84	5.06 (4.08 - 6.27)	<0.001
	2nd inter lockdown	67,061	259	2.88 (2.55 - 3.26)	<0.001
	3rd lockdown	9,562	101	5.49 (4.51 - 6.68)	<0.001
	Post lockdowns	121,515	991	2.43 (2.28 - 2.58)	<0.001
	p-value of effect modifier	..	..	..	<0.001

Abbreviations: HR, Hazard Ratio; CI, Confidence Interval.

[a] The estimates are HRs with 95% CI from Cox Proportional Hazards model stratified by age and adjusted for confounders (sex, Charlson Comorbidity Index (CCI), parental CCI, parental mental health, employment status, income, and highest level of education). All statistical tests were two-sided without correction for multiple comparisons.
[b] Results from ≤5 patients are displayed as "≤5" to ensure data privacy.
[c] Results are censored to ensure data privacy.

Sensitivity analysis 10: Vaccination status.

Since December 2020 people in Denmark were offered vaccination against COVID-19 and the vaccines have been connected to protection against contracting COVID-19 and severe COVID-19.

To explore the effect of vaccinations, we included number of vaccinations as an effect modifier and stratified by it, i.e., admissions with COVID-19 were compared to no admission with COVID-19 within each vaccination group. The overall effect was significant (p-value <0.001), and the hazard ratios for mental disorders and general medical conditions were increased for all individuals irrespective of vaccinations status.

		Cases, No.			
**Outcome**	**Vaccination**	**No COVID-19 admission**	**COVID-19 admission**	**HR (95% CI)[a]**	**p-value**
Mental disorders or general medical conditions	No vaccination	98,170	580	2.97 (2.73 - 3.22)	<0.001
	1 vaccination	10,053	42	2.02 (1.49 - 2.73)	<0.001
	2 vaccinations	62,948	282	2.26 (2.01 - 2.54)	<0.001
	3+ vaccinations	93,675	587	2.52 (2.32 - 2.73)	<0.001
	p-value of effect modifier	..	..	..	<0.001
Mental disorder	No vaccination	39,237	219	1.65 (1.45 - 1.89)	<0.001
	1 vaccination	4,043	30	1.92 (1.34 - 2.75)	<0.001
	2 vaccinations	28,796	191	1.80 (1.56 - 2.08)	<0.001
	3+ vaccinations	28,035	379	2.10 (1.90 - 2.33)	<0.001
	p-value of effect modifier	..	..	..	0.035
General medical conditions	No vaccination	87,630	592	3.13 (2.88 - 3.39)	<0.001
	1 vaccination	8,975	44	2.26 (1.68 - 3.04)	<0.001
	2 vaccinations	54,268	300	2.45 (2.19 - 2.75)	<0.001
	3+ vaccinations	90,541	617	2.49 (2.30 - 2.69)	<0.001
	p-value of effect modifier	..	..	..	<0.001

Abbreviations: HR, Hazard Ratio; CI, Confidence Interval.

[a] The estimates are HRs with 95% CI from Cox Proportional Hazards model stratified by age and adjusted for confounders (sex, Charlson Comorbidity Index (CCI), parental CCI, parental mental health, employment status, income, and highest level of education). All statistical tests were two-sided without correction for multiple comparisons.
[b] Results from ≤5 patients are displayed as "≤5" to ensure data privacy.

Sensitivity analysis 11: Immigration status.

The purpose of the analysis was to investigate how immigrants and their descendants influence results because information on pre-existing comorbidities and parental information is not available before immigration. Based on immigration status, all individuals were divided into three groups, i) Danish origin, ii) immigrated to Denmark, and iii) descendants of immigrants. Admissions with COVID-19 were compared to no admission with COVID-19 within each group. The effect modification with immigration status was not significant (p-value 0.122), which meant that the admission status alone explains the risk of neurological disorders and that immigration status did not influence the outcome.

		Cases, No.			
**Outcome**	**Immigration status**	**No COVID-19 admission**	**COVID-19 admission**	**HR (95% CI)[a]**	**p-value**
Mental disorders or general medical conditions	Danish origin	230,253	1,130	2.66 (2.51 - 2.82)	<0.001
	Immigrants	26,951	295	2.34 (2.08 - 2.62)	<0.001
	Immigrant descendants	7,667	66	2.74 (2.15 - 3.50)	<0.001
	p-value of effect modifier	..	..	..	0.122
Mental disorder	Danish origin	86,765	628	2.00 (1.84 - 2.16)	<0.001
	Immigrants	10,212	158	1.80 (1.54 - 2.11)	<0.001
	Immigrant descendants	3,155	33	2.07 (1.47 - 2.92)	<0.001
	p-value of effect modifier	..	..	..	0.497
General medical conditions	Danish origin	211,208	1,194	2.69 (2.54 - 2.85)	<0.001
	Immigrants	23,788	297	2.46 (2.19 - 2.75)	<0.001
	Immigrant descendants	6,432	62	3.05 (2.38 - 3.92)	<0.001
	p-value of effect modifier	..	..	..	0.199

Abbreviations: HR, Hazard Ratio; CI, Confidence Interval.

[a] The estimates are HRs with 95% CI from Cox Proportional Hazards model stratified by age and adjusted for confounders (sex, Charlson Comorbidity Index (CCI), parental CCI, parental mental health, employment status, income, and highest level of education). All statistical tests were two-sided without correction for multiple comparisons.
[b] Results from ≤5 patients are displayed as “≤5” to ensure data privacy.

Sensitivity analysis 12: Excluding individuals with hospital contacts within five years of study start only.

The purpose of the analysis was to investigate how exclusion criterion affected results. Instead of excluding individuals with a pre-existing outcome disorder at some point before the start of the study, we only excluded individuals with a hospital contact for the outcome disorder five years before the start of the study. We show rates of specific first mental health or general medical disorders among individuals admitted with COVID-19 with and without intensive care unit (ICU) admission compared to individuals without admission to a hospital with COVID-19.

Outcome	Admission status[b]	Cases, No.	HR (95% CI)[a]	p-value
Mental disorders or general medical conditions	No admission	459,178	1.00 [reference]	..
	Admission without ICU	2,850	2.26 (2.18 - 2.35)	<0.001
	Admission with ICU	262	4.20 (3.72 - 4.74)	<0.001
Mental disorders	No admission	116,288	1.00 [reference]	..
	Admission without ICU	936	1.82 (1.71 - 1.94)	<0.001
	Admission with ICU	91	2.48 (2.02 - 3.04)	<0.001
General medical conditions	No admission	416,360	1.00 [reference]	..
	Admission without ICU	2,885	2.29 (2.20 - 2.37)	<0.001
	Admission with ICU	267	4.17 (3.70 - 4.71)	<0.001

Abbreviations: HR, Hazard Ratio; CI, Confidence Interval; ICU, Intensive Care Unit.

[a] The estimates are HRs with 95% CI from Cox Proportional Hazards model stratified by age and adjusted for confounders (sex, Charlson Comorbidity Index (CCI), parental CCI, parental mental health, employment status, income, highest level of education). All statistical tests were two-sided without correction for multiple comparisons.
[b] The reference group *no COVID-19-admission* consisted of individuals without admission to a hospital with SARS-CoV-2 infection, i.e., all individuals with negative or positive test results but no hospital admission.

Sensitivity Analysis: Admission with COVID-19 compared with no admission with COVID-19 (secondary outcomes)

Sensitivity analysis 8 (secondary outcomes): Virus variants.

Outcome	Virus variant	Cases, No.		HR (95% CI)[a]	p-value
		No COVID-19 admission	COVID-19 admission		
Mental disorder or general medical conditions	Original	43,280	183	3.13 (2.70 - 3.63)	<0.001
	Alpha	35,592	133	3.39 (2.86 - 4.02)	<0.001
	Delta	43,013	132	2.43 (2.05 - 2.89)	<0.001
	Omicron	45,536	420	3.75 (3.41 - 4.13)	<0.001
	Omicron sub types	97,425	623	2.04 (1.88 - 2.20)	<0.001
	p-value of effect modifier	..	..	..	<0.001
Mental disorder	Original	16,728	49	1.55 (1.17 - 2.06)	0.002
	Alpha	13,699	55	2.03 (1.56 - 2.65)	<0.001
	Delta	17,110	75	1.83 (1.46 - 2.29)	<0.001
	Omicron	17,425	217	2.52 (2.20 - 2.89)	<0.001
	Omicron sub types	35,149	423	1.79 (1.62 - 1.97)	<0.001
	p-value of effect modifier	..	..	..	0.001
General medical conditions	Original	39,942	188	3.05 (2.64 - 3.53)	<0.001
	Alpha	31,917	131	3.28 (2.76 - 3.90)	<0.001
	Delta	38,478	142	2.63 (2.23 - 3.10)	<0.001
	Omicron	41,045	436	3.86 (3.51 - 4.24)	<0.001
	Omicron sub types	90,032	656	2.09 (1.94 - 2.26)	<0.001
	p-value of effect modifier	..	..	..	<0.001
Neurological disorders	Original	27,885	139	1.91 (1.62 - 2.26)	<0.001
	Alpha	23,268	133	1.86 (1.57 - 2.20)	<0.001
	Delta	27,410	182	2.02 (1.75 - 2.34)	<0.001
	Omicron	31,209	394	1.96 (1.77 - 2.17)	<0.001
	Omicron sub types	72,105	967	1.57 (1.47 - 1.68)	<0.001
	p-value of effect modifier	..	..	..	<0.001
Respiratory disorders	Original	13,219	239	4.15 (3.64 - 4.74)	<0.001
	Alpha	7,666	141	4.50 (3.80 - 5.32)	<0.001
	Delta	9,387	132	3.35 (2.82 - 3.98)	<0.001
	Omicron	9,196	346	4.32 (3.88 - 4.82)	<0.001
	Omicron sub types	20,139	642	2.71 (2.51 - 2.94)	<0.001
	p-value of effect modifier	..	..	..	<0.001
Circulatory disorders	Original	21,397	237	2.97 (2.61 - 3.39)	<0.001
	Alpha	15,767	144	2.83 (2.40 - 3.34)	<0.001
	Delta	18,675	133	2.12 (1.79 - 2.52)	<0.001
	Omicron	20,113	468	3.67 (3.34 - 4.02)	<0.001
	Omicron sub types	43,234	650	1.77 (1.64 - 1.91)	<0.001
	p-value of effect modifier	..	..	..	<0.001
Kidney disorders	Original	4,444	120	3.56 (2.96 - 4.28)	<0.001
	Alpha	2,713	60	2.75 (2.12 - 3.55)	<0.001
	Delta	3,336	83	3.00 (2.41 - 3.73)	<0.001
	Omicron	3,858	236	3.67 (3.21 - 4.19)	<0.001
	Omicron sub types	8,225	419	2.10 (1.90 - 2.32)	<0.001
	p-value of effect modifier	..	..	..	<0.001
Gastrointestinal disorders	Original	13,196	47	0.95 (0.72 - 1.27)	0.751
	Alpha	8,379	64	1.93 (1.51 - 2.47)	<0.001
	Delta	9,240	71	1.84 (1.45 - 2.32)	<0.001
	Omicron	9,756	210	2.63 (2.29 - 3.02)	<0.001
	Omicron sub types	19,741	403	1.79 (1.62 - 1.98)	<0.001
	p-value of effect modifier	..	..	..	<0.001
Endocrine disorders	Original	5,110	92	3.70 (3.00 - 4.57)	<0.001
	Alpha	3,631	45	2.65 (1.97 - 3.55)	<0.001
	Delta	4,110	65	3.23 (2.53 - 4.13)	<0.001
	Omicron	4,544	155	3.54 (3.01 - 4.16)	<0.001
	Omicron sub types	9,559	263	2.03 (1.80 - 2.30)	<0.001
	p-value of effect modifier	..	..	..	<0.001
Hematological disorders	Original	7,247	87	1.94 (1.57 - 2.41)	<0.001
	Alpha	4,379	54	1.82 (1.39 - 2.38)	<0.001
	Delta	5,270	58	1.62 (1.25 - 2.10)	<0.001
	Omicron	5,753	272	3.40 (3.00 - 3.84)	<0.001
	Omicron sub types	12,644	612	2.50 (2.30 - 2.71)	<0.001
	p-value of effect modifier	..	..	..	<0.001
Musculoskeletal disorders	Original	4,949	7	0.64 (0.31 - 1.35)	0.240
	Alpha	4,466	12	0.94 (0.53 - 1.66)	0.830
	Delta	5,485	20	1.11 (0.71 - 1.72)	0.650
	Omicron	5,866	52	1.35 (1.03 - 1.78)	0.031
	Omicron sub types	13,057	126	1.08 (0.91 - 1.29)	0.378
	p-value of effect modifier	..	..	..	0.306

Dermatological disorders	Original	2,956	13	2.25 (1.30 - 3.89)	0.004
	Alpha	2,492	11	1.93 (1.06 - 3.49)	0.030
	Delta	2,905	15	2.00 (1.20 - 3.32)	0.008
	Omicron	3,131	51	2.97 (2.25 - 3.92)	<0.001
	Omicron sub types	6,772	87	1.68 (1.36 - 2.07)	<0.001
	p-value of effect modifier	..	..	..	0.044
Neuropsychiatric symptoms	Original	12,186	109	2.15 (1.78 - 2.60)	<0.001
	Alpha	9,413	90	2.00 (1.63 - 2.47)	<0.001
	Delta	11,891	109	1.86 (1.54 - 2.25)	<0.001
	Omicron	11,341	369	3.24 (2.92 - 3.60)	<0.001
	Omicron sub types	25,215	902	2.46 (2.30 - 2.63)	<0.001
	p-value of effect modifier	..	..	..	<0.001

Abbreviations: HR, Hazard Ratio; CI, Confidence Interval.

[a] The estimates are HRs with 95% CI from Cox Proportional Hazards model stratified by age and adjusted for confounders (sex, Charlson Comorbidity Index (CCI), parental CCI, parental mental health, employment status, income, and highest level of education). All statistical tests were two-sided without correction for multiple comparisons.

Sensitivity analysis 9 (secondary outcomes): Lockdown.

Outcome	Lockdown	Cases, No. No COVID-19 admission	Cases, No. COVID-19 admission	HR (95% CI)[a]	p-value
Mental disorder or general medical conditions	1st lockdown	278	25	2.26 (1.48 - 3.44)	<0.001
	1st inter lockdown	30,936	84	2.34 (1.89 - 2.90)	<0.001
	2nd lockdown	15,832	91	5.62 (4.57 - 6.91)	<0.001
	2nd inter lockdown	74,839	248	2.77 (2.45 - 3.14)	<0.001
	3rd lockdown	10,639	102	5.58 (4.59 - 6.79)	<0.001
	Post lockdowns	132,322	941	2.35 (2.21 - 2.51)	<0.001
	p-value of effect modifier	..	..	..	<0.001
Mental disorder	1st lockdown	103	censored[c]	..	..
	1st inter lockdown	11,656	censored[c]	..	..
	2nd lockdown	6,472	31	2.83 (1.99 - 4.03)	<0.001
	2nd inter lockdown	29,306	124	1.89 (1.59 - 2.26)	<0.001
	3rd lockdown	4,181	38	2.77 (2.01 - 3.82)	<0.001
	Post lockdowns	48,393	602	1.95 (1.80 - 2.11)	<0.001
	p-value of effect modifier	..	..	..	0.001
General medical conditions	1st lockdown	292	26	2.15 (1.43 - 3.24)	<0.001
	1st inter lockdown	28,881	92	2.44 (1.99 - 3.00)	<0.001
	2nd lockdown	14,103	84	5.06 (4.08 - 6.27)	<0.001
	2nd inter lockdown	67,061	259	2.88 (2.55 - 3.26)	<0.001
	3rd lockdown	9,562	101	5.49 (4.51 - 6.68)	<0.001
	Post lockdowns	121,515	991	2.43 (2.28 - 2.58)	<0.001
	p-value of effect modifier	..	..	..	<0.001
Neurological disorders	1st lockdown	100	11	2.39 (1.26 - 4.51)	0.007
	1st inter lockdown	19,884	78	1.72 (1.38 - 2.15)	<0.001
	2nd lockdown	10,327	66	2.19 (1.72 - 2.80)	<0.001
	2nd inter lockdown	48,252	299	1.94 (1.73 - 2.17)	<0.001
	3rd lockdown	6,788	66	2.24 (1.76 - 2.85)	<0.001
	Post lockdowns	96,526	1,295	1.65 (1.56 - 1.74)	<0.001
	p-value of effect modifier	..	..	..	0.010
Respiratory disorders	1st lockdown	278	47	3.16 (2.30 - 4.33)	<0.001
	1st inter lockdown	10,168	95	2.91 (2.37 - 3.56)	<0.001
	2nd lockdown	3,552	113	7.94 (6.57 - 9.59)	<0.001
	2nd inter lockdown	16,274	257	3.82 (3.37 - 4.32)	<0.001
	3rd lockdown	2,133	70	5.57 (4.39 - 7.08)	<0.001
	Post lockdowns	27,202	918	3.02 (2.83 - 3.23)	<0.001
	p-value of effect modifier	..	..	..	<0.001
Circulatory disorders	1st lockdown	323	30	1.66 (1.14 - 2.43)	0.009
	1st inter lockdown	15,225	98	2.18 (1.78 - 2.66)	<0.001
	2nd lockdown	7,602	130	5.41 (4.54 - 6.44)	<0.001
	2nd inter lockdown	32,689	256	2.38 (2.10 - 2.69)	<0.001
	3rd lockdown	4,954	102	4.61 (3.79 - 5.61)	<0.001
	Post lockdowns	58,393	1,016	2.15 (2.02 - 2.29)	<0.001
	p-value of effect modifier	..	..	..	<0.001
Kidney disorders	1st lockdown	107	35	5.85 (3.96 - 8.66)	<0.001
	1st inter lockdown	3,247	36	1.95 (1.40 - 2.71)	<0.001
	2nd lockdown	1,373	61	5.55 (4.29 - 7.18)	<0.001
	2nd inter lockdown	5,766	131	2.78 (2.34 - 3.31)	<0.001
	3rd lockdown	969	46	4.32 (3.21 - 5.82)	<0.001
	Post lockdowns	11,114	609	2.41 (2.22 - 2.62)	<0.001
	p-value of effect modifier	..	..	..	<0.001
Gastrointestinal disorders	1st lockdown	92	censored[c]	..	..
	1st inter lockdown	10,117	censored[c]	..	..
	2nd lockdown	3,856	31	2.17 (1.52 - 3.09)	<0.001
	2nd inter lockdown	16,750	130	1.89 (1.59 - 2.25)	<0.001
	3rd lockdown	2,430	38	2.80 (2.03 - 3.86)	<0.001
	Post lockdowns	27,067	575	1.98 (1.82 - 2.15)	<0.001
	p-value of effect modifier	..	..	..	<0.001
Endocrine disorders	1st lockdown	98	13	2.58 (1.44 - 4.63)	0.002
	1st inter lockdown	3,684	37	2.64 (1.91 - 3.66)	<0.001
	2nd lockdown	1,736	46	5.79 (4.31 - 7.78)	<0.001
	2nd inter lockdown	7,333	106	3.01 (2.48 - 3.65)	<0.001
	3rd lockdown	1,125	36	4.94 (3.54 - 6.89)	<0.001
	Post lockdowns	12,978	382	2.30 (2.08 - 2.55)	<0.001
	p-value of effect modifier	..	..	..	<0.001
Hematological disorders	1st lockdown	102	≤5[b]	..	
	1st inter lockdown	5,567	42	1.46 (1.08 - 1.99)	0.014
	2nd lockdown	2,050	49	3.48 (2.61 - 4.62)	<0.001
	2nd inter lockdown	9,177	105	1.69 (1.39 - 2.05)	<0.001
	3rd lockdown	1,337	39	3.24 (2.35 - 4.46)	<0.001
	Post lockdowns	17,060	845	2.70 (2.52 - 2.90)	<0.001
	p-value of effect modifier	..	..	..	<0.001
Musculoskeletal disorders	censored [c]	..	..	..	..

Dermatological disorders	1st lockdown	20	censored[c]	..	..
	1st inter lockdown	2,112	censored[c]	..	..
	2nd lockdown	1,098	6	2.66 (1.19 - 5.97)	0.017
	2nd inter lockdown	5,123	24	1.90 (1.27 - 2.84)	0.002
	3rd lockdown	753	12	4.46 (2.52 - 7.92)	<0.001
	Post lockdowns	9,150	126	1.90 (1.59 - 2.27)	<0.001
	p-value of effect modifier	..	..	..	0.233
Neuropsychiatric symptoms	1st lockdown	116	≤5[b]	..	
	1st inter lockdown	9,008	70	2.21 (1.74 - 2.79)	<0.001
	2nd lockdown	3,976	43	2.33 (1.72 - 3.14)	<0.001
	2nd inter lockdown	20,390	192	1.94 (1.68 - 2.23)	<0.001
	3rd lockdown	2,748	50	2.75 (2.08 - 3.65)	<0.001
	Post lockdowns	33,808	1,221	2.65 (2.50 - 2.80)	<0.001
	p-value of effect modifier	..	..	..	<0.001

Abbreviations: HR, Hazard Ratio; CI, Confidence Interval.

[a] The estimates are HRs with 95% CI from Cox Proportional Hazards model stratified by age and adjusted for confounders (sex, Charlson Comorbidity Index (CCI), parental CCI, parental mental health, employment status, income, and highest level of education). All statistical tests were two-sided without correction for multiple comparisons.

[b] Results from ≤5 patients are displayed as "≤5" to ensure data privacy.

[c] Results are censored to ensure data privacy.

Sensitivity analysis 10 (secondary outcomes): Vaccination status.

Outcome	Vaccination	Cases, No. No COVID-19 admission	Cases, No. COVID-19 admission	HR (95% CI)[a]	p-value
Mental disorder or general medical conditions	No vaccination	98,170	580	2.97 (2.73 - 3.22)	<0.001
	1 vaccination	10,053	42	2.02 (1.49 - 2.73)	<0.001
	2 vaccinations	62,948	282	2.26 (2.01 - 2.54)	<0.001
	3+ vaccinations	93,675	587	2.52 (2.32 - 2.73)	<0.001
	p-value of effect modifier	..	..	..	<0.001
Mental disorder	No vaccination	39,237	219	1.65 (1.45 - 1.89)	<0.001
	1 vaccination	4,043	30	1.92 (1.34 - 2.75)	<0.001
	2 vaccinations	28,796	191	1.80 (1.56 - 2.08)	<0.001
	3+ vaccinations	28,035	379	2.10 (1.90 - 2.33)	<0.001
	p-value of effect modifier	..	..	..	0.035
General medical conditions	No vaccination	87,630	592	3.13 (2.88 - 3.39)	<0.001
	1 vaccination	8,975	44	2.26 (1.68 - 3.04)	<0.001
	2 vaccinations	54,268	300	2.45 (2.19 - 2.75)	<0.001
	3+ vaccinations	90,541	617	2.49 (2.30 - 2.69)	<0.001
	p-value of effect modifier	..	..	..	<0.001
Neurological disorders	No vaccination	56,624	433	2.03 (1.85 - 2.24)	<0.001
	1 vaccination	6,182	65	2.30 (1.80 - 2.94)	<0.001
	2 vaccinations	40,160	321	1.75 (1.57 - 1.95)	<0.001
	3+ vaccinations	78,911	996	1.62 (1.52 - 1.72)	<0.001
	p-value of effect modifier	..	..	..	<0.001
Respiratory disorders	No vaccination	23,396	484	4.32 (3.94 - 4.74)	<0.001
	1 vaccination	1,846	48	4.28 (3.21 - 5.70)	<0.001
	2 vaccinations	11,619	190	2.82 (2.44 - 3.25)	<0.001
	3+ vaccinations	22,746	778	3.06 (2.85 - 3.29)	<0.001
	p-value of effect modifier	..	..	..	<0.001
Circulatory disorders	No vaccination	34,454	530	3.39 (3.11 - 3.70)	<0.001
	1 vaccination	4,171	46	2.40 (1.79 - 3.21)	<0.001
	2 vaccinations	23,763	235	2.14 (1.88 - 2.44)	<0.001
	3+ vaccinations	56,798	821	2.09 (1.95 - 2.24)	<0.001
	p-value of effect modifier	..	..	..	<0.001
Kidney disorders	No vaccination	6,142	227	3.66 (3.20 - 4.20)	<0.001
	1 vaccination	614	27	3.78 (2.57 - 5.56)	<0.001
	2 vaccinations	4,483	139	2.76 (2.33 - 3.28)	<0.001
	3+ vaccinations	11,337	525	2.28 (2.09 - 2.49)	<0.001
	p-value of effect modifier	..	..	..	<0.001
Gastrointestinal disorders	No vaccination	21,864	174	1.65 (1.42 - 1.92)	<0.001
	1 vaccination	2,041	30	2.50 (1.73 - 3.61)	<0.001
	2 vaccinations	12,557	141	1.91 (1.62 - 2.26)	<0.001
	3+ vaccinations	23,850	450	1.91 (1.74 - 2.10)	<0.001
	p-value of effect modifier	..	..	..	0.153
Endocrine disorders	No vaccination	8,430	197	3.99 (3.46 - 4.61)	<0.001
	1 vaccination	884	8	1.38 (0.69 - 2.77)	0.363
	2 vaccinations	5,568	89	2.42 (1.96 - 2.99)	<0.001
	3+ vaccinations	12,072	326	2.31 (2.06 - 2.58)	<0.001
	p-value of effect modifier	..	..	..	<0.001
Hematological disorders	No vaccination	10,863	199	2.15 (1.87 - 2.48)	<0.001
	1 vaccination	1,063	29	2.89 (1.99 - 4.18)	<0.001
	2 vaccinations	7,266	147	2.11 (1.79 - 2.49)	<0.001
	3+ vaccinations	16,101	708	2.60 (2.41 - 2.81)	<0.001
	p-value of effect modifier	..	..	..	0.021
Musculoskeletal disorders	No vaccination	10,372	46	1.15 (0.86 - 1.54)	0.345
	1 vaccination	1,209	≤5[b]	..	..
	2 vaccinations	7,602	35	0.95 (0.68 - 1.32)	0.752
	3+ vaccinations	14,640	132	1.19 (1.00 - 1.41)	0.051
	p-value of effect modifier	..	..	..	0.514
Dermatological disorders	No vaccination	7,715	61	2.55 (1.98 - 3.29)	<0.001
	1 vaccination	781	≤5[b]	..	..
	2 vaccinations	3,984	31	1.84 (1.29 - 2.62)	0.001
	3+ vaccinations	5,776	81	1.81 (1.45 - 2.25)	<0.001
	p-value of effect modifier	..	..	..	0.161
Neuropsychiatric symptoms	No vaccination	21,055	283	2.27 (2.02 - 2.56)	<0.001
	1 vaccination	2,778	38	2.07 (1.50 - 2.85)	<0.001
	2 vaccinations	15,806	230	2.08 (1.82 - 2.37)	<0.001
	3+ vaccinations	30,407	1,028	2.64 (2.48 - 2.81)	<0.001
	p-value of effect modifier	..	..	..	0.002

Abbreviations: HR, Hazard Ratio; CI, Confidence Interval.

[a] The estimates are HRs with 95% CI from Cox Proportional Hazards model stratified by age and adjusted for confounders (sex, Charlson Comorbidity Index (CCI), parental CCI, parental mental health, employment status, income, and highest level of education). All statistical tests were two-sided without correction for multiple comparisons.
[b] Results from ≤5 patients are displayed as "≤5" to ensure data privacy.

Sensitivity analysis 11 (secondary outcomes): Immigration status.

Outcome	Immigration status	Cases, No.		HR (95% CI)[a]	p-value
		No COVID-19 admission	COVID-19 admission		
Mental disorder or general medical conditions	Danish origin	230,253	1,130	2.66 (2.51 - 2.82)	<0.001
	Immigrants	26,951	295	2.34 (2.08 - 2.62)	<0.001
	Immigrant descendants	7,667	66	2.74 (2.15 - 3.50)	<0.001
	p-value of effect modifier	..	..	..	0.122
Mental disorder	Danish origin	86,765	628	2.00 (1.84 - 2.16)	<0.001
	Immigrants	10,212	158	1.80 (1.54 - 2.11)	<0.001
	Immigrant descendants	3,155	33	2.07 (1.47 - 2.92)	<0.001
	p-value of effect modifier	..	..	..	0.497
General medical conditions	Danish origin	211,208	1,194	2.69 (2.54 - 2.85)	<0.001
	Immigrants	23,788	297	2.46 (2.19 - 2.75)	<0.001
	Immigrant descendants	6,432	62	3.05 (2.38 - 3.92)	<0.001
	p-value of effect modifier	..	..	..	0.199
Neurological disorders	Danish origin	164,685	1,497	1.73 (1.64 - 1.82)	<0.001
	Immigrants	14,281	276	1.76 (1.57 - 1.99)	<0.001
	Immigrant descendants	2,918	42	2.97 (2.19 - 4.03)	<0.001
	p-value of effect modifier	..	..	..	0.006
Respiratory disorders	Danish origin	53,877	1,257	3.42 (3.23 - 3.62)	<0.001
	Immigrants	4,287	218	3.33 (2.91 - 3.82)	<0.001
	Immigrant descendants	1,445	25	4.60 (3.09 - 6.83)	<0.001
	p-value of effect modifier	..	..	..	0.350
Circulatory disorders	Danish origin	109,103	1,387	2.43 (2.31 - 2.57)	<0.001
	Immigrants	9,155	222	2.13 (1.86 - 2.43)	<0.001
	Immigrant descendants	930	23	3.68 (2.44 - 5.57)	<0.001
	p-value of effect modifier	..	..	..	0.028
Kidney disorders	Danish origin	21,044	801	2.62 (2.44 - 2.81)	<0.001
	Immigrants	1,388	112	3.15 (2.60 - 3.82)	<0.001
	Immigrant descendants	144	≤5[b]	..	..
	p-value of effect modifier	..	..	..	0.186
Gastrointestinal disorders	Danish origin	52,266	651	1.89 (1.75 - 2.05)	<0.001
	Immigrants	6,943	120	1.42 (1.19 - 1.71)	<0.001
	Immigrant descendants	1,106	24	3.19 (2.13 - 4.78)	<0.001
	p-value of effect modifier	..	..	..	0.001
Endocrine disorders	Danish origin	23,340	479	2.50 (2.28 - 2.74)	<0.001
	Immigrants	3,283	134	2.82 (2.37 - 3.36)	<0.001
	Immigrant descendants	331	7	3.12 (1.48 - 6.60)	0.003
	p-value of effect modifier	..	..	..	0.422
Hematological disorders	Danish origin	31,072	941	2.57 (2.41 - 2.74)	<0.001
	Immigrants	3,572	125	1.75 (1.46 - 2.09)	<0.001
	Immigrant descendants	651	17	3.02 (1.87 - 4.90)	<0.001
	p-value of effect modifier	..	..	..	<0.001
Musculoskeletal disorders	Danish origin	30,490	174	1.10 (0.95 - 1.28)	0.210
	Immigrants	2,764	39	1.24 (0.90 - 1.70)	0.194
	Immigrant descendants	569	≤5[b]	..	..
	p-value of effect modifier	..	..	..	0.780
Dermatological disorders	Danish origin	15,460	148	2.20 (1.87 - 2.59)	<0.001
	Immigrants	1,812	23	1.29 (0.86 - 1.95)	0.221
	Immigrant descendants	985	6	1.31 (0.59 - 2.93)	0.507
	p-value of effect modifier	..	..	..	0.023
Neuropsychiatric symptoms	Danish origin	61,943	1,343	2.51 (2.38 - 2.65)	<0.001
	Immigrants	6,791	205	2.09 (1.82 - 2.40)	<0.001
	Immigrant descendants	1,314	31	3.24 (2.27 - 4.62)	<0.001
	p-value of effect modifier	..	..	..	0.017

Abbreviations: HR, Hazard Ratio; CI, Confidence Interval.

[a] The estimates are HRs with 95% CI from Cox Proportional Hazards model stratified by age and adjusted for confounders (sex, Charlson Comorbidity Index (CCI), parental CCI, parental mental health, employment status, income, and highest level of education). All statistical tests were two-sided without correction for multiple comparisons.
[b] Results from ≤5 patients are displayed as "≤5" to ensure data privacy.

Sensitivity analysis 12 (secondary outcomes): Excluding individuals with hospital contacts within five years of study start only.

Outcome	Admission status[b]	Cases, No.	HR (95% CI)[a]	p-value
Mental disorders or general medical conditions	No admission	459,178	1.00 [reference]	..
	Admission without ICU	2,850	2.26 (2.18 - 2.35)	<0.001
	Admission with ICU	262	4.20 (3.72 - 4.74)	<0.001
Mental disorders	No admission	116,288	1.00 [reference]	..
	Admission without ICU	936	1.82 (1.71 - 1.94)	<0.001
	Admission with ICU	91	2.48 (2.02 - 3.04)	<0.001
General medical conditions	No admission	416,360	1.00 [reference]	..
	Admission without ICU	2,885	2.29 (2.20 - 2.37)	<0.001
	Admission with ICU	267	4.17 (3.70 - 4.71)	<0.001
Neurological disorders	No admission	244,706	1.00 [reference]	..
	Admission without ICU	2,495	1.55 (1.49 - 1.62)	<0.001
	Admission with ICU	244	2.06 (1.82 - 2.34)	<0.001
Respiratory disorders	No admission	75,358	1.00 [reference]	..
	Admission without ICU	1,603	2.82 (2.68 - 2.96)	<0.001
	Admission with ICU	224	6.49 (5.69 - 7.40)	<0.001
Circulatory disorders	No admission	179,016	1.00 [reference]	..
	Admission without ICU	2,372	2.06 (1.98 - 2.14)	<0.001
	Admission with ICU	258	3.34 (2.96 - 3.78)	<0.001
Kidney disorders	No admission	24,191	1.00 [reference]	..
	Admission without ICU	832	2.33 (2.17 - 2.49)	<0.001
	Admission with ICU	141	7.02 (5.94 - 8.28)	<0.001
Gastrointestinal disorders	No admission	73,657	1.00 [reference]	..
	Admission without ICU	926	1.75 (1.64 - 1.87)	<0.001
	Admission with ICU	74	1.77 (1.41 - 2.23)	<0.001
Endocrine disorders	No admission	36,230	1.00 [reference]	..
	Admission without ICU	741	2.12 (1.97 - 2.28)	<0.001
	Admission with ICU	97	3.68 (3.01 - 4.49)	<0.001
Hematological disorders	No admission	38,541	1.00 [reference]	..
	Admission without ICU	1,093	2.39 (2.25 - 2.54)	<0.001
	Admission with ICU	91	3.14 (2.56 - 3.86)	<0.001
Musculoskeletal disorders	No admission	35,512	1.00 [reference]	..
	Admission without ICU	214	1.10 (0.96 - 1.25)	0.186
	Admission with ICU	17	1.16 (0.72 - 1.87)	0.542
Dermatological disorders	No admission	19,002	1.00 [reference]	..
	Admission without ICU	162	1.87 (1.60 - 2.19)	<0.001
	Admission with ICU	22	3.72 (2.45 - 5.66)	<0.001
Neuropsychiatric symptoms	No admission	75,908	1.00 [reference]	..
	Admission without ICU	1,617	2.40 (2.29 - 2.53)	<0.001
	Admission with ICU	132	2.83 (2.39 - 3.36)	<0.001

Abbreviations: HR, Hazard Ratio; CI, Confidence Interval; ICU, Intensive Care Unit.

[a] The estimates are HRs with 95% CI from Cox Proportional Hazards model stratified by age and adjusted for confounders (sex, Charlson Comorbidity Index (CCI), parental CCI, parental mental health, employment status, income, highest level of education). All statistical tests were two-sided without correction for multiple comparisons.
[b] The reference group *no COVID-19-admission* consisted of individuals without admission to a hospital with SARS-CoV-2 infection, i.e., all individuals with negative or positive test results but no hospital admission.

Supplementary Methods
Statistical analysis:

We reported hazard ratios (HRs) including 95% CIs based on the Wald statistic. The Cox proportional hazard assumption was assessed with visual inspections of the Schoenfeld residuals. Statistical analyses were done in R, version 4.4.1 with the *survival* package version 3.7-0, and the statistical significance level was set to a two-sided p-value < 0.05.

Analysis on: Time since positive SARS-CoV-2 test.

Time since positive SARS-CoV-2 test was defined as a time-varying variable, where individuals changed exposure groups after a given amount of time. E.g., an individual followed for 5 months after a positive SARS-COV-2 test until censoring was in the <1 month group for the first month. After 1 month the individual changed to the 1-2 months group, where the individual was until 3 months after positive test. Then the individual changed to the 3-5 months group until the 5 months had passed and so on.

Analysis on: Number of admissions (readmissions).

The number of admissions with COVID-19 was identified in a time-varying manner. All individuals were initially in the *no admissions with COVID-19* group. In case of a first admission with COVID-19 they changed to the *one admission with COVID-19* group until a potential re-admission with COVID-19, where they changed to the *two or more admissions with COVID-19* group. A third admission with COVID-19 did not lead to a change exposure groups.

Analysis on: Number of days in hospital (duration of admission).

The number of days in hospital during admission with COVID-19 was identified in a time-varying manner. As in the analysis on number of admissions, all individuals were initially in the *no admissions with COVID-19* group. In case of a first admission with COVID-19 they started in the *1-2 bed days* in hospital with COVID-19, and during admission they gradually changed groups. E.g., an individual in hospital for 5 days until censoring started in the *1-2 bed days* group and on the start of the third day, they changed to the *3-6 bed days* group until 5 days had passed.

Analysis on: Peak C-Reactive Protein (CRP) level and SARS-CoV-2 test.

We identified CRP measurements from blood samples. The peak CRP level was identified for individuals with a negative or positive SARS-CoV-2 test in a hierarchical and time-varying manner. Only individuals with a CRP measurement within two weeks (14 days) before the date of the SARS-CoV-2 test were included in the analysis. Note, that this deviates slightly from the analysis plan in the pre-registered protocol, where we wrote that we would consider peak CRP two weeks before *or after* the date of a SARS-CoV-2 test. The maximum CRP value within this timeframe was used as the CRP level and the date was chosen as the date of the SARS-CoV-2 test, irrespective of when the CRP measurement was taken. Individuals changed exposure groups later in case of either i) a higher CRP measurement, or ii) a lower CRP measurement and a first positive SARS-V-2 test.

Peak CRP level and SARS-CoV-2 PCR test

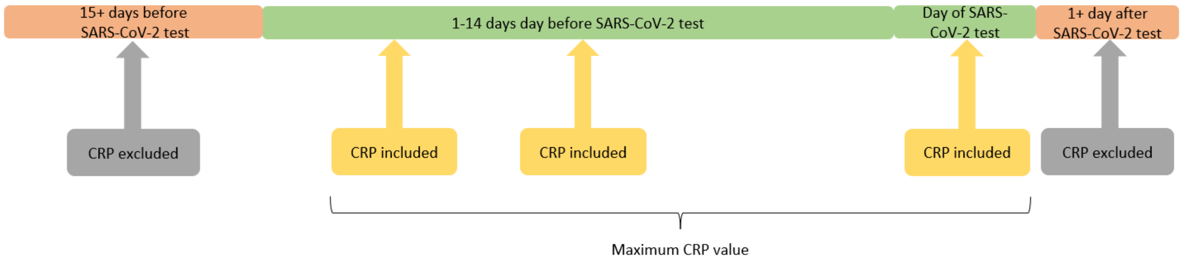

We analyzed how the outcome rates depended on CRP in two different ways:

First, we considered all individuals with negative or positive SARS-CoV-2 test results and CRP measurements corresponding to the above-mentioned definition. We then compared the interaction of the SARS-CoV-2 test result (negative or positive) to the peak CRP levels (<4 mg/L, 4-10 mg/L, 10-40 mg/L, 40-100 mg/L, and ≥100 mg/L). The reference was negative SARS-CoV-2 test results and peak CRP <4 mg/L.

Second, we only considered individuals with positive SARS-CoV-2 test results and CRP measurements corresponding to the above-mentioned definition. We then divided the peak CRP levels into 18 equally sized groups depending on case

distribution and compared the different levels. The reference was positive SARS-CoV-2 test results and the lowest CRP group (e.g., <0.7 mg/L).

Analysis on: Peak C-Reactive Protein (CRP) level and hospitalization with COVID-19.

We identified the peak CRP level for individuals hospitalized with COVID-19 in a hierarchical and time-varying manner. Only individuals with a CRP measurement two days before or during admission were included in the analysis. The maximum CRP value within this timeframe was used as the CRP level and the date was chosen as the date of CRP measurement. Individuals changed exposure groups in case a higher CRP measurement later during the same admission or during a subsequent admission.

Peak CRP level and hospitalization with COVID-19

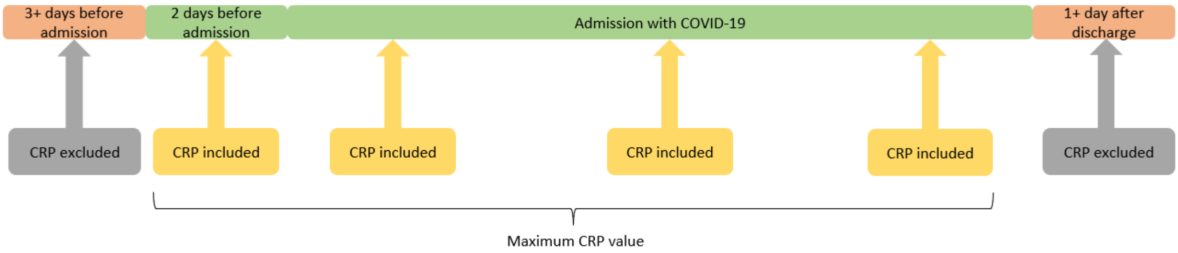

We analyzed how the outcome rates depended on CRP in two different ways:

First, we considered all individuals admitted with COVID-19 and with CRP measurements corresponding to the above-mentioned definition. We then compared the peak CRP levels (<4 mg/L, 4-10 mg/L, 10-40 mg/L, 40-100 mg/L, and ≥100 mg/L). The reference was admissions with COVID-19 and peak CRP <4 mg/L.

Second, again we considered all individuals admitted with COVID-19 and with CRP measurements corresponding to the above-mentioned definition. We then divided the peak CRP levels into 20 (primary outcome) or 10 (secondary outcomes) equally sized groups depending on case distribution and compared the different levels. The reference was admissions with COVID-19 and the lowest CRP group (e.g., <3 mg/L).

Analysis on: Positive SARS-CoV-2 test compared with out-of-hospital infections.

We excluded individuals with a prescription for any anti-infective agent between January 2019, and February 2020 to rule out recurring infections. We divided individuals into three exposure groups based on test result and redemption of anti-infective agent status (SARS-CoV-2 negative, prescription for anti-infective agents, SARS-CoV-2 positive). Since the three groups overlapped, we defined them in a time-varying hierarchical manner:

1. No infection: Individuals with only negative SARS-CoV-2 tests and without prescriptions for anti-infective agents.
2. Any prescription for anti-infective agents: Individuals with only negative SARS-CoV-2 tests and with a prescription for anti-infective agents.
3. SARS-CoV-2 positive: Individuals with a positive SARS-CoV-2 test, regardless of whether they have redeemed a prescription for anti-infective agents).

That is, individuals with both a positive SARS-CoV-2 test and a prescription for any anti-infective agent were allocated in the SARS-CoV-2 positive exposure group.

Analysis on: Hospitalization with COVID-19 compared with hospital-treated infections.

We excluded individuals with any hospital-treated pulmonary infection between January 2010, and February 2020 to rule out recurring infections. We divided individuals into three exposure groups based on admission status and infection type (no admission, admission with non-COVID-19 (pulmonary) infection, admission with COVID-19). Since the three groups overlapped, we defined them in a time-varying hierarchical manner:

1. No admission: Individuals with negative or positive SARS-CoV-2 test and no admission to hospital with COVID-19 or other infections.
2. Admission with non-COVID-19 (pulmonary) infection: Individuals with only SARS-CoV-2 negative tests and admission to hospital with non-COVID-19 (pulmonary) infections.
3. Admission with COVID-19: Individuals admitted with COVID-19, regardless of whether they were admitted with other (pulmonary) infections.

That is, individuals admitted with both COVID-19 and non-COVID-19 infections were allocated to the COVID-19 exposure group.

Supplementary Definitions

Supplementary Table 27: Disorders categorized by ICD-8, and ICD-10 codes.

Disorder	ICD-8	ICD-10
**Mental disorders**	All the below	All the below
Mental and behavioral disorders due to psychoactive substance use	291.x9, 294.39, 303.x9, 303.20, 303.28, 303.90, 304.x9	F10-F19
Schizophrenia spectrum disorder[1]	295.x9, 297.x9, 298.x9 (excl. 298.09, 298.19), 299.04, 299.05, 299.09, 301.83	F20-F29
Mood (affective) disorder[1]	296.x9, 298.09, 298.19, 300.49, 301.19	F30-F39
Depression	296.09, 296.29, 298.09, 300.49	F32-F33
Anxiety disorder[1]	300.x9, 305.x9, 305.68, 305.69, 307.99	F40-F48
PTSD	..	F43.1
**General medical conditions**	All the categories below	All the categories below
**Neurological disorders**	All the below	All the below
Vision problem	374.02, 364.02, 375 (excl. 375.03, 375.12), 379	H25, H40, H54[2]
Hearing problem	388, 389[3]	H90-H91, H93.1[2]
Encephalitis and Encephalopathy	065.99, 323, 999.19, 347.91, 347.99, 781.79	A86, G04-G05, G92
Parkinson's disease[4]	342	G20-G26
Dementia[4]	290.09, 290.10, 290.19, 293.09, 293.19	F00-F03, F05.1, G30
Alzheimer's disease	290.09, 290.10, 290.19	F00, G30
Guillain Barré[4]	354.00	G61.0
Epilepsy[4]	345 (excl. 345.29)	G40
Headache[4]	346, 791	G43, G44
Migraine[5]	346.00, 346.08, 346.09	G43[2,6]
Sleep apnea	..	G47.3[6]
Cerebrovascular disease[7]	430-438	I60-I69, G45[8], G46
Hemorrhagic stroke[7]	430, 431	I60, I61
Ischemic stroke[7]	433, 434	I63
Nerve/nerve root and plexus disorders[4]	350-352, 354.01, 354.08, 354.09, 355-357	G50-G59, G62.9, G63
Bell palsy[9]	350	G51.0
Peripheral neuropathy	354.01, 354.08, 354.09	G62.9, G63
Neuromuscular disease[4]	330	G70-G73
Myopathy[4]	..	G72
Disorders of the autonomic nervous system	358.00, 358.08, 358.09	G90, G99.1
**Respiratory disorders**	All the below	All the below
Chronic lung disease	490-493, 518.99	J40-J47[2]
Interstitial lung disease	484.99, 517, 519.21	J84[6]
Respiratory failure	..	J96.0, J96.1[6]
Acute respiratory failure	..	J96.0[6]
Chronic respiratory failure	..	J96.1[6]
**Circulatory disorders**	All the below	All the below
Hypertension	400-404	I10-I13 (excl. I11.0, I13.0, I13.2), I15[2]
Heart failure	427.0, 427.1, 782.4	I11.0, I13.0, I13.2, I50[8,10]
Lung hypertension	..	I27
Ischemic heart disease/coronary disease[11]	410-414	I20-I25[2,12]
Angina Pectoris	413	I20.9, I25.1[13]
Myocardial infarction	410	I20.0, I21-I23[8,13]
Cardiomyopathy	425.99	I25.5, I42.0-I42.5, I42.8, I42.9, I43, A36.81[6]
Venous thromboembolism (VTE)	450-451	I26, I80
Lung embolism (PE)	450	I26[12]
Deep vein thrombosis (DVT)	451	I80[12]
Pericarditis	393, 420, 423	I30-I32[14]
Myocarditis	422.99	I40-I41, I51.4
Cardiac arrhythmias	427.9, 782.29	I47-I49, R00.0
Tachycardia	427.90-427.92, 782.29	I47, R00.0[6]
Atrial fibrillation and flutter	427.93-427.94	I48[2,12]
Other cardiac arrhythmias	427.95-427.99	I49[6]
**Kidney disorders**	All the below	All the below
Chronic kidney disease	582, 590.09, 593.20, 792	N03, N11, N18-N19[2]
Acute renal failure	590.14, 593.19, 593.25	N17
**Gastrointestinal disorders**	All the below	All the below
Gastro-esophageal reflux disease	..	K21[15]
Peptic ulcer disease	530.91, 530.98, 531-534	K22.1, K25-K28[8]
Gastritis and duodenitis	535, 537.06	K29
Irritable bowel syndrome[16]	564.19	K58.0, K58.9
Liver disease	456.0, 571, 573.00, 573.01, 573.04, 700.0, 700.2, 700.4, 700.6, 700.8	B15.0, B16.0, B16.2, B18, B19.0, K70-K74, K76.0, K76.6, I85[8]
Mild liver disease	571, 573.01, 573.04	B18, K70.0-K70.3, K70.9, K71, K73, K74, K76.0[8]
Moderate/severe liver disease	456.0, 573.00, 700.0, 700.2, 700.4, 700.6, 700.8	B15.0, B16.0, B16.2, B19.0, K70.4, K72, K76.6, I85[8]

Cholangitis	575.04	K83.0
Acute pancreatitis	577	K85 (excl. K85.2, K85.3)
**Endocrine disorders**	All the below	All the below
Diabetes mellitus	249-250	E10-E14[2]
**Hematological disorders**	All the below	All the below
Anemia	280-285	D50-D53, D55-D61, D63-D64[2]
**Musculoskeletal disorders**	All the below	All the below
Pain in joint (arthralgia)	787.39	M25.5
**Dermatological disorders**	All the below	All the below
Urticaria	708.9, 709.92	L50[6]
Skin rash	788.29	R21
**Symptoms**	All the below	All the below
Somnolence	..	R40.0
Amnesia/Memory Difficulty	780.7	R41.1-R41.3
Dizziness and giddiness	780.59	R42
Disturbances of smell and taste	781.41, 781.42, 781.60, 781.64	R43
Loss of smell (anosmia)	781.64	R43.0[6]
Distortion of smell (parosmia)	781.42	R43.1
Distortion of taste (dysgeusia/parageusia)	..	R43.2
Fatigue diagnosis (Myalgic encephalomyelitis/chronic fatigue syndrome, fatigue syndrome, malaise, and fatigue)	790.10, 790.19, 300.59	R53, G93.3, F48.0[6]
Myalgia	..	M79.1

Abbreviations: ICD-8, International Classification of Diseases, Revision 8; ICD-10, International Classification of Diseases, Revision 10.

Table description: ICD-8 codes are used to exclude pre-existing disorders. ICD-10 codes are used to exclude pre-existing disorders and define outcome diagnoses. ATC codes were used in sensitivity analysis to identify individuals who had redeemed a prescription related to a specific diagnosis.

Supplementary Table 28: Charlson Comorbidity Index categorized by ICD-8 and ICD-10 codes.

Disorder within CCI	ICD-8	ICD-10
Myocardial infarction	410	I21-I23
Congestive heart failure	427.09, 427.10, 427.11, 427.19, 428.99, 782.49	I50, I11.0, I13.0, I13.2
Peripheral vascular disease	440, 441, 442, 443, 444, 445	I70-I74, I77
Cerebrovascular disease	430, 440	I60-I69, G45-G46
Dementia	290.09, 290.20, 293.09	F00-F03, F05.1, G30
Chronic lung disease	430, 440, 515, 520	J40-J47, J60-J67, J68.4, J70.1, J70.3, J84.1, J92.0, J96.1, J98.2, J98.3
Rheumatic disease (CTD)	712, 716, 734, 446, 153.99	M05-M06, M09-M08, M30-M36, D86
Peptic ulcer disease	530.91, 530.98, 531, 535	K22.1, K25-K28
Mild liver disease	571, 573.01, 573.04	K70.0-K70.3, K709, K71, K73-K74, K760
Diabetes without chronic complication (DIAB)	24900, 24906, 24907, 24909, 25000, 25006, 25007, 25009	E100-E101, E109-E111, E119
Diabetes with chronic complication (DIAB_O)	249.01-249.05, 249.08, 250.01-250.05, 250.08	E10.2-08, E11.2-E11.8
Hemiplegia or paraplegia	344	G81, G82
Renal disease	403, 404, 580, 581, 582, 583, 584, 590.09, 593.19, 753.1, 792	I12, I13, N00-N05, N11, N14, N17-N19, Q61
Any malignancy, including lymphoma and leukemia, except malignant neoplasm of skin	204-207, 200-203, 275.59	C91-C95, C81-C85, C88, C90, C96
Moderate or severe liver disease	700.0, 700.2, 700.4, 700.6, 700.8, 573.00, 456.0	B15.0, B16.0, B16.2, B19.0, K70.4
Metastatic solid tumor	140, 195-199	C00-C80
AIDS/HIV	798.3	B21-B24

Abbreviations: CCI, Charlson Comorbidity Index

Table description: Note, that some of the outcome diagnoses are part of the Charlson Comorbidity Index (CCI), but since individuals with prior diagnoses of the outcome diagnoses are excluded from the study population, the outcome diagnosis within CCI is not included in the model. Only in the adjustment for parental CCI are the outcome diagnoses included.

Supplementary Table 29: Definition of variables.

Variable	Categories	Registers and Comments
Confounders		At baseline
Age	Eight groups:<br><18 years<br>years<br>years<br>years<br>years<br>years<br>years<br>80+ years	Danish Civil Registration disorders (1968 – 2022-12-01)[17]<br>In case of too few events, broader age groups were used.
Sex	Two groups:<br>Male<br>Female	Danish Civil Registration disorders (1968 – 2022-12-01)[17]
CCi	Four groups:<br>0<br>1<br>2<br>3+	Danish National Patient Register version 2 and 3 (updated to 2023-06-30)[18]
Parental CCI	Four groups:<br>0<br>1<br>2<br>3+	Danish National Patient Register version 2 and 3 (updated to 2023-06-30)[18]
Parental mental disorder	Two groups:<br>No<br>Yes	Danish National Patient Register version 2 and 3 (updated to 2023-06-30)[18]<br>Any history of any mental disorder for either parent measured as an in-patient diagnosis of any kind (F00-99) at a mental hospital prior to time-zero.
Socio-Economic variables		At baseline
Highest achieved education level	Five groups:<br>No education<br>Elementary school<br>High school and vocational education<br>University (bachelor, professions bachelor)<br>University (master's, PhD)	Danish Education Registers (updated to 2020-12-31)[19]
Employment status	Five groups:<br>Kids/during education<br>Employed<br>Not in the workforce (disability pension)<br>Retired<br>Unemployed	Danish Occupational Register (updated to 2020-12-31)
Income quantile	Five groups:<br>0%-19%<br>20%-39%<br>40%-59%<br>60%-79%<br>80%-100%	The Income Statistics Register (updated to 2020-12-31)[20]
Sensitivity analysis		Time-varying
Vaccination status	Four groups:<br>0 vaccinations<br>1 vaccination<br>2 vaccination<br>3+ vaccinations	Danish Vaccination Register[21]

Abbreviations: CCI, Charlson Comorbidity Index.

Supplementary Table 30: Definition of exposures.

Variable	Definition	Comment
Confirmed SARS-CoV-2 infection (COVID-19-positive)	Positive SARS-CoV-2 polymerase chain reaction (PCR) by nasopharyngeal/tracheal test result	
COVID-19-negative	Negative SARS-CoV-2 polymerase chain reaction (PCR) by nasopharyngeal/tracheal test result	
COVID-19 reinfection	Positive SARS-CoV-2 test after a minimum of 60 days since the last positive test.	
ICU admission	Admitted to ICU with procedure codes of either intensive care observation or intensive care treatment (NABE, NABB)	As defined in previous studies[22,23] and validated with a positive predictive value of 87.2%.[24]<br>Note: Not conditioning on the duration of admission.
Hospital admission	Admitted to the hospital as an inpatient (with a duration of at least 12 hours or ICU admission)	
Hospital admission with COVID-19	Admission as an inpatient in the hospital either a) with a COVID-19 diagnosis code (DB342, DB972A) or b) (-2, 14) days after a positive SARS-CoV-2 test.	Individual were moved from reference to exposure group on the date where both criteria were met, i.e., inpatient, and positive COVID-19 test to avoid conditioning on future events.<br>The COVID-19 diagnosis codes were validated by six infectious disease departments in Denmark. They found that 99% of the patients included in the study were admitted with a positive SARS-CoV-2 test and symptoms suggestive of COVID-19.[25]
ICU admission with COVID-19	ICU admission either a) with a COVID-19 diagnosis code (DB342 or DB972A) or b) (-2, 14) days after a positive SARS-CoV-2 test.	Individuals were moved from 'Hospital admission with COVID-19' to 'ICU admission with COVID-19' on the date of admission to ICU.
CRP level	The information is available from the Danish Laboratory Database with the codes NPU01423 (hCRP, measured in nmol/L) or NPU19748 (CRP, measured in mg/L).	Lower detection limits were not existing for NPU01423. Lower detection limits were either <4 mg/L or <10 mg/L for NPU19748. All observations below the lower detection limit were imputed by simulating from a uniform distribution with min 0 and max equal to the lower detection limit.<br>Conversion from nmol/L to mg/L was done with the following formula:<br>CRP in mg/L = CRP in nmol/L / 9.52380952.

Abbreviations: ICU, Intensive Care Unit; CRP, C-Reactive Protein; hCRP, High Sensitivity C-Reactive Protein

Supplementary Table 31: Anti-infective agents categorized by ATC codes.

Type of anti-infective agent	ATC	Comment
Antibacterial	All the below	Including antiseptics
Anti-infectives	J01A-G, J01M, J01R, J01X	
Mycobacterium	J04A-B	Treatment of tuberculosis and lepra
Respiratory disorders	R02AA-AB	Antiseptics and antibiotics
Antiviral	J05A	Direct acting antivirals
Antimycotic	J02AA-AC, J02AX	

Table description: ATC codes are used to identify individuals who have redeemed a prescription for any anti-infective agent.

Supplementary Table 32: Infection codes categorized by ICD-10 codes.

Type of infection	ICD-10
Any infection[1]	A00–A99,<br>B00–B99,<br>D73.3,<br>E06, E23.6A, E32.1,<br>G00–G09, G53.0, G53.1, G63.0, G73.4, G95.1,<br>H00.0, H01, H03, H04.3, H05.0, H05.1, H06.1, H10, H13.1, H19.0-H19.3, H22.0, H32.0, H33.1C, H44.0, H44.1A, H45.1A-C, H48.1A, H48.1C, H60.0, H60.3, H62.0-4, H65, H66, H67.0, H67.1, H70.0, H70.1, H73.0A, H74.8C, H75.0, H91.8A4, H91.8A5,<br>I00-I02, I09.0-I09.2, I30, I31.0, I31.1, I31.3, I31.8B, I32.0, I32.1, I33, I39.8, I40, I41.0, I41.1, I41.2, I43.0, I51.8A, I51.8B, I52.0, I52.1, I52.8A, I68.1, I79.1A, I98.0, I98.1,<br>J00–J22, J32, J34.0, J35.0, J36, J37, J38.2A, J38.3C, J38.3D, J38.7B, J38.7G, J39.0, J39.1, J40-J42, J44.0, J85.0A, J85.1–J85.3, J86, J95.0A, J99.8A-J99.8C,<br>K02.0-K02.3, K02.5-K02.9, K04.0A, K04.6, K05.2A, K10.2, K11.2-K11.4, K12.2, K13.0A, K13.4A, K14.0A, K20.9A, K22.1C, K23.0, K29, K35, K36, K57, K61, K62.8N, K63.0, K65.0A-N, K67, K72.0A- K72.0C, K72.0E, K72.0F, K73, K75.0, K77.0, K81.0, K83.0C, K85.8A, K85.8E, K85.9A, K87.1A-K87.1B, K93.0,<br>L00–L08,<br>M00, M01, M03, M46.3, M49.0–M49.3, M60.0, M60.8A, M63.0-M63.2, M65, M68.0, M71.0, M71.1, M72.5A, M72.6, M73.0, M73.1, M86, M90.0-M90.2,<br>N08.0, N10.9A, N11, N12, N13.6A-E, N15.1, N16.0, N20.0I, N20.1I, N29.0, N29.1, N30, N33.0, N34, N37.0A, N39.0, N41.0–N41.3, N43.1, N45, N48.1–N48.2, N49.1–N49.2, N49.8A, N49.9, N51.0A–C, N51.1, N51.2A–B, N51.8B, N51.8C, N61, N70–N74, N75.0, N75.1, N75.8A-N75.8C, N76, N77, N98.0, N98.5,<br>O03.0, O04.0, O04.5, O07.0, O07.5, O08.0, O08.8D–F, O23, O26.4, O35.3, O36.8K-O36.8N, O41.1, O75.3, O85, O86, O91, O98, O00.2,<br>P23, P35–P39,<br>R09.3B, R57.2,<br>T79.3, T80.2, T81.4, T82.6–T82.7, T83.5–T83.6, T84.5–84.7, T85.7, T87.4, T88.0, T89,<br>Z22, Z83.0-Z83.1, Z86.1, Z92.8A
Any pulmonary infection (excl. COVID–19)	J00–J06, J09–J18, J20–22
Influenza	J09–11
Bacterial pneumonia	J12–18
Other pulmonary infection	J00–J06, J20–22

Table description: ICD-10 codes used to identify individuals hospitalized for any non-COVID-19 pulmonary infection. Note, that the ICD-10 codes G04-G05 are included in the outcome of *Encephalitis and Encephalopathy*. Thus, if this is part of the outcome, the codes are in practice not included in the *Any infection* group as individuals with pre-existing outcome disorders are excluded.

Supplementary Table 33: Dominating virus variant periods.

Dominating virus variant	Date from	Date to
Original	2020-03-01	2021-02-12
Alpha (B.1.1.7)	2021-02-13	2021-06-27
Delta (B.1.617.2)	2021-06-28	2021-12-18
Omicron (B.1.1.529)	2021-12-19	2022-06-12
Omicron subvariants (BA.5, XBB, etc.)[26]	2022-06-13	2023-06-30

Table description: The periods with the most dominant SARS-CoV-2 variants/pango lineages in Denmark are defined in this Supplementary Table.[27]

Supplementary Table 34: Lockdown periods.

Lockdown period	Date from	Date to
1st lockdown	2020-03-11	2020-04-19
1st inter lockdown	2020-04-20	2020-12-16
2nd lockdown	2020-12-17	2021-02-28
2nd inter lockdown	2021-03-01	2021-12-18
3rd lockdown	2021-12-19	2022-01-31
Post lockdowns	2022-02-01	2023-06-30

Table description: The periods with and without lockdowns in Denmark are defined in this Supplementary Table.[27]

Supplementary Figure 9: Timeline of lockdown and periods of dominating virus variants.

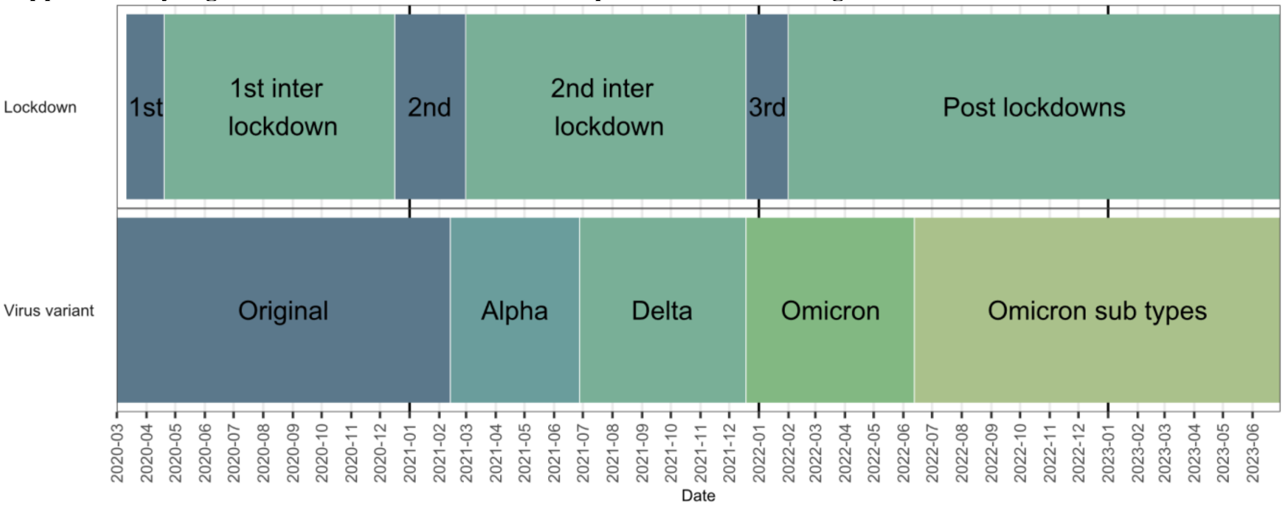

STROBE statement: Checklist of items that should be included in reports of cohort studies

	Item No	Recommendation	Page
**Title and abstract**	1	(*a*) Indicate the study's design with a commonly used term in the title or the abstract	2
		(*b*) Provide in the abstract an informative and balanced summary of what was done and what was found	2
**Introduction**			
Background/rationale	2	Explain the scientific background and rationale for the investigation being reported	3
Objectives	3	State specific objectives, including any prespecified hypotheses	4
**Methods**			
Study design	4	Present key elements of study design early in the paper	17
Setting	5	Describe the setting, locations, and relevant dates, including periods of recruitment, exposure, follow-up, and data collection	17-18
Participants	6	(*a*) Give the eligibility criteria, and the sources and methods of selection of participants. Describe methods of follow-up	17-19
		(*b*) For matched studies, give matching criteria and number of exposed and unexposed	NA
Variables	7	Clearly define all outcomes, exposures, predictors, potential confounders, and effect modifiers. Give diagnostic criteria, if applicable	17-19
Data sources/ measurement	8*	For each variable of interest, give sources of data and details of methods of assessment (measurement). Describe comparability of assessment methods if there is more than one group	*17-19*
Bias	9	Describe any efforts to address potential sources of bias	17-22
Study size	10	Explain how the study size was arrived at	17-18
Quantitative variables	11	Explain how quantitative variables were handled in the analyses. If applicable, describe which groupings were chosen and why	Supplementary Methods
Statistical methods	12	(*a*) Describe all statistical methods, including those used to control for confounding	21-22
		(*b*) Describe any methods used to examine subgroups and interactions	20-21
		(*c*) Explain how missing data were addressed	20
		(*d*) If applicable, explain how loss to follow-up was addressed	NA
		(*e*) Describe any sensitivity analyses	21
**Results**			
Participants	13*	(a) Report numbers of individuals at each stage of study—eg numbers potentially eligible, examined for eligibility, confirmed eligible, included in the study, completing follow-up, and analysed	5
		(b) Give reasons for non-participation at each stage	5
		(c) Consider use of a flow diagram	Suppl. Fig. 1

Descriptive data	14*	(a) Give characteristics of study participants (eg demographic, clinical, social) and information on exposures and potential confounders	Supplementary Table 2-4
		(b) Indicate number of participants with missing data for each variable of interest	Supplementary Table 2-4
		(c) Summarise follow-up time (eg, average and total amount)	Supplementary Table 2-4
Outcome data	15*	Report numbers of outcome events or summary measures over time	5
Main results	16	(*a*) Give unadjusted estimates and, if applicable, confounder-adjusted estimates and their precision (eg, 95% confidence interval). Make clear which confounders were adjusted for and why they were included	Sensitivity analysis 1
		(*b*) Report category boundaries when continuous variables were categorized	Supplementary Table 29
		(*c*) If relevant, consider translating estimates of relative risk into absolute risk for a meaningful time period	NA
Other analyses	17	Report other analyses done—eg analyses of subgroups and interactions, and sensitivity analyses	10-12
**Discussion**			
Key results	18	Summarise key results with reference to study objectives	12
Limitations	19	Discuss limitations of the study, taking into account sources of potential bias or imprecision. Discuss both direction and magnitude of any potential bias	15
Interpretation	20	Give a cautious overall interpretation of results considering objectives, limitations, multiplicity of analyses, results from similar studies, and other relevant evidence	16-17
Generalisability	21	Discuss the generalisability (external validity) of the study results	16
**Other information**			
Funding	22	Give the source of funding and the role of the funders for the present study and, if applicable, for the original study on which the present article is based	31

*Give information separately for exposed and unexposed groups.

Note: An Explanation and Elaboration article discusses each checklist item and gives methodological background and published examples of transparent reporting. The STROBE checklist is best used in conjunction with this article (freely available on the Web sites of PLoS Medicine at http://www.plosmedicine.org/, Annals of Internal Medicine at http://www.annals.org/, and Epidemiology at http://www.epidem.com/). Information on the STROBE Initiative is available at http://www.strobe-statement.org.

References

1. Nersesjan, V., Christensen, R. H. B., Kondziella, D. & Benros, M. E. COVID-19 and Risk for Mental Disorders Among Adults in Denmark. *JAMA psychiatry* 80, 778–786 (2023).

2. Momen, N. C. *et al.* Association between Mental Disorders and Subsequent Medical Conditions. *The New England journal of medicine* 382, 1721–1731 (2020).

3. Osler, M. *et al.* Hearing loss, cognitive ability, and dementia in men age 19-78 years. *European journal of epidemiology* 34, 125–130 (2019).

4. Grønkjær, C. S., Christensen, R. H. B., Kondziella, D. & Benros, M. E. Long-term neurological outcome after COVID-19 using all SARS-CoV-2 test results and hospitalisations in Denmark with 22-month follow-up. *Nature communications* 14, 4235 (2023).

5. Islamoska, S. *et al.* Mid- to late-life migraine diagnoses and risk of dementia: a national register-based follow-up study. *The journal of headache and pain* 21, 98 (2020).

6. Cohen, K. *et al.* Risk of persistent and new clinical sequelae among adults aged 65 years and older during the post-acute phase of SARS-CoV-2 infection: retrospective cohort study. *BMJ (Clinical research ed.)* 376, e068414 (2022).

7. McCormick, N., Bhole, V., Lacaille, D. & Avina-Zubieta, J. A. Validity of Diagnostic Codes for Acute Stroke in Administrative Databases: A Systematic Review. *PloS one* 10, e0135834 (2015).

8. Thygesen, S. K., Christiansen, C. F., Christensen, S., Lash, T. L. & Sørensen, H. T. The predictive value of ICD-10 diagnostic coding used to assess Charlson comorbidity index conditions in the population-based Danish National Registry of Patients. *BMC medical research methodology* 11, 83 (2011).

9. Skuladottir, A. T. *et al.* A meta-analysis uncovers the first sequence variant conferring risk of Bell's palsy. *Scientific reports* 11, 4188 (2021).

10. Schjødt, I., Nakano, A., Egstrup, K. & Cerqueira, C. The Danish Heart Failure Registry. *Clinical epidemiology* 8, 497–502 (2016).

11. Gilljam, T. *et al.* Development of heart failure in young patients with congenital heart disease: a nation-wide cohort study. *Open heart* 6, e000858 (2019).

12. Schmidt, M., Andersen, L. V., Friis, S., Juel, K. & Gislason, G. Data Resource Profile: Danish Heart Statistics. *International journal of epidemiology* 46, 1368-1369g (2017).

13. Bork, C. S., Al-Zuhairi, K. S., Hansen, S. M., Delekta, J. & Joensen, A. M. Accuracy of angina pectoris and acute coronary syndrome in the Danish National Patient Register. *Danish medical journal* 64, (2017).

14. Wan, E. Y. F. *et al.* Association of COVID-19 with short- and long-term risk of cardiovascular disease and mortality: a prospective cohort in UK Biobank. *Cardiovascular research* 119, 1718–1727 (2023).

15. Backer, V. *et al.* A register-based study: cough - a frequent phenomenon in the adult population. *BMC pulmonary medicine* 22, 426 (2022).

16. Nørgaard, M. *et al.* Irritable bowel syndrome and risk of colorectal cancer: a Danish nationwide cohort study. *British journal of cancer* 104, 1202–1206 (2011).

17. Pedersen, C. B. The Danish Civil Registration System. *Scandinavian Journal of Public Health* 39, 22–25 (2011).

18. Lynge, E., Sandegaard, J. L. & Rebolj, M. The Danish National Patient Register. *Scandinavian Journal of Public Health* 39, 30–33 (2011).

19. Jensen, V. M. & Rasmussen, A. W. Danish Education Registers. *Scandinavian journal of public health* 39, 91–94 (2011).

20. Baadsgaard, M. & Quitzau, J. Danish registers on personal income and transfer payments. *Scandinavian journal of public health* 39, 103–105 (2011).

21. Grove Krause, T., Jakobsen, S., Haarh, M. & Mølbak, K. The Danish Vaccination Register. *Euro Surveillance* 17, (2012).

22. Reilev, M. *et al.* Characteristics and predictors of hospitalization and death in the first 11 122 cases with a positive RT-PCR test for SARS-CoV-2 in Denmark: a nationwide cohort. *International Journal of*

Epidemiology 49, 1468–1481 (2020).

23. Jacobsen, P. A. *et al.* Return to work after COVID-19 infection – A Danish nationwide registry study. *Public Health* 203, 116–122 (2022).

24. Blichert-Hansen, L., Nielsson, M. S., Nielsen, R. B., Christiansen, C. F. & Nørgaard, M. Validity of the coding for intensive care admission, mechanical ventilation, and acute dialysis in the Danish National Patient Registry: a short report. *Clinical epidemiology* 5, 9–12 (2013).

25. Bodilsen, J. *et al.* Positive Predictive Value of ICD-10 Diagnosis Codes for COVID-19. *Clinical epidemiology* 13, 367–372 (2021).

26. State Serum Institute. Weekly trends: COVID-19 and other respiratory tract infections week 25 2022 (in Danish). https://www.ssi.dk/-/media/cdn/files/covid19/tendensrapport/rapport/ugentlige-tendenser-covid19-andre-luftvejs-uge25-2022-q3s5.pdf (2022).

27. State Serum Institute. Timeline for COVID-19 (in Danish). https://www.ssi.dk/-/media/arkiv/subsites/covid19/presse/tidslinje-over-covid-19/covid-19-tidslinje-for-2020-2022-lang-version---version-1---april-2022.pdf (2022).
