# Supplementary material for: Post-infection sequelae of COVID-19 and other infectious diseases—a nationwide Danish study with 40-month follow-up
Source: Nat Commun. 2026 Mar 13;17:3894. doi: 10.1038/s41467-026-70351-0 (PMC13125258; doi:10.1038/s41467-026-70351-0)
Supplement: Supplementary file 2 — Reporting Summary [file 41467_2026_70351_MOESM2_ESM.pdf]

nature portfolio

Corresponding author(s): Michael E. Benros

Last updated by author(s): Feb 3, 2026

Reporting Summary

Nature Portfolio wishes to improve the reproducibility of the work that we publish. This form provides structure for consistency and transparency in reporting. For further information on Nature Portfolio policies, see our Editorial Policies and the Editorial Policy Checklist.

Statistics

For all statistical analyses, confirm that the following items are present in the figure legend, table legend, main text, or Methods section.

n/a	Confirmed	
☐	☒	The exact sample size (*n*) for each experimental group/condition, given as a discrete number and unit of measurement
☐	☒	A statement on whether measurements were taken from distinct samples or whether the same sample was measured repeatedly
☐	☒	The statistical test(s) used AND whether they are one- or two-sided<br>*Only common tests should be described solely by name; describe more complex techniques in the Methods section.*
☐	☒	A description of all covariates tested
☐	☒	A description of any assumptions or corrections, such as tests of normality and adjustment for multiple comparisons
☐	☒	A full description of the statistical parameters including central tendency (e.g. means) or other basic estimates (e.g. regression coefficient) AND variation (e.g. standard deviation) or associated estimates of uncertainty (e.g. confidence intervals)
☐	☒	For null hypothesis testing, the test statistic (e.g. *F*, *t*, *r*) with confidence intervals, effect sizes, degrees of freedom and *P* value noted<br>*Give P values as exact values whenever suitable.*
☒	☐	For Bayesian analysis, information on the choice of priors and Markov chain Monte Carlo settings
☒	☐	For hierarchical and complex designs, identification of the appropriate level for tests and full reporting of outcomes
☒	☐	Estimates of effect sizes (e.g. Cohen's *d*, Pearson's *r*), indicating how they were calculated

Our web collection on statistics for biologists contains articles on many of the points above.

Software and code

Policy information about availability of computer code

Data collection	We used R, version 4.4.1. Packages used (version): survival (3.7-0), data.table (1.16.2), dplyr (1.1.4), lubridate (1.9.3), foreach (1.5.2), doParallel (1.0.17).
Data analysis	We used R, version 4.4.1. Additional packages used: emmeans (1.10.2). The code is available at https://github.com/Biological-and-Precision-Psychiatry/COVID-19-post-infection-sequelae.

For manuscripts utilizing custom algorithms or software that are central to the research but not yet described in published literature, software must be made available to editors and reviewers. We strongly encourage code deposition in a community repository (e.g. GitHub). See the Nature Portfolio guidelines for submitting code & software for further information.

Data

Policy information about availability of data

All manuscripts must include a data availability statement. This statement should provide the following information, where applicable:

- Accession codes, unique identifiers, or web links for publicly available datasets
- A description of any restrictions on data availability
- For clinical datasets or third party data, please ensure that the statement adheres to our policy

We used the following registers: the Microbiology Database, the Danish National Patient Register, the Danish Psychiatric Central Research Register, the Population Education Register, the Income Statistics Register, and the Danish National Prescription Register. The data are available from Statistics Denmark, https://

www.dst.dk/en/TilSalg/Forskningsservice/Dataadgang. To access health data in Denmark, applications must be submitted to the Danish Data Protection Agency, the Danish National Board of Health, and Statistics Denmark. For more information, see https://www.itgovernance.eu/ da-dk/eu-gdpr-compliance-dk, https://sundhedsdatastyrelsen.dk/da/english, and https://dst.dk/en.

Research involving human participants, their data, or biological material

Policy information about studies with human participants or human data. See also policy information about sex, gender (identity/presentation), and sexual orientation and race, ethnicity and racism.

Reporting on sex and gender	Analyses were adjusted for sex, which was the information available in the data. This was stated in the pre-registered protocol.
Reporting on race, ethnicity, or other socially relevant groupings	No information on race, ethnicity, or other socially relevant groupings was available.
Population characteristics	When considering mental disorders as the outcome, the study population consisted of 5,147,288 individuals, and when considerend general medical conditions as the outcome, the study population consisted of 4,074,487 individuals. In the study population, 48.9% were female, and the mean age at start was 33.4 (standard deviation 21.2) years.
Recruitment	All individuals in the population of Denmark without pre-existing mental health disorders or general medical conditions. This was stated in the pre-registered protocol.
Ethics oversight	This study was approved by the Danish Data Protection Agency and the Danish Health and Medicine Authority. According to Danish legislation, no further ethical approval or informed consent is required for register-based studies.

Note that full information on the approval of the study protocol must also be provided in the manuscript.

Field-specific reporting

Please select the one below that is the best fit for your research. If you are not sure, read the appropriate sections before making your selection.

☒ Life sciences ☐ Behavioural & social sciences ☐ Ecological, evolutionary & environmental sciences

For a reference copy of the document with all sections, see nature.com/documents/nr-reporting-summary-flat.pdf

Life sciences study design

All studies must disclose on these points even when the disclosure is negative.

Sample size	To attain the most precise estimates, the entire population of Denmark without pre-existing mental health disorders or general medical conditions were included. When considering mental disorders as the outcome, the study population consisted of 5,147,288 individuals, and when considerend general medical conditions as the outcome, the study population consisted of 4,074,487 individuals.
Data exclusions	Individuals with pre-existing mental health disorders or general medical conditions were excluded as we only considered new onset disorders. This was stated in the pre-registered protocol.
Replication	The findings were not replicated as there was no external dataset with appropriate information available.
Randomization	Participants were not allocated into experimental groups.
Blinding	Since the study was observational, blinding was not possible.

Reporting for specific materials, systems and methods

We require information from authors about some types of materials, experimental systems and methods used in many studies. Here, indicate whether each material, system or method listed is relevant to your study. If you are not sure if a list item applies to your research, read the appropriate section before selecting a response.

Materials & experimental systems

n/a	Involved in the study
☒	☐ Antibodies
☒	☐ Eukaryotic cell lines
☒	☐ Palaeontology and archaeology
☒	☐ Animals and other organisms
☒	☐ Clinical data
☒	☐ Dual use research of concern
☒	☐ Plants

Methods

n/a	Involved in the study
☒	☐ ChIP-seq
☒	☐ Flow cytometry
☒	☐ MRI-based neuroimaging

Plants

Seed stocks	*Report on the source of all seed stocks or other plant material used. If applicable, state the seed stock centre and catalogue number. If plant specimens were collected from the field, describe the collection location, date and sampling procedures.*
Novel plant genotypes	*Describe the methods by which all novel plant genotypes were produced. This includes those generated by transgenic approaches, gene editing, chemical/radiation-based mutagenesis and hybridization. For transgenic lines, describe the transformation method, the number of independent lines analyzed and the generation upon which experiments were performed. For gene-edited lines, describe the editor used, the endogenous sequence targeted for editing, the targeting guide RNA sequence (if applicable) and how the editor was applied.*
Authentication	*Describe any authentication procedures for each seed stock used or novel genotype generated. Describe any experiments used to assess the effect of a mutation and, where applicable, how potential secondary effects (e.g. second site T-DNA insertions, mosiacism, off-target gene editing) were examined.*
